# Asymmetric Total Synthesis of Illisimonin A

**DOI:** 10.1021/jacs.3c01262

**Published:** 2023-03-16

**Authors:** Christoph Etling, Giada Tedesco, Anna Di Marco, Markus Kalesse

**Affiliations:** Institute of Organic Chemistry, Leibniz Universität Hannover, Schneiderberg 1B, 30167 Hannover, Germany

## Abstract

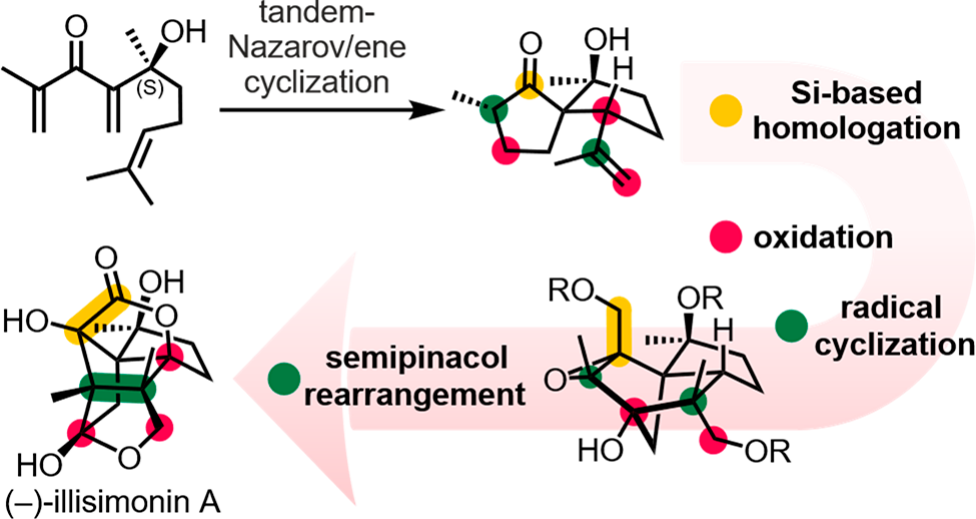

The discovery of illisimonin A in 2017 extended the structural repertoire of the *Illicium* sesquiterpenoids—a class of natural products known for their high oxidation levels and neurotrophic properties—with a new carbon backbone combining the strained *trans*-pentalene and norbornane substructures. We report an asymmetric total synthesis of (−)-illisimonin A that traces its tricyclic carbon framework back to a spirocyclic precursor, generated by a tandem-Nazarov/ene cyclization. As crucial link between the spirocyclic key intermediate and illisimonin A, a novel approach for the synthesis of tricyclo[5.2.1.0^1,5^]decanes via radical cyclization was explored. This approach was applied in a two-stage strategy consisting of Ti(III)-mediated cyclization and semipinacol rearrangement to access the natural product’s carbon backbone. These key steps were combined with carefully orchestrated C–H oxidations to establish the dense oxidation pattern.

## Introduction

The plants of the genus *Illicium* have been known for decades as a rich source of sesquiterpenoid natural products, notorious for their highly oxidized, polycyclic structures as well as potent neurotrophic properties. Since the isolation of their first member, anisatin, in 1952,^1^ the *Illicium* sesquiterpenoids have grown into a large, structurally diverse collection of natural products with over 100 members isolated from more than 40 species.^2^ Seminal work by the Fukuyama group showed that members of the *Illicium* sesquiterpenoids act as potent promotors of neurite outgrowth,^3^ which inspired a large number of total syntheses.^4^ These established access to several congeners of the three largest subclasses, categorized by their carbon backbones as *seco*-prezizaanes,^5^*allo*-cedranes,^6^ or anislactone-type sesquiterpenoids^7^ and built the foundation for deeper studies of the neurotrophic properties of these natural products.^8^

Despite the large number of known members, novel carbon backbones for *Illicium*-derived sesquiterpenoids have been discovered recently, posing new synthetic challenges.^9^ As a prominent example, illisimonin A (**1**), which was isolated from the fruits of *Illicium simonsii*, features the unprecedented “illisimonane skeleton”.^9a^ This unique backbone is based on a bridged tricyclo[5.2.1.0^1,5^]decane ring system, which combines two distinctly strained motifs, a *trans*-pentalene and a norbornane substructure.^10^

**Scheme 1:**
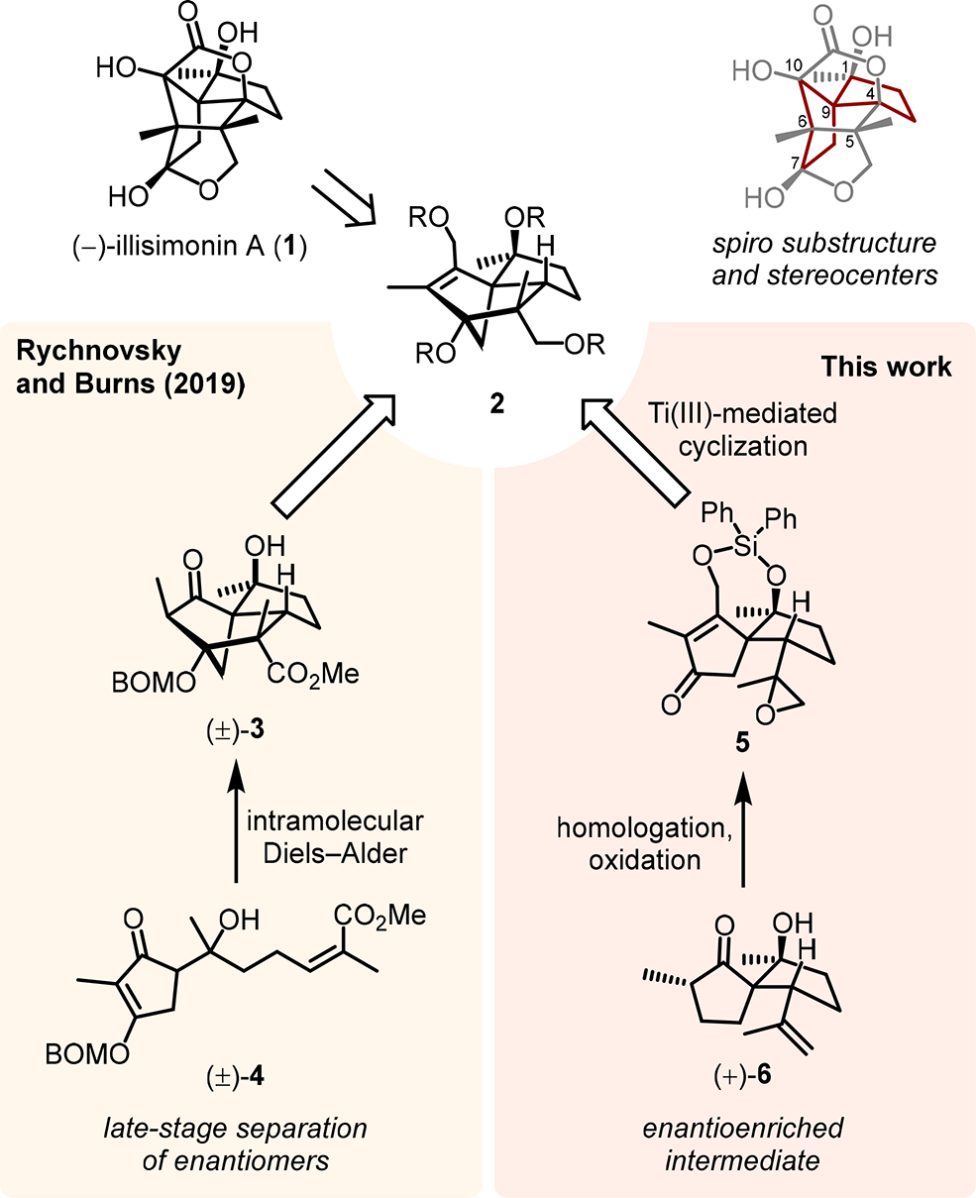
Structure of (−)-Illisimonin A (**1**) and Approaches to Enantioenriched Tricyclo[5.2.1.0^1,5^]decane **2** BOM = benzyloxymethyl.

The ring system is additionally bridged by a γ-lactone and a γ-lactol ring, resulting in a cage-like 5/5/5/5/5 pentacyclic structure. Illisimonin A’s carbon backbone and oxidation pattern add up to a total of seven contiguous fully substituted stereocenters, three of which (C5, C6, and C9) are quaternary centers, making it an appealing, yet challenging target for total synthesis (Scheme 1).

Furthermore, illisimonin A shows neuroprotective effects against oxygen–glucose deprivation-induced cell injury in SH-SY5Y cells, making it a potential candidate for the development of drugs against neurodegenerative diseases.^9a^

In the five years since its discovery, the challenging structure and promising bioactivity have prompted several groups to develop methods to access its structural motifs,^11^ to explore strategies for backbone construction,^12^ and to investigate the potential biosynthetic origin of this molecule.^13^ Yet, only one total synthesis of illisimonin A has been accomplished so far.^12a^ Rychnovsky and Burns accessed the strained carbon backbone of the natural product via a semipinacol rearrangement of precursor **2**, whose tricyclo[5.2.1.0^1,5^]decane backbone was obtained through an intramolecular Diels–Alder reaction (Scheme 1).^12a^ Given the fact that their approach was not enantioselective, chiral resolution at an advanced stage was necessary to obtain enantioenriched (−)-illisimonin A (**1**), allowing them to revise the initially proposed absolute configuration.^9a,12a^

In this work, we present an asymmetric total synthesis of the natural enantiomer of illisimonin A (**1**), following a new approach for the synthesis of enantioenriched tricyclo[5.2.1.0^1,5^]decane **2** (generalized structure, Scheme 1).

## Results and Discussion

**Scheme 2:**
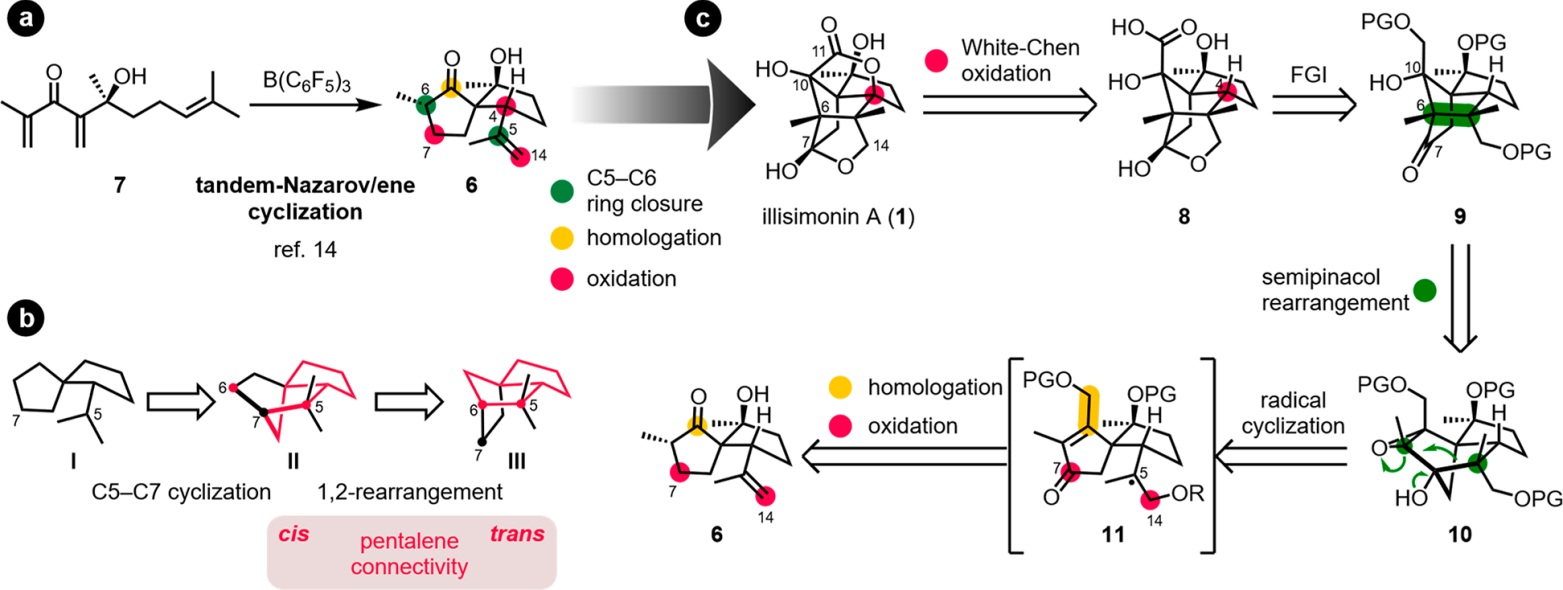
(a) Access to Spirocyclic Ketones via the Tandem-Nazarov/Ene Cyclization, (b) C5–C6 Bond Formation Strategy, and (c) Retrosynthetic Analysis FGI = functional group interconversion, PG = protecting group.

The foundation for our approach toward illisimonin A was laid by the discovery of the tandem-Nazarov/ene cyclization that produces carbocyclic spiro compounds from linear precursors like **7** in a single, diastereoselective transformation (Scheme 2a).^14^ Spiro ketone **6** perfectly maps out the spiro substructure hidden inside the natural product’s cage-like ring system and already contains 14 of the sesquiterpenoid’s 15 carbon atoms (Scheme 1).

From **6**, the synthesis faced three main challenges: (I) formation of the missing C5–C6 bond (green), (II) introduction of the carboxylic carbon C11 (yellow), and (III) oxidation of three positions (C4, C7, and C14, red, Scheme 2a).

For the formation of the crucial C5–C6 bond, we envisioned a two-step strategy consisting of an initial C5–C7 cyclization, followed by a 1,2-rearrangement to establish the desired connectivity (Scheme 2b). We chose this approach based on a comparison of the ring connectivity and strain between tricycles **II** and **III**, which can be traced back to early considerations by Corey and was applied 50 years later by Rychnovsky and Burns:^12a,15^ The tricyclo[5.2.1.0^1,5^]decane carbon framework of illisimonin A (**III**, Scheme 2b), which features a *trans*-fused pentalene substructure, is about 7 kcal·mol^–1^ higher in energy than its isomer **II**, featuring the *cis*-fused counterpart as analyzed by Rychnovsky and Burns.^12a,15^ Consequently, we reasoned that the cyclization of spirocyclic framework **I** to tricyclo[5.2.1.0^1,5^]decane **II** (C5–C7 cyclization) would be energetically more favorable than cyclization to **III** (C5–C6 cyclization), as its ring system would have to overcome less ring strain for the reacting centers to come into close proximity. The combination of the C5–C7 ring closure with a subsequent 1,2-rearrangement would then allow the transition from the *cis-* to the *trans*-pentalene substructure as well as the construction of the two contiguous quaternary centers (C5 and C6) of illisimonin A in one step (Scheme 2b).

The synthesis of tricyclo[5.2.1.0^1,5^]decanes like **II** is well established, with common approaches being intramolecular Diels–Alder reactions,^15,16^ rearrangements, ring contractions,^12b,17^ or single bond disconnections starting from fused bicyclic systems and norbornanes.^18^

To the best of our knowledge, the disconnection to a spirocyclic precursor, instead, is unprecedented. The structural simplicity of the Nazarov cyclization precursor **7** further offered an intriguing chance for an asymmetric entry, since only one stereocenter, i.e., the tertiary alcohol of **7**, has to be set at an early stage of the synthesis (Scheme 2a). We considered this aspect particularly valuable, as only one method for the asymmetric synthesis of tricyclo[5.2.1.0^1,5^]decanes has been described so far.^11b^

Our considerations led to the following retrosynthesis: the γ-lactone moiety of **1** was first disconnected to carboxylic acid **8** via White–Chen oxidation of the C4 methine group. **8** was traced back to intermediate **9** with the intact carbon backbone of the natural product. As demonstrated by Rychnovsky and Burns for a related substrate,^12a^**9** should be accessible via a semipinacol rearrangement from epoxyalcohol **10**. This rearrangement would not only allow the construction of the carbon backbone of illisimonin A from a less strained and more readily accessible predecessor but would also offer the advantage that the 1,3-aldol motif (C6–C7–C10, compare **1** and **9**, Scheme 2c) can be introduced in a stepwise manner. Therefore, functional complexity prior to this stage could be reduced, which would be crucial for the design of an appropriate precursor for **10**. The tricyclo[5.2.1.0^1,5^]decane backbone of **10** was envisioned to be formed via radical cyclization of a reactive intermediate as **11**, bearing a carbonyl group as a radical acceptor at C7. We considered metal-hydride hydrogen atom transfer (MHAT)-based methods as well as a Ti(III)-mediated reductive epoxide opening to be viable options to generate the tertiary radical at C5. The cyclization precursor was traced back to spirocycle **6** through one-carbon homologation and oxidation of C7 and C14 (Scheme 2c).

We started the asymmetric synthesis from literature-known propargylic alcohol **12**, available in three steps from geraniol with 91% *ee*.^19^ A nickel-catalyzed hydrocyanation using Liu’s method^20^ proceeded with excellent Markovnikov selectivity to build up the central 1,1-disubstituted double bond of the Nazarov cyclization precursor **7.**

**Scheme 3:**
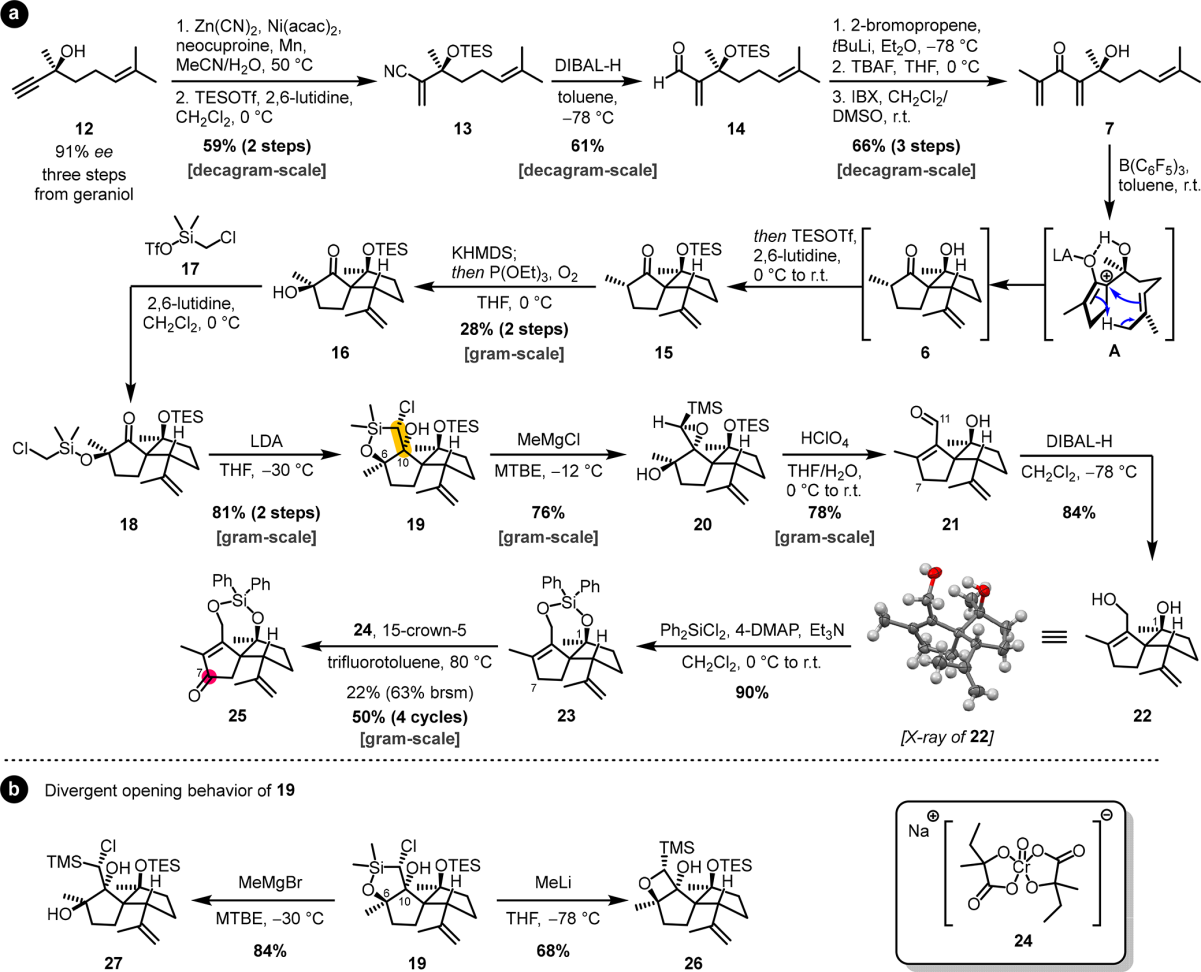
(a) Asymmetric Synthesis of Spirocyclic Key Intermediate **15** and Further Derivatization via a Silicon-Based One-Carbon Homologation and C7 Oxidation and (b) Divergent Opening Behavior of Oxasilolane **19** acac = acetylacetonate, brsm = based on recovered starting material, DIBAL-H = diisobutylaluminum hydride, 4-DMAP = 4-dimethylaminopyridine, DMSO = dimethyl sulfoxide, IBX = 2-iodoxybenzoic acid, KHMDS = potassium bis(trimethylsilyl)amide, LDA = lithium diisopropylamide, MTBE = methyl *tert*-butyl ether, r.t. = room temperature, TBAF = tetra-*n*-butylammonium fluoride, TES = triethylsilyl, Tf = trifluoromethanesulfonyl, THF = tetrahydrofuran.

The low reactivity of the obtained acrylonitrile toward 1,2-addition made it necessary to protect the tertiary alcohol and reduce the nitrile to aldehyde **14**, which smoothly underwent reaction with isopropenyl lithium to give enantioenriched Nazarov cyclization precursor **7** after TES deprotection and IBX oxidation (Scheme 3a). The highly scalable sequence allowed the preparation of decagram quantities of **7**, which built a solid foundation for the challenging ring construction to follow.

From **7**, spirocyclic key intermediate **6** was accessible through a B(C_6_F_5_)_3_-catalyzed tandem-Nazarov/ene cyclization.^14^ The yield of cyclization product **6** had shown to drop during scale-up experiments, an observation we attributed to a potential retro-aldol reaction during purification on silica. A one-pot procedure with concomitant TES protection was therefore applied on large scale, affording protected spirocycle **15** as a key intermediate for our synthetic endeavor (Scheme 3a). TES deprotection and reprotection in the sequence from **14** to **15** could not be avoided, as the TES-protected derivative of **7** did not deliver desired spirocycle **15** under our Nazarov cyclization conditions.

Preliminary investigations on the one-carbon homologation had shown that unprotected spirocycle **6** mainly underwent decomposition when attempting 1,2-addition of metal organyls or homologation via hydrazone-based reactions (such as Barton’s hydrazone iodination or the Shapiro reaction). In contrast, TES-protected congener **15** proved to be virtually unreactive. All attempts to address the carbonyl moiety of **15**, including 1,2-addition (metal organyls, hydrazine derivatives, or cyanide), olefination, or vinyl triflate formation for a cross-coupling-based approach, failed. A silicon-tethered Barbier reaction, using the tertiary alcohol of **6** as an anchor, was also unsuccessful.

**15** was, however, susceptible to α-oxidation with molecular oxygen to deliver α-hydroxy ketone **16** in 26% yield starting from Nazarov cyclization precursor **7**. The α-oxidation proved to be a crucial derivatization for two reasons: it rendered the carbonyl function nonenolizable and offered the α-hydroxy group as an anchor point for tethering of a nucleophile. Inspired by Zhou’s bifunctional cyanosilylation reagent,^21^ we used silyltriflate **17** to prepare chloromethyl silyl ether **18**, which underwent cyclization to oxasilolane **19** in good yield after deprotonation with LDA (Scheme 3a). After finding a way of introducing the missing skeletal carbon atom of illisimonin A, we faced the problem of how to convert oxasilolane **19** into a useful intermediate for further manipulation. With a Peterson olefination-type reaction in mind, we decided to open the silacycle to allow the required elimination.

A screening of reaction conditions employing nucleophilic methyl sources revealed that, under most reaction conditions, the Si–O bond scission was followed by intramolecular nucleophilic displacement of the chloride by one of the adjacent hydroxyl groups (C6 or C10), leading either to TMS-epoxide **20** or oxetane **26** (Scheme 3a,b). We found that the mode of cyclization was strongly dependent on the combination of solvent, counterion, and reaction temperature. For example, the use of MeLi in THF favored the formation of oxetane **26** (Scheme 3b), while the use of the respective Grignard reagents in MTBE or CPME favored formation of TMS-epoxide **20** at temperatures around 0 °C. Lower reaction temperatures allowed the isolation of chlorohydrin **27** (Scheme 3b). The reaction conditions were optimized toward TMS-epoxide **20**, since we considered it a suitable precursor for an aldehyde or enal. Best results were obtained with MeMgCl in MTBE at −12 °C (Scheme 3a, for details, see the Supporting Information).

Acidic opening of TMS-epoxide **20** under standard conditions^22^ proceeded concomitantly with TES deprotection and afforded enal **21** in good yield. The one-carbon homologation was completed with introduction of an oxygen functionality at C11, and an endocyclic double bond, making C7 accessible to the pending allylic oxidation. To circumvent chemoselectivity issues in the subsequent allylic oxidation and radical cyclization, aldehyde **21** was reduced to diol **22**, and both hydroxyl groups were protected using Ph_2_SiCl_2_ (Scheme 3a). The use of a bidentate protecting group was key to access the tertiary alcohol at C1.

Examination of reaction conditions for the subsequent allylic oxidation showed that C7 was the most reactive allylic position of diene **23** under H-abstraction-based conditions. However, extensive decomposition and low yields for enone **25** were observed under most conditions (for details, see the Supporting Information). We were able to render this transformation synthetically facile by using Cr(V) complex **24** under a variation of Baran’s conditions,^23^ which gave enone **25** in a reasonable yield of 50% (after four cycles with reisolation of starting material) (Scheme 3a). The introduction of the keto group at C7 set the stage for the envisioned radical cyclization.

**Scheme 4:**
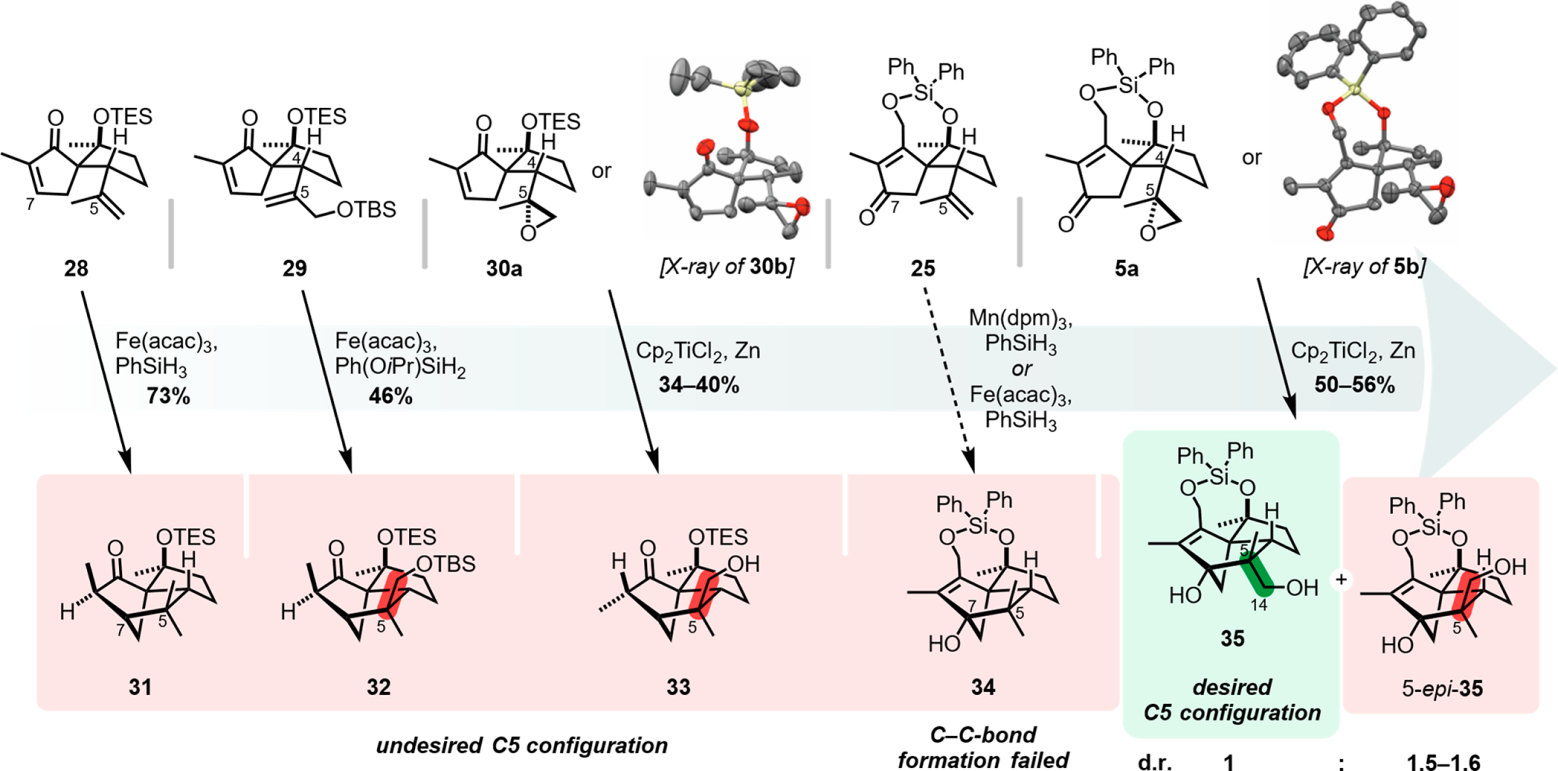
Evolution of the Tricyclo[5.2.1.0^1,5^]decane Synthesis—Studies on the C5 Stereocontrol for Radical-Based C5–C7 Cyclizations Cp = cyclopentadienyl, dpm = 2,2,6,6-tetramethyl-3,5-heptanedionate, TBS = *tert*-butyldimethylsilyl.

At the beginning of our investigations, we wanted to probe if the molecular geometry of our spirocyclic intermediates allowed a radical cyclization between C5 and C7 and to which degree the substrate would exert stereocontrol over the newly formed C5 quaternary center. For this purpose, substrates **5**, **25**, and **28**–**30** were examined using MHAT- or Ti(III)-based methods to initiate cyclization (Scheme 4).

We started with easily accessible enones **28**–**30**, aiming at a Giese-type cyclization. As proof-of-principle, simple dienone **28** underwent smooth cyclization to tricyclodecane **31** under Baran’s conditions.^24b^ For enones **29** and **30**, MHAT- and Ti(III)-mediated cyclization resulted in exclusive formation of tricycles **32** and **33** with the undesired C5 stereoconfiguration (Scheme 4).^24a,25b^ For **30**, the C5 configuration of the product was independent of the diastereomer (**30a** or **30b**) used, which is in accordance with reports by Chiara and co-workers on related cyclizations.^25c^

The observed stereochemical outcome could be explained through minimization of steric interactions between the substituents of the side chain and the adjacent ring during cyclization. We propose that in these cases, the large OR residue (R = TBS or Cp_2_TiCl) prefers an orientation *syn* to the methine proton at C4, leading to the undesired C5 configuration.

As the envisioned semipinacol rearrangement demanded a hydroxyl function at C7, we expanded the study to substrates bearing a carbonyl function as an acceptor moiety at this position. Inspired by Bonjoch and Bradshaw’s work on MHAT-initiated radical cyclizations of alkene-tethered ketones,^24c^ we tested the cyclization of dienone **25** (Scheme 4). The examined MHAT conditions, however, failed to deliver the desired product **34**. We reasoned that the failure of the reaction might be attributed to fragmentation of the intermediately formed alkoxy radical,^24c,26^ potentially driven by release of the norbornene ring strain. This result, together with the undesired C5 configuration observed for **29**, ruled out a MHAT-mediated cyclization as a viable option for the synthesis.

We were finally able to access tricycle **35** with the desired C5 configuration via the Ti(III)-mediated cyclization of epoxy enones **5a** and **5b**. We deemed this an extraordinary result, as the previous experiments had suggested that the spirocyclic scaffold favored the formation of products with the undesired C5 configuration (Scheme 4). However, the reaction did not show a pronounced selectivity for **35** and afforded the desired product as a mixture with diastereomer 5-*epi*-**35** in a ratio of 1:1.5–1.6, independent of the epoxide used (Scheme 4). The diastereoselectivity could not be increased in favor of **35** during our investigations. The best results were obtained with Bermejo’s inverse addition protocol (addition of epoxide solution to Cp_2_TiCl solution).^25b^ Attempts to render the cyclization catalytic using Gansäuer’s method failed in our hands.^25a^

We propose that in the case of **5** the formation of major diastereomer 5-*epi*-**35** is favored by minimization of steric interactions as outlined above. In contrast, formation of **35** could be the result of a competing process with coordination of the titanium complex to both the former epoxide oxygen and the carbonyl group, orienting the hydroxymethyl group (C14) toward the carbonyl group. This chelation had also been proposed by Bermejo and co-workers in the case of epoxy-carvone.^25b^

**Scheme 5:**
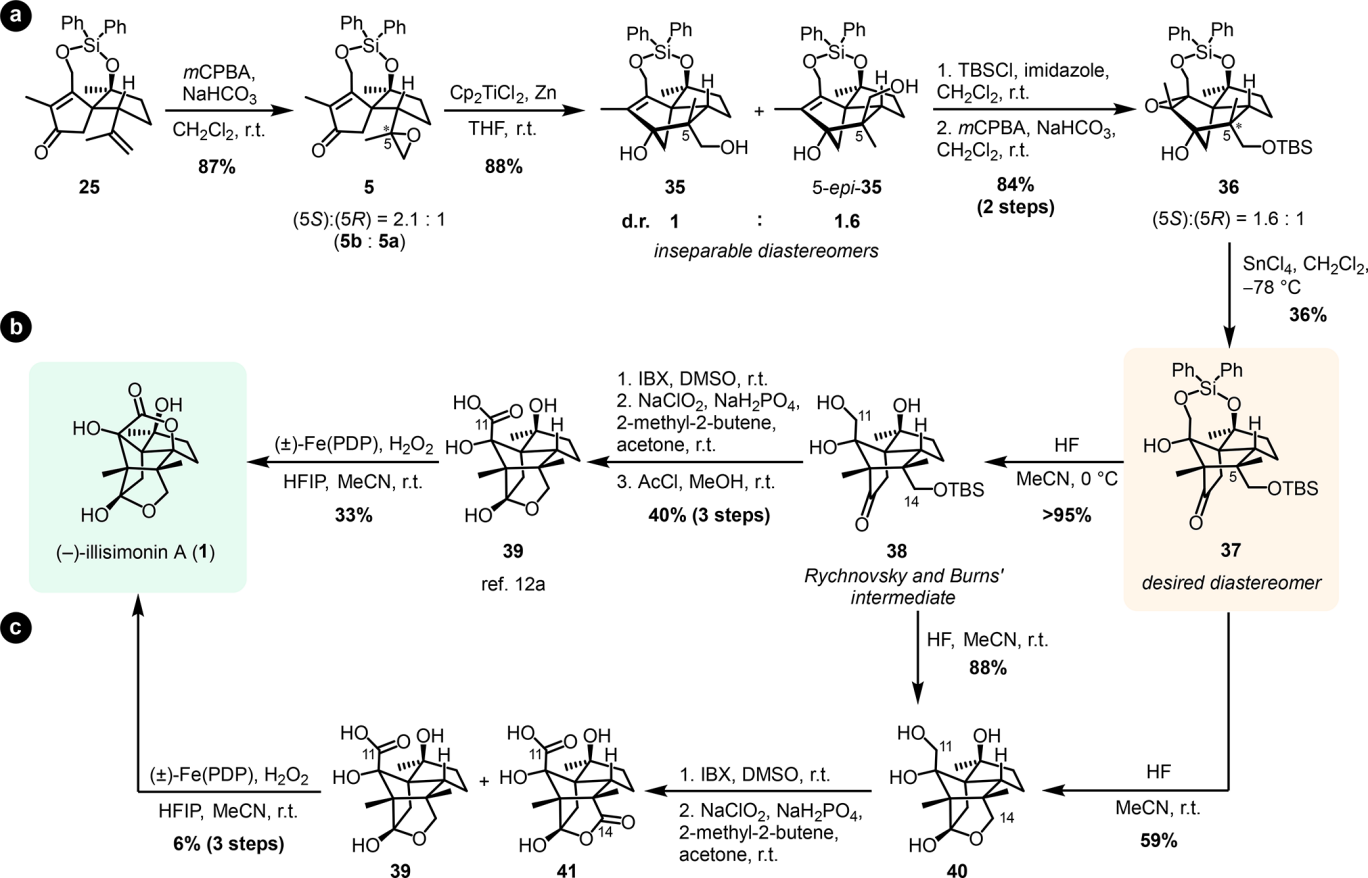
(a) Access to the Carbon Framework of Illisimonin A via Our Cyclization/Rearrangement Strategy, (b) Completion of the Total Synthesis via Rychnovsky and Burns’ Route, and (c) Exploration of an Alternative Endgame *m*CPBA = *meta*-chloroperoxybenzoic acid, HFIP = 1,1,1,3,3,3-hexafluoropropan-2-ol, PDP = [*N*,*N*′-bis(2-pyridylmethyl)]-2,2′-bipyrrolidine.

These insights were transferred to our approach to illisimonin A (**1**). The diastereomeric mixture of epoxide **5**, obtained by epoxidation of the isopropenyl moiety of **25**, was subjected to reductive cyclization conditions using stoichiometric amounts of Cp_2_TiCl. The reaction reliably afforded a mixture of **35** and 5-*epi*-**35** in high yield with a d.r. of 1:1.6 (Scheme 5).

The primary alcohol was TBS-protected, and the double bond was epoxidized to set the stage for the pivotal semipinacol rearrangement. In contrast to the substrate reported by Rychnovsky and Burns,^12a^ epoxy alcohol **36** did not undergo rearrangement under Brønsted acidic conditions. Instead, the use of Lewis acids proved to be crucial, and exposure of **36** to SnCl_4_ successfully triggered the rearrangement. The C5 epimers were separable at this stage, allowing the isolation of **37** with the desired (5*S*)-configuration in 36% yield. Considering that **36** was used as a diastereomeric mixture, the rearrangement proceeded with high efficiency for the desired diastereomer in a yield of over 90% based on (5*R*)-**36**.

The undesired diastereomer 5-*epi-***37** could be isolated as side product in low yield, which suggested that the semipinacol rearrangement of (5*S*)-**36** may not proceed as efficiently or that 5-*epi-***37** underwent decomposition under the given conditions. Without protection of **35**, only low yields and difficulties with reproducibility were observed.

Continuing from **37**, we explored the endgame toward illisimonin A. With the intention to refrain from stepwise removal of the diphenyl silyl and the TBS-protecting group, we performed global deprotection to tetraol **40**, using aqueous HF in acetonitrile at room temperature (Scheme 5c). **40** was planned to be oxidized to carboxylic acid **39**, which had been reported before. However, the majority of examined one- and two-step procedures for the conversion of alcohol to carboxylic acid failed to deliver desired acid **39**.^27^ Only with a stepwise combination of IBX and Pinnick oxidation^12a^ were we able to observe the formation of **39** as a mixture with carboxylic acid **41**, featuring additional oxidation at C14. A subsequent White–Chen oxidation according to the protocol by Burns and Rychnovsky allowed the synthesis of (−)-illisimonin A (**1**), albeit with low yield (Scheme 5c).^12a,28^

Realizing that the competing C14 oxidation was a significant drawback of the route proceeding through **40**, we turned efforts toward reproduction of the literature endgame, during which the C14 hydroxyl group remained TBS-protected for the duration of the C11 oxidation. Thus, the Ph_2_Si-protecting group of **37** was selectively removed using aqueous HF at 0 °C to gain access to common intermediate **38** in excellent yield. From **38**, pure carboxylic acid **39** could be synthesized in 40% yield over three literature-known steps. A White–Chen oxidation afforded (−)-illisimonin A (**1**) in 33% yield (Scheme 5b).^12a^ The NMR spectroscopic data and circular dichroism of the synthetic material were in agreement with the published data.^9a^

## Conclusion

In conclusion, we accomplished the first asymmetric total synthesis of (−)-illisimonin A (**1**), using a spirocyclic scaffold generated by the tandem-Nazarov/ene cyclization as a template for the successive construction of the natural product’s strained carbon backbone. It thus represents the second example of an interrupted Nazarov cyclization in total synthesis.^29^

As a crucial link between the backbone of illisimonin A (**1**) and our spirocyclic core intermediate **6**, a novel approach for the synthesis of tricyclo[5.2.1.0^1,5^]decanes via radical cyclization of spirocyclic precursors was explored. Investigations on the stereocontrol of these cyclizations showed that a Ti(III)-mediated epoxide-ketone coupling was the only method capable of delivering the product with the desired relative configuration. The carbon backbone of illisimonin A was accessed via a semipinacol rearrangement, which allowed the facile transition between a *cis*- and *trans*-pentalene substructure within the tricyclo[5.2.1.0^1,5^]decane framework. We finally explored an alternative to the previously reported endgame, demonstrating that this shorter sequence was capable to deliver (−)-illisimonin A. The endgame reported by Rychnovsky and Burns, however, remains more viable.

Our robust synthesis allowed the preparation of a total of 200 mg of enantioenriched intermediate **38** in 0.3% yield over 21 steps from literature-known propargylic alcohol **12**. **38** could be converted to (−)-illisimonin A (**1**) in three literature-known steps. Together with the extraordinary efforts of other groups, this work will contribute to making illisimonin A and structurally related compounds available for deeper investigations into their neurotrophic properties.

## Supporting Information Available

General methods; experimental procedures and spectral data; comparison of synthetic and natural illisimonin A; ^1^H and ^13^C NMR spectra; X-ray crystallographic data (PDF)

## References

[ref1] Lane J. F.; Koch W. T.; Leeds N. S.; Gorin G. On the Toxin of *Illicium Anisatum*. I. The Isolation and Characterization of a Convulsant Principle: Anisatin. J. Am. Chem. Soc. 1952, 74, 3211–3215. 10.1021/ja01133a002.

[ref2] Fukuyama Y.; Huang J. M. Chemistry and neurotrophic activity of *seco*-prezizaane- and anislactone-type sesquiterpenes from *Illicium* species. Stud. Nat. Prod. Chem. 2005, 32, 395–427. 10.1016/S1572-5995(05)80061-4.

[ref3] bKubo M.; Okada C.; Huang J. M.; Harada K.; Hioki H.; Fukuyama Y. Novel Pentacyclic *seco*-Prezizaane-Type Sesquiterpenoids with Neurotrophic Properties from *Illicium jiadifengpi*. Org. Lett. 2009, 11, 5190–5193. 10.1021/ol9021029. 19873982

[ref4] aUrabe D.; Inoue M. Total syntheses of sesquiterpenes from *Illicium* species. Tetrahedron 2009, 65, 6271–6289. 10.1016/j.tet.2009.06.010.

[ref5] bHung K.; Condakes M. L.; Novaes L. F. T.; Harwood S. J.; Morikawa T.; Yang Z.; Maimone T. J. Development of a Terpene Feedstock-Based Oxidative Synthetic Approach to the *Illicium* Sesquiterpenes. J. Am. Chem. Soc. 2019, 141, 3083–3099. 10.1021/jacs.8b12247. 30698435 PMC6563921

[ref6] bMehta G.; Maity P. A total synthesis of 11-*O*-methyldebenzoyltashironin. Tetrahedron Lett. 2011, 52, 1749–1752. 10.1016/j.tetlet.2011.02.012.

[ref7] bShi L.; Meyer K.; Greaney M. F. Synthesis of (±)-Merrilactone A and (±)-Anislactone A. Angew. Chem., Int. Ed. 2010, 49, 9250–9253. 10.1002/anie.201005156. 20967815

[ref8] aCarcache D. A.; Cho Y. S.; Hua Z.; Tian Y.; Li Y. M.; Danishefsky S. J. Total Synthesis of (±)-Jiadifenin and Studies Directed to Understanding Its SAR: Probing Mechanistic and Stereochemical Issues in Palladium-Mediated Allylation of Enolate-Like Structures. J. Am. Chem. Soc. 2006, 128, 1016–1022. 10.1021/ja056980a. 16417394

[ref9] aMa S.-G.; Li M.; Lin M.-B.; Li L.; Liu Y.-B.; Qu J.; Li Y.; Wang X.-J.; Wang R.-B.; Xu S.; Hou Q.; Yu S.-S. Illisimonin A, a Caged Sesquiterpenoid with a Tricyclo[5.2.1.0^1,6^]decane Skeleton from the Fruits of *Illicium simonsii*. Org. Lett. 2017, 19, 6160–6163. 10.1021/acs.orglett.7b03050. 29077414

[ref10] aAllinger N. L.; Hirsch J. A.; Miller M. A.; Tyminski I. J.; Van-Catledge F. A. Conformational analysis. LX. Improved calculations of the structures and energies of hydrocarbons by the Westheimer method. J. Am. Chem. Soc. 1968, 90, 1199–1210. 10.1021/ja01007a017.

[ref11] aRiveira M. J.; Marcarino M. O.; La-Venia A. Multicomponent Domino Synthesis of Cyclopenta[b]furan-2-ones. Org. Lett. 2018, 20, 4000–4004. 10.1021/acs.orglett.8b01567. 29874085

[ref12] aBurns A. S.; Rychnovsky S. D. Total Synthesis and Structure Revision of (−)-Illisimonin A, a Neuroprotective Sesquiterpenoid from the Fruits of *Illicium simonsii*. J. Am. Chem. Soc. 2019, 141, 13295–13300. 10.1021/jacs.9b05065. 31408328

[ref13] McCulley C. H.; Tantillo D. J. Predicting Rearrangement-Competent Terpenoid Oxidation Levels. J. Am. Chem. Soc. 2020, 142, 6060–6065. 10.1021/jacs.9b12398. 32157874

[ref14] Etling C.; Tedesco G.; Kalesse M. A Nazarov-Ene Tandem Reaction for the Stereoselective Construction of Spiro Compounds. Chem. Eur. J. 2021, 27, 9257–9262. 10.1002/chem.202101041. 33899295 PMC8361765

[ref15] Corey E. J.; Glass R. S. Molecular geometry of the norbornyl cation. I. Synthesis and acetolysis of the *exo*- and *endo*-4,5-*exo*-trimethylene-2-norbornyl *p*-toluenesulfonates. J. Am. Chem. Soc. 1967, 89, 2600–2610. 10.1021/ja00987a018.

[ref16] aBreitholle E. G.; Fallis A. G. Total synthesis of (±)-cedrol and (±)-cedrene *via* an intramolecular Diels-Alder reaction. J. Org. Chem. 1978, 43, 1964–1968. 10.1021/jo00404a025.

[ref17] aNarula A. S.; Trifilieff E.; Bang L.; Ourisson G. Oxidation of Cedrane and Cedrol with iodine tris-(trifluoroacetate). Tetrahedron Lett. 1977, 18, 3959–3960. 10.1016/S0040-4039(01)83402-X.

[ref18] aTochtermann W.; Sonnichsen F.; Wolff C.; Peters E.; Peters K.; von Schnering H. G. Synthese mittlerer und großer Ringe, XXV: Synthese funktionalisierter *trans*-Hydrindane mit angularer α-Ketoestergruppe. Chem. Ber. 1989, 122, 1969–1975. 10.1002/cber.19891221023.

[ref19] López-Suárez L.; Riesgo L.; Bravo F.; Ransom T. T.; Beutler J. A.; Echavarren A. M. Synthesis and Biological Evaluation of New (−)-Englerin Analogues. ChemMedChem. 2016, 11, 1003–1007. 10.1002/cmdc.201600040. 27005578 PMC4926265

[ref20] Zhang X.; Xie X.; Liu Y. Nickel-Catalyzed Highly Regioselective Hydrocyanation of Terminal Alkynes with Zn(CN)2 Using Water as the Hydrogen Source. J. Am. Chem. Soc. 2018, 140, 7385–7389. 10.1021/jacs.8b02542. 29851478

[ref21] Zeng X. P.; Zhou J. Me2(CH2Cl)SiCN: Bifunctional Cyanating Reagent for the Synthesis of Tertiary Alcohols with a Chloromethyl Ketone Moiety *via* Ketone Cyanosilylation. J. Am. Chem. Soc. 2016, 138, 8730–8733. 10.1021/jacs.6b05601. 27399262

[ref22] Burford C.; Cooke F.; Roy G.; Magnus P. Silicon in synthesis—17: Chloromethyl(trimethylsilyl)lithium—a new reagent for the direct conversion of aldehydes and ketones into α,β,-epoxytrimethylsilanes. Tetrahedron 1983, 39, 867–876. 10.1016/S0040-4020(01)88585-9.

[ref23] Wilde N. C.; Isomura M.; Mendoza A.; Baran P. S. Two-Phase Synthesis of (−)-Taxuyunnanine D. J. Am. Chem. Soc. 2014, 136, 4909–4912. 10.1021/ja501782r. 24625050 PMC3985808

[ref24] aGeorge D. T.; Kuenstner E. J.; Pronin S. V. A Concise Approach to Paxilline Indole Diterpenes. J. Am. Chem. Soc. 2015, 137, 15410–15413. 10.1021/jacs.5b11129. 26593869 PMC7556741

[ref25] aGansäuer A.; Pierobon M. Titanocene Catalyzed 5-*exo* Cyclizations of Epoxides. Synlett 2000, 2000, 1357–1359. 10.1055/s-2000-7133.

[ref26] Beckwith A. L. J.; Hay B. P. Kinetics of the reversible β-scission of the cyclopentyloxy radical. J. Am. Chem. Soc. 1989, 111, 230–234. 10.1021/ja00183a035.

[ref27] aZhao M.; Li J.; Mano E.; Song Z.; Tschaen D. M.; Grabowski E. J. J.; Reider P. J. Oxidation of Primary Alcohols to Carboxylic Acids with Sodium Chlorite Catalyzed by TEMPO and Bleach. J. Org. Chem. 1999, 64, 2564–2566. 10.1021/jo982143y.

[ref28] Chen M. S.; White M. C. A Predictably Selective Aliphatic C–H Oxidation Reaction for Complex Molecule Synthesis. Science 2007, 318, 783–787. 10.1126/science.1148597. 17975062

[ref29] Kong L.; Su F.; Yu H.; Jiang Z.; Lu Y.; Luo T. Total Synthesis of (−)-Oridonin: An Interrupted Nazarov Approach. J. Am. Chem. Soc. 2019, 141, 20048–20052. 10.1021/jacs.9b12034. 31801344

