# Supplementary material for: Asymmetric Total Synthesis of Illisimonin A
Source: J Am Chem Soc. 2023 Mar 16;145(12):7021–9. doi: 10.1021/jacs.3c01262 (PMC10064331; doi:10.1021/jacs.3c01262)
Supplement: Supplementary file 1 — ja3c01262_si_001.pdf [file ja3c01262_si_001.pdf]

Asymmetric Total Synthesis of Illisimonin A

Christoph Etling, Giada Tedesco, Anna Di Marco, Markus Kalesse*

Institute of Organic Chemistry, Leibniz Universität Hannover,
Schneiderberg 1b, 30167 Hannover, Germany

*Correspondence to: markus.kalesse@oci.uni-hannover.de

Supporting Information

Table of Contents

1 Substance overview 3
2 Abbreviations 4
3 General information 5
4 Experimental procedures
- 4.1 Asymmetric total synthesis of (–)-illisimonin A
  - Main sequence 6
  - Alternative endgame 24
  - NMR data of illisimonin A 26
  - CD spectrum of (–)-illisimonin A 27
- 4.2 Preparation of racemic Nazarov cyclization precursor 28
- 4.3 Reaction optimization 30
- 4.4 Studies on the synthesis of tricyclo[5.2.1.0^{1,5}]decanes from spirocyclic precursors *via* radical cyclizations 35

5 X-ray data 40
6 References 44
7 NMR spectra 45

1 Substance overview

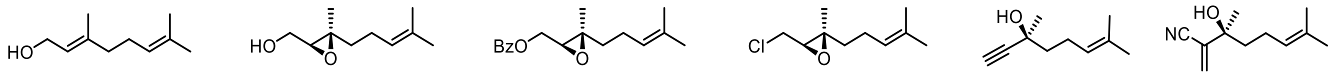

S1 **S2** **S3** **S4** **S5** **S6**

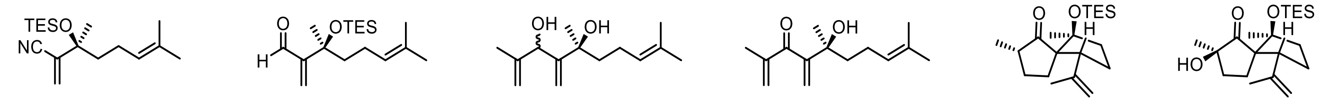

S7 **S8** **S9** **S10** **S11** **S12**

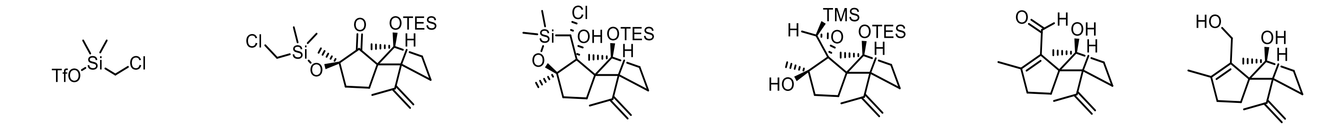

S13 **S14** **S15** **S16** **S17** **S18**

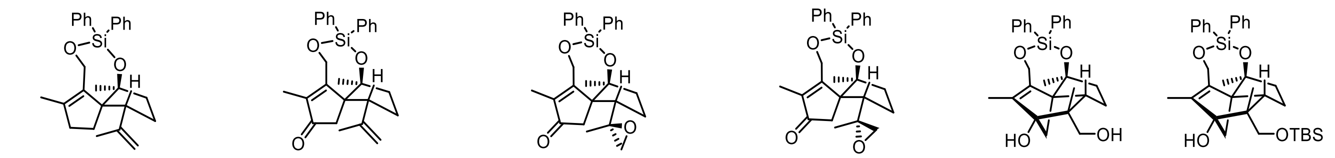

S19 **S20** **S21a** **S21b** **S22** **S23**

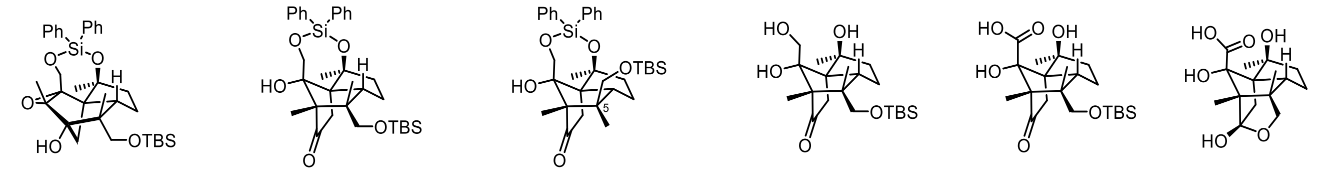

S24 **S25** 5-*epi*-**S25** **S26** **S27** **S28**

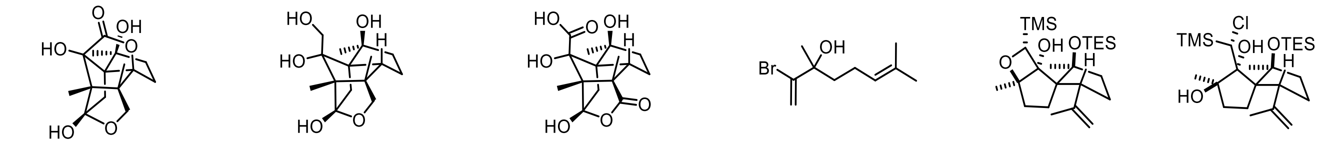

S29 **S30** **S31** (±)-**S32** (±)-**S33** (±)-**S34**

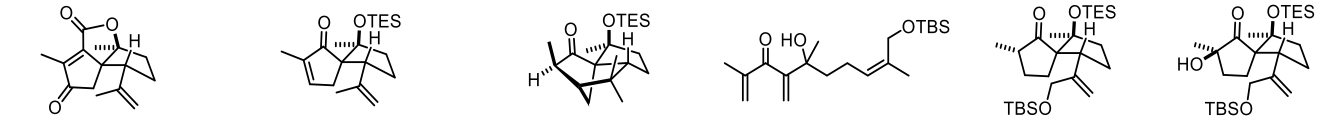

(±)-**S35** (±)-**S36** (±)-**S37** (±)-**S38** (±)-**S39** (±)-**S40**

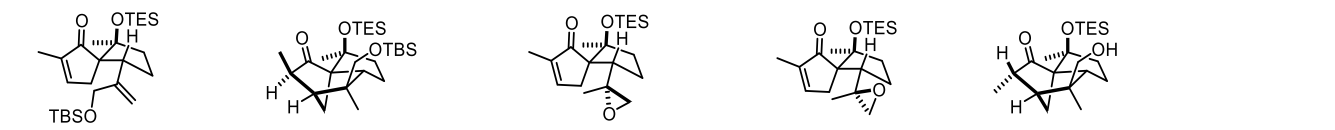

(±)-**S41** (±)-**S42** (±)-**S43a** (±)-**S43b** (±)-**S44**

2 Abbreviations

acac	acetylacetonate
Cap	caprolactamate
Cp	cyclopentadienyl
*m*CPBA	*meta*-chloroperbenzoic acid
CPME	cyclopentyl methyl ether
DCE	dichloroethane
DET	diethyltartrate
DIBAL-H	diisobutylaluminium hydride
D.I.T.	digital integration time
4-DMAP	4-dimethylaminopyridine
DMSO	dimethylsulfoxide
equiv	equivalents
esp	α,α,α',α'-tetramethyl-1,3-benzenedipropanoic acid
HFIP	hexafluoroisopropanol
IBX	iodoxybenzoic acid
KHMDS	potassium bis(trimethylsilyl)amide
LDA	lithium diisopropylamide
MS	molecular sieves
MTBE	methyl *tert*-butyl ether
n.d.	not determined
NHPI	*N*-hydroxyphtalimide
o2s	over two steps
o3s	over three steps
PDP	[[2-[1-(pyridin-2-ylmethyl)pyrrolidin-2-yl]pyrrolidin-1-yl]methyl]pyridine
PE	petroleum ether
r.t.	room Temperature
TBAF	tetra-*n*-butylammonium fluoride
TBS	*tert*-butyl-dimethylsilyl
TES	triethylsilyl
TFA	trifluoroacetic acid
TFT	trifluorotoluene
THF	tetrahydrofurane
TMS	trimethylsilyl

3 General information

Reactions employing air- or moisture-sensitive reagents and anhydrous solvents were performed under argon atmosphere. In these cases, the used glassware was dried by heating with a Bunsen burner under fine vacuum prior to use. Air- and moisture-sensitive liquids and solutions were transferred *via* syringe flushed with argon prior to use. All reagents were purchased from commercial suppliers and used without further purification unless otherwise noted. Tris(pentafluorophenyl)borane,[1] IBX,[2] anhydrous *t*BuOOH,[3] Fe(PDP)[4] and (±)-**S38**[5] were prepared according to literature procedures. Unless stated otherwise, temperatures, except room temperature (22 to 29 °C), refer to bath temperatures. Unless stated otherwise, all reactions were stirred magnetically.

Anhydrous solvents Dichloromethane, diisopropylamine and triethylamine were distilled under an inert atmosphere over calcium hydride. Tetrahydrofuran (stabilized with BHT and stored over molecular sieves), diethyl ether (stabilized with BHT and stored over molecular sieves), MTBE (stored over molecular sieves), CPME (stabilized with BHT and stored over molecular sieves) and *n*-pentane (stored over molecular sieves) were purchased from Acros Organics. Benzene was bought from Sigma Aldrich.

Organolithium reagents were purchased from Acros Organics.

Thin layer chromatography All reactions were monitored using pre-coated TLC sheets ALUGRAM® Xtra SIL G/UV_{254} (0.2 mm, silica gel, F_{254}, aluminium-backed, MACHEREY-NAGEL) with detection by UV light (λ = 254 nm) and/or by staining with either acidic vanillin stain, acidic anisaldehyde, basic potassium permanganate or Hanessian's stain.

Flash column chromatography was performed using silica gel (0.04-0.063 mm, 240-400 mesh) obtained from MACHEREY-NAGEL. The applied petroleum ether fraction had a bp of 40−60 °C. The eluent is given in volume ratios (v/v).

NMR experiments were recorded in $CDCl_3$, C_6D_6 or methanol-d_4 purchased from deutero GmbH. The following NMR spectrometers were used: Bruker Ultrashield 400 MHz (ULS400), Bruker Ascend 400 MHz (ASC400), Bruker Ascend 400 MHz with Prodigy BBFO probe head, Bruker Ultrashield 500 MHz with TCI cyro probe head, Bruker Ascend 600 MHz with DUL cryo probe head. The ^{1}H-NMR spectra were calibrated using the residual solvent peak: $\delta(CDCl_3)$ = 7.26 ppm, $\delta(C_6D_6)$ = 7.16 ppm, $\delta(CD_3OD)$ = 3.31 ppm. ^{13}C-NMR spectra were calibrated using the solvent's carbon signal: $\delta(CDCl_3)$ = 77.16 ppm, $\delta(C_6D_6)$ = 128.06 ppm, $\delta(CD_3OD)$ = 49.00 ppm. The methanol-d_4 contained trace amounts of ethanol-d_6, as confirmed by deutero GmbH. Chemical shifts δ are given in parts per million (ppm), coupling constants *J* in Hertz (Hz) and multiplicities as follows: s, singlet; d, doublet; t, triplet; q, quartet; quint, quintet; m, multiplet; or combinations of these acronyms. Broad signals will be denoted by addition of the letter "b"; e.g. "broad singlet" is written as "bs". Peak integrals are given as multiples of protons *Y*H, with *Y* being the number of protons belonging to the given signal.

For all substances NOE experiments were performed with, a signal-structure assignments is given. For the assignment of NMR signals to atoms in the structures, NMR active positions are labeled with arabic numbers. Those numbers were assigned arbitrarily and do not necessarily follow the numbering by the IUPAC system. The atom labels are written in the form "H-x" for proton spectra, where "x" marks the position number. The notation "C-x" is used for carbon spectra. In case of multiplets that consist of several overlapping proton signals, the "grouped" notation "H-{x, y, ...}" is used, listing the associated positions in brackets.

Signals that belong to heteroatom bound groups that are not part of the actual backbone of a molecule (such as protecting groups), are referred to in the notation "X*R*", with "X" being the corresponding heteroatom and "*R*" being the abbreviation for the particular residue/group.

If the samples contained a mixture of two inseparable isomers NMR data is reported in the following way: ^{1}H-NMR data: For signals that can be assigned to only one of the two diastereomers, the descriptor "major" or "minor" is added, e.g. "1.23 (s, 3H, major)". If a signal can be assigned to the same group of both diastereomers, the descriptor "major+minor" is added. E.g. "4.32-4.23 (m, 1H, major + minor)" corresponds to 1H for the major and 1H for the minor diastereomer. If multiplets arise from overlapping of several, different signals of both diastereomers, the number of protons belonging to each diastereomer in the multiplet is specified, e.g. "1.88–1.70 (m, 2H(major) + 3H(minor))". ^{13}C-NMR data: Signals corresponding to the minor diastereomer are marked with an asterisk *. If carbon signals overlap, the descriptor "both diastereomers" is added in parentheses.

NMR spectra were processed using TopSpin (Bruker) Version 4.1.4 or MestreNova version 6.22.0-7238.

High Resolution Mass Spectra (HRMS) were obtained either using a Waters Q-Tof Premier (ESI), a Waters LCT Premier (ESI) or Micromass GCT (CI).

GC/MS (Electron-impact ionization) data was recorded with a GC-system Agilent 6890 hyphenated with an Agilent 5977B mass-sensitive detector. For EI mass spectra, the fragmentation patterns with relative intensities are given.

Melting points were measured with a MPA100 melting point apparatus by Stanford Research Systems. The solvent from which the compounds were crystallized is specified for each compound.

Optical rotations were measured with A. Krüss optronic P3000 polarimeter or a Perkin-Elmer 241 polarimeter. Measurements were performed in a cuvette with a cell length of d = 1 dm and at wavelength of λ_{max} = 589.3 nm (sodium D-line). The solvent used is specified for each substance. Concentrations are given in g/100 mL.

CD spectra were recorded with a Jasco J-815 CD Spectrometer. Range of measurement: 190–400 nm, resolution: 0.2 nm, speed: 50 nm/min, D.I.T. 0.5 s. A Hellma Analytics Suprasil 110-QS quartz cuvette with a thickness of 1 mm and a volume of 350 µL was used.

4 Experimental Procedures

4.1 Asymmetric total synthesis of (–)-illisimonin A – main sequence

(2*S*,3*S*)-2,3-Epoxy geraniol (S2)

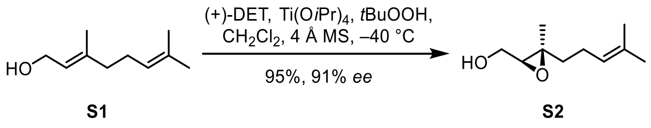

S2 was synthesized according to the procedure published by Echavarren and co-workers.[6]

A 2 L three-necked flask, equipped with an overhead stirrer with inert gas inlet, dropping funnel and thermometer, was charged under argon with 4 Å MS (25.0 g) and anhydrous CH_2Cl_2 (500 mL). The stirred mixture was cooled to −20 °C (internal temperature), before (+)-DET (7.8 mL, 45.4 mmol, 14 mol%) and Ti(O*i*Pr)$_4$ (9.0 mL, 30.5 mmol, 9.4 mol%) were added. *t*BuOOH (4.64 M in CH_2Cl_2, 237 mL, 1.10 mol, 3.4 equiv) was added to the reaction mixture *via* dropping funnel over 30 min. The internal temperature was maintained below –20 °C. The dropping funnel was rinsed with anhydrous CH_2Cl_2 (20.0 mL) and stirring at −20 °C was continued for 30 min. The mixture was cooled to −40 °C and a solution of geraniol (**S1**) (56.2 mL, 324 mmol, 1.0 equiv) in anhydrous CH_2Cl_2 (50.0 mL) was added over 1 h 50 min *via* dropping funnel. After complete addition, the dropping funnel was rinsed with anhydrous CH_2Cl_2 (20.0 mL) and stirring at −40 °C was continued for 40 min. Water (200 mL) was added at −40 °C under vigorous stirring and the mixture was allowed to warm to r.t.. 100 mL of an aqueous solution containing 30% NaOH and 5% NaCl were added and the mixture was stirred vigorously at r.t. for 45 min; phase separation was obtained after addition of a small volume of MeOH (approx. 5.0 mL) to the mixture. The phases were separated and the white aqueous phase was extracted with CH_2Cl_2 (3x 200 mL). The combined organic phases were dried over Na_2SO_4, filtered over Celite® and concentrated under reduced pressure. The crude product was dried with a rotary evaporator to remove the majority of excess *t*BuOOH.[1] Vacuum distillation gave epoxy geraniol (**S2**) (52.3 g, 307 mmol, 95%, 91% *ee*) as a colorless oil.

b.p.(1.3 mbar) = 97 °C.
R*f* (PE:EtOAc = 4:1; vanillin) = 0.20 (dark blue).
^{1}H-NMR (400 MHz, $CDCl_3$): *δ*(ppm) = 5.11–5.04 (m, 1H), 3.86–3.76 (m, 1H), 3.73–3.63 (m, 1H), 2.97 (dd, *J* = 6.7, 4.3 Hz, 1H), 2.13–2.03 (m, 2H), 1.94–1.86 (m, 1H), 1.72–1.63 (m, 4H), 1.60 (s, 3H), 1.51–1.42 (m, 1H), 1.29 (s, 3H).
^{13}C-NMR (101 MHz, $CDCl_3$): *δ*(ppm) = 132.2, 123.4, 63.2, 61.5, 61.3, 38.6, 25.7, 23.8, 17.7, 16.8.
HRMS(ESI): Calcd for $C_{10}H_{18}O_2Na$ $[M+Na]^+$: 193.1204; found: 193.1207.
$[\alpha]_D^{22}$ = −4.8 (*c* = 1.7, $CHCl_3$).
The obtained analytical data match the data reported by Mohapatra and co-workers.[7]

The enantiomeric excess was determined by chiral HPLC after formation of the corresponding benzoate:

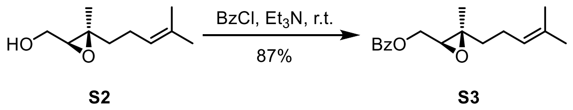

Et_3N (48.8 µL, 350 µmol, 1.8 equiv) and benzoyl chloride (27.7 µL, 240 µmol, 1.2 equiv) were added sequentially at r.t. to a stirred solution of epoxy geraniol (**S2**) (34.0 mg, 200 µmol, 1.0 equiv) in anhydrous CH_2Cl_2 (1.0 mL). Stirring at r.t. was continued for 5.5 h, the mixture was diluted with MTBE and washed with aqueous NaOH (1.0 M, 3x 1.0 mL), aqueous $NaHCO_3$ (sat., 3x 1.0 mL) and brine (1x 1.0 mL). The organic phase was dried over Na_2SO_4 and concentrated under reduced pressure. Purification by column chromatography (PE:EtOAc = 25:1) afforded benzoate **S3** (47.5 mg, 173 µmol, 87%) as a colorless oil.

R*f* (PE:EtOAc = 10:1; vanillin) = 0.57 (dark blue).
^{1}H-NMR (400 MHz, $CDCl_3$): *δ*(ppm) = 8.08 (d, *J* = 7.3 Hz, 2H), 7.57 (t, *J* = 7.3 Hz, 1H), 7.45 (t, *J* = 7.7 Hz, 2H), 5.13–5.05 (m, 1H), 4.57 (dd, *J* = 12.1, 4.3 Hz 1H), 4.29 (dd, *J* = 12.1, 6.7 Hz, 1H), 3.14 (dd, *J* = 6.8, 4.2 Hz, 1H), 2.21–2.02 (m, 2H), 1.77–1.68 (m, 1H), 1.66 (s, 3H), 1.61 (s, 3H), 1.56–1.46 (m, 1H), 1.38 (s, 3H).
^{13}C-NMR (101 MHz, $CDCl_3$): *δ*(ppm) = 166.6, 133.3, 132.4, 129.93, 129.88, 128.5, 123.3, 64.1, 60.8, 59.9, 38.5, 25.8, 23.8, 17.8, 17.1.
HRMS(ESI): Calcd for $C_{17}H_{22}O_3Na$ $[M+Na]^+$: 297.1467; found: 297.1457.
$[\alpha]_D^{22}$ = –13.0 (*c* = 2.2, $CHCl_3$).

[1] Extended evaporation times were necessary to remove the majority of *t*BuOOH.

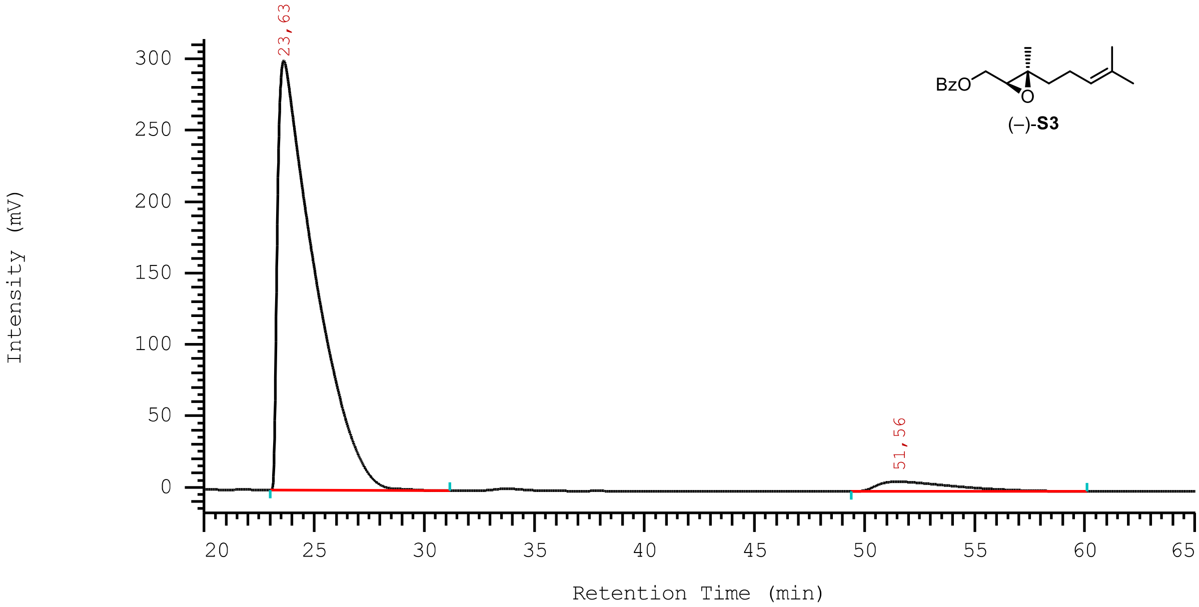

No.	RT	Area	Conc 1	BC
1	23,63	35011225	95,487	BB
2	51,56	1654714	4,513	MC
		36665939	100,000	

Figure S1. Chromatogram of enantioenriched epoxy geraniol benzoate (**S3**).

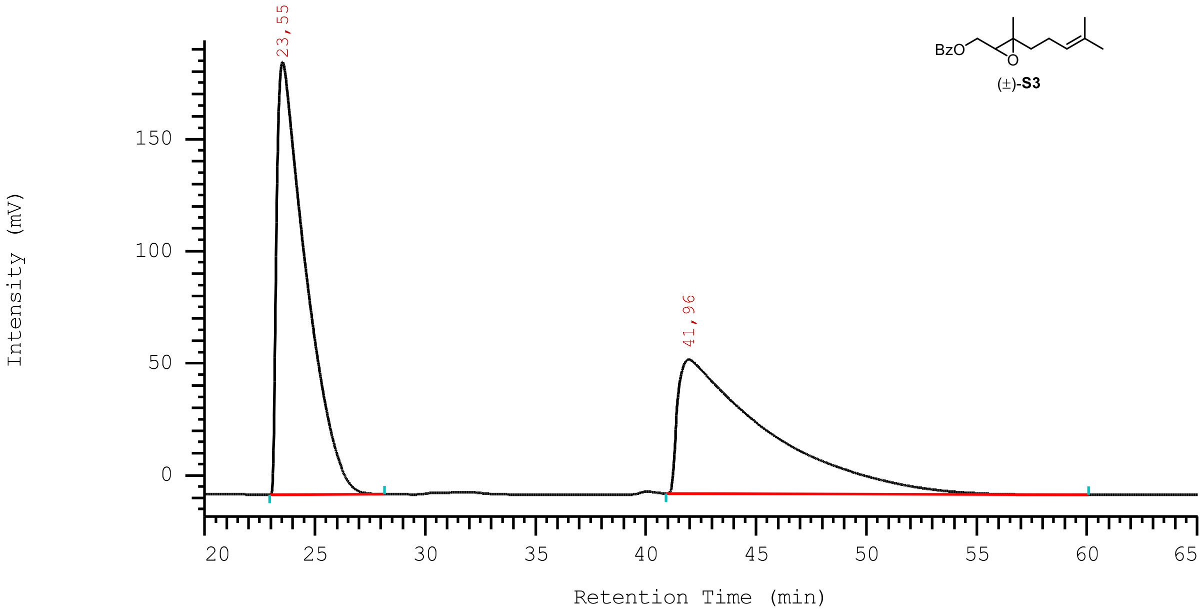

No.	RT	Area	Conc 1	BC
1	23,55	17641064	51,386	MC
2	41,96	16689500	48,614	MC
		34330564	100,000	

Figure S2. Chromatogram of racemic epoxy geraniol benzoate ((±)-**S3**).

HPLC setup:

Merck/Hitachi La Chrome®-HPLC-system with L-7150 pump, L-7200 autosampler and L-7400-UV-detector
Column: Daicel Chiracel® OD-H (250 mm x 4.6 mm)
Eluent: *n*-Hexane:*iso*-propanol = 200:1
Flow rate: 0.5 mL/min

(2*S*,3*R*)-3-(Chloromethyl)-2-methyl-2-(4-methylpent-3-en-1-yl)oxirane (S4)

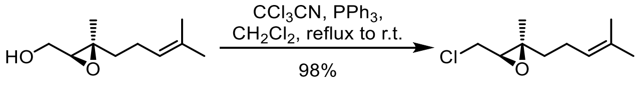

S2 **S4**

S4 was synthesized after a modification of the procedure published by Chavasiri and co-workers.[8]
A 1 L two-necked flask equipped with a reflux condenser and a rubber septum was charged under argon with PPh_3 (95.2 g, 363 mmol, 1.2 equiv), epoxy geraniol **S2** (51.5 g, 302 mmol, 1.0 equiv) and anhydrous CH_2Cl_2 (300 mL). Trichloroacetonitrile (36.4 mL, 363 mmol, 1.2 equiv) was added carefully at r.t.. The addition rate was adjusted so that the reaction mixture maintained a gentle reflux. The amber reaction mixture was stirred at r.t. for additional 40 min, water (200 mL) was added and the mixture stirred vigorously for 15 min. The phases were separated and the aqueous phase was extracted with CH_2Cl_2 (3x 200 mL). The combined organic phases were dried over Na_2SO_4 and concentrated under reduced pressure. The crude product was applied on silica and purified by column chromatography (PE:EtOAc = 25:1) to afford epoxy chloride **S4** (55.7 g, 295 mmol, 98%) as a light yellow oil.

R*f* (PE:EtOAc = 10:1; vanillin) = 0.60 (grey-blue).
^{1}H-NMR (400 MHz, $CDCl_3$): *δ*(ppm) = 5.13–5.05 (m, 1H), 3.69 (dd, *J* = 11.4, 5.9 Hz, 1H), 3.44 (dd, *J* = 11.4, 7.2 Hz, 1H), 3.03 (dd, *J* = 7.1, 5.9 Hz, 1H), 2.17–2.02 (m, 2H), 1.76–1.65 (m, 4H), 1.61 (s, 3H), 1.51–1.41 (m, 1H), 1.32 (s, 3H).
^{13}C-NMR (101 MHz, $CDCl_3$): *δ*(ppm) = 132.4, 123.3, 62.3, 61.7, 42.4, 38.4, 25.8, 23.9, 17.8, 16.4.
GCMS(EI): *m/z* (%): 109 (79), 82 (17), 81 (20), 69 (96), 67 (52), 55 (26), 53 (17), 43 (64), 41 (100), 39 (32).
HRMS(CI): Calcd for $C_{10}H_{18}OCl$ $[M+H]^+$: 189.1046; found: 189.1039.
$[\alpha]_D^{22}$ = +12.2 (*c* = 5.3, $CHCl_3$).
The obtained analytical data match the data reported by Mohapatra and co-workers.[7]

(*S*)-3,7-Dimethyloct-6-en-1-yn-3-ol (S5)

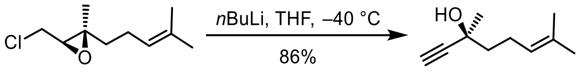

S4 **S5**

S5 was synthesized according to the procedure published by Echavarren and co-workers.[6]
*n*BuLi (2.5 M in hexanes, 413 mL, 1.03 mol, 3.5 equiv) was added over 1.5 h to a stirred solution of epoxy chloride **S4** (55.7 g, 295 mmol, 1.0 equiv) in anhydrous THF (420 mL) at −40 °C. The solution turned deep brown upon addition of the organolithium reagent. After complete addition, stirring at −40 °C was continued for 2 h, before aqueous NH_4Cl (sat., 100 mL) was added and the mixture was allowed to warm to r.t. under vigorous stirring. The phases were separated and the aqueous phase was extracted with Et_2O (3x 100 mL). The combined organic phases were washed with brine (200 mL), dried over Na_2SO_4 and concentrated under reduced pressure to afford a dark-brown oil. After purification by vacuum distillation propargylic alcohol **S5** (38.5 g, 253 mmol, 86%) was obtained as a colorless oil.

b.p. (40 mbar) = 103 °C.
R*f* (PE:EtOAc = 10:1; vanillin) = 0.36 (blue).
^{1}H-NMR (400 MHz, $CDCl_3$): *δ*(ppm) = 5.21–5.12 (m, 1H), 2.46 (s, 1H), 2.36–2.24 (m, 1H), 2.24–2.12 (m, 1H), 1.97 (bs, 1H), 1.74–1.67 (m, 2H), 1.70 (s, 3H), 1.66 (s, 3H), 1.50 (s, 3H).
^{13}C-NMR (101 MHz, $CDCl_3$): *δ*(ppm) = 132.7, 123.8, 87.7, 71.6, 68.4, 43.3, 29.9, 25.8, 23.7, 17.8.
GCMS(EI): *m/z* (%): 137 (28), 119 (70), 109 (22), 91 (51), 79 (26), 69 (100), 68 (19), 67 (41), 55 (69), 53 (29), 43 (91), 41 (100), 39 (46).
HRMS(CI): Calcd for $C_{10}H_{17}O$ $[M+H]^+$: 153.1279; found: 153.1284.
$[\alpha]_D^{22}$ = −12.9 (*c* = 2.9, $CHCl_3$).
The obtained analytical data match the data reported by Mohapatra and co-workers.[7]

β-Hydroxy acrylonitrile S6

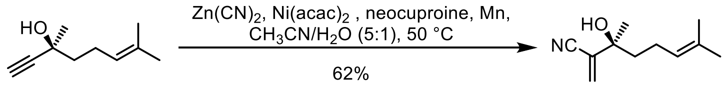

S5 **S6**

S6 was synthesized after a variation of the protocol published by Liu and co-workers.[9]

$Ni(acac)_2$ (6.41 g, 25.0 mmol, 0.1 equiv), neocuproine (6.24 g, 30.0 mmol, 0.12 equiv), $Zn(CN)_2$ (23.5 g, 200 mmol, 0.8 equiv) and Mn (6.86 g, 125 mmol, 0.5 equiv) were placed in a Schlenk flask with a rubber septum and the flask was evacuated and backfilled with argon three times. Degassed[2] MeCN (1.0 L) was added and the green-grey suspension was stirred at r.t. for 20 min. Propargylic alcohol **S5** (38.0 g, 250 mmol, 1.0 equiv) was added, followed by degassed[2] water (210 mL). The septum was replaced by a glass stopper, the reaction mixture was heated to 50 °C and stirred for 16 h. The mixture was allowed to cool to r.t., water and MTBE (500 mL each) were added and the mixture was filtered over Celite®. The phases were separated and the aqueous phase was extracted with MTBE (3x 250 mL). The combined organic phases were washed with water and brine, dried over Na_2SO_4 and concentrated under reduced pressure. The crude product was purified by column chromatography (PE:EtOAc = 25:1 to 20:1 to 15:1 to 10:1), followed by bulb-to-bulb distillation (0.4 mbar, 120–130 °C) to give β-hydroxy acrylonitrile **S6** (27.7 g, 155 mmol, 62%) as a light yellow oil.

R*f* (PE:EtOAc = 5:1; vanillin) = 0.37 (purple).
^{1}H-NMR (400 MHz, $CDCl_3$): *δ*(ppm) = 6.09 (d, *J* = 0.6 Hz, 1H), 6.00 (s, 1H), 5.18–5.11 (m, 1H), 2.15–1.96 (m, 2H), 1.89–1.80 (m, 2H) 1.74 (ddd, *J* = 14.2, 9.0, 7.3 Hz, 1H), 1.70–1.68 (m, 3H), 1.61 (s, 3H), 1.44 (s, 3H).
^{13}C-NMR (101 MHz, $CDCl_3$): *δ*(ppm) = 133.5, 130.6, 128.9, 123.4, 117.8, 74.6, 40.4, 27.9, 25.8, 22.6, 17.9.
HRMS(ESI): Calcd for $C_{11}H_{17}NONa$ $[M+Na]^+$: 202.1208; found: 202.1201.
$[\alpha]_D^{24}$ = +0.9 (*c* = 30.0, $CHCl_3$).

TES-protected β-hydroxy acrylonitrile S7

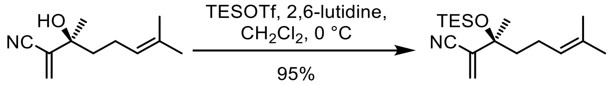

S6 **S7**

β-Hydroxy acrylonitrile **S6** (26.7 g, 149 mmol, 1.0 equiv) and 2,6-lutidine (34.5 mL, 298 mmol, 2.0 equiv) were dissolved under argon in anhydrous CH_2Cl_2 (500 mL). The solution was cooled to 0 °C and TESOTf (40.7 mL, 179 mmol, 1.2 equiv) was added over 1 h *via* syringe pump. After complete addition, stirring at 0 °C was continued for 25 min, before water (250 mL) was added and the mixture allowed to warm to r.t. under vigorous stirring. The phases were separated and the aqueous phase extracted with CH_2Cl_2 (3x, 200 mL). The combined organic phases were washed with water (2x 250 mL) and brine (250 mL), dried over Na_2SO_4 and concentrated under reduced pressure. Purification *via* column chromatography (PE:EtOAc = 100:1 to 50:1) gave TES-protected β-hydroxy acrylonitrile **S7** (41.3 g, 141 mmol, 95%) as a yellow oil.

R*f* (PE:EtOAc = 25:1; vanillin) = 0.60 (dark-blue).
^{1}H-NMR (400MHz, $CDCl_3$): *δ*(ppm) = 6.04–6.02 (m, 1H), 5.95–5.94 (m, 1H), 5.11–5.04 (m, 1H), 2.07–1.95 (m, 1H), 1.93–1.81 (m, 1H), 1.80–1.71 (m, 1H), 1.67 (s, 3H), 1.64–1.55 (m, 1H), 1.59 (s, 3H), 1.48 (s, 3H), 0.97 (t, *J* = 7.9 Hz, 9H), 0.64 (q, *J* = 7.9 Hz, 6H).
^{13}C-NMR (101MHz, $CDCl_3$): *δ*(ppm) = 132.2, 130.9, 128.9, 123.6, 118.1, 76.2, 42.1, 28.0, 25.8, 22.5, 17.7, 7.2, 6.8.
HRMS(ESI): Calcd for $C_{17}H_{31}NOSiNa$ $[M+Na]^+$: 316.2073; found: 316.2082.
$[\alpha]_D^{24}$ =+0.3 (*c* = 24.2, $CHCl_3$).

[2] Degassed by sonication under an argon atmosphere for 30 min.

Aldehyde S8

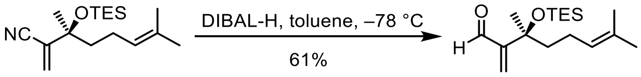

S7 S8

DIBAL-H (1.0 M in toluene, 81.0 mL, 81.0 mmol, 1.2 equiv) was added over 30 min to a stirred solution of TES-protected β-hydroxy acrylonitrile **S7** (19.8 g, 67.5 mmol, 1.0 equiv) in anhydrous toluene (675 mL) at –78 °C. Stirring at –78 °C was continued for 3 h, before the reaction was then quenched at –78 °C by slow addition of MeOH (16.0 mL). Aqueous potassium sodium tartrate (sat., 1.4 L) was added at –78 °C, the mixture was diluted with MTBE (0.5 L) and allowed to warm to r.t. under vigorous stirring. After 1 h, the phases were separated and the aqueous phase was extracted with MTBE (3x 300 mL). The combined organic phases were washed with brine (400 mL), dried over Na_2SO_4 and concentrated under reduced pressure. Purification by column chromatography (PE:EtOAc = 200:1 to 6:1) gave aldehyde **S8** (12.3 g, 41.5 mmol, 61%) as a light yellow oil.

R*f* (PE:EtOAc = 25:1; vanillin) = 0.61 (purple).
^{1}H-NMR (400 MHz, C_6D_6): *δ*(ppm) = 9.33 (s, 1H), 6.44 (d, *J* = 1.9 Hz, 1H), 5.49 (d, *J* = 1.9 Hz, 1H), 5.22–5.15 (m, 1H), 2.31 (ddd, *J* = 13.4, 11.7, 5.1 Hz, 1H), 2.21–2.09 (m, 1H), 1.85–1.73 (m, 1H), 1.67–1.58 (m, 4H), 1.55 (s, 3H), 1.48 (s, 3H), 0.97 (t, *J* = 7.9 Hz, 9H), 0.58 (q, *J* = 7.9 Hz, 6H).
^{13}C-NMR (101 MHz, C_6D_6): *δ*(ppm) = 192.6, 155.7, 135.3, 131.2, 124.9, 76.9, 41.3, 28.5, 25.8, 23.4, 17.7, 7.4, 7.3.
HRMS(ESI): Calcd for $C_{17}H_{32}O_2SiNa$ $[M+Na]^+$: 319.2069; found: 319.2078.
$[\alpha]_D^{22}$ = +17.9 (*c* = 5.2, $CHCl_3$).

Diol S9

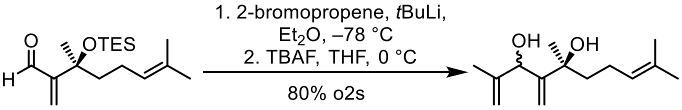

S8 S9

A 1 L Schlenk flask was charged under argon with Et_2O (95 mL) and 2-bromopropene (5.8 mL, 61.4 mmol, 1.5 equiv). The solution was cooled to −78 °C and *t*BuLi (1.9 M in pentane, 63.0 mL, 120 mmol, 2.9 equiv) was added dropwise over 50 min. After 4 h, a −78 °C cold solution of aldehyde **S8** (12.1 g, 40.8 mmol, 1.0 equiv) in anhydrous Et_2O (200 mL) was added from a second Schlenk flask *via* transfer cannula over 40 min. The resulting orange solution was stirred at −78 °C for additional 50 min. Aqueous NH_4Cl (sat., 100 mL) was added and the mixture allowed to warm to r.t. under vigorous stirring. The phases were separated and the aqueous phase was extracted with Et_2O (3x 100 mL). The combined organic phases were washed with brine (100 mL), dried over Na_2SO_4 and concentrated under reduced pressure. The so obtained mono-TES-protected diol was used in the subsequent deprotection without further purification.
The mono-TES-protected diol was dissolved in THF (400 mL), cooled to 0 °C and TBAF (1.0 M in THF, 49.1 mL, 49.1 mmol, 1.2 equiv) was added dropwise over 25 min. Stirring at 0 °C was continued for 1 h, before aqueous NH_4Cl (sat., 100 mL) was added and the mixture was allowed to warm to r.t.. The phases were separated and the aqueous phase was extracted with EtOAc (3x 100 mL). The combined organic phases were washed with brine, dried over Na_2SO_4 and all volatiles were removed under reduced pressure. Purification by column chromatography (PE:EtOAc = 4:1) gave diol **S9** (7.32 g, 32.6 mmol, 80% o2s, d.r. 4:1) as a pale yellow oil that solidified in the freezer.

R*f* (PE:EtOAc = 5:1; vanillin) = 0.21 (dark blue).
^{1}H-NMR (400 MHz, $CDCl_3$): *δ*(ppm) = 5.19–5.16 (m, 1H), 5.15–5.14 (m, 1H), 5.14–5.08 (m, 2H), 5.04–5.00 (m, 1H), 4.75 (bs, 1H, major), 4.74 (bs, 1H, minor), 2.38 (bs, 2H), 2.08–1.97 (m, 2H), 1.78–1.63 (m, 2H), 1.72–1.70 (m, 3H), 1.69–1.67 (m, 3H), 1.62–1.59 (m, 3H), 1.40 (s, 3H, minor), 1.39 (s, 3H, major).
^{13}C-NMR (101 MHz, $CDCl_3$): *δ*(ppm) = 153.8, 153.3*, 146.5, 146.1*, 132.2 (both diastereomers), 124.3, 124.2*, 112.7*, 112.1, 111.9*, 111.7, 76.6*, 76.4, 75.63*, 75.56, 42.3*, 42.2, 29.3, 29.0*, 25.8 (both diastereomers), 23.1*, 22.8, 19.8*, 19.6, 17.9, 17.8*.
HRMS(ESI): Calcd for $C_{14}H_{24}O_2Na$ $[M+Na]^+$: 247.1674; found: 247.1674.
$[\alpha]_D^{22}$ = +8.0 (*c* = 1.0, $CHCl_3$).

Cross-conjugated ketone S10

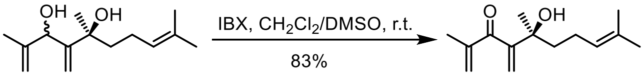

S9 **S10**

IBX (10.8 g, 38.6 mmol, 1.2 equiv) was added in one portion to a stirred solution of diol **S9** (7.21 g, 32.1 mmol, 1.0 equiv) in CH_2Cl_2/DMSO (1:1, 130 mL) and the mixture was stirred at r.t. for 1 h 45 min. Water (130 mL) was added under vigorous stirring and the mixture was diluted with CH_2Cl_2 (100 mL). The phases were separated and the aqueous phase was extracted with CH_2Cl_2 (5x 100 mL). The combined organic phases were washed with water (2x 300 mL) and brine (300 mL), dried over Na_2SO_4 and concentrated under reduced pressure. Purification *via* column chromatography (PE:EtOAc = 25:1 to 10:1) gave cross-conjugated ketone **S10** (5.95 g, 26.8 mmol, 83%) as a light yellow oil.

b.p. (0.5 mbar) = 91 °C.
R_f (PE:EtOAc = 9:1; vanillin) = 0.44 (turquoise).
^{1}H-NMR (400 MHz, $CDCl_3$): δ(ppm) = 5.85–5.82 (m, 2H,), 5.78 (bs, 1H), 5.59 (s, 1H), 5.11–5.04 (m, 1H), 3.49 (s, 1H,), 2.03–1.95 (m, 2H), 1.93 (s, 3H), 1.82–1.73 (m, 1H), 1.69–1.61 (m, 4H),1.56 (s, 3H), 1.38 (s, 3H).
^{13}C-NMR (101 MHz, $CDCl_3$): δ(ppm) = 201.6, 151.7, 144.7, 132.0, 127.9, 124.2, 122.7, 74.8, 41.5, 27.1, 25.8, 23.1, 18.0, 17.8.
HRMS(ESI): Calcd for $C_{14}H_{22}O_2Na$ $[M+Na]^+$: 245.1517; found: 245.1517.
$[\alpha]_D^{22}$ = +6.0 (*c* = 1.3, $CHCl_3$).

α-Hydroxy ketone S12

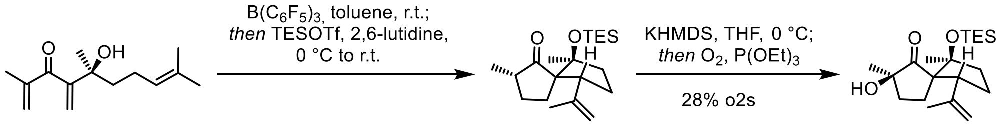

S10 **S11** **S12**

$B(C_6F_5)_3$ (341 mg, 666 µmol, 2.5 mol%) was added in one portion to a stirred solution of cross-conjugated ketone **S10** (5.92 g, 26.6 mmol, 1.0 equiv) in toluene (270 mL) at r.t. and stirring at this temperature was continued for 24 h. During this time the solution color changed from nearly colorless over bright pink to orange. The reaction mixture was cooled to 0 °C and 2,6-lutidine (12.3 mL, 107 mmol, 4.0 equiv) and TESOTf (12.1 mL, 53.2 mmol, 2.0 equiv) were added successively. The cooling bath was removed and stirring was continued at r.t. for 2 h 45 min. Water (200 mL) was added, the phases were separated and the aqueous phase was extracted with EtOAc (3x 150 mL). The combined organic phases were washed with brine (200 mL), dried over Na_2SO_4 and concentrated under reduced pressure. The crude product purified by column chromatography (PE:EtOAc = 20:1) to separate protected spiro ketone **S11** from the main impurities. Impure **S11** (4.32 g) was obtained as a yellow oil and was used in the following α-oxidation without further purification.

Analytical data of spiro ketone **S11**[3]:
R_f (PE:EtOAc = 25:1; vanillin) = 0.64 (purple).
^{1}H-NMR (400 MHz, $CDCl_3$): δ(ppm) = 4.78 (s, 1H), 4.63 (s, 1H), 3.21 (dd, *J* = 11.4, 7.9 Hz, 1H), 2.13–1.95 (m, 3H), 1.94–1.82 (m, 1H), 1.81–1.72 (m, 1H), 1.69–1.60 (m, 2H), 1.59–1.48 (m, 5H), 1.29 (s, 3H), 1.11 (d, *J* = 6.8 Hz, 3H), 0.92 (t, *J* = 7.9 Hz, 9H), 0.54 (q, *J* = 7.9Hz, 6H).
^{13}C-NMR (101 MHz, $CDCl_3$): δ(ppm) = 222.8, 146.1, 113.3, 86.2, 63.8, 51.6, 44.2, 40.4, 28.9, 28.1, 27.8, 26.2, 22.4, 14.1, 7.1, 6.6.
HRMS(ESI): Calcd for $C_{20}H_{36}O_2SiNa$ $[M+Na]^+$: 359.2382; found: 359.2386.
$[\alpha]_D^{26}$ = +68.2 (*c* = 1.1, $CHCl_3$).

A solution of purified **S11** (4.31 g, 12.8 mmol, 1.0 equiv) in anhydrous THF (6.5 mL) was placed in a 100 mL Schlenk tube under argon, the solution was cooled to 0 °C and KHMDS (1.0 M in THF, 26.0 mL, 26.0 mmol, 2.0 equiv)[4] was added under vigorous stirring over 20 min. The yellow solution was stirred at 0 °C for 1 h, before $P(OEt)_3$ (4.4 mL, 25.6 mmol, 2.0 equiv) was added and pressured air was bubbled through the solution (*via* a gas inlet tube with frit). Upon exposure to oxygen, the solution gradually became darker,

[3] An analytically pure sample of **S11** was obtained by successive Nazarov cyclization and TES-protection with isolation of the unprotected spiro ketone by column chromatography after the protocol described earlier.[5] The TES-protection was conducted as follows: A solution of the unprotected spiro ketone (1.78 g, 8.01 mmol, 1.0 equiv) in anhydrous CH_2Cl_2 (5.0 mL) was added dropwise to a stirred solution of 2,6-lutidine (2.8 mL, 24.0 mmol, 3.0 equiv) and TESOTf (2.7 mL, 12.0 mmol, 1.5 equiv) in anhydrous CH_2Cl_2 (75 mL) at 0 °C. After complete addition, the solution was allowed to warm to r.t. and stirring was continued for 2.5 h. Water (50 mL) was added, the phases were separated and the aqueous phase was extracted with CH_2Cl_2 (3x). The combined organic phases were washed with brine, dried over Na_2SO_4 and concentrated under reduced pressure. Column chromatography (PE:EtOAc = 200:1 to 100:1) gave TES protected spiro ketone **S11** (2.39 g, 7.10 mmol, 89%) as a light yellow oil.

[4] KHMDS (1.0 M in THF) was freshly prepared prior to use: 5.19 g of KHMDS were dissolved in 26.0 mL of anhydrous THF.

with a dark orange solution indicating completion of the reaction. After exposure to the air stream for 15 min, aqueous NH_4Cl (sat., 20 mL) was added and the mixture was diluted with MTBE (25 mL). The phases were separated, the aqueous phase extracted with MTBE (5x 25 mL) and the combined organic phases were washed with brine (50 mL), dried over Na_2SO_4 and concentrated under reduced pressure. Purification *via* column chromatography (PE:EtOAc = 15:1) gave α-hydroxy ketone **S12** (2.60 g, 7.37 mmol, 28% over two steps) as a light yellow oil that solidified in the freezer.

R_f (PE:EtOAc = 4:1; vanillin) = 0.66 (purple).
^{1}H-NMR (400 MHz, $CDCl_3$): *δ*(ppm) = 4.90–4.86 (m, 1H), 4.72–4.69 (m, 1H), 3.14 (dd, *J* = 10.8, 8.2 Hz, 1H), 2.22–2.12 (m, 1H), 1.95–1.73 (m, 5H), 1.72–1.53 (m, 2H), 1.66 (s, 3H), 1.28 (s, 6H), 0.91 (t, *J* = 7.9 Hz, 9H), 0.54 (q, *J* = 7.9 Hz, 6H).
^{13}C-NMR (101 MHz, $CDCl_3$): *δ*(ppm) = 221.8, 145.2, 114.0, 85.7, 76.7, 63.5, 51.9, 39.9, 34.7, 27.8, 26.0, 24.8, 22.9, 22.8, 7.0, 6.4.
HRMS(ESI): Calcd for $C_{20}H_{36}O_3SiNa$ $[M+Na]^+$: 375.2331; found: 375.2330.
$[\alpha]_D^{26}$ = +39.1 (*c* = 1.3, $CHCl_3$).

For determination of the relative stereoconfiguration, NOE, spectra were measured in methanol-d_4 since the change of solvent allowed to distinguish all methyl groups of the compound.
^{1}H-NMR (400 MHz, CD_3OD): *δ*(ppm) = 4.89–4.86 (m, 1H, H-11b), 4.74–4.71 (m, 1H, H-11a), 3.14 (q, *J* = 10.8, 8.1 Hz, 1H, H-9), 2.23–2.11 (m, 1H, H-7a), 1.95–1.60 (m, 7H, H-{3,4,7b,8}), 1.69–1.67 (m, 3H, CH_3-14), 1.32 (s, 3H, CH_3-13), 1.26 (s, 3H, CH_3-12), 0.94 (t, *J* = 7.9 Hz, 9H, OTES), 0.62–0.54 (m, 6H, OTES);
^{13}C-NMR (101 MHz, CD_3OD): *δ*(ppm) = 221.8 (C-1), 146.7 (C-10), 114.2 (C-11), 87.1 (C-6), 76.8 (C-2), 64.4 (C-5), 53.0 (C-9), 40.9 (C-7), 36.4 (C-3), 28.6 (C-8), 26.6 (C-13), 25.9 (C-4), 23.1 (C-14), 22.5 (C-12), 7.3 (OTES).

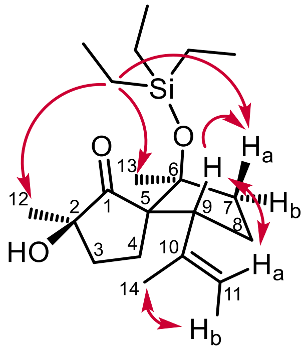

Figure S3. Signal assignment and relevant NOE correlations for α-hydroxyl ketone **S12**; spectra measured in methanol-*d*4.

(Chloromethyl)dimethylsilyl trifluoromethanesulfonate (S13)

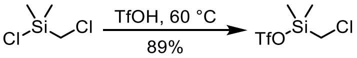

S13

A flame-dried 50 mL Schlenk tube capped with a rubber septum was charged under argon with triflic acid (6.6 mL, 74.8 mmol, 1.0 equiv). Chloro(chloromethyl)dimethylsilane (10.0 mL, 75.9 mmol, 1.0 equiv) was added carefully over 5 min at r.t. and the rubber septum was replaced by a glass stopper. The colorless mixture was stirred at 60 °C for 13 h, was allowed to cool to r.t. and the glass stopper was replaced with an argon-flushed flame-dried distillation apparatus. Distillation from the reaction flask delivered (chloromethyl)dimethylsilyl trifluoromethanesulfonate (**S13**) (17.4 g, 67.8 mmol, 89%) as a colorless liquid.

b.p. (22 mbar) = 64–65 °C.
^{1}H-NMR (400 MHz, C_6D_6): *δ*(ppm) = 2.39 (s, 2H), 0.09 (s, 6H).
^{13}C-NMR (101 MHz, C_6D_6): *δ*(ppm) = 118.9 (q, *J* = 317.5 Hz), 27.0, –3.6.

Oxasilolane S15

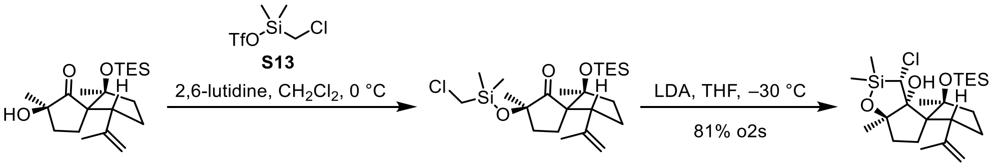

2,6-Lutidine (6.2 mL, 53.9 mmol, 4.0 equiv) was added to a stirred solution of hydroxy ketone **S12** (4.75 g, 13.5 mmol, 1.0 equiv) in anhydrous CH_2Cl_2 (68 mL) at 0 °C, followed by dropwise addition of silyl triflate **S13** (6.92 g, 27.0 mmol, 2.0 equiv) over 15 min. The solution was allowed to warm to r.t. and stirring was continued for 30 min. Water (35 mL) was added under vigorous stirring, the phases were separated and the aqueous phase was extracted with CH_2Cl_2 (3x 50 mL). The combined organic phases were washed with brine (50 mL), dried over Na_2SO_4 and concentrated under reduced pressure. The oily residue was coevaporated with anhydrous benzene three times and dried under fine vacuum overnight. The so obtained crude silyl ether **S14** was pure enough to be used in the subsequent step without further purification.

Analytical data of silyl ether **S14**:[5]
$\mathbf{R_f}$ (PE:EtOAc = 25:1; vanillin) = 0.68 (dark purple).
^{1}H-NMR (400 MHz, C_6D_6): δ(ppm) = 4.88–4.85 (m, 1H), 4.80–4.77 (m, 1H), 3.30–3.23 (m, 1H), 2.87–2.79 (m, 2H), 2.36–2.24 (m, 1H), 1.84–1.61 (m, 5H), 1.58–1.55 (m, 3H), 1.46–1.35 (m, 2H), 1.34 (s, 3H), 1.02–1.00 (m, 3H), 0.97 (t, *J* = 7.9 Hz, 9H), 0.57–0.50 (m, 6H), 0.37 (s, 3H), 0.35 (s, 3H).
^{13}C-NMR (101 MHz, C_6D_6): δ(ppm) = 218.6, 145.4, 114.4, 85.7, 80.0, 63.0, 52.3, 39.6, 37.4, 31.4, 28.1, 26.0, 24.9, 23.0, 22.7, 7.2, 6.7, −0.7.
HRMS(ESI): Calcd for $C_{23}H_{43}ClO_3Si_2Na$ $[M+Na]^+$: 481.2337; found: 481.2341.
$[\alpha]_D$ = n.d.[6]

Crude silyl ether **S14** was dissolved in anhydrous THF (68 mL) and the colorless solution was cooled to −30 °C. A freshly prepared LDA solution (1.0 M in THF, 40.0 mL, 40.0 mmol, 3.0 equiv) was added dropwise over 5 min at −30 °C and stirring at this temperature was continued for 3.5 h. Aqueous NH_4Cl (sat., 50 mL) was added at −30 °C and the mixture was allowed to warm to r.t. under vigorous stirring. The phases were separated and the aqueous phase was extracted with MTBE (3x 50 mL). The combined organic phases were washed with brine (50 mL), dried over Na_2SO_4 and concentrated under reduced pressure. After purification by column chromatography (PE:EtOAc = 100:1), oxasilolane **S15** (5.02 g, 10.9 mmol, 81% o2s) was obtained as an off-white solid.

$\mathbf{R_f}$ (PE:EtOAc = 50:1; vanillin) = 0.41 (purple).
^{1}H-NMR (400 MHz, $CDCl_3$): δ(ppm) = 5.23–5.19 (m, 1H, H-12a), 4.87–4.84 (m, 1H, H-12b), 4.86 (s, 1H, O*H*), 4.13 (s, 1H, H-1), 3.85 (t, *J* = 8.7 Hz, 1H, H-10), 1.89–1.86 (m, 3H, CH_3-17), 1.85–1.52 (m, 8H, H-{4,5,8,9}), 1.51 (s, 3H, CH_3-16), 1.38 (s, 3H, CH_3-15), 1.00 (t, *J* = 7.9 Hz, 9H, OTES, CH_3), 0.72 (q, *J* = 7.9 Hz, 6H, OTES, CH_2), 0.31 (s, 3H, CH_3-14), 0.24 (s, 3H, CH_3-13).
^{13}C-NMR (101 MHz, $CDCl_3$): δ(ppm) = 148.5 (C-11), 114.9 (C-12), 91.5 (C-3), 89.1 (C-7), 88.3 (C-2), 64.3 (C-6), 51.2 (C-1), 49.3 (C-10), 39.2 (C-4), 38.2 (C-8), 28.0 (C-5), 27.4 (C-9), 24.8 (C-16), 24.6 (C-1
5), 21.8 (C-17), 7.2 (OTES), 6.6 (OTES), −1.1 (C-14), −1.5 (C-13).
HRMS(ESI): Calcd for $C_{23}H_{43}ClO_3Si_2Na$ $[M+Na]^+$: 481.2337; found: 481.2338.
m.p.(CH_2Cl_2) = 45–47 °C.
$[\alpha]_D^{26}$ = +13.9 (*c* = 1.7, $CHCl_3$).

[5] Pure samples of **S14** were obtained by column chromatography (PE:EtOAc = 250:1 to 100:1).
[6] Silyl ether **S14** was only isolated and characterized for reaction optimization during the scouting of the synthesis with racemic intermediates. For the asymmetric synthesis, only the two-step procedure described here was used. Therefore the optical rotation of **S14** was not determined.

The relative stereoconfiguration was assigned based on NOE experiments:

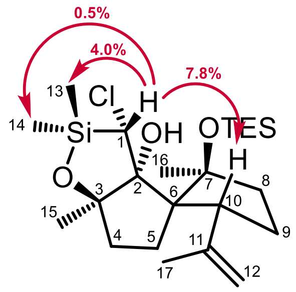

Figure S4. Signal assignment and relevant NOE correlations for oxasilolane **S15**.

Trimethylsilyl epoxide S16

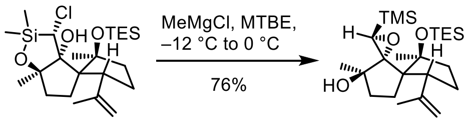

S15 **S16**

A 100 mL two-neck flask, equipped with a stir bar, an internal thermometer and a "fake-Schlenk head",[7] was charged with oxasilolane **S15** (2.00 g, 4.36 mmol, 1.0 equiv) and anhydrous MTBE (43 mL). The colorless solution was cooled with an acetone-ice bath under vigorous stirring until the internal temperature reached −12 °C. MeMgCl (3.0 M, 4.4 mL, 13.2 mmol, 3.0 equiv) was added quickly over 15 s. Addition of the nucleophile was accompanied by a swift gas evolution, a rise in internal temperature to −1 °C, as well as the formation of a turbid reaction mixture. Stirring was continued under cooling for 15 min. The reaction was quenched by careful addition of aqueous NH_4Cl (sat., 10 mL) until no further gas evolution was observed. The mixture was allowed to warm to r.t., the phases were separated and the aqueous phase was extracted with MTBE (3x 10 mL). The combined organic phases were concentrated under reduced pressure and purification by column chromatography (PE:EtOAc = 25:1 to 11:1) gave TMS epoxide **S16** (1.45 g, 3.30 mmol, 76%) as a sticky colorless oil.

R_f (PE:EtOAc = 25:1; vanillin) = 0.33 (dark blue).
^{1}H-NMR (400 MHz, C_6D_6): δ(ppm) = 4.99–4.93 (m, 2H, CH_2-12), 2.81 (t, J = 9.4 Hz, 1H, H-10), 2.12–2.00 (m, 2H, H-{4a,5a}), 2.07 (s, 1H, H-1), 2.00–1.90 (m, 1H, H-8a), 1.77 (s, 3H, CH_3-15), 1.67–1.52 (m, 2H, H-{5b,9a}), 1.51–1.33 (m, 3H, H-{4b,8b,9b}), 1.20 (s, 3H, CH_3-14), 1.13 (s, 3H, CH_3-13), 1.08 (t, J = 7.9 Hz, 9H, OTES, CH_3), 0.89 (bs, 1H, OH), 0.79–0.69 (m, 6H, OTES, CH_2), 0.21 (s, 9H, TMS).
^{13}C-NMR (101 MHz, C_6D_6): δ(ppm) = 146.7 (C-11), 112.7 (C-12), 86.1 (C-7), 83.4 (C-2), 81.0 (C-3), 57.9 (C-6), 53.9 (C-1), 50.6 (C-10), 40.0 (C-8), 38.7 (C-4), 29.0 (C-5), 27.2 (C-14), 27.1 (C-9), 24.6 (C-15), 22.9 (C-13), 7.6 (OTES), 7.3 (OTES), −0.6 (TMS).
HRMS(ESI): Calcd for $C_{24}H_{46}O_3Si_2Na$ $[M+Na]^+$: 461.2883; found: 461.2885.
$[\alpha]_D^{26}$ = +31.6 (c = 1.1, $CHCl_3$).

The relative stereoconfiguration was assigned based on NOE experiments:

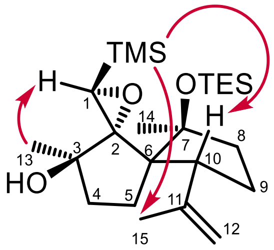

Figure S5. Signal assignment and relevant NOE correlations for TMS-epoxide **S16**.

[7] Y-shaped two-neck adapter; the joint of the straight neck is equipped with a rubber septum and the bend neck is equipped with an inert gas inlet.

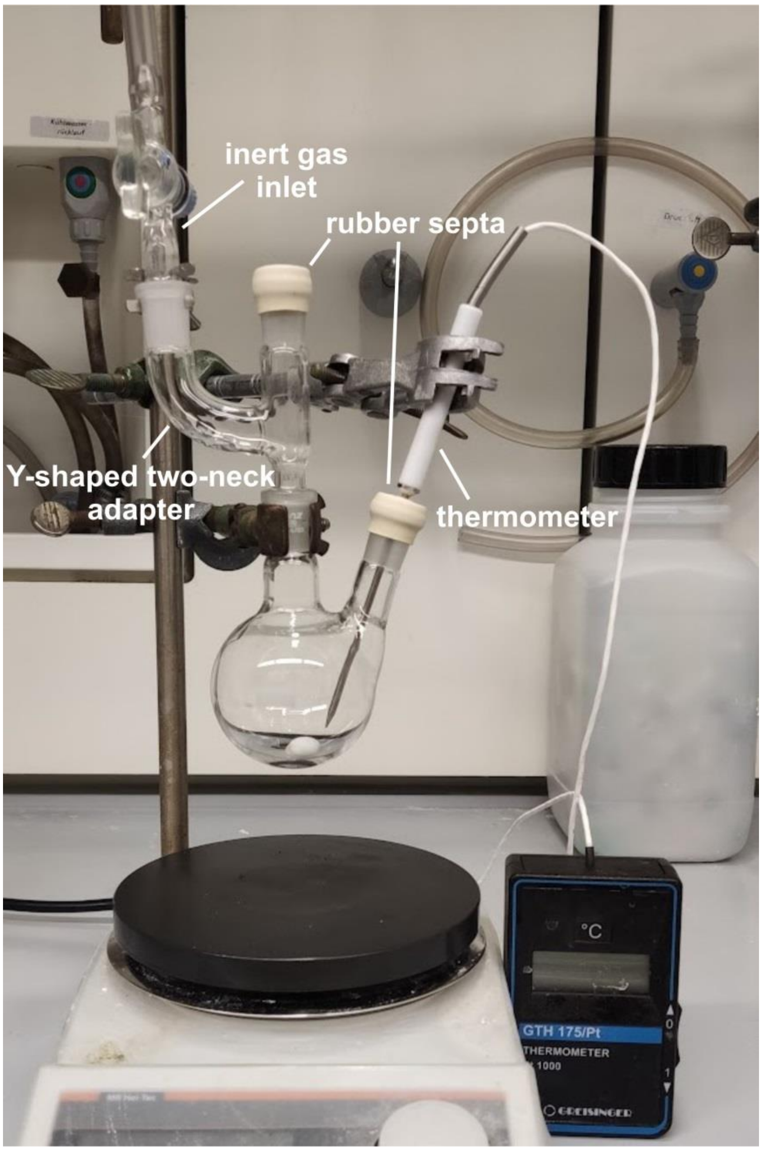

Figure S6. Photograph of the experimental setup used for the preparation of trimethylsilyl epoxide **S16**.

Aldehyde S17

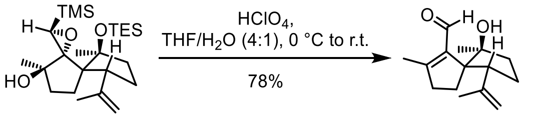

S16 **S17**

To a solution of TMS epoxide **S16** (1.11 g, 2.52 mmol, 1.0 equiv) in a 4:1 mixture of THF/water (25 mL) was added $HClO_4$ (45.5 µL, 756 µmol, 0.3 equiv) dropwise at 0 °C. After 30 min at 0 °C, the solution was allowed to warm to r.t. and stirred for 24h. The slightly yellow reaction was quenched with aqueous $NaHCO_3$ (sat., 10 mL) and the phases were separated. The aqueous phase was extracted with MTBE (3x 20 mL) and the combined organic phases were washed with brine (20 mL), dried over Na_2SO_4 and all volatiles were removed under reduced pressure. Purification by column chromatography (PE:EtOAc = 4:1) gave aldehyde **S17** (458 mg, 1.95 mmol, 78%) as a sticky colorless oil that crystallized in the freezer to give a yellow solid.

$\mathbf{R_f}$ (PE:EtOAc = 2:1; vanillin) = 0.47 (dark-blue).
^{1}H-NMR (400 MHz, C_6D_6): δ(ppm) = 9.76 (s, 1H), 4.95–4.91 (m, 1H), 4.79–4.76 (m, 1H), 4.36 (bs, 1H), 3.91–3.83 (m, 1H), 2.00–1.73 (m, 4H), 1.70–1.57 (m, 3H), 1.56–1.54 (m, 3H), 1.50–1.46 (m, 3H), 1.16–1.09 (m, 1H), 1.08 (s, 3H).
^{13}C-NMR (101 MHz, C_6D_6): δ(ppm) = 191.8, 167.4, 146.3, 138.4, 112.4, 84.8, 68.0, 47.9, 38.3, 37.9, 27.5, 25.2, 24.6, 23.1, 14.8.
HRMS(ESI): Calcd for $C_{15}H_{22}O_2Na$ $[M+Na]^+$: 257.1517; found: 257.1515.
m.p. (CH_2Cl_2) = 74−77 °C.
$[\alpha]_D^{24}$ = +104.7 (c = 1.3, $CHCl_3$).

Diol S18

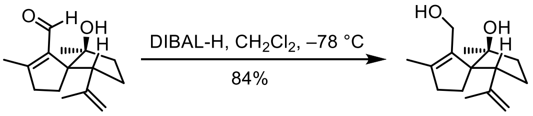

S17 **S18**

DIBAL-H (1.0 M in THF, 7.5 mL, 7.50 mmol, 2.0 equiv) was added dropwise to a stirred solution of aldehyde **S17** (870 mg, 3.71 mmol, 1.0 equiv) in anhydrous THF (37 mL) at −78 °C. Stirring was continued at this temperature for 45 min, before aqueous potassium sodium tartrate (sat., 40 mL) was added and the mixture was allowed to warm to r.t.. The phases were separated and the aqueous phase was extracted with MTBE (3x 30 mL). The combined organic phases were washed with brine (40 mL), dried over Na_2SO_4, and all volatiles were removed under reduced pressure. Purification by column chromatography (PE:EtOAc = 2:1) gave diol **S18** (740 mg, 3.13 mmol, 84%) as a white solid.

$\mathbf{R_f}$ (PE:EtOAc = 2:1; vanillin) = 0.19 (dark-purple).
^{1}H-NMR (400 MHz, $CDCl_3$): δ(ppm) = 4.86 (s, 1H), 4.70 (s, 1H), 4.31 (d, J = 11.4 Hz, 1H), 4.04 (d, J = 11.4 Hz, 1H), 3.28–3.19 (m, 1H), 2.43 (bs, 2H), 2.34–2.22 (m, 1H), 2.18–2.07 (m, 1H), 1.91–1.62 (m, 5H), 1.82 (s, 3H), 1.55 (s, 3H), 1.41–1.32 (s, 1H) , 1.18 (s, 3H).
^{13}C-NMR (101 MHz, $CDCl_3$): δ(ppm) = 146.6, 143.7, 135.3, 111.6, 85.1, 68.2, 56.1, 48.0, 37.2, 36.3, 27.5, 24.6, 24.4, 23.0, 14.7.
HRMS(ESI): Calcd for $C_{15}H_{24}O_2Na$ $[M+Na]^+$: 259.1674; found: 259.1676.
m.p. (CH_2Cl_2) = 103–106 °C.
$[\alpha]_D^{29}$ = +125.4 (c = 1.2, $CHCl_3$).

Diphenyl silyl protected diol S19

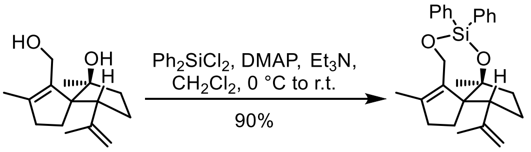

S18 **S19**

Diol **S18** (740 mg, 3.13 mmol, 1.0 equiv) was dissolved in anhydrous CH_2Cl_2 (31 mL) and the solution was cooled to 0 °C. 4-DMAP (382 mg, 3.13 mmol, 1.0 equiv), Et_3N (1.7 mL, 12.5 mmol, 4.0 equiv) and Ph_2SiCl_2 (725 µL, 3.44 mmol, 1.1 equiv) were added successively and the turbid reaction mixture was allowed to warm to r.t.. Stirring at r.t. was continued for 4 h, before the reaction was

quenched by addition of water (20 mL), and the mixture was diluted with CH_2Cl_2 (20 mL). The phases were separated and the aqueous phase extracted with CH_2Cl_2 (3x 20 mL). The combined organic phases were washed with brine (25 mL), dried over Na_2SO_4 and all volatiles were removed under reduced pressure. Purification by column chromatography (PE:EtOAc = 19:1) afforded protected diol **S19** (1.18 g, 2.83 mmol, 90%) as a white solid.

$\mathbf{R}_f$ (PE:EtOAc = 25:1; vanillin) = 0.57 (dark blue).
^{1}H-NMR (400 MHz, $CDCl_3$): δ(ppm) = 7.72–7.62 (m, 4H), 7.47–7.30 (m, 6H), 4.92–4.88 (m, 1H), 4.82–4.76 (m, 1H), 4.58 (d, J = 12.3 Hz, 1H), 4.25 (d, J = 12.3 Hz, 1H), 3.47–3.34 (m, 1H), 2.35–2.13 (m, 2H), 2.11–1.96 (m, 2H), 1.92 (s, 3H), 1.89–1.77 (m, 3H), 1.57 (s, 3H), 1.39 (ddd, J = 13.6, 8.8, 2.8 Hz, 1H), 1.10 (s, 3H).
^{13}C-NMR (101 MHz, $CDCl_3$): δ(ppm) = 146.9, 143.5, 135.3, 135.1, 134.90, 134.88, 134.7, 130.1, 130.0, 127.9, 127.7, 111.6, 91.2, 69.4, 56.9, 48.2, 38.2, 36.3, 27.6, 24.6, 24.4, 22.9, 14.7.
HRMS(ESI): Calcd for $C_{27}H_{32}O_2SiNa$ $[M+Na]^+$: 439.2062; found: 439.2065.
m.p. (CH_2Cl_2) = 56−60 °C.
$[\alpha]_D^{28}$ = –15.2 (c = 2.3, $CHCl_3$).

Enone S20

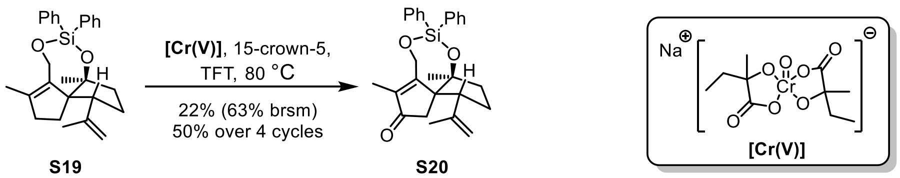

S19 S20

The allylic oxidation of **S19** was performed according to a modification of the procedure described by Baran and co-workers.[10]
Sodium bis(2-hydroxy-2-methyl-butyrato)oxochromate(V) (2.16 g, 6.67 mmol, 2.0 equiv) was added in one portion to a solution of protected diol **S19** (1.39 g, 3.34 mmol, 1.0 equiv) and 15-crown-5 (2.0 mL, 10.1 mmol, 3.0 equiv) in anhydrous trifluorotoluene (20.0 mL) at r.t.. The mixture was heated to 80 °C in a sealed Schlenk tube for 24 h. After cooling to r.t., the reaction mixture was applied on silica and the crude material was purified *via* column chromatography (PE:EtOAc = 15:1) to give enone **S20** (318 mg, 0.74 mmol, 22% (63% brsm)) as a white solid. The recovered starting material (899 mg, 2.16 mmol, 65%) was recycled for further oxidations, affording in total 713 mg (1.66 mmol, 50% over 4 cycles) of enone **S20**.

R_f (PE:EtOAc = 4:1; vanillin) = 0.60 (light blue).
^{1}H-NMR (400 MHz, $CDCl_3$): δ(ppm) = 7.71–7.66 (m, 2H), 7.63–7.58 (m, 2H), 7.49–7.44 (m, 1H), 7.43–7.37 (m, 3H), 7.36–7.30 (m, 2H), 4.95–4.92 (m, 1H), 4.79 (bs, 1H), 4.76 (d, J = 12.3 Hz, 1H), 4.61 (d, J = 12.3 Hz, 1H), 3.65 (t, J = 8.8 Hz, 1H), 2.45 (d, J = 18.8 Hz, 1H), 2.22–2.07 (m, 2H), 1.96 (d, J = 18.8 Hz, 1H), 1.91 (s, 3H), 1.89–1.72 (m, 2H), 1.51 (s, 3H), 1.06 (s, 3H).
^{13}C-NMR (100 MHz, $CDCl_3$): δ(ppm) = 207.9, 167.1, 144.4, 142.4, 134.9, 134.7, 133.8, 133.3, 130.7, 130.6, 128.1, 127.9, 113.4, 89.9, 63.3, 56.9, 48.4, 41.4, 39.4, 25.1, 24.8, 22.4, 8.4.
HRMS(ESI): Calcd for $C_{27}H_{30}O_3SiNa$ $[M+Na]^+$: 453.1862; found: 453.1868.
m.p. (CH_2Cl_2) = 127 °C.
$[\alpha]_D^{27}$ = +14.2 (c = 1.3, $CHCl_3$).

Epoxide S21

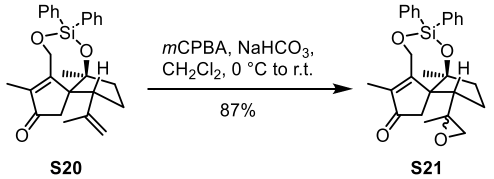

S20 S21

*m*CPBA (75% in water, 705 mg, 3.07 mmol, 1.5 equiv) was added in one portion to a suspension of enone **S20** (880 mg, 2.04 mmol, 1.0 equiv) and $NaHCO_3$ (515 mg, 6.13 mmol, 3.0 equiv) in CH_2Cl_2 (40 mL) at 0 °C. The mixture was allowed to warm to r.t. and stirred for 15 h. It was then cooled to 0 °C and quenched by addition of aqueous $Na_2S_2O_3$ (sat., 20 mL) and aqueous $NaHCO_3$ (sat., 20 mL). The phases were separated and the aqueous phase was extracted with CH_2Cl_2 (3x 40 mL). The combined organic phases were washed with brine (25 mL), dried over Na_2SO_4 and the solvent was removed under reduced pressure. Purification *via* column chromatography (PE:EtOAc = 4:1) gave a 2.1:1 mixture of epoxides **S21a** and **S21b** (797 mg, 1.78 mmol, 87%) as a white solid.

m.p.(CH_2Cl_2) = 115–150 °C (mixture of diastereomers).
The diastereomers were not separated for the subsequent Ti-mediated cyclization. They can however be separated by column chromatography to give pure epoxides **S21a** and **S21b**. The relative stereoconfiguration of the major isomer **S21a** was determined *via* X-ray single crystal diffraction.

Major diastereomer **S21a**:

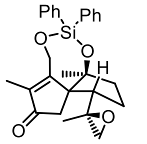

S21a

R_f (PE:EtOAc = 7:3; vanillin) = 0.16 (light blue).
^{1}H-NMR (400 MHz, $CDCl_3$): *δ*(ppm) = 7.70–7.64 (m, 2H), 7.62–7.55 (m, 2H), 7.50–7.45 (m, 1H), 7.44–7.37 (m, 3H), 7.36–7.29 (m, 2H), 4.78 (d, *J* = 12.3 Hz, 1H), 4.56 (d, *J* = 12.3 Hz, 1H), 3.44 (t, *J* = 9.2 Hz, 1H), 2.77 (d, *J* = 4.1 Hz, 1H), 2.60 (d, *J* = 18.9 Hz, 1H), 2.53 (d, *J* = 4.2 Hz, 1H), 2.24–2.11 (m, 1H), 2.10–2.00 (m, 2H), 1.96 (s, 3H), 1.73–1.63 (m, 1H), 1.46–1.35 (m, 1H), 1.09 (s, 3H), 1.01 (s, 3H).
^{13}C-NMR (101 MHz, CDCl3): δ(ppm) = 207.3, 167.0, 142.8, 134.8, 134.7, 133.6, 133.1, 130.7, 130.6, 128.2, 128.0, 90.3, 62.6, 57.0, 56.5, 51.1, 45.7, 42.0, 39.2, 23.2, 22.5, 21.4, 8.4.
HRMS(ESI): Calcd for $C_{27}H_{30}O_{4Si}Na$ [M+Na]+: 469.1811; found: 469.1809.
m.p. (CH_2Cl_2) = 146–149 °C.
$[\alpha]_D^{27}$ = +5.6 (*c* = 1.0, $CHCl_3$).

Minor diastereomer **S21b**:

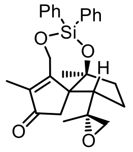

S21b

R_f (PE:EtOAc = 7:3; vanillin) = 0.23 (green).
^{1}H-NMR (400 MHz, $CDCl_3$): *δ*(ppm) = 7.71–7.65 (m, 2H), 7.61–7.55 (m, 2H), 7.50–7.45 (m, 1H), 7.44–7.37 (m, 3H), 7.34–7.29 (m, 2H), 4.82 (d, *J* = 12.2 Hz, 1H), 4.55 (d, *J* = 12.2 Hz, 1H), 3.44 (t, *J* = 9.3 Hz, 1H), 2.98 (d, *J* = 19.3 Hz, 1H), 2.54 (d, *J* = 4.6 Hz, 1H), 2.33 (d, *J* = 4.6 Hz, 1H), 2.09–1.99 (m, 2H), 1.97 (s, 3H), 1.95–1.85 (m, 1H), 1.75–1.65 (m, 1H), 1.56–1.44 (m, 1H), 1.06 (s, 3H), 0.97 (s, 3H).
^{13}C-NMR (101 MHz, $CDCl_3$): δ(ppm) = 207.6, 166.8, 142.9, 134.7, 134.6, 133.6, 133.2, 130.53, 130.47, 128.0, 127.8, 90.5, 62.9, 56.6, 55.9, 51.0, 44.6, 42.3, 38.8, 22.0, 21.1, 21.0, 8.3.
HRMS(ESI): Calcd for $C_{27}H_{30}O_4SiNa$ $[M+Na]^{+:}$ 469.1811; found: 469.1806.
m.p.(CH_2Cl_2) = 136–139 °C.
$[\alpha]_D^{28}$ = –4.3 (*c* = 1.5, $CHCl_3$).

Tricycle S22

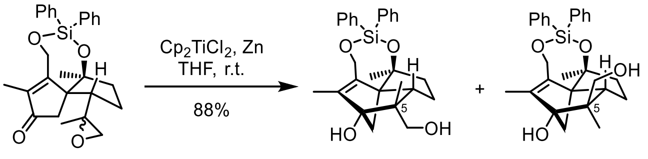

**S21**		**S22**		5-*epi*-**S22**
	**d.r.**	**1**	**:**	**1.6**

For the reductive cyclization of **S21**, a variation of the procedure published by Bermejo and co-workers was used.[11]
Degassed[8] THF (60 mL) was added to Zn powder (813 mg, 12.4 mmol, 8.9 equiv) and Cp_2TiCl_2 (1.03 g, 4.15 mmol, 3.0 equiv) under an argon atmosphere. The green mixture was stirred at r.t. for 1 h, before a solution of epoxide **S21** (619 mg, 1.39 mmol, 1.0 equiv) in degassed[8] THF (43 mL) was added dropwise over 30 min. The brown reaction mixture was stirred at r.t. for 1 h, before aqueous NaH_2PO_4 (10%, 30 mL) and brine (20 mL) were added. The phases were separated and the aqueous phase was extracted with EtOAc (3x 50 mL). The collected organic phases were dried over Na_2SO_4 and all volatiles were removed under reduced pressure. Purification by column chromatography (PE:EtOAc = 7:3) afforded a 1:1.6 mixture of tricycles **S22** and 5-*epi*-**S22** (554 mg, 1.23 mmol, 88%) as a white solid.

R_f (PE:EtOAc = 7:3; vanillin) = 0.09 (dark blue).
^{1}H-NMR (400 MHz, C_6D_6): *δ*(ppm) = 7.96–7.90 (m, 2H, major+minor Ph_2Si), 7.88–7.82 (m, 2H, major+minor Ph_2Si), 7.23–7.17 (m, 6H, major+minor Ph_2Si), 4.59 (d, *J* = 14.5 Hz, 1H, major H-11a), 4.57 (d, *J* = 14.5 Hz, 1H, minor H-11a), 4.49–4.41 (m, 1H,

[8] The freeze-pump-thaw technique was used.

major+minor H-11b), 3.73 (d, J = 10.6 Hz, 1H, minor H-13a), 3.40 (d, J = 10.5 Hz, 1H, minor H-13b), 2.88 (d, J = 9.8 Hz, 1H, major H-13a), 2.75 (d, J = 9.8 Hz, 1H, major H-13b), 2.60 (bs, 1H, OH), 2.16–2.07 (m, 1H(major H-2a) + 2H(minor H-2a, H-4)), 2.03–1.83 (m, 1H, major+minor H-3a), 1.75 (dd, J = 11.0, 5.5 Hz, 1H, major H-4), 1.67–1.55 (m, 1H, major+minor H-2b, HSQC), 1.63–1.61 (m, 3H, major CH_3-12), 1.60–1.58 (m, 3H, minor CH_3-12), 1.54–1.47 (m, 1H, major H-3b, HSQC), 1.50 (d, J = 7.7 Hz, 1H, minor H-8a), 1.44 (s, 2H, major CH_2-8), 1.38–1.35 (m, 1H, minor H-8b), 1.34 (s, 3H, minor CH_3-15), 1.33–1.26 (m, 1H, minor H-3b, HSQC), 1.32 (s, 3H, major CH_3-15), 1.16 (s, 3H, major CH_3-14), 0.85 (s, 3H, minor CH_3-14).
^{13}C-NMR (101 MHz, C_6D_6): δ(ppm) = 142.0 (minor C-6), 141.4 (major C-6), 138.6 (minor C-10), 138.5 (major C-10), 135.60 (minor Ph_2Si), 135.56 (major Ph_2Si), 135.43 (minor Ph_2Si), 135.41 (major Ph_2Si), 135.2 (major+minor Ph_2Si), 134.6 (major Ph_2Si), 134.5 (minor Ph_2Si), 130.6 (minor Ph_2Si), 130.5 (major Ph_2Si), 130.4 (major+minor Ph_2Si), 128.2 (major Ph_2Si), 128.1 (minor Ph_2Si), 90.5 (minor C-7), 89.8 (major C-7), 83.8 (major C-1), 83.5 (minor C-1), 72.2 (major C-13), 70.5 (minor C-13), 66.7 (major C-9), 66.0 (minor C-9), 58.9 (minor C-11), 58.8 (major C-11), 55.9 (minor C-4), 53.7 (minor C-8), 53.2 (major C-8), 52.4 (major C-4), 48.6 (major C-5), 46.2 (minor C-5), 43.71 (minor C-2), 43.66 (major C-2), 25.53 (minor C-15), 25.50 (major C-15), 22.4 (major C-3), 22.3 (minor C-14), 20.7 (minor C-3), 18.2 (major C-14), 10.9 (minor C-12), 10.3 (major C-12).[9]
HRMS(ESI): Calcd for $C_{27}H_{32}O_4NaSi$ $[M+Na]^+$: 471.1968; found: 471.1979.
m.p. (CH_2Cl_2) = 43–50 °C.
$[\alpha]_D^{28}$ = –35.0 (c = 1.2, $CHCl_3$).

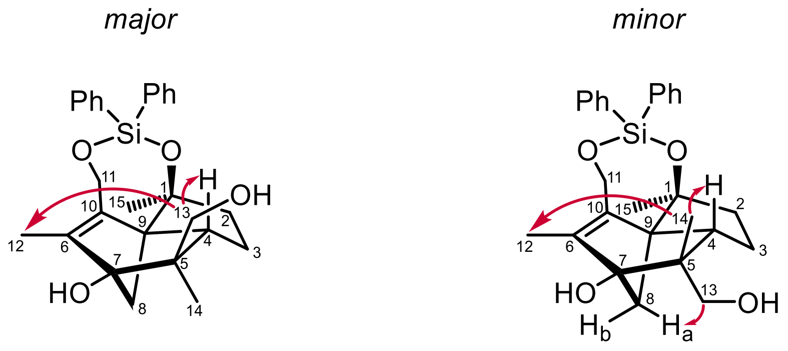

Figure S7. Signal assignment and relevant NOE correlations for both diastereomers of tricycle **S22**.

Epoxide S24

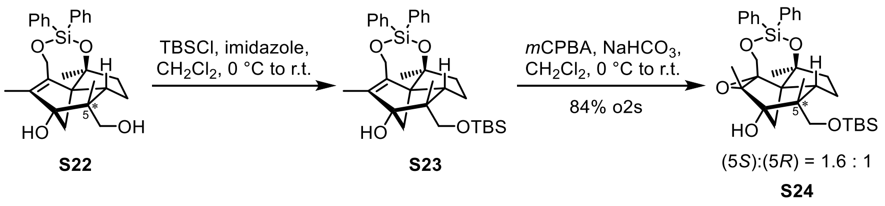

TBSCl (514 mg, 3.43 mmol, 2.3 equiv) was added to a solution of tricycle **S22** (680 mg, 1.52 mmol, 1.0 equiv) and imidazole (310 mg, 4.55 mmol, 3.0 equiv) in anhydrous CH_2Cl_2 (15.0 mL) at 0 °C. The mixture was stirred at r.t. for 13 h, before additional imidazole (103 mg, 1.52 mmol, 1.0 equiv) and TBSCl (172 mg, 1.14 mmol, 0.75 equiv) were added. After 1 h, brine (10 mL) was added and the phases were separated. The aqueous phase was extracted with CH_2Cl_2 (3x 10 mL). The combined organic phases were dried over Na_2SO_4 and all volatiles were removed under reduced pressure. Purification by column chromatography (PE:EtOAc = 20:1) gave impure TBS-protected product **S23** (860 mg) as a white solid, which was used in the next step without further purification.

Impure allylic alcohol **S23** (860 mg) was dissolved in CH_2Cl_2 (27 mL) and $NaHCO_3$ (343 mg, 4.08 mmol, 2.7 equiv) was added. The mixture was cooled to 0 °C and *m*CPBA (70% in water, 670 mg, 2.72 mmol, 1.8 equiv) was added in one portion. The mixture was allowed to warm to r.t. and stirred for 2 h. The reaction was quenched by addition of aqueous $Na_2S_2O_3$ (sat., 10 mL) and aqueous $NaHCO_3$ (sat., 10 mL) at 0 °C, the phases were separated and the aqueous phase was extracted with CH_2Cl_2 (3x 20 mL). The combined organic phases were washed with brine (20 mL), dried over Na_2SO_4 and the solvent was removed under reduced pressure. Purification *via* column chromatography (PE:EtOAc = 10:1) gave epoxide **S24** (mixture of C-5 epimers, d.r. 1.6:1) (741 mg, 1.28 mmol, 84% o2s) as a white solid.

R_f (PE:EtOAc = 4:1; vanillin) = 0.39 (dark-pink).
^{1}H-NMR (500 MHz, C_6D_6): δ(ppm) = 7.97–7.92 (m, 2H, major+minor), 7.90–7.85 (m, 2H, major+minor), 7.26–7.17 (m, 6H, major+minor), 4.55 (d, J = 13.0 Hz, 1H, major), 4.53 (d, J = 13.1 Hz, 1H, minor), 3.93 (d, J = 10.0 Hz, 1H, minor), 3.87 (s, 1H, minor),

[9] For the ^{13}C-spectrum of **S22** (diastereomeric mixture) in C_6D_6 only seven carbon environments per diastereomer could be distinguished for the Ph_2Si-protecting group. We contribute this either to strong overlapping of the phenyl signals, or the respective signals lying under the solvent signal. The circumstance could not be unambiguously clarified by HSQC and HMBC analysis. ^{1}H-NMR and mass spectrometry however strongly confirm the identity of the substance.

3.75 (d, J = 13.0 Hz, 1H, major), 3.73 (d, J = 13.1 Hz, 1H, minor), 3.47 (d, J = 10.0 Hz, 1H, minor), 3.28 (d, J = 9.9 Hz, 1H, major), 3.13 (d, J = 9.9 Hz, 1H, major), 2.41 (s, 1H, major), 2.34–2.28 (m, 1H, minor), 2.14–1.93 (m, 4H(major) + 3H(minor)), 1.63–1.48 (m, 2H(major) + 2H(minor)), 1.42 (s, 3H, major), 1.39 (s, 3H, minor), 1.37 (s, 3H, minor), 1.33 (s, 3H, major), 1.16–1.12 (m, 3H(major) + 1H(minor)), 1.01 (d, J = 9.1 Hz, 1H, major), 0.93 (s, 3H, minor), 0.90 (s, 9H, minor), 0.86 (s, 9H, major), 0.00 (s, 3H, minor), –0.01 (s, 3H, minor), –0.09 (s, 3H, major), –0.13 (s, 3H, major).
^{13}C-NMR (126 MHz, C_6D_6): δ(ppm) = 135.5*, 135.4, 135.3 (both diastereomers), 134.82*, 134.75, 134.52, 134.51*, 130.7 (both diastereomers), 130.57, 130.55*, 128.2 (both diastereomers), 87.5*, 87.2, 84.2, 84.0*, 71.0, 70.7*, 64.9, 64.2*, 64.1, 63.9*, 62.3*, 61.7, 60.7*, 60.5, 54.3*, 49.9, 49.5, 46.4*, 43.1, 42.8*, 37.1*, 36.6, 25.94, 25.90*, 24.50*, 24.45, 23.6*, 22.3, 21.0*, 19.4, 18.3*, 18.2, 12.6*, 11.7, –5.5, –5.6, –5.72*, –5.74*.[10]
HRMS(ESI): Calcd for $C_{33}H_{46}O_5NaSi_2$ $[M+Na]^+$: 601.2782; found: 601.2789.
m.p.(CH_2Cl_2) = 45–50 °C.
$[\alpha]_D^{28}$ = –29.4 (c = 1.0, $CHCl_3$).

Rearranged product S25

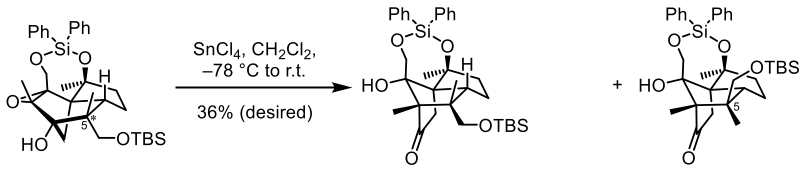

S24 (5S):(5R) = 1.6 : 1 **S25** 5-*epi*-**S25**

$SnCl_4$ (1.0 M in CH_2Cl_2, 2.1 mL, 2.10 mmol, 2.0 equiv) was added dropwise to a solution of epoxy alcohol **S24** (619 mg, 1.07 mmol, 1.0 equiv) in anhydrous dichloromethane (10.0 mL) at –78 °C. After 5 h, the reaction was quenched with water (5 mL) and allowed to warm to r.t.. The phases were separated and the aqueous phase was extracted CH_2Cl_2 (3x 10 mL). The combined organic phases were dried over Na_2SO_4 and all volatiles were removed under reduced pressure. Purification *via* column chromatography (PE:EtOAc = 19:1) gave rearranged product **S25** (222 mg, 0.38 mmol, 36% desired diastereomer) as a white solid.
R_f (PE:EtOAc = 5:1; vanillin) = 0.50 (dark pink).
^{1}H-NMR (600 MHz, C_6D_6): δ(ppm) = 7.84–7.81 (m, 2H, $SiPh_2$), 7.74–7.70 (m, 2H, $SiPh_2$), 7.20–7.13 (m, 6H, $SiPh_2$), 4.59 (d, J = 11.7 Hz, 1H, H-11a), 3.48 (d, J = 11.7 Hz, 1H, H-11b), 3.38 (d, J = 10.0 Hz, 1H, H-14a), 3.31 (d, J = 10.0 Hz, 1H, H-14b), 3.15–3.09 (m, 1H, H-4), 2.97 (s, 1H, OH), 2.49 (dd, J = 17.4, 1.9 Hz, 1H, H-8b), 2.33 (ddd, J = 14.6, 9.1, 5.6 Hz, 1H, H-2a), 1.89–1.83 (m, 1H, H-3a), 1.84 (d, J = 17.4 Hz, 1H, H-8a), 1.72 (ddd, J = 14.4, 11.2, 3.3 Hz, 1H, H-2b), 1.42 (dq, J = 11.9, 5.9 Hz, 1H, H-3b), 1.031 (s, 3H, CH_3-12), 1.027 (s, 3H, CH_3-15), 0.95 (s, 9H, OTBS), 0.85 (s, 3H, CH_3-13), 0.03 (s, 3H, OTBS), 0.01 (s, 3H, OTBS).
^{13}C-NMR (151 MHz, C_6D_6): δ(ppm) = 209.9 (C-7), 135.1 ($SiPh_2$), 134.9 ($SiPh_2$), 134.3 ($SiPh_2$), 133.8 ($SiPh_2$), 130.9 ($SiPh_2$), 130.7 ($SiPh_2$), 128.4 (HMBC, $SiPh_2$)[11], 128.3 (HMBC, $SiPh_2$)[11], 85.7 (C-10), 81.3 (C-1), 69.5 (C-6), 66.7 (C-14), 64.7 (C-9), 62.7 (C-11), 52.6 (C-4), 45.9 (C-2), 39.9 (C-8), 39.2 (C-5), 26.1 (OTBS), 25.0 (C-15), 23.6 (C-13), 19.8 (C-3), 18.4 (OTBS), 6.8 (C-12), –5.6 (OTBS), –5.7 (OTBS).
HRMS(ESI): Calcd for $C_{33}H_{46}O_5NaSi_2$ $[M+Na]^+$: 601.2782; found: 601.2780.
m.p. (CH_2Cl_2) = 158–160 °C.
$[\alpha]_D^{29}$ = –101.1 (c = 0.9, $CHCl_3$).

The relative configuration of **S25** was determined by NOE experiments:

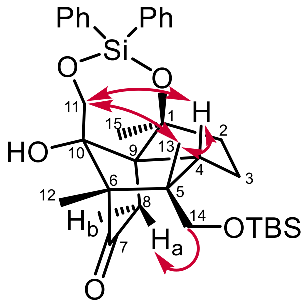

Figure S8. Signal assignment and relevant NOE correlations for the proposed relative stereoconfiguration of **S25**.

[10] For the ^{13}C-spectrum of **S22** (diastereomeric mixture) in C_6D_6 only seven carbon environments per diastereomer could be distinguished for the Ph_2Si-protecting group. We contribute this either to strong overlapping of the phenyl signals, or the respective signals lying under the solvent signal. The circumstance could not be unambiguously clarified by HSQC and HMBC analysis. ^{1}H-NMR and mass spectrometry however strongly confirm the identity of the substance.
[11] Signal underneath the solvent signal.

For the experiment outlined above, the undesired diastereomer 5-*epi*-**S25** was not isolated. During the scouting of the sequence with racemic intermediates, 5-*epi*-**S25** was isolated and characterized. 5-*epi*-**S25** was usually obtained in 21% yield alongside the desired product. Its relative stereoconfiguration was determined by X-ray crystallography (for details, see below).
Analytical data for (±)-5-*epi*-**S25**:[12]

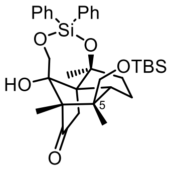

5-*epi*-**S25**

R*f* (PE:EtOAc, vanillin) = 0.42 (bright pink).
^{1}H-NMR (400 MHz, C_6D_6): *δ*(ppm) = 7.85–7.78 (m, 2H), 7.73–7.68 (m, 2H), 7.27–7.11 (m, 6H), 4.74 (d, *J* = 11.6 Hz, 1H), 3.53 (d, *J* = 11.6 Hz, 1H), 3.43–3.35 (m, 1H), 3.21 (d, *J* = 9.6 Hz, 1H), 3.09 (d, *J* = 9.6 Hz, 1H), 3.06 (s, 1H), 2.49 (dd, *J* = 17.3, 1.9 Hz, 1H), 2.37–2.28 (m, 1H), 1.78 (d, *J* = 17.3 Hz, 1H), 1.77–1.66 (m, 2H), 1.35–1.23 (m, 1H), 1.04 (s, 3H), 0.95 (s, 3H), 0.91 (s, 9H), 0.83 (s, 3H), –0.15 (s, 6H).
^{13}C-NMR (101 MHz, C_6D_6): *δ*(ppm) = 209.4, 135.3, 134.9, 134.4, 133.8, 130.8, 130.7, 128.4, 85.5, 81.6, 70.7, 69.1, 64.3, 63.0, 49.5, 45.9, 39.7, 39.2, 26.1, 24.9, 19.9, 18.4, 17.4, 5.2, –5.5, –5.7.[13]
HRMS(ESI): Calcd for $C_{33}H_{46}O_5NaSi_2$ $[M+Na]^+$: 601.2782; found: 601.2803.
m.p. (CH_2Cl_2) = 158–160 °C.

mono-TBS-protected tetraol S26

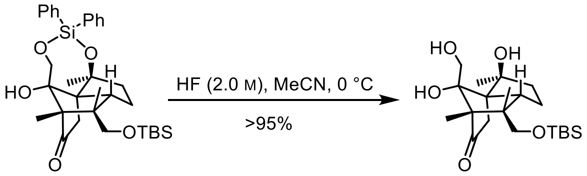

S25 **S26**

Tri-protected tetraol **S25** (238 mg, 411 µmol, 1.0 equiv) was dissolved in MeCN (7.5 mL) and HF (2.0 M in water, 820 µL, 1.64 mmol, 4.0 equiv) was added at 0 °C. Stirring at 0 °C was continued for 14 h, before aqueous $NaHCO_3$ (sat., 5 mL) and EtOAc (5 mL) were added and the mixture was allowed to warm to r.t.. The phases were separated and the aqueous phase extracted with EtOAc (4x 10 mL). The combined organic phases were dried over Na_2SO_4 and all volatiles were removed under reduced pressure. Purification by column chromatography (PE:EtOAc = 1:1 to 1:1.5) gave mono-TBS-protected tetraol **S26** (164 mg, 411 µmol, quant.) as a white solid.
Analytical data are in accordance with the data reported by Rychnovsky and Burns.[12]

R*f* (PE:EtOAc = 1:1; vanillin) = 0.57 (red).
^{1}H-NMR (400 MHz, $CDCl_3$): *δ*(ppm) = 4.39 (d, *J* = 12.1 Hz, 1H), 4.22 (bs, 1H), 3.39–3.30 (m, 1H), 3.28 (bs, 1H), 3.21 (d, *J* = 10.0 Hz, 1H), 3.12 (d, *J* = 10.0 Hz, 1H), 2.91 (ddd, *J* = 12.0, 8.2, 1.6 Hz, 1H), 2.57 (bs, 1H), 2.52 (dd, *J* = 17.5, 1.6 Hz, 1H), 2.18–2.08 (m, 1H), 2.09 (d, *J* = 17.5 Hz, 1H), 1.96 (ddd, *J* = 14.8, 9.0, 5.8 Hz, 1H), 1.84–1.74 (m, 1H), 1.52 (dq, *J* = 12.0, 5.8 Hz, 1H), 1.35 (s, 3H), 1.00 (s, 3H), 0.88 (s, 3H), 0.85 (s, 9H), –0.026 (s, 3H), –0.034 (s, 3H).
^{13}C-NMR (101 MHz, $CDCl_3$): *δ*(ppm) = 213.5, 84.1, 76.6, 69.7, 66.0, 63.1, 60.6, 51.5, 45.1, 39.5, 39.4, 25.9, 25.0, 23.5, 19.6, 18.2, 6.4, –5.6, –5.7.
HRMS(ESI): Calcd for $C_{21}H_{38}O_5SiNa$ $[M+Na]^+$: 421.2386; found: 421.2383.
m.p. (CH_2Cl_2) = 75–79 °C.
$[\alpha]_D^{25}$ = –14.9 (*c* = 1.3, $CHCl_3$).

---

12 5-*epi*-**S25** was only isolated and characterized during the racemic scouting of the synthesis. Therefore, no optical rotation was determined.
13 For the ^{13}C-spectrum of 5-*epi*-**S25** in C_6D_6 only seven carbon environments could be distinguished for the Ph_2Si-protecting group. We contribute this either to strong overlapping of the phenyl signals, or the respective signals lying under the solvent signal. The circumstance could not be unambiguously clarified by HSQC and HMBC analysis. ^{1}H-NMR and mass spectrometry however strongly confirm the identity of the substance.

Synthesis of carboxylic acid S28

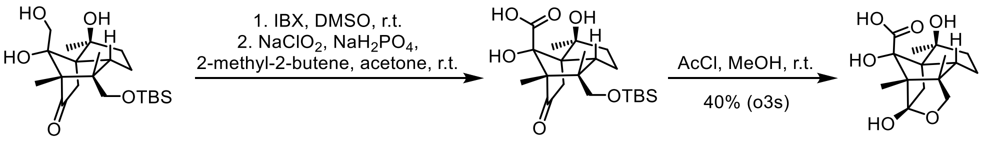

A slight variation of the procedure published by Burns and Rychnovsky was used for the synthesis of **S28**.[12]

IBX (75.3 mg, 269 µmol, 5.5 equiv) was added in one portion to a stirred solution of Rychnovsky's intermediate (**S26**) (19.5 mg, 48.9 µmol, 1.0 equiv) in anhydrous DMSO (325 µL) at r.t.. The walls of the flask were rinsed with CH_2Cl_2 (100 µL) to ensure that all starting material was inside the reaction mixture and stirring at r.t. was continued for 5 h 45 min. Water (1 mL) was added and the mixture was stirred vigorously for 5 min to achieve precipitation of IBX residues. The mixture was filtered over a fine sintered glass frit and the filter cake was washed with cold CH_2Cl_2 (3x 1 mL). The phases were separated and the aqueous phase was extracted with cold CH_2Cl_2 (2x 2 mL). The combined organic phases were washed with water (2 mL) and the resulting aqueous phase was back extracted with cold CH_2Cl_2 (2x 2 mL). The washing and back extraction procedure was repeated with $NaHCO_3/Na_2S_2O_3$ (4 mL), the combined organic phases were dried over Na_2SO_4 and all volatiles were removed under reduced pressure (rotavap bath temperature: 0 °C). The so obtained crude aldehyde was dried under vacuum for 30 min prior to the subsequent Pinnick oxidation.

The light-yellow residue was dissolved in acetone (548 µL), 2-methyl-2-butene (104 µL, 979 µmol, 20.0 equiv) and NaH_2PO_4 (2.0 M in water, 269 µL, 538 µmol, 11.0 equiv) were added at r.t. and the colorless solution was stirred at r.t. for 3 min. $NaClO_2$ (2.15 M in water, 273 µL, 587 µmol, 12.0 equiv) was added at r.t. and the mixture was stirred vigorously for 45 min. The light-yellow solution was cooled to 0 °C, and pH 2.5 buffer[14] (1 mL) was added. The mixture was diluted with EtOAc (2 mL), the phases were separated and the aqueous phase was extracted with EtOAc (5x 1 mL). The combined organic phases were dried over Na_2SO_4 and all volatiles were removed under reduced pressure. Purification *via* preparative thin-layer chromatography (plate size: 20x10 cm, eluent: EtOAc + 0.5% formic acid)[15] gave impure carboxylic acid **S27** (14.8 mg) that was used in the subsequent deprotection without detailed characterization.

Acetyl chloride (25.6 µL, 359 µmol, 7.3 equiv) was added to stirred MeOH (640 µL) at r.t. and the solution was stirred at this temperature for 25 min. The solution was then added to carboxylic acid **S27** (14.8 mg) and the mixture was stirred at r.t. for 4.5 h. The light-yellow solution was diluted with MeOH (1 mL) and all volatiles were removed under reduced pressure (rotavap bath temperature: 30 °C). Purification *via* preparative thin-layer chromatography (plate size: 10x10 cm, eluent: CH_2Cl_2:MeOH = 5:1 + 0.5% formic acid) afforded pure acid **S28** (5.9 mg, 19.8 µmol, 40%)[16] as a colorless residue.[17]

Analytical data are in accordance with the data reported by Rychnovsky and Burns.[12]

R_f (EtOAc + 0.5% formic acid, Hanessian's) = 0.34.

^{1}H-NMR (600 MHz, CD_3OD): δ(ppm) = 3.65 (d, J = 8.5 Hz, 1H), 3.48 (d, J = 8.5 Hz, 1H), 3.13–3.07 (m, 1H), 2.41 (dd, J = 12.9, 2.3 Hz, 1H), 2.08 (ddd, J = 14.5, 11.0, 3.5 Hz, 1H), 2.00 (ddd, J = 14.5, 8.9, 6.0 Hz, 1H), 1.78 (d, J = 12.9 Hz, 1H), 1.65–1.57 (m, 1H), 1.57–1.50 (m, 1H), 1.30 (s, 3H), 1.08 (s, 3H), 1.00 (s, 3H).

^{13}C-NMR (151 MHz, CD_3OD): δ(ppm) = 176.2, 111.8, 86.6, 77.0, 70.5, 66.2, 63.9, 54.1, 45.5, 45.1, 44.6, 24.6, 20.7, 19.4, 7.0.

HRMS(ESI): Calcd for $C_{15}H_{21}O_6$ [M-H]$^-$: 297.1338; found: 297.1333.

$[\alpha]_D^{23}$ = +4.5 (c = 0.1, MeOH).

(–)-Illisimonin A (S29)

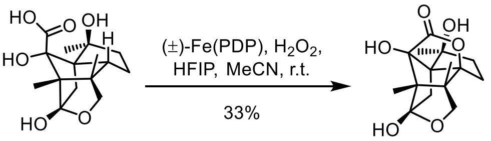

Illisimonin A (**S29**) was prepared after the protocol by Burns and Rychnovsky.[12]

Acid **S28** (4.9 mg, 16.4 µmol, 1.0 equiv) was coevaporated three times with HFIP:MeCN (3:1, 0.5 mL), before it was dissolved in HFIP (125 µL).

Racemic White's catalyst was prepared by mixing equal amounts of (+)-Fe(*R*,*R*)PDP and (–)-Fe(*S*,*S*)PDP. Two stock solutions were prepared:

---

[14] Prepared as described by Burns and Rychnovsky.[12]

[15] After preparative TLC, the product was eluted from the detached silica using EtOAc containing 0.5% of formic acid.

[16] After preparative TLC, the product was eluted from the detached silica using EtOAc containing 0.5% of formic acid. Slow elution of acid **S28** in this eluent made it necessary to repeat the washing procedure several times. For the scale described, we used a total of 30 mL of eluent for isolation of **S28**.

[17] For the scale described we deemed it inaccurate to specify the physical appearance (solid, oil, etc.) for the obtained product. The product was usually obtained as a film covering the walls of the flask it was collected in; therefore, we suggest the formulation "residue" as a compromise.

H_2O_2 (30 wt% in water, 20 µL) + MeCN (552 µL)
(±)-Fe(PDP) (16.9 mg) + MeCN (477 µL)

110 µL of both solutions were added simultaneously *via* syringe pump over 45 min to the stirred solution of **S28** in HFIP at r.t.. After complete addition, *i*PrOH (500 µL) was added and all volatiles were removed under reduced pressure. After purification by preparative thin-layer chromatography (plate size: 5x10 cm, eluent: EtOAc, developed 3x), illisimonin A (**S29**) (1.6 mg, 5.40 µmol, 33%) was obtained as a colorless residue.[17]
Analytical data are in accordance with the literature.[12]

R_f (EtOAc, Hanessian's) = 0.33.
^{1}H NMR (600 MHz, CD_3OD): δ(ppm) = 3.79 (d, *J* = 10.1 Hz, 1H), 3.55 (d, *J* = 10.1 Hz, 1H), 2.37 (d app. t, *J* = 14.2, 8.6 Hz, 1H), 2.31 (d, *J* = 14.3 Hz, 1H), 2.25 (ddd, *J* = 14.0, 10.6, 3.2 Hz, 1H), 2.02 (ddd, *J* = 14.3, 10.4, 8.5 Hz, 1H), 1.98 (d, *J* = 14.3 Hz, 1H), 1.91 (ddd, *J* = 14.3, 8.9, 3.1 Hz, 1H), 1.27 (s, 3H), 1.03 (s, 3H), 0.91 (s, 3H).
^{13}C-NMR (151 MHz, CD_3OD): δ(ppm) = 177.6, 111.5, 104.9, 88.4, 76.7, 71.0, 69.7, 63.9, 52.8, 46.5, 39.1, 28.1, 23.1, 16.7, 5.9.
HRMS(ESI): Calcd for $C_{15}H_{20}O_6Na$ $[M+Na]^+$: 319.1158; found: 319.1160.
$[\alpha]_D^{29}$ = –11.9 (*c* = 0.3, MeOH).

Alternative endgame

Lactol S30

Procedure A[18] — starting from tri-protected tetraol **S25**

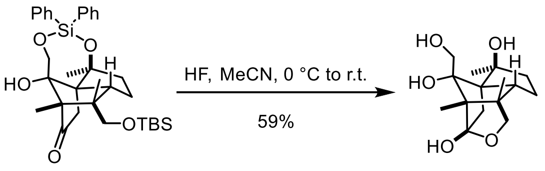

(±)-**S25** (±)-**S30**

HF (2.0 M in water, 760 µL, 1.52 mmol, 4.0 equiv) was added dropwise to a stirred solution of ketone (±)-**S25** (220 mg, 380 µmol, 1.0 equiv) in acetonitrile (7.2 mL) at 0 °C. The mixture was stirred at 0 °C for 2 h, then warmed to r.t. and stirred for 7 d. The reaction was quenched with aqueous $NaHCO_3$ (sat., 2.5 mL), the phases were separated and the aqueous phase was extracted with EtOAc (4x 10 mL). The combined organic phases were dried over Na_2SO_4 and all volatiles were removed under reduced pressure. Purification *via* column chromatography (EtOAc:PE = 1.5:1 to 2.5:1) gave lactol (±)-**S30** (63.3 mg, 223 µmol, 59%) as a white solid.

Procedure B — starting from mono-protected tetraol **S26**

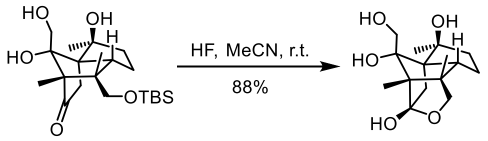

S26 **S30**

HF (2.0 M in water, 100 µL, 200 µmol, 4.0 equiv) was added in one portion to a stirred solution of ketone **S26** (20.0 mg, 50.2 µmol, 1.0 equiv) in acetonitrile (1.0 mL) at r.t.. After 5 d, the reaction was quenched by addition of aqueous $NaHCO_3$ (sat., 1 mL) and the mixture was diluted with EtOAc (5 mL). The phases were separated and the aqueous phase was extracted with EtOAc (5x 2 mL). The combined organic phases were dried over Na_2SO_4 and concentrated under reduced pressure. Purification *via* column chromatography (EtOAc) gave lactol **S30** (12.6 mg, 44.3 µmol, 88%) as a white solid.

Analytical data for **S30**:

R_f (EtOAc; vanillin) = 0.24 (dark-blue).
^{1}H-NMR (400 MHz, CD_3OD): δ(ppm) = 4.09 (d, J = 11.5 Hz, 1H), 3.62 (d, J = 8.4 Hz, 1H), 3.44 (d, J = 8.5 Hz, 1H), 3.35 (d, J = 11.5 Hz, 1H), 2.94–2.83 (m, 1H), 2.36 (dd, J = 12.7, 2.3 Hz, 1H), 2.11–1.93 (m, 2H), 1.72 (d, J = 12.7 Hz, 1H), 1.66–1.45 (m, 2H), 1.27 (s, 3H), 0.98 (s, 3H), 0.83 (s, 3H).
^{13}C-NMR (101 MHz, CD_3OD): δ(ppm) = 113.0, 85.5, 76.3, 70.5, 64.1, 63.8, 61.2, 54.4, 44.53, 44.47, 44.4, 24.7, 21.7, 19.4, 6.3.
HRMS(ESI): Calcd for $C_{15}H_{24}O_5Na$ $[M+Na]^+$: 307.1521; found: 307.1523.
m.p. (CH_2Cl_2) = 147–149 °C.
$[\alpha]_D^{25}$ = +6.1 (c = 0.6, MeOH).

Carboxylic acid S28 from lactol S30

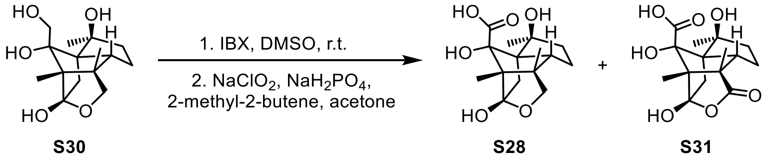

S30 **S28** **S31**

IBX (33.8 mg, 121 µmol, 2.9 equiv) was added to a solution of lactol **S30** (12.0 mg, 42.2 µmol, 1.0 equiv) in DMSO (420 µL) at r.t. After 19 h, water (1 mL) was added, the white residues were filtered off and washed with CH_2Cl_2 (2 mL). The phases were separated and the aqueous phase was extracted with CH_2Cl_2 (3x 1 mL). The combined organic phases were dried over Na_2SO_4 and all volatiles were removed under reduced pressure. The crude material was used in the next reaction without further purification.
The residue was dissolved in acetone (183 µL) and 2-methyl-2-butene (89.4 µL, 841 µmol, 19.9 equiv) and NaH_2PO_4 (2.0 M in water, 232 µL, 464 µmol, 11.0 equiv) were added under stirring at r.t.. After 5 min, $NaClO_2$ (2.0 M in water, 232 µL, 464 µmol, 11.0 equiv) was added and the mixture was stirred vigorously for 50 min. HCl (1.0 M in water, 2 mL) was added and the mixture diluted with EtOAc (2 mL). The phases were separated and the aqueous phase was extracted with EtOAc (5x 2 mL). The combined organic

[18] Procedure A was performed on racemic material.

phases were dried over Na_2SO_4 and concentrated under reduced pressure. After purification *via* preparative thin-layer chromatography (plate size: 10x10 cm, eluent: EtOAc + 0.5% formic acid), an impure mixture of desired carboxylic acid **S28** and overoxidized product **S31** (**S28**:**S31** = 1:1, 3.0 mg) was obtained as a colorless residue.[17] The mixture was used in the subsequent step without further purification.

Analytical data for **S31**:[19]
R_f (EtOAc, Hanessian's) = 0.33.
^{1}H-NMR (600 MHz, CD_3OD): δ(ppm) = 3.32–3.27 (m, 1H, HSQC), 2.55 (dd, *J* = 13.3, 1.7 Hz, 1H), 2.10–1.96 (m, 2H), 1.94 (d, *J* = 13.3 Hz, 1H), 1.77–1.71 (m, 1H), 1.30–1.26 (m, 1H, HSQC), 1.28 (s, 3H), 1.27 (s, 3H), 1.05 (s, 3H).
^{13}C-NMR (151 MHz, CD_3OD): δ(ppm) = 180.0, 176.9 (HMBC), 112.1, 86.9, 76.2, 70.3, 65.9, 52.6, 51.6, 45.1, 41.8, 24.3, 20.9, 17.6, 7.1.
HRMS(ESI): Calcd for $C_{15}H_{19}O_7$ $[M\text{-}H]^-$: 311.1131; found: 311.1140.

White-Chen oxidation of the mixture of S28 and S31

The reaction was performed after the protocol by Burns and Rychnovsky.[12]
The mixture of **S28** and **S31** was azeotroped three times with HFIP:MeCN (3:1, 0.5 mL) before it was dissolved in HFIP (85 µL).
Racemic White's catalyst was prepared by mixing equal amounts of (+)-Fe(*R,R*)PDP and (–)-Fe(*S*,*S*)PDP. Two stock solutions were prepared:

H_2O_2 (30 wt% in water, 20 µL) + MeCN (552 µL)
(±)-Fe(PDP) (16.9 mg) + MeCN (477 µL)

50.0 µL of both solutions were added simultaneously *via* syringe pump to the stirred solution of **S28** and **S31** in HFIP at r.t. over 45 min. After complete addition, *i*PrOH (100 µL) was added and all volatiles were removed under reduced pressure. After purification *via* preparative thin-layer chromatography (plate size: 5x10 cm, eluent: EtOAc, developed 2x), illisimonin A (**S29**) (0.8 mg, 2.7 µmol, 6% o3s) was obtained as a colorless residue.[17]

[19] The NMR data of **S31** was obtained from the impure mixture using 2D-NMR experiments for signal assignment.

NMR data of illisimonin A (S29)

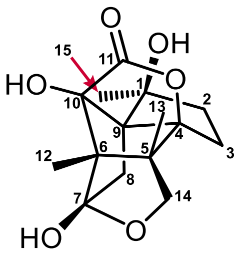

Table S1. Comparison of the obtained ^{1}H-NMR data with reported data.

Position	Isolation (Qu and co-workers[13]) ^{1}H NMR (500 MHz, CD_3OD)	Synthetic (Burns and Rychnovsky[12]) ^{1}H NMR (500 MHz, CD_3OD)	Synthetic (this work) ^{1}H NMR (600 MHz, CD_3OD)
2α	2.25 (ddd, *J* = 14.0, 10.5, 3.5 Hz, 1H)	2.25 (ddd, *J* = 14.0, 10.6, 3.3 Hz, 1H)	2.25 (ddd, *J* = 14.0, 10.6, 3.2 Hz, 1H)
2β	2.36 (ddd, *J* = 14.0, 8.5, 8.5 Hz, 1H)	2.37 (d app. t, *J* = 14.2, 8.7 Hz, 1H)	2.37 (d app. t, *J* = 14.2, 8.6 Hz, 1H)
3α	2.03 (ddd, *J* = 14.5, 10.5, 8.5 Hz, 1H)	2.02 (ddd, *J* = 14.3, 10.3, 8.5 Hz, 1H)	2.02 (ddd, *J* = 14.3, 10.4, 8.5 Hz, 1H)
3β	1.91 (ddd, *J* = 14.5, 8.5, 3.5 Hz, 1H)	1.91 (ddd, *J* = 14.3, 8.9, 3.1 Hz, 1H)	1.91 (ddd, *J* = 14.3, 8.9, 3.1 Hz, 1H)
8α	1.97 (d, *J* = 14.5 Hz, 1H)	1.97 (d, *J* = 14.3 Hz, 1H)	1.98 (d, *J* = 14.3 Hz, 1H)
8β	2.30 (d, *J* = 14.5 Hz, 1H)	2.31 (d, *J* = 14.3 Hz, 1H)	2.31 (d, *J* = 14.3 Hz, 1H)
12	0.91 (s, 3H)	0.91 (s, 3H)	0.91 (s, 3H)
13	1.03 (s, 3H)	1.03 (s, 3H)	1.03 (s, 3H)
14α	3.79 (d, *J* = 10.0 Hz, 1H)	3.79 (d, *J* = 10.1 Hz, 1H)	3.79 (d, *J* = 10.1 Hz, 1H)
14β	3.55 (d, *J* = 10.0 Hz, 1H)	3.55 (d, *J* = 10.1 Hz, 1H)	3.55 (d, *J* = 10.1 Hz, 1H)
15	1.27 (s, 3H)	1.27 (s, 3H)	1.27 (s, 3H)

Table S2. Comparison of the obtained ^{13}C-NMR data with reported data.

Position	Isolation (Qu and co-workers[13]) ^{13}C NMR (126 MHz, CD_3OD)	Synthetic (Burns and Rychnovsky[12]) ^{13}C NMR (151 MHz, CD_3OD)	Synthetic (this work) ^{13}C NMR (151 MHz, CD_3OD)
1	76.7	76.7	76.7
2	46.5	46.5	46.5
3	23.1	23.1	23.1
4	104.9	104.9	104.9
5	52.8	52.8	52.8
6	63.9	63.9	63.9
7	111.5	111.5	111.5
8	39.0	39.1	39.1
9	71.0	71.0	71.0
10	88.4	88.4	88.4
11	177.6	177.6	177.6
12	6.0	5.9	5.9
13	16.7	16.7	16.7
14	69.7	69.7	69.7
15	28.1	28.1	28.1

CD spectrum of (–)-illisimonin A

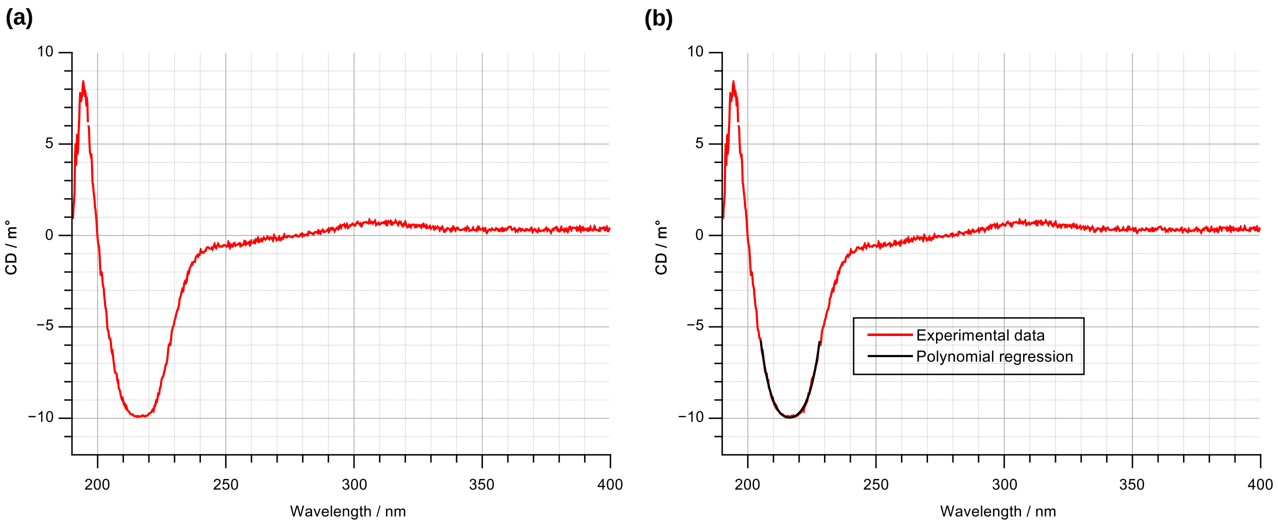

CD spectrum of synthetic (–)-illisimonin A. (a) Plain spectrum; (b) experimental data superimposed with polynomial function fit to the minimum region.

The minimum was determined by polynomial regression of the area between 205 nm and 228 nm. The following function was fit to the data (coefficients rounded to the second decimal):

$$y = 1.69\cdot10^{-3}+7.98\cdot10^{-2}\cdot x+5.27\cdot x^{2}-9.69\cdot10^{-2}\cdot x^{3}+6.68\cdot10^{-4}\cdot x^{4}-2.05\cdot10^{-6}\cdot x^{5}+2.36\cdot10^{-9}\cdot x^{6}$$

The local minimum in the area between 205 nm and 228 nm was determined to be 216.2 nm. This value is in accordance with the value of 216.5 nm reported by Qu and co-workers.[13]

4.2 Preparation of racemic Nazarov cyclization precursor (±)-S10

The synthetic sequence described in this publication was established using racemic material up to formal intermediate **S26**, before the asymmetric approach was developed. The test substrates and side products were only characterized as racemic substances during our initial scouting of the route. For the sake of completeness, we would therefore like to describe the preparation of *rac*-**S10**. The following procedure is an updated version of the procedure outlined in our earlier publication on the tandem-Nazarov/ene cyclization.[5] The updated procedure showed superior reproducibility in our hands.

(±)-2-Bromo-3,7-dimethylocta-1,6-dien-3-ol (S32)

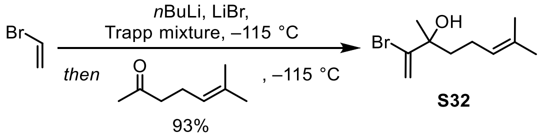

S32

A solution of vinyl bromide (23.5 mL, 345 mmol, 2.1 equiv) and LiBr (7.23 g, 83.3 mmol, 0.5 equiv)[20] in Trapp mixture (THF:Et_2O:*n*-pentane = 4:1:1, 160 mL) was placed inside a three-neck round-bottom flask, further referred to as "main flask", equipped with a low-temperature thermometer, an overhead stirrer with argon inlet and a rubber septum. A Schlenk flask, further referred to as "side flask", was filled with Trapp mixture (240 mL) and both flasks were cooled to –78 °C.[21] *n*BuLi (2.5 M in hexanes, 100 mL, 250 mmol, 1.5 equiv) was added to the solvent in the side flask and the solution was mixed by gently shaking the flask outside the cooling bath for approx. 30 s. Both solutions were cooled to −118 °C and the *n*BuLi solution was transferred to the main flask *via* transfer cannula over 2 h. After complete addition, the side flask was rinsed with Trapp mixture (2x 10 mL). During the entire addition and reaction the temperature of both main and side flask was maintained between −115 and −112 °C. Stirring in this temperature range was continued for 1 h during which time the formation of a white suspension occurred, indicating the precipitation of 1-bromo-1-lithioethene. Precipitation was accompanied by a swift rise of the main flask temperature which was countered by quick addition of liquid nitrogen to the coolant.
6-Methyl-5-hepten-2-one (25.0 mL, 168 mmol, 1.0 equiv) and Trapp mixture (80 mL) were placed in the side flask and the solution was cooled to −115 °C. The solution was then added to the reaction solution *via* transfer cannula over the course of 40 min, maintaining the temperature between −115 and −112 °C. After complete addition, stirring was continued in this temperature range for 1 h, before a solution of acetic acid (7.5 mL, 131 mmol, 0.8 equiv) in Trapp mixture (80 mL) was added at −115 °C over 20 min. The solution was allowed to warm to r.t. overnight, was washed with aqueous $NaHCO_3$ (5%, 250 mL) and the aqueous phase was extracted with Et_2O (3x 250 mL). The combined organic phases were washed with brine (150 mL), dried over $MgSO_4$ and concentrated under reduced pressure. The resulting bright yellow crude product was purified *via* vacuum distillation to afford bromo alcohol **S32** (36.6 g, 157 mmol, 93%) as a colorless oil.

b.p. (1 mbar) = 75 °C.
R_f (PE:EtOAc = 10:1; vanillin) = 0.46 (dark-blue).
^{1}H-NMR (400 MHz, $CDCl_3$): δ(ppm) = 5.93 (d, J = 2.0 Hz, 1H), 5.56 (d, J = 2.0 Hz, 1H), 5.18–5.11 (m, 1H), 2.12–1.92 (m, 3H), 1.91–1.83 (m, 1H), 1.71–1.64 (m, 4H), 1.62 (s, 3H), 1.44 (s, 3H).
^{13}C-NMR (101 MHz, $CDCl_3$): δ(ppm) = 140.8, 132.9, 123.9, 116.3, 77.1, 40.2, 27.1, 25.9, 22.9, 17.9.
HRMS(CI): Calcd for $C_{10}H_{18}OBr$ $[M+H]^+$: 233.0541; found: 233.0536.

---

[20] Prior to use, LiBr was melted over a Bunsen burner and poured directly into the main reaction flask where it was allowed to cool to r.t. under a constant stream of argon.

[21] *n*-Pentane/liquid nitrogen was used as cooling mixture. Controlled cooling was achieved by using a glass tube that was immersed in *n*-pentane and was filled with liquid nitrogen. This technique proved very successful for maintaining the reaction temperature over the course of the experiment. For initial cooling liquid nitrogen and *n*-pentane were mixed directly under stirring.

(±)-5-Hydroxy-2,5,9-trimethyl-4-methylenedeca-1,8-dien-3-one ((±)-S10)

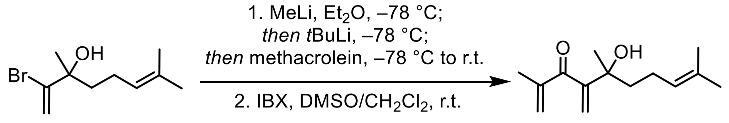

S32 (±)-**S10**

MeLi (1.6 M in Et_2O, 200 mL, 320 mmol, 1.0 equiv) was added at −78 °C to a stirred solution of bromo alcohol **S32** (74.5 g, 320 mmol, 1.0 equiv) in anhydrous Et_2O (1.3 L) over 50 min. After 40 min, a cooled solution of *t*BuLi (1.9 M in pentane, 340 mL, 646 mmol, 2.0 equiv) was added dropwise over 2 h. The yellow solution was stirred at −78 °C for 4 h, before methacrolein (34.5 mL, 409 mmol, 1.3 equiv) was added over 30 min. The resulting light yellow mixture was allowed to warm to r.t. overnight. MeOH (50 mL) was added, followed by aqueous potassium sodium tartrate (sat., 400 mL) and aqueous NH_4Cl (sat., 100 mL). The phases were separated and the aqueous phase was extracted with EtOAc (3x 400 mL). The combined organic phases were washed with brine (400 mL), dried over Na_2SO_4 and all volatiles were removed under reduced pressure. The obtained light-yellow oil (73.0 g) was used in the subsequent step without further purification.

The crude diol (±)-**S9** was dissolved in CH_2Cl_2/DMSO (1:1, 1.3 L) and IBX (107 g, 382 mmol, 1.2 equiv) was added at r.t.. Stirring was continued for 75 min and water (1 L) was added. The mixture was cooled with ice bath and stirred for 30 min. The phases were separated, the aqueous phase was extracted with CH_2Cl_2 (5x 500 mL) and the combined organic phases were washed with water (2x1 L) and brine (1 L). After drying over Na_2SO_4, the solvent was removed under reduced pressure and the oily residue was purified by column chromatography (PE:EtOAc = 50:1 to 25:1) to give cross-conjugated ketone (±)-**S10** (58.3 g, 262 mmol, 82%) as a yellow oil.

Analytical data can be found under the procedure for (*S*)-**S10**.

4.3 Reaction optimization

The following experimental results were obtained during the scouting of the synthetic route with racemic intermediates. Therefore, no optical rotations are given for any side product described herein.

Optimization for nucleophilic opening of oxasilolane S15

General procedure A Oxasilolane (±)-**S15** (1.0 equiv) was dissolved under argon atmosphere in the stated solvent (0.1 M) and the solution was cooled to the stated temperature. The nucleophile (type and equivalents stated in **table S3**) was added dropwise to the stirred solution. After reaction control by TLC indicated full conversion of starting material, the reaction was quenched by careful addition of aqueous NH_4Cl (sat.) and the mixture was allowed to warm to r.t.. The phases were separated and the aqueous phase was extracted with MTBE (3x). The combined organic phases were washed with brine, dried over Na_2SO_4 and concentrated under reduced pressure. The products were isolated by column chromatography (PE:EtOAc = 50:1 to 25:1).

General procedure B A 25 mL two neck flask, equipped with a stir bar, an internal thermometer and a "fake-Schlenk head",[22] was charged with oxasilolane (±)-**S15** (500 mg, 1.09 mmol, 1.0 equiv) and anhydrous MTBE (10 mL). The colorless solution was brought to the indicated starting temperature and MeMgCl (3.0 M in THF, 1.1 mL, 3.30 mmol, 3.0 equiv) was added quickly. The addition was accompanied by a swift gas evolution, as well as the formation of a turbid reaction mixture. The internal temperature was monitored after addition; the observed temperature range is given in **table S3**.[23] After reaction control by TLC indicated full conversion of starting material, aqueous NH_4Cl (sat., 5 mL) was added carefully until no further gas evolution was observed. The mixture was allowed to warm to r.t., the phases were separated and the aqueous phase was extracted with MTBE (3x 10 mL). The combined organic phases were concentrated under reduced pressure and the reaction products isolated by column chromatography (PE:EtOAc = 25:1 to 11:1).

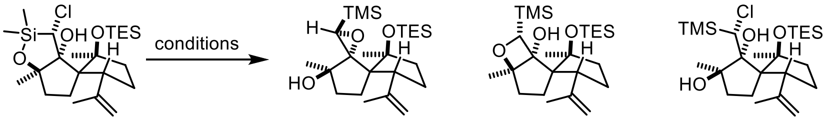

(±)-**S15** (±)-**S16** (±)-**S33** (±)-**S34**

Table S3. Reaction optimization for the nucleophilic opening of oxasilolane (±)-**S15**.

Entry	Conditions	Procedure	Result
	Initial reagent and solvent screening[a]		
1	MeLi (2.2 equiv), THF, −78 °C	A	(±)-**S33** (68%)
2	MeLi (2.2 equiv), THF, −78 °C; *then* 12-crown-4 (2.2 equiv), −78 °C to r.t.	A	(±)-**S33** (71%)
3	MeLi (2.2 equiv), Et_2O, −78 °C to r.t.	A	(±)-**S16** + (±)-**S34** (mixture)[b]
4	MeMgBr (3.0 equiv), THF, 0 °C	A	(±)-**S33** (main)[b]
5	MeMgBr (3.0 equiv), Et_2O, 0 °C	A	(±)-**S16** (50%)
6	MeMgBr (3.0 equiv), 1,4-dioxane, 0 °C to r.t.	A	complex mixture
7	MeMgBr (3.0 equiv), toluene, 0 °C	A	complex mixture
8	MeMgBr (3.0 equiv), CPME, 0 °C	A	(±)-**S16** (68%)
9	MeMgBr (3.0 equiv), CPME, r.t.	A	(±)-**S16** (65%)
10	MeMgBr (3.0 equiv), MTBE, 0 °C	A	(±)-**S16** (62%)
11	MeMgBr (3.0 equiv), CPME, −30 °C	A	(±)-**S34** (69%)
12	MeMgBr (3.0 equiv), MTBE, −30 °C	A	(±)-**S34** (84%)
	Refinement and scale-up[c]		
13	MeMgBr (3.0 equiv), CPME, 0 °C	A	(±)-**S16** (61%)[d]
14	MeMgBr (2.1 equiv), CPME, 0 °C	A	(±)-**S16** (55%)
15	MeMgBr (5.0 equiv), CPME, 0 °C	A	(±)-**S16** (56%)
16	MeMgBr (3.0 equiv), CPME, −30 °C to r.t.	A	(±)-**S16** (49%)
17	MeMgCl (6.0 equiv), CPME, 0 °C to r.t.	A	(±)-**S16** (47%), (±)-**S33** (53%)

[22] Y-shaped two-neck adapter; the joint of the straight neck is equipped with a rubber septum and the bend neck is equipped with an inert gas inlet.

[23] After reaching the high point, the temperature dropped again.

18	MeMgCl (6.0 equiv), MTBE, 0 °C to r.t.	A	(±)-**S16** (54%), (±)-**S33** (38%)
19	MeMgI (5.0 equiv), MTBE, 0 °C	A	(±)-**S16** (33%)
20	MeMgCl (3.0 equiv), MTBE, r.t.	B[e]	(±)-**S16** (63–71%)[f]
21	MeMgCl (3.0 equiv), MTBE/*n*-pentane (1:1), r.t.	B[e]	(±)-**S16** (51%)
22	MeMgCl (3.0 equiv), MTBE/*n*-pentane (1:1), r.t.	B[e]	(±)-**S16** (52%)
	Optimization of general procedure B[g]		
23	MeMgCl (3.0 equiv), MTBE, 56 °C	B	(±)-**S16** (52%)
24	MeMgCl (3.0 equiv), MTBE, 33 °C to 39 °C	B	(±)-**S16** (68%), (±)-**S33** (31%)
25	MeMgCl (3.0 equiv), MTBE, 24 °C to 32 °C	B	(±)-**S16** (73%), (±)-**S33** (26%)
26	MeMgCl (3.0 equiv), MTBE, 10 °C to 16 °C	B	(±)-**S16** (75%), (±)-**S33** (25%)
27	MeMgCl (3.0 equiv), MTBE, 2 °C to 8 °C	B	(±)-**S16** (76%), (±)-**S33** (24%)
28	MeMgCl (3.0 equiv), MTBE, −12 °C to −1 °C	B	(±)-**S16** (78%)[h]
29	MeMgCl (3.0 equiv), MTBE, −21 °C to −17 °C	B	(±)-**S16** (75%)[h]

[a] 10 mg scale; [b] Product identification by ^{1}H-NMR of the crude product mixture; [c] 50–100 mg scale if not noted otherwise; [d] These conditions failed to give reliable results upon scale-up; [e] Experiments performed in a regular Schlenk tube without measurement of internal temperature; [f] For scales ranging from 50 mg to 6.0 g. No clear scale-yield correlation observed; [g] 500 mg scale; [h] Side product (±)-**S33** only isolated as mixture with unidentified side products.

Analytical data of opening products (±)-S33 and (±)-S34

Oxetane (±)-**S33**:

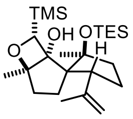

(±)-**S33**

Appearance: colorless oil.
R$_f$ (PE:EtOAc = 25:1; vanillin) = 0.34 (dark purple).
^{1}H-NMR (400 MHz, CDCl$_3$): δ(ppm) = 4.87–4.81 (m, 1H, H-12a), 4.77–4.73 (m, 1H, H-12b), 4.70 (d, J = 1.4 Hz, 1H, H-1), 4.07 (d, J = 1.4 Hz, 1H, OH), 2.84 (dd, J = 11.2, 8.4 Hz, 1H, H-10), 2.14–1.97 (m, 2H, H-{5a,8a}), 1.93–1.83 (m, 1H, H-5b), 1.78–1.61 (m, 4H, H-{4a,4b,8b,9a}), 1.74–1.71 (m, 3H, CH_3-15), 1.50–1.38 (m, 1H, H-9b), 1.35 (s, 3H, CH_3-14), 1.32 (s, 3H, CH_3-13), 0.97 (t, J = 7.9 Hz, 9H, OTES), 0.67 (q, J = 7.9 Hz, 6H, OTES), 0.16 (s, 9H, TMS).
^{13}C-NMR (101 MHz, CDCl$_3$): δ(ppm) = 148.0 (C-11), 115.1 (C-12), 93.4 (C-3), 90.6 (C-2), 87.0 (C-7), 80.4 (C-1), 60.3 (C-6), 50.7 (C-10), 42.3 (C-8), 37.9 (C-4), 28.7 (C-5), 28.1 (C-9), 26.3 (C-14), 20.6 (C-13), 20.4 (C-15), 7.2 (OTES), 6.8 (OTES), −2.1 (TMS).
HRMS(ESI): Calcd for $C_{24}H_{46}O_3Si_2Na$ $[M+Na]^+$: 461.2883; found: 461.2880.

The relative configuration of **S33** was proposed based on the following NOE correlations:

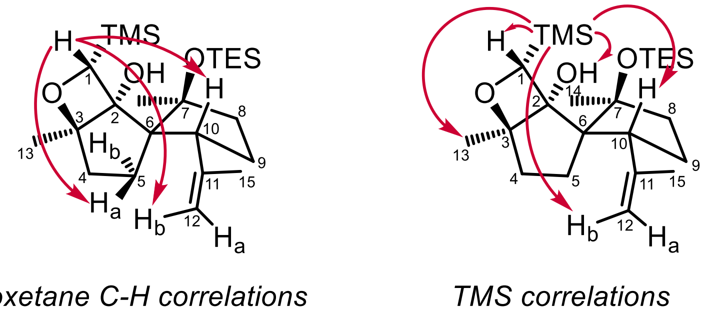

Figure S9. Signal assignment and relevant NOE correlations for the proposed relative stereoconfiguration of oxetane **S33**.

Chlorohydrine (±)-**S34**:

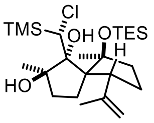

(±)-**S34**

Appearance: colorless oil.
$\mathbf{R_f}$ (PE:EtOAc = 25:1; vanillin) = 0.38 (purple).
^{1}H-NMR (400 MHz, $CDCl_3$): δ(ppm) = 5.19–5.17 (m, 1H), 5.10 (s, 1H), 4.81–4.78 (m, 1H), 4.14 (s, 1H), 3.98 (dd, J = 11.4, 8.3 Hz, 1H), 2.31 (dt, J = 13.7, 6.6 Hz, 1H), 2.01–1.91 (m, 4H), 1.89–1.79 (m, 2H), 1.75–1.64 (m, 1H), 1.63–1.56 (m, 1H), 1.53–1.46 (m, 1H), 1.49 (s, 3H), 1.42 (s, 3H), 1.28–1.22 (m, 1H), 1.09 (s, 1H, OH), 0.99 (t, J = 7.9 Hz, 9H), 0.76–0.67 (m, 6H), 0.25 (s, 9H).
^{13}C-NMR (101 MHz, $CDCl_3$): δ(ppm) = 149.4, 113.5, 90.1, 89.7, 84.7, 69.5, 60.7, 49.1, 41.0, 39.1, 28.31, 28.26, 26.7, 24.2, 22.4, 7.3, 6.7, 1.4.
HRMS(ESI): Calcd for $C_{24}H_{47}ClO_3Si_2Na$ $[M+Na]^+$: 497.2650; found: 497.2649.

Condition screening for the allylic oxidation of diene S19

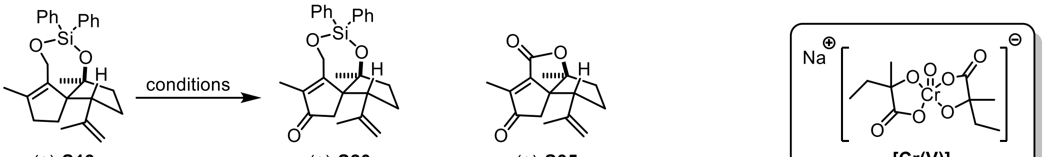

(±)-**S19** (±)-**S20** (±)-**S35** **[Cr(V)]**

Table S4. Condition screening for the allylic oxidation of diene (±)-**S19**.

Entry	Conditions	Result
1	$Rh_2(cap)_4(MeCN)_2$ (5 mol%), *t*BuOOH (70% in water), DCE, 45 °C, 12 h	(±)-**S20** (12%)
2	$Rh_2(cap)_4(MeCN)_2$ (5 mol%), *t*BuOOH (5.5 M in decane), DCE, r.t., 33 h	(±)-**S20** (25%)
3	$Rh_2(esp)_2$ (5 mol%), *t*BuOOH (70% in water), DCE, r.t., 6 h	(±)-**S20** (40%)
4	$Rh_2(esp)_2$ (0.5 mol%), NHPI (10 mol%), EtOAc, air, r.t., 19 h	(±)-**S20** (17%, 25% brsm)
5	$Mn(OAc)_3{\cdot}2\ H_2O$ (10 mol%), *t*BuOOH (5.5 M in decane), 3 Å MS, EtOAc, r.t., 24 h	(±)-**S20** (19%)
6	CuI, *t*BuOOH (5.5 M in decane), MeCN, r.t., 33 h	(±)-**S20** (16%)
7	$Co(OAc)_2{\cdot}4\ H_2O$ (1 mol%), NHPI (10 mol%), *t*BuOOH (5.5 M in decane), acetone, r.t., 19 h	(±)-**S20** (31%)
8	CrO_3 (12.0 equiv), 3,5-dimethylpyrazole (12.0 equiv), CH_2Cl_2, –20 to –10 °C	(±)-**S20** (20%, 26% brsm)
9	CrO_3 (12.0 equiv), 3,5-dimethylpyrazole (12.0 equiv), CH_2Cl_2, –20 to –15 °C	(±)-**S20** (20%, 26% brsm)
10	Cr(V)-complex **[Cr(V)]** (5.0 equiv), 15-crown-5 (7.0 equiv), MnO_2 (25.0 equiv), trifluorotoluene, 80 °C, 36 h	(±)-**S20** (31%, 39% brsm), (±)-**S35** (6%)
11	Cr(V)-complex **[Cr(V)]** (5.0 equiv), 15-crown-5 (7.0 equiv), MnO_2 (10.0 equiv), DCE, 80 °C, 48 h	(±)-**S20** (36%, 47% brsm)
12	Cr(V)-complex **[Cr(V)]** (5.0 equiv), 15-crown-5 (7.0 equiv), MnO_2 (25.0 equiv), trifluorotoluene, 100 °C, 48 h	(±)-**S20** (5%)
13	Cr(V)-complex **[Cr(V)]** (5.0 equiv), 15-crown-5 (7.0 equiv), trifluorotoluene, 80 °C, 19 h	(±)-**S20** (21%, 45% brsm)
14	Cr(V)-complex **[Cr(V)]**[a],15-crown-5 (7.0 equiv), trifluorotoluene, 80 °C, 19 h	(±)-**S20** (18%, 43% brsm)
15	Cr(V)-complex **[Cr(V)]** (2.0 equiv), 15-crown-5 (3.0 equiv), trifluorotoluene, 80 °C, 19 h	(±)-**S20** (22%, 63% brsm); 50% in 4 cycles[b]

[a] Successive addition of 1.0 equiv per hour; [b] Starting material was recovered by column chromatography and reused for the oxidation.

Analytical data for lactone (±)-S35

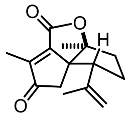

(±)-**S35**

Appearance: colorless oil.
R_f (PE:EtOAc = 5:1; vanillin) = 0.42 (dark-green).
^{1}H-NMR (400 MHz, $CDCl_3$): δ(ppm) = 4.98–4.94 (m, 1H), 4.75–4.70 (m, 1H), 2.85 (dd, J = 11.8, 5.8 Hz, 1H), 2.59 (d, J = 18.0 Hz, 1H), 2.33 (ddd, J = 14.9, 10.1, 8.2 Hz, 1H), 2.22 (d, J = 18.0 Hz, 1H), 2.07–1.96 (m, 2H), 2.04 (s, 3H), 1.89–1.77 (m, 1H), 1.58 (s, 3H), 1.33 (s, 3H).
^{13}C-NMR (101 MHz, $CDCl_3$): δ(ppm) = 208.4, 164.8, 160.5, 143.9, 142.7, 114.7, 98.5, 60.9, 53.4, 40.6, 36.8, 30.0, 23.4, 22.5, 9.1.
HRMS(ESI): Calcd for $C_{15}H_{18}O_3Na$ $[M+Na]^+$ = 269.1154; found: 269.1159.

Condition screening for the Ti(III)-mediated reductive cyclization of S21

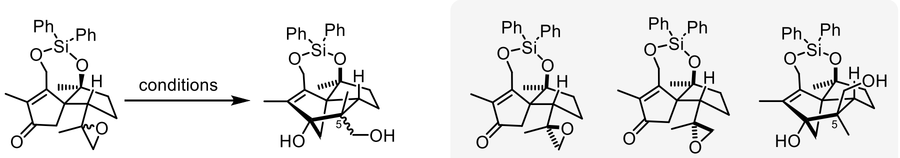

(±)-**S21** (±)-**S22** (±)-**S21a** (±)-**S21b** (±)-5-*epi*-**S22**

Table S5. Reaction optimization for the Ti(III)-mediated cyclization of (±)-**S21**.

Entry	Conditions[a]	Result[c]
1	(±)-**S21a**, Cp_2TiCl_2 (3.0 equiv), Zn (9.0 equiv), 3 h; addition of epoxide solution to Ti(III) solution	(±)-**S22** (56%), d.r. 1:1.6
2	(±)-**S21b**,Cp_2TiCl_2 (3.0 equiv), Zn (9.0 equiv), 3 h; addition of epoxide solution to Ti(III) solution	(±)-**S22** (50%), d.r. 1:1.5
3	(±)-**S21a** + (±)-**S21b** (2.1:1)[b], Cp_2TiCl_2 (3.0 equiv), Zn (9.0 equiv), 24 h; addition of Ti(III) solution to epoxide solution *via* syringe pump[d]	Recovery of starting material (49%)
4	(±)-**S21a** + (±)-**S21b** (2.1:1)[b], Cp_2TiCl_2 (3.0 equiv), Zn (9.0 equiv), 5 h; addition of epoxide solution to Ti(III) solution *via*syringe pump[e]	(±)-**S22** (63%), d.r. 1:1.7
5	(±)-**S21a** + (±)-**S21b** (2.1:1)[b], Cp_2TiCl_2 (0.1 equiv), Zn (3.0 equiv), collidine·HCl (2.0 equiv), 24 h; addition of epoxide solution to solution of Ti(III)-complex and collidine·HCl *via* syringe pump[e]	No reaction
6	(±)-**S21a** + (±)-**S21b** (2.1:1)[b], Cp_2TiCl_2 (3.0 equiv), Zn (9.0 equiv), 1 h; addition of epoxide solution to Ti(III) solution over 30 min	(±)-**S22** (89%), d.r. 1:1.6[f]

[a] Reactions performed at r.t. with THF as solvent; [b] Diastereomeric epoxides not separated prior to cyclization; [c] The main isomer was found to be the undesired (±)-5-*epi*-**S22** (see NOE correlations); [d] Addition time: 2 h; [e] Addition time: 1.5 h; [f] Yield increased upon scale-up.

Condition screening for the semipinacol rearrangement of S24

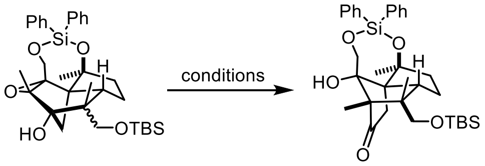

(±)-**S24** (±)-**S25**

Table S6. Reaction optimization for the semipinacol rearrangement of (±)-**S24**.

Entry	Conditions	Result
1	TFA (0.9 equiv), $CHCl_3$, r.t.	No reaction
2	TFA (0.9 equiv), HFIP, r.t.	Decomposition
3	TMSOTf (3.6 equiv), *i*Pr_2NEt (3.6 equiv), CH_2Cl_2, −78 °C[14]	Inconclusive NMR data
4	$AlMe_3$ (2 M in heptane, 2.0 equiv), CH_2Cl_2, −78 °C to 0 °C[15]	No reaction
5	BF_3·OEt_2 (2.0 equiv), CH_2Cl_2, −78 °C[16]	(±)-**S25** (<36%)
6	$SnCl_4$ (2.0 equiv), CH_2Cl_2, −78 °C[17]	(±)-**S25** (36%)

4.4 Studies on the synthesis of tricyclo[5.2.1.0^{1,5}]decanes from spirocyclic precursors *via* radical cyclizations

For the following cyclization studies racemic substances were used.

MHAT-initiated cyclization of enone S36

Enone S36

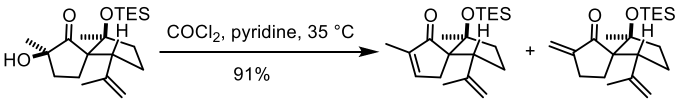

α-Hydroxy ketone (±)-**S12** (1.96 g, 5.56 mmol, 1.0 equiv) was dissolved in anhydrous pyridine (110 mL) and the solution was heated to 35 °C. Phosgene (15 wt% in toluene, 40.0 mL, 56.0 mmol, 10.1 equiv) was added quickly *via* dropping funnel. After 16 h, the brown heterogeneous mixture was cooled to 0 °C and water (50 mL) was added carefully. The mixture was diluted with MTBE (100 mL), the phases were separated and the aqueous phase was extracted with MTBE (3x 50 mL). The combined organic phases were washed with brine (100 mL), dried over Na_2SO_4 and concentrated under reduced pressure (60 °C water bath temperature to remove residual pyridine). Purification *via* column chromatography (PE:EtOAc = 100:1) gave a mixture of desired enone *endo*-**S36** and its double bond isomer *exo*-**S36** (*endo*:*exo* = 5:1, 1.69 g, 5.05 mmol, 91%) as a light yellow oil.
An analytically pure sample of *endo*-**S36** was obtained by preparative TLC (50.0 mg sample, plate size: 20x10 cm, eluent: PE:EtOAc = 200:1, developed 5x).

R_f (PE:EtOAc = 25:1; vanillin) = 0.66 (dark-purple).
^{1}H-NMR (400 MHz, $CDCl_3$): δ(ppm) = 7.15–7.12 (m, 1H), 4.80–4.77 (m, 1H), 4.67–4.64 (m, 1H), 3.49 (t, *J* = 8.0 Hz, 1H), 2.34 (dquint, *J* = 18.9, 2.5 Hz, 1H), 2.17–2.09 (m, 1H), 2.00–1.89 (m, 2H), 1.77–1.66 (m, 5H), 1.49 (s, 3H), 1.14 (s, 3H), 0.94 (t, *J* = 7.9 Hz, 9H), 0.60–0.53 (6H, m).
^{13}C-NMR (101 MHz, $CDCl_3$): δ(ppm) = 208.9, 153.8, 146.8, 142.3, 111.2, 86.3, 63.6, 49.2, 39.7, 33.6, 25.5, 23.9, 23.7, 10.7, 7.2, 6.8.
HRMS(ESI): Calcd for $C_{20}H_{34}O_2SiNa$ $[M+Na]^+$: 357.2226; found: 357.2225.

Fe(acac)$_3$-mediated cyclization of enone S36

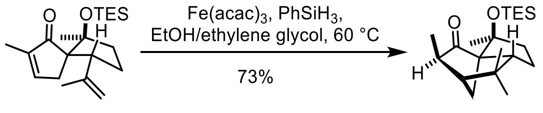

Tricycle **S37** was synthesized according to the protocol by Baran and co-workers.[18]
$PhSiH_3$ (1.5 mL, 12.2 mmol, 2.5 equiv) was added at r.t. to a stirred solution of enone **S36** (*endo*:*exo* = 5:1, 1.65 g, 4.93 mmol, 1.0 equiv) and $Fe(acac)_3$ (434 mg, 1.23 mmol, 0.25 equiv) in EtOH/ethylene glycol (5:1, 96 mL) and the reaction mixture was heated to 60 °C.[24] After 4 h, the mixture was allowed to cool to r.t. and was diluted with brine (100 mL). The phases were separated, the aqueous phase was extracted with MTBE (5x 100 mL) and the combined organic phases were dried over Na_2SO_4 and all volatiles were removed under reduced pressure. Purification *via* column chromatography (PE:EtOAc = 200:1 to 100:1) gave tricycle **S37** (1.22 g, 3.62 mmol, 73%) as a light yellow oil.

R_f (PE:EtOAc = 25:1; vanillin) = 0.62 (red).
^{1}H-NMR (400 MHz, $CDCl_3$): δ(ppm) = 2.27 (ddq, *J* = 7.4, 4.2, 1.2 Hz, 1H, H-8), 2.15 (ddd, *J* = 10.8, 4.6, 2.0 Hz, 1H, H-5), 2.04–2.00 (m, 1H, H-7), 1.94–1.81 (m, 1H, H-4a), 1.77–1.71 (m, 3H, H-{3,10b}), 1.63–1.55 (m, 1H, H-4b), 1.37–1.32 (m, 1H, H-10a), 1.31 (s, 3H, H-11), 1.23 (d, *J* = 7.4 Hz, 3H, CH_3-14), 1.14 (s, 3H, CH_3-13), 1.07 (s, 3H, CH_3-12), 0.97 (t, *J* = 7.9 Hz, 9H, OTES), 0.72–0.53 (m, 6H, OTES).
^{13}C-NMR (101 MHz, $CDCl_3$): δ(ppm) = 216.0 (C-9), 81.1 (C-2), 74.4 (C-1), 55.0 (C-5), 52.8 (C-8), 51.5 (C-7), 42.39, 42.38 (C-{3,6}), 40.8 (C-10), 29.6 (C-13), 29.1 (C-12), 23.7 (C-11), 22.5 (C-4), 13.6 (C-14), 7.3 (OTES), 6.9 (OTES).
HRMS(ESI): Calcd for $C_{20}H_{36}O_2SiNa$ $[M+Na]^+$: 359.2382; found: 359.2383.

---

[24] The reaction flask should be only loosely closed to allow evolving hydrogen gas to escape.

The relative configuration of **S37** was proposed based on the following NOE correlations

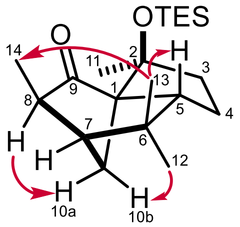

Figure S10. Signal assignment and relevant NOE correlations for the proposed relative stereoconfiguration of tricycle **S37**.

MHAT-initiated cyclization with OTBS-functionalized side chain

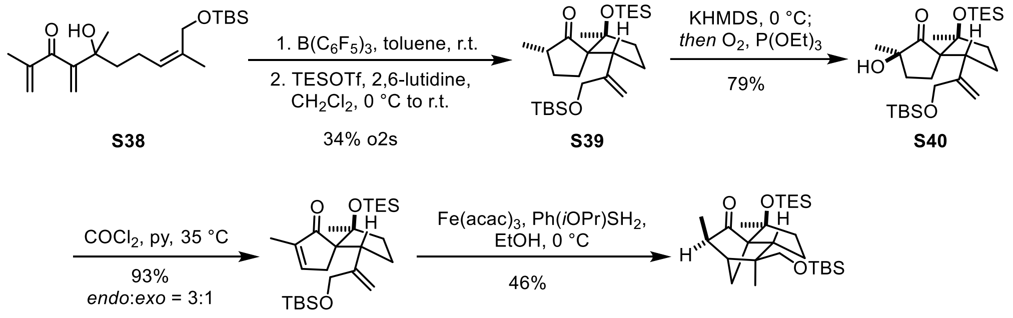

Spiroketone S39

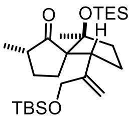

S39

$B(C_6F_5)_3$ (150 mg, 293 µmol, 2.5 mol%) was added in one portion to a stirred solution of cross-conjugated ketone (±)-**S38** (4.13 g, 11.7 mmol, 1.0 equiv) in toluene (690 mL) at r.t.. After 3 d, the purple reaction mixture was evaporated and the crude material was purified *via* column chromatography (PE:EtOAc = 99:1 to 19:1) to separate the unprotected spiro ketone from the main impurities. The so obtained material was used in the subsequent TES-protection without further characterization.

2,6-lutidine (940 µL, 8.10 mmol, 1.5 equiv) and TESOTf (1.3 mL, 5.75 mmol, 1.1 equiv) were added successively to a solution of the obtained spiro ketone (1.91 g, 5.42 mmol, 1.0 equiv) in anhydrous CH_2Cl_2 (50 mL) at 0 °C. The cooling bath was removed and stirring was continued at r.t. for 60 min. Water (25 mL) was added, the phases were separated and the aqueous phase was extracted with CH_2Cl_2 (3x 25 mL). The combined organic phases were dried over Na_2SO_4 and concentrated under reduced pressure. Purification *via* column chromatography (PE:EtOAc = 49:1) gave TES-protected spiroketone **S39** (1.84 g, 3.94 mmol, 34% o2s) as a light yellow oil.

$\mathbf{R_f}$ (PE:EtOAc = 4:1; vanillin) = 0.71 (purple).
^{1}H-NMR (400 MHz, $CDCl_3$) δ(ppm) = 5.25 (d, *J* = 1.8 Hz, 1H), 4.81 (s, 1H), 3.86 (d, *J* = 15.1 Hz, 1H), 3.75 (d, *J* = 15.0 Hz, 1H), 3.03 (dd, *J* = 11.6, 7.8 Hz, 1H), 2.08–1.80 (m, 5H), 1.68–1.61 (m, 2H), 1.54–1.45 (m, 2H), 1.30 (s, 3H), 1.11 (d, *J* = 6.9 Hz, 3H), 0.92 (t, *J* = 8.0 Hz, 9H), 0.89 (s, 9H), 0.55 (q, *J* = 7.9 Hz, 6H), 0.03 (s, 3H), 0.02 (s, 3H).
^{13}C-NMR (101 MHz, $CDCl_3$) δ(ppm) = 222.7, 149.2, 110.2, 86.4, 66.4, 63.8, 46.5, 44.3, 40.4, 28.90, 28.88, 28.8, 26.3, 26.1, 18.6, 14.1, 7.1, 6.6, –5.3.
HRMS(ESI): Calcd for $C_{26}H_{50}O_3Si_2Na$ $[M+Na]^+$: 489.3196; found: 489.3192.

α-Hydroxy ketone S40

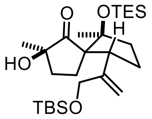

S40

A solution of **S39** (500 mg, 1.07 mmol, 1.0 equiv) in anhydrous THF (0.5 mL) was cooled to 0 °C and KHMDS (1.0 M in THF, 2.1 mL, 2.10 mmol, 2.0 equiv) was added dropwise. The yellow solution was stirred at 0 °C for 1 h. $P(OEt)_3$ (350 µL, 2.02 mmol, 1.9 equiv) was added and pressured air was bubbled through the solution. Upon exposure to oxygen, the solution gradually got darker, with a dark orange solution usually indicating completion of the reaction. After exposure to the air stream for 10 min, aqueous NH_4Cl (sat., 2 mL) was added and the mixture was diluted with MTBE (4 mL). The phases were separated, the aqueous phase extracted with MTBE (3x 5 mL) and the combined organic phases were washed with brine (5 mL), dried over Na_2SO_4 and concentrated under reduced pressure. Purification *via* column chromatography (PE:EtOAc = 11:1) gave α-hydroxy ketone **S40** (412 mg, 0.85 mmol, 79%) as a light yellow oil.

$\mathbf{R_f}$ (PE:EtOAc = 2.5:1; vanillin) = 0.69 (blue).
^{1}H-NMR (400MHz, $CDCl_3$): δ(ppm) = 5.32 (q, J = 1.8 Hz, 1H), 4.87 (s, 1H), 3.89–3.87 (m, 2H), 2.97 (dd, J = 11.0, 8.1 Hz, 1H), 2.12 (td, J = 11.7, 7.0 Hz, 1H), 1.93–1.86 (m, 2H), 1.82–1.79 (m, 2H), 1.75–1.69 (m, 2H), 1.64–1.58 (m, 1H), 1.284 (s, 3H), 1.278 (s, 3H), 0.91 (t, J = 6.0 Hz, 9H), 0.89 (s, 9H), 0.54 (q, J = 7.9 Hz, 6H), 0.04 (s, 3H), 0.03 (s, 3H).
^{13}C-NMR (101 MHz, $CDCl_3$): δ(ppm) = 221.6, 148.9, 110.7, 86.0, 76.8, 66.3, 63.4, 46.9, 40.0, 34.7, 29.0, 26.2, 26.1, 25.4, 22.7, 18.5, 7.1, 6.5, –5.24, –5.26.
HRMS(ESI): Calcd for $C_{26}H_{50}O_4SiNa$ $[M+Na]^+$: 505.3145; found: 505.3135.

Enone S41

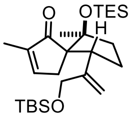

S41

α-Hydroxy ketone **S40** (76.7 mg, 159 µmol, 1.0 equiv) was dissolved in anhydrous pyridine (3.2 mL) and heated to 35 °C. After 10 min, phosgene (15 wt% in toluene, 170 µL, 238 µmol, 1.5 equiv) was added quickly. After 29 h, the mixture was cooled to 0 °C, and water (5 mL) was added under vigorous stirring. The phases were separated and the aqueous phase was extracted with MTBE (3x 10 mL). The combined organic phases were washed with brine (10 mL), dried over Na_2SO_4 and concentrated under reduced pressure. Purification *via* column chromatography (PE:EtOAc = 11:1) gave enone **S41** (*endo*:*exo* = 3:1, 68.7 mg, 148 mol, 93%) as a colorless oil.

Analytical data (main isomer):

$\mathbf{R_f}$ (PE:EtOAc = 4:1; vanillin) = 0.77 (blue).
^{1}H-NMR (400 MHz, $CDCl_3$): δ(ppm) = 7.14 (bs, 1H), 5.27–5.26 (m, 1H), 4.86–4.84 (m, 1H), 3.83 (d, J = 15.4 Hz, 1H), 3.59 (d, J = 15.6 Hz, 1H), 3.41 (t, J = 9.0 Hz, 1H), 2.39 (dt, J = 18.9, 2.5 Hz, 1H), 2.12 (d, J = 18.9 Hz, 1H), 2.01–1.85 (m, 2H), 1.85–1.77 (m, 1H), 1.74–1.73 (m, 4H), 1.15 (s, 3H), 0.93 (t, J = 7.9 Hz, 9H), 0.86 (s, 9H), 0.56 (td, J = 7.9, 1.7 Hz, 6H), –0.018 (s, 3H), –0.022 (s, 3H).
^{13}C-NMR (101 MHz, $CDCl_3$): δ(ppm) = 208.7, 154.0, 149.5, 142.3, 108.4, 86.5, 65.9, 63.8, 45.0, 39.6, 33.6, 26.1, 25.6, 23.7, 18.5, 10.7, 7.2, 6.8, –5.34, –5.38.
HRMS(ESI): Calcd for $C_{26}H_{48}O_3Si_2Na$ [M+Na]+: 487.3040; found: 487.3046.

Tricycle S42

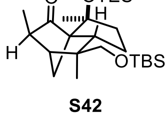

S42

Cyclization of enone **S41** was performed according to a procedures by Pronin and co-workers.[19]
$PhSi(iOPr)H_2$[25] (11.0 mg, 66.1 µmol, 3.2 equiv) was added dropwise to a degassed mixture of enone **S41** (9.5 mg, 20.4 µmol, 1.0 equiv) and $Fe(acac)_3$ (4.0 mg, 11.3 µmol, 0.6 equiv) in degassed[26] anhydrous EtOH (300 µL) at 0 °C. After 4 h, degassed aqueous $NaHCO_3$ (sat., 200 µL) was added, and the mixture was diluted with water and brine (200 µL each). The aqueous phase was extracted with Et_2O (3x 2 mL). The combined organic phases were washed with brine (2 mL), dried over Na_2SO_4 and all volatiles

[25] Reagent prepared according to the procedure reported by Yamada and co-workers.[20]
[26] Degassed by sonication under an argon atmosphere for 30 min.

were removed under reduced pressure. Purification by column chromatography (PE:EtOAc = 15:1) gave tricycle **S42** (4.4 mg, 9.4 µmol, 46%) as a colorless oil.

R_f (PE:EtOAc = 4:1; vanillin) = 0.64 (blue).
^{1}H-NMR (500 MHz, $CDCl_3$): *δ*(ppm) = 3.44 (d, *J* = 10.0 Hz, 1H, H-13a), 3.36 (d, *J* = 9.9 Hz, 1H, H-13b), 2.37–2.34 (m, 2H, H-{8,9}), 2.07–2.04 (m, 1H, H-6), 1.90–1.87 (m, 1H, H-4a), 1.86–1.79 (m, 3H, H-{5a,10a,10b}), 1.74–1.68 (m, 1H, H-4b), 1.41 (d, *J* = 10.0 Hz, 1H, H-5b), 1.29 (s, 3H, CH_3-14), 1.26 (s, 3H, CH_3-11), 1.13 (s, 3H, CH_3-12), 0.97 (t, *J* = 7.9 Hz, 9H, OTES), 0.89 (s, 9H, OTBS), 0.60 (q, *J* = 7.9 Hz, 6H, OTES), 0.02 (s, 3H, OTBS), 0.01 (s, 3H, OTBS).
^{13}C-NMR (126 MHz, $CDCl_3$) δ(ppm) = 223.2 (C-1), 79.7 (C-3), 72.7 (C-2), 70.2 (C-13), 52.2 (C-9), 49.6 (C-6), 48.30 (C-8), 48.29 (C-7), 40.9 (C-4), 39.2 (C-10), 26.0 (OTBS), 24.8 (C-12), 23.9 (C-14), 22.6 (C-5), 18.3 (OTBS), 12.0 (C-11), 6.7 (OTES), –5.3 (OTBS), –5.5 (OTBS).
HRMS(ESI): Calcd for $C_{26}H_{50}O_3Si_2Na$ $[M+Na]^+$: 489.3196; found: 489.3201.

The relative stereoconfiguration was assigned based on NOE experiments:

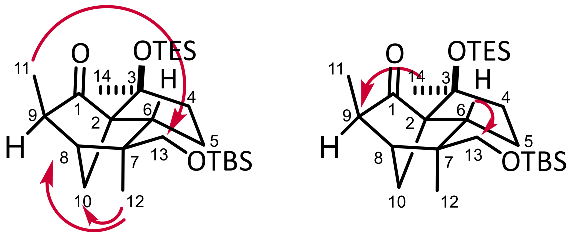

Figure S11. Signal assignment and relevant NOE correlations for cyclized product **S42**.

Ti(III)-initiated cyclization of epoxyenone S43

Epoxyenone S36

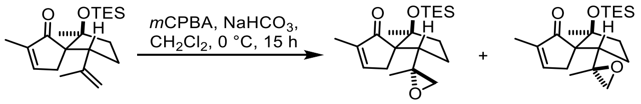

S36 **S43a** (47%) **S43b** (31%)

$NaHCO_3$ (62.3 mg, 742 µmol, 2.0 equiv) and *m*CPBA (75% in water, 128 mg, 556 µmol, 1.5 equiv) were added to a stirred solution of enone **S36** (*endo*:*exo* = 5:1, 124 mg, 371 µmol, 1.0 equiv) in CH_2Cl_2 (4.0 mL) at 0 °C. After 15 h, aqueous $Na_2S_2O_3$ (sat., 2 mL) and aqueous $NaHCO_3$ (sat., 2 mL) were added. The mixture was diluted with CH_2Cl_2 (5 mL), and stirred vigorously until two clear phases were obtained. The phases were separated and the aqueous phase was extracted with CH_2Cl_2 (3x 5 mL). The combined organic phases were washed with $NaHCO_3$ (15 mL) and brine (15 mL), dried over Na_2SO_4 and concentrated under reduced pressure. Purification *via* column chromatography (PE:EtOAc = 25:1 to 10:1) gave epoxide **S43a** (60.5 mg, 173 µmol, 47%) as a coloress oil and **S43b** (40.3 mg, 115 µmol, 31%) as a white solid.
The relative configuration of the epoxides could not be determined. For the purpose of trying the Ti-mediated reductive cyclization, both diastereomers were distinguished by their R_f-values:

Analytical data for **S43a**:[27]
R_f (PE:EtOAc = 10:1; vanillin) = 0.42 (bright-green).
^{1}H-NMR (400 MHz, $CDCl_3$): *δ*(ppm) = 7.22–7.17 (m, 1H), 3.40 (t, *J* = 8.8 Hz, 1H), 2.89–2.80 (m, 1H), 2.50 (d, *J* = 4.8 Hz, 1H), 2.35 (d, *J* = 4.8 Hz, 1H), 2.22–2.13 (m, 1H), 1.80–1.76 (m, 3H), 1.75–1.59 (m, 3H), 1.38–1.29 (m, 1H), 1.07 (s, 3H), 0.97 (s, 3H), 0.93 (t, *J* = 7.9 Hz, 9H), 0.60–0.52 (m, 6H).
^{13}C-NMR (101 MHz, $CDCl_3$): *δ*(ppm) = 207.9, 153.6, 142.5, 87.1, 63.8, 56.8, 51.4, 46.0, 39.6, 34.6, 22.3, 22.0, 20.8, 10.7, 7.3, 6.8.
HRMS(ESI): Calcd for $[M+Na]^+$: $C_{20}H_{34}O_3SiNa$: 373.2175; found: 373.2184.

Analytical data for **S43b**:
R_f (PE:EtOAc = 10:1; vanillin) = 0.32 (dark-green).

[27] Epoxide **S43a** was obtained as an inseparable mixture with two other compounds which we identified as the epoxides of *exo*-**S36:**

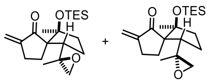

^{1}H-NMR (400 MHz, $CDCl_3$): δ(ppm) = 7.19–7.15 (m, 1H), 3.22 (t, J = 9.1 Hz, 1H), 2.76–2.66 (m, 1H), 2.59 (d, J = 4.4 Hz, 1H), 2.47 (d, J = 4.4 Hz, 1H), 2.25–2.16 (m, 1H), 1.98–1.84 (m, 2H), 1.80–1.72 (m, 3H), 1.67–1.57 (m, 1H), 1.36–1.27 (m, 1H), 1.13 (s, 6H), 0.92 (t, J = 7.9 Hz, 9H), 0.60–0.49 (m, 6H).
^{13}C-NMR (101 MHz, $CDCl_3$): δ(ppm) = 208.4, 153.6, 142.0, 86.6, 63.3, 57.7, 52.0, 46.5, 39.6, 33.7, 23.2, 23.1, 22.8, 10.7, 7.2, 6.7.
m.p. (CH_2Cl_2) = 62–66 °C.
HRMS(ESI): Calcd for $[M+Na]^+$: $C_{20}H_{34}O_3SiNa$: 373.2175; found: 373.2169.
The relative stereoconfiguration of **S43b** was determined by X-ray crystallography.

Tricycle S44

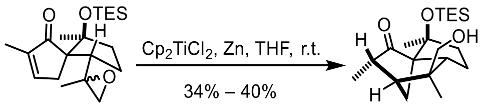

S43 **S44**

The cyclization of **S43** was performed according to a variation of the procedure published by Bermejo and co-workers.[11]
Degassed[28] THF (2.5 mL) was added at r.t. to a mixture of Cp_2TiCl_2 (59.7 mg, 240 µmol, 3.0 equiv) and Zn powder (47.0 mg, 719 µmol, 9.0 equiv). After 70 min, a solution of epoxide **S43a** (28.0 mg, 79.9 µmol, 1.0 equiv) in degassed[28] THF (2.5 mL) was added dropwise at r.t. over 6 min. After 45 min, the reaction was quenched by addition of aqueous NaH_2PO_4 (10 wt%, 1 mL) and brine (1 mL). The mixture was stirred vigorously for 30 min, diluted with MTBE (5 mL), the phases were separated and the aqueous phase was extracted with MTBE (3x 5 mL). The combined organic phases were washed with brine (10 mL), dried over Na_2SO_4 and concentrated under reduced pressure. Purification by column chromatography (PE:EtOAc = 10:1) gave cyclized product **S44** (9.5 mg, 26.9 µmol, 34%) as a colorless oil.

R*f* (PE:EtOAc =2:1; vanillin) = 0.64 (turquoise).
^{1}H-NMR (600 MHz, C_6D_6): δ(ppm) = 3.09 (d, J = 10.4 Hz, 1H, H-13a), 3.02 (d, J = 10.4 Hz, 1H, H-13b), 2.22–2.16 (m, 1H, H-8), 2.13–2.08 (m, 1H, H-5), 1.92–1.81 (m, 1H, H-4a), 1.72–1.65 (m, 2H, H-{3a,7}), 1.56–1.45 (m, 2H, H-{3b,4b}), 1.35–1.31 (m, 1H, H-10a), 1.31–1.27 (m, 1H, H-10b), 1.29 (s, 3H, CH_3-11), 1.15 (t, J = 7.9 Hz, 9H, OTES), 1.01 (d, J = 7.4 Hz, 3H, CH_3-14), 1.00 (s, 3H, CH_3-12), 0.88–0.80 (m, 3H, OTES), 0.77–0.68 (m, 3H, OTES), 0.60 (bs, 1H, OH).
^{13}C-NMR (151MHz, C_6D_6): δ(ppm) = 212.6 (C-9), 80.7 (C-2), 73.0 (C-1), 69.3 (C-13), 49.0 (C-7), 48.6 (C-5), 47.0 (C-6), 43.6 (C-8), 42.4 (C-3), 36.0 (C-10), 23.2 (C-11), 22.0 (C-12), 21.8 (C-4), 14.9 (C-14), 7.6 (OTES), 7.3 (OTES).
HRMS(ESI): Calcd for $C_{20}H_{36}O_3SiNa$ $[M+Na]^+$: 375.2331; found: 375.2339.

The relative configuration was assigned based on NOE experiments:

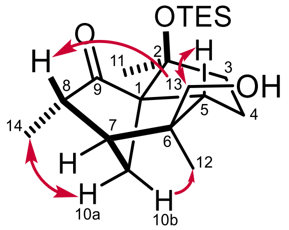

Figure S12. Signal assignment and relevant NOE correlations for cyclized product **S44**.

The same procedure was applied to epoxide **S43b** (18.0 mg, 51.3 µmol, 1.0 equiv) yielding cyclization product **S44** (7.3 mg, 20.7 µmol, 40%) as a colorless oil.
The cyclization of both epoxide diastereomers **S43a** and **S43b** afforded the same product **S44**.

---
[28] The freeze-pump-thaw technique was used.

5 X-ray data

Single-crystal X-ray crystallography was performed with a Bruker SMART X2S diffractometer. Structures were solved using SHELXT 2014/5 (Sheldrick, 2014). Structures were refined using SHELXL2018/3 (Sheldrick, 2018).
All crystal structures were obtained from racemic intermediates that were synthesized during the initial scouting of the reaction sequence.

Diol (±)-S18[29]

CCDC deposition number	2238017	
Chemical formula	$C_{15}H_{22}O_2$	
Molecular weight	234.32 g/mol	
Temperature	200 K	
Radiation	MoKα (0.71073 Å)	
Crystal size	0.600 x 0.300 x 0.100 mm	
Crystal system	monoclinic	
Space group	$P2_1/c$	
Unit cell dimensions	a = 11.9587 (19) Å	α = 90°
	b = 7.9774 (10) Å	β = 108.128(4)°
	c = 15.082 (5) Å	γ = 90°
Volume	1367.4(4) $Å^3$	
Z	4	
Density (calculated)	1.148 g/cm^3	
Absorption coefficient	0.073	
F(000)	520	
Theta range for data collection	2.83° to 24.09°	
Index ranges	–15 ≤ h ≤ 15, –10 ≤ k ≤ 10, –19 ≤ l ≤ 19	
Reflections collected	28213	
Independent reflections	2973 [R_{int} = 0.118]	
Absorption correction	multi-scan, SADABS-2016/2 - Bruker AXS area detector scaling and absorption correction	
Max. and min. transmission	0.99 and 0.49	
Refinement method	Full-matrix least-squares on F^2	
Refinement program	SHELXL-2018/3 (Sheldrick, 2018)	
Data / restraints / parameters	2973 / 0 / 161	
Goodness-of-fit on F^2	1.052	
Final R indices [I>2σ(I)]	R_1 = 0.0549, wR_2 = 0.1396	
Final R indices [all data]	R_1 = 0.0769, wR_2 = 0.1503	
H atom treatment	mixture of independent and constrained refinement	
Largest diff. peak and hole	0.281 and –0.178 $Å^{-3}$	

[29] Enantiomeric structure was generated while solving the crystal structure. As stated above, the crystals for X-ray crystallography were obtained from racemic (±)-**S18**. This intermediate was the first one in the sequence that allowed confirming the relative configuration of the tertiary alcohol and the isopropenyl group by X-ray crystallography. In the article, focusing on the asymmetric synthesis of illisimonin A, the enantiomeric projection of **S18** is used.

Epoxide (±)-S21a

CCDC deposition number	2238015
Chemical formula	$C_{33}H_{36}O_4Si$ (**S21a** + C_6H_6)[30]
Molecular weight	524.94 g/mol (**S21a** + C_6H_6)
Temperature	200 K
Radiation	MoKα (0.71073 Å)
Crystal size	0.600 x 0.49 x 0.46 mm
Crystal system	triclinic
Space group	$P\bar{1}$
Unit cell dimensions	a = 11.074(12) Å α = 72.63(5)°
	b = 11.380(14) Å β = 76.53(3)°
	c = 12.423(19) Å γ = 80.99°
Volume	1447 $Å^3$
Z	2
Density (calculated)	1.205 g/cm^3
Absorption coefficient	0.12 mm^{-1}
F(000)	560
Theta range for data collection	2.2° to 23.1°
Index ranges	–13 ≤ h ≤ 13, –12 ≤ k ≤ 14, –13 ≤ l ≤ 15
Reflections collected	10144
Independent reflections	5673 [R_{int} = 0.090]
Absorption correction	multi-scan, SADABS-2016/2 - Bruker AXS area detector scaling and absorption correction
Max. and min. transmission	0.95 and 0.25
Refinement method	Full-matrix least-squares on F^2
Refinement program	SHELXL-2018/3 (Sheldrick, 2018)
Data / restraints / parameters	5673 / 354 / 401
Goodness-of-fit on F^2	0.97
Final R indices [I>2σ(I)]	R_1 = 0.0825, wR_2 = 0.1958
Final R indices [all data]	R_1 = 0.1324, wR_2 = 0.2280
H atom treatment	constrained
Largest diff. peak and hole	0.438 and –0.572 $Å^{-3}$

[30] Epoxide **S21a** co-crystallized with benzene.

(±)-5-*epi*-S25

CCDC deposition number	2238018
Chemical formula	$C_{33}H_{46}O_5Si_2$ + C_6H_6[31]
Molecular weight	655.89 g/mol (5-*epi*-**S25** + C_6H_6)
Temperature	200 K
Radiation	MoKα (0.71073 Å)
Crystal size	0.84 x 0.73 x 0.19 mm
Crystal system	monoclinic
Space group	$P2_1/c$
Unit cell dimensions	a = 19.8948 Å α = 90°
	b = 10.4978 Å β = 108.347
	c = 18.9207 Å γ = 90°
Volume	3750.7 $Å^3$
Z	62
Density (calculated)	1.161 g/cm^3
Absorption coefficient	0.14 mm^{-1}
F(000)	1414
Theta range for data collection	2.2° to 27.1°
Index ranges	$-25 \le h \le 20$, $-13 \le k \le 12$, $-22 \le l \le 23$
Reflections collected	29795
Independent reflections	8530 [R_{int} = 0.081]
Absorption correction	multi-scan, SADABS-2016/2 - Bruker AXS area detector scaling and absorption correction
Max. and min. transmission	0.97 and 0.51
Refinement method	Full-matrix least-squares on F^2
Refinement program	SHELXL-2018/3 (Sheldrick, 2018)
Data / restraints / parameters	8530 / 348 / 482
Goodness-of-fit on F^2	1.03
Final R indices [I>2σ(I)]	R_1 = 0.0635, wR_2 = 0.1523
Final R indices [all data]	R_1 = 0.0873, wR_2 = 0.1678
H atom treatment	mixture of independent and constrained refinement
Largest diff. peak and hole	0.609 and –0.306 $Å^{-3}$

[31] Rearrangement product (±)-5-*epi*-**S25** co-crystallized with benzene.

Epoxide (±)-S43b

CCDC deposition number	2238016
Chemical formula	$C_{20}H_{34}O_3Si$
Molecular weight	350.56 g/mol
Temperature	200 K
Radiation	MoKα (0.71073 Å)
Crystal size	0.83 x 0.48 x 0.19 mm
Crystal system	monoclinic
Space group	$P2_1/c$
Unit cell dimensions	a = 8.3239 Å α = 90°
	b = 19.292(3) Å β = 102.8°
	c = 13.3143 Å γ = 90°
Volume	$Å^3$
Z	4
Density (calculated)	1.117 g/cm^3
Absorption coefficient	0.126 mm^{-1}
F(000)	768
Theta range for data collection	2.5° to 27.6°
Index ranges	–10 ≤ h ≤ 10, –25 ≤ k ≤ 14, –16 ≤ l ≤ 15
Reflections collected	20212
Independent reflections	3177 [R_{int} = 0.087]
Absorption correction	multi-scan, SADABS-2016/2 - Bruker AXS area detector scaling and absorption correction
Max. and min. transmission	0.98 and 0.57
Refinement method	Full-matrix least-squares on F^2
Refinement program	SHELXL-2018/3 (Sheldrick, 2018)
Data / restraints / parameters	4675 / 0 / 223
Goodness-of-fit on F^2	1.05
Final R indices [I>2σ(I)]	R_1 = 0.1031, wR_2 = 0.2573
Final R indices [all data]	R_1 = 0.1415, wR_2 = 0.2830
H atom treatment	constrained
Largest diff. peak and hole	0.698 and –0.457 $Å^{-3}$

6 References

[1] Simon Lancaster, *ChemSpider Synth.* **2003**, 215.

[2] M. Frigerio, M. Santagostino, S. Sputore, *J. Org. Chem.* **1999**, *64*, 4537–4538.

[3] Y. Gao, K. B. Sharpless, J. M. Klunder, R. M. Hanson, S. Y. Ko, H. Masamune, *J. Am. Chem. Soc.* **1987**, *109*, 5765–5780.

[4] M. S. Chen, M. C. White, *Science* **2007**, *318*, 783–787.

[5] C. Etling, G. Tedesco, M. Kalesse, *Chem. - A Eur. J.* **2021**, *27*, 9257–9262.

[6] L. López-Suárez, L. Riesgo, F. Bravo, T. T. Ransom, J. A. Beutler, A. M. Echavarren, *ChemMedChem* **2016**, *11*, 1003–1007.

[7] D. K. Mohapatra, C. Pramanik, M. S. Chorghade, M. K. Gurjar, *Eur. J. Org. Chem.* **2007**, *2007*, 5059–5063.

[8] W. Pluempanupat, W. Chavasiri, *Tetrahedron Lett.* **2006**, *47*, 6821–6823.

[9] X. Zhang, X. Xie, Y. Liu, *J. Am. Chem. Soc.* **2018**, *140*, 7385–7389.

[10] N. C. Wilde, M. Isomura, A. Mendoza, P. S. Baran, *J. Am. Chem. Soc.* **2014**, *136*, 4909–4912.

[11] F. A. Bermejo, A. Fernández Mateos, A. Marcos Escribano, R. Martín Lago, L. Mateos Burón, M. Rodríguez López, R. Rubio González, *Tetrahedron* **2006**, *62*, 8933–8942.

[12] A. S. Burns, S. D. Rychnovsky, *J. Am. Chem. Soc.* **2019**, *141*, 13295–13300.

[13] S.-G. Ma, M. Li, M.-B. Lin, L. Li, Y.-B. Liu, J. Qu, Y. Li, X.-J. Wang, R.-B. Wang, S. Xu, Q. Hou, S.-S. Yu, *Org. Lett.* **2017**, *19*, 6160–6163.

[14] M. E. Jung, W. S. Lee, D. Sun, *Org. Lett.* **1999**, *1*, 307–309.

[15] K. Tanino, K. Onuki, K. Asano, M. Miyashita, T. Nakamura, Y. Takahashi, I. Kuwajima, *J. Am. Chem. Soc.* **2003**, *125*, 1498–1500.

[16] T. Mohri, Y. Takahashi, E. Kwon, S. Kuwahara, Y. Ogura, *Org. Lett.* **2020**, *22*, 9234–9238.

[17] C. M. Marson, A. J. Walker, J. Pickering, A. D. Hobson, R. Wrigglesworth, S. J. Edge, *J. Org. Chem.* **1993**, *58*, 5944–5951.

[18] J. C. Lo, D. Kim, C. M. Pan, J. T. Edwards, Y. Yabe, J. Gui, T. Qin, S. Gutiérrez, J. Giacoboni, M. W. Smith, P. L. Holland, P.S. Baran, *J. Am. Chem. Soc.* **2017**, *139*, 2484–2503.

[19] D. T. George, E. J. Kuenstner, S. V. Pronin, *J. Am. Chem. Soc.* **2015**, *137*, 15410–15413.

[20] Y. Gunji, Y. Yamashita, T. Ikeno, T. Yamada, *Chem. Lett.* **2006**, *35*, 714–715.

7 NMR Spectra

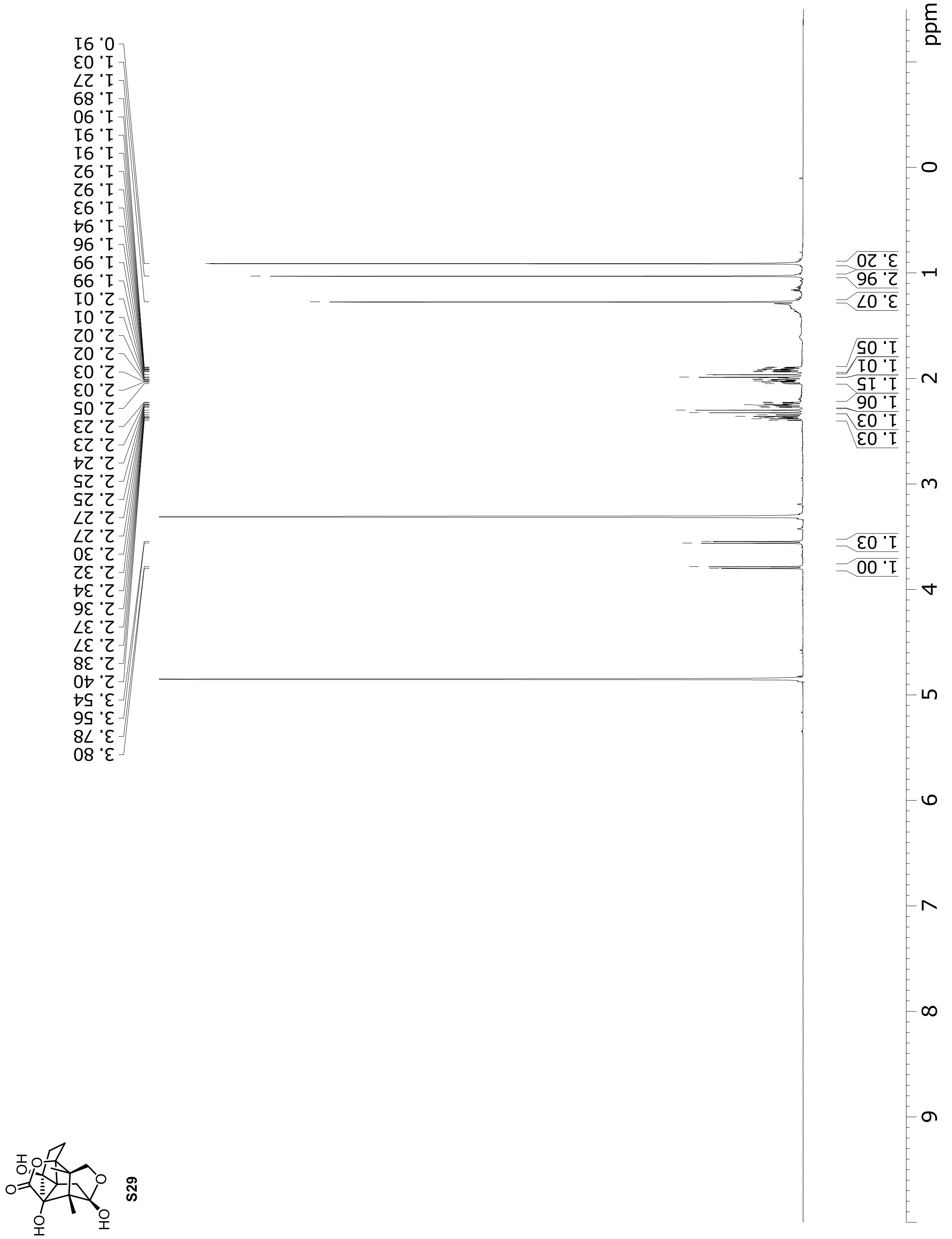

^{1}H NMR spectrum of illisimonin A (**S29**) measured in methanol-d_4 at 600 MHz.

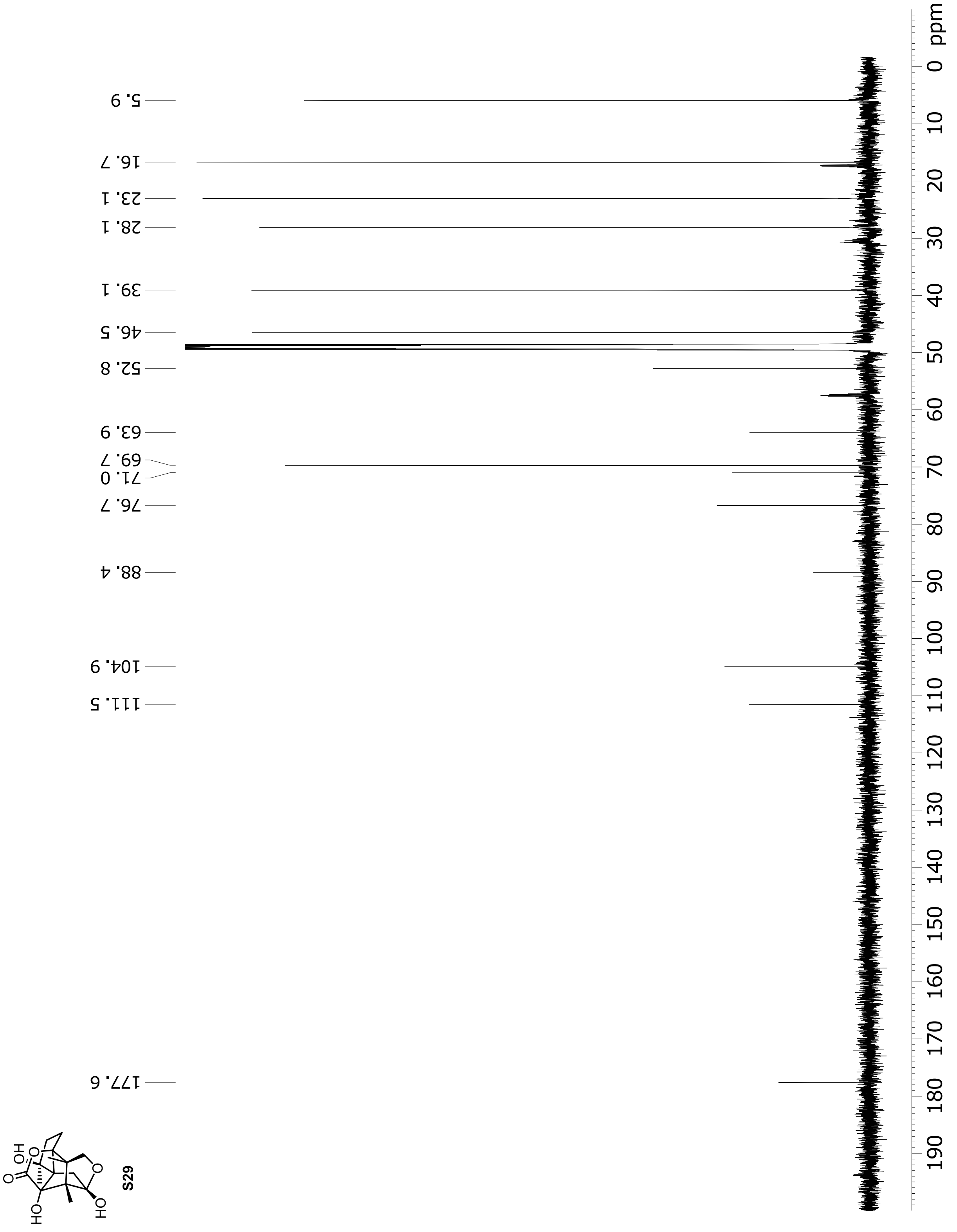

[13]**C NMR spectrum** of illisimonin A (**S29**) measured in methanol-d_4 at 151 MHz.

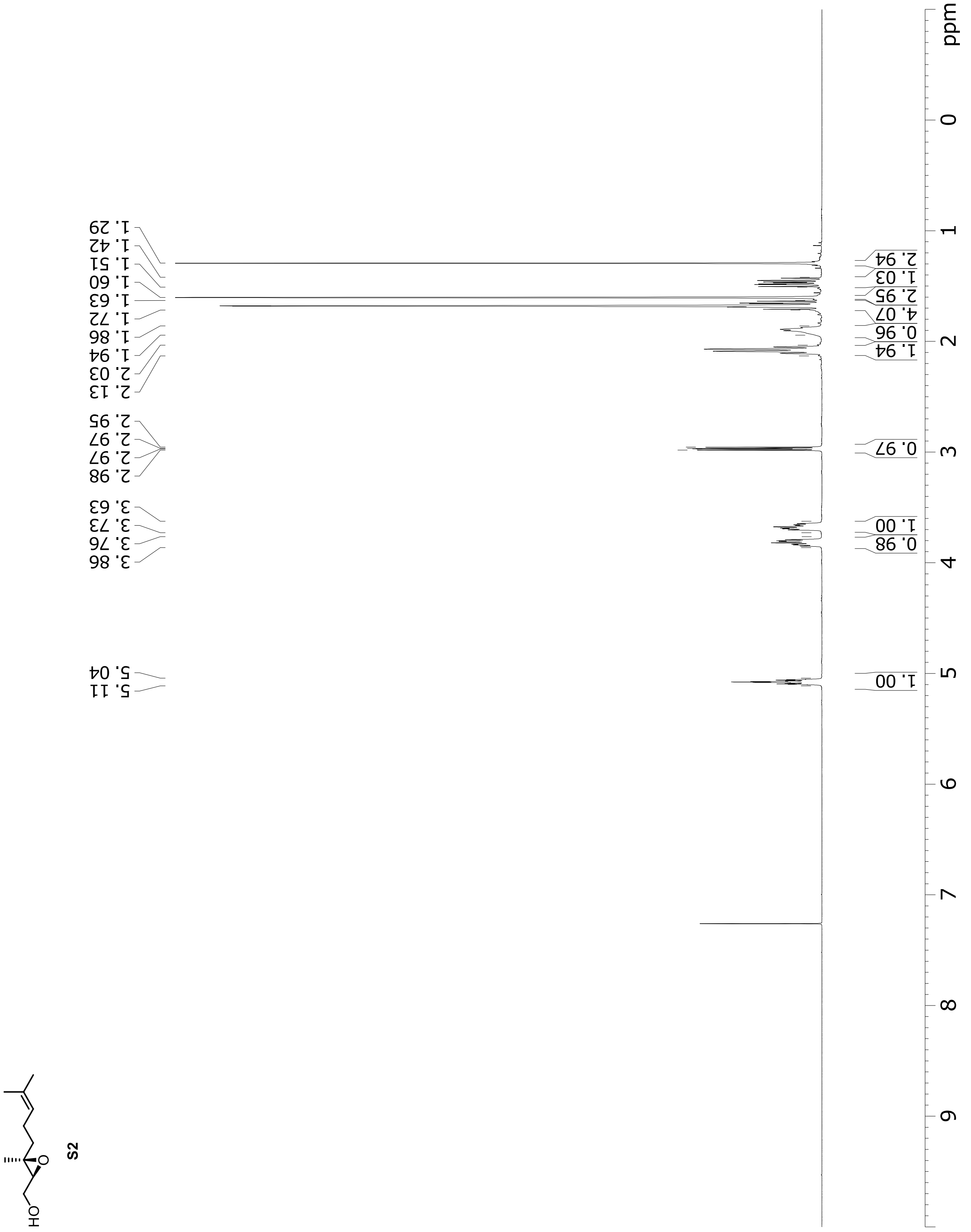

^{1}H NMR spectrum of epoxy alcohol **S2** measured in $CDCl_3$ at 400 MHz.

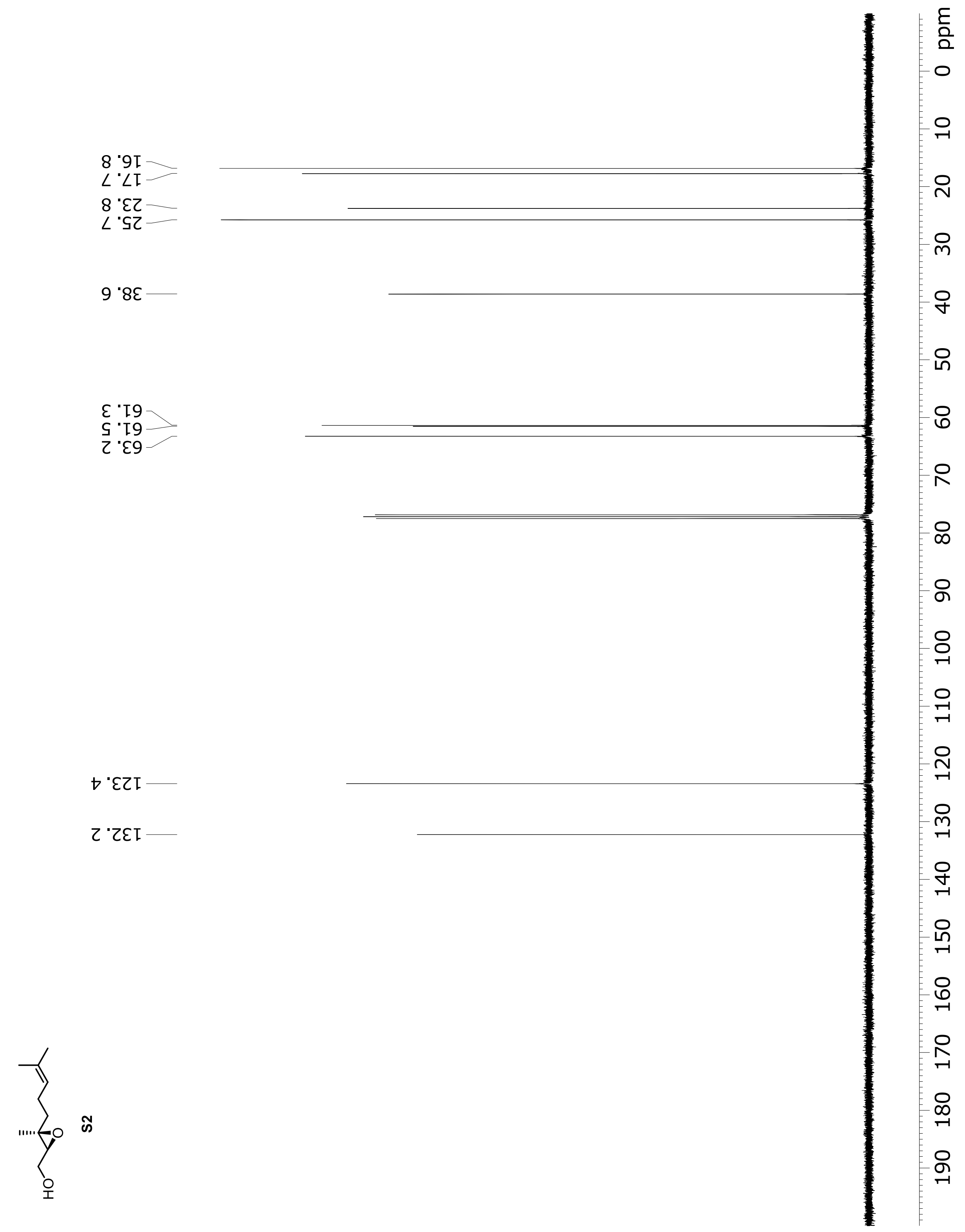

^{13}C NMR spectrum of epoxy alcohol **S2** measured in $CDCl_3$ at 101 MHz.

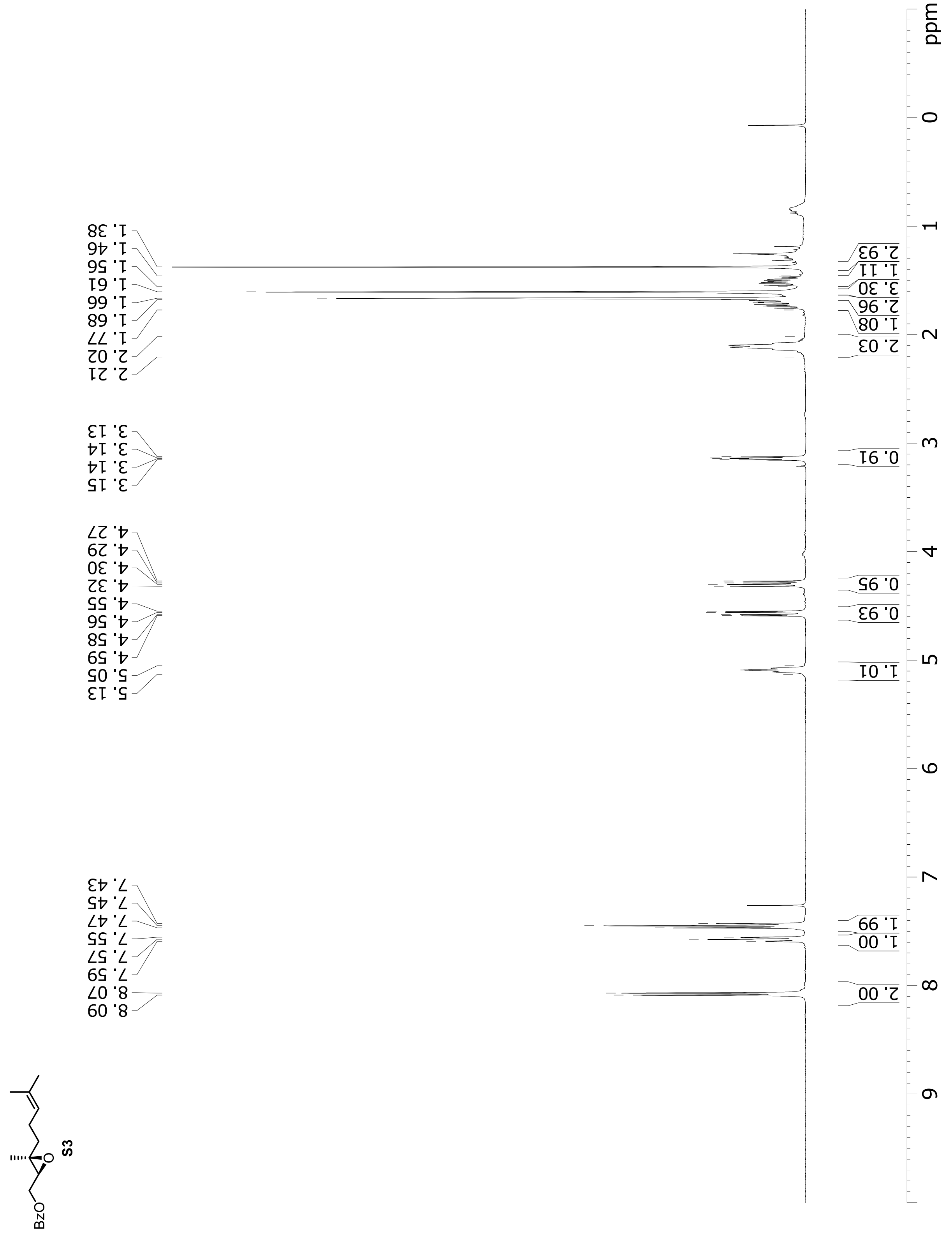

^{1}H NMR spectrum of benzoate **S3** measured in $CDCl_3$ at 400 MHz.

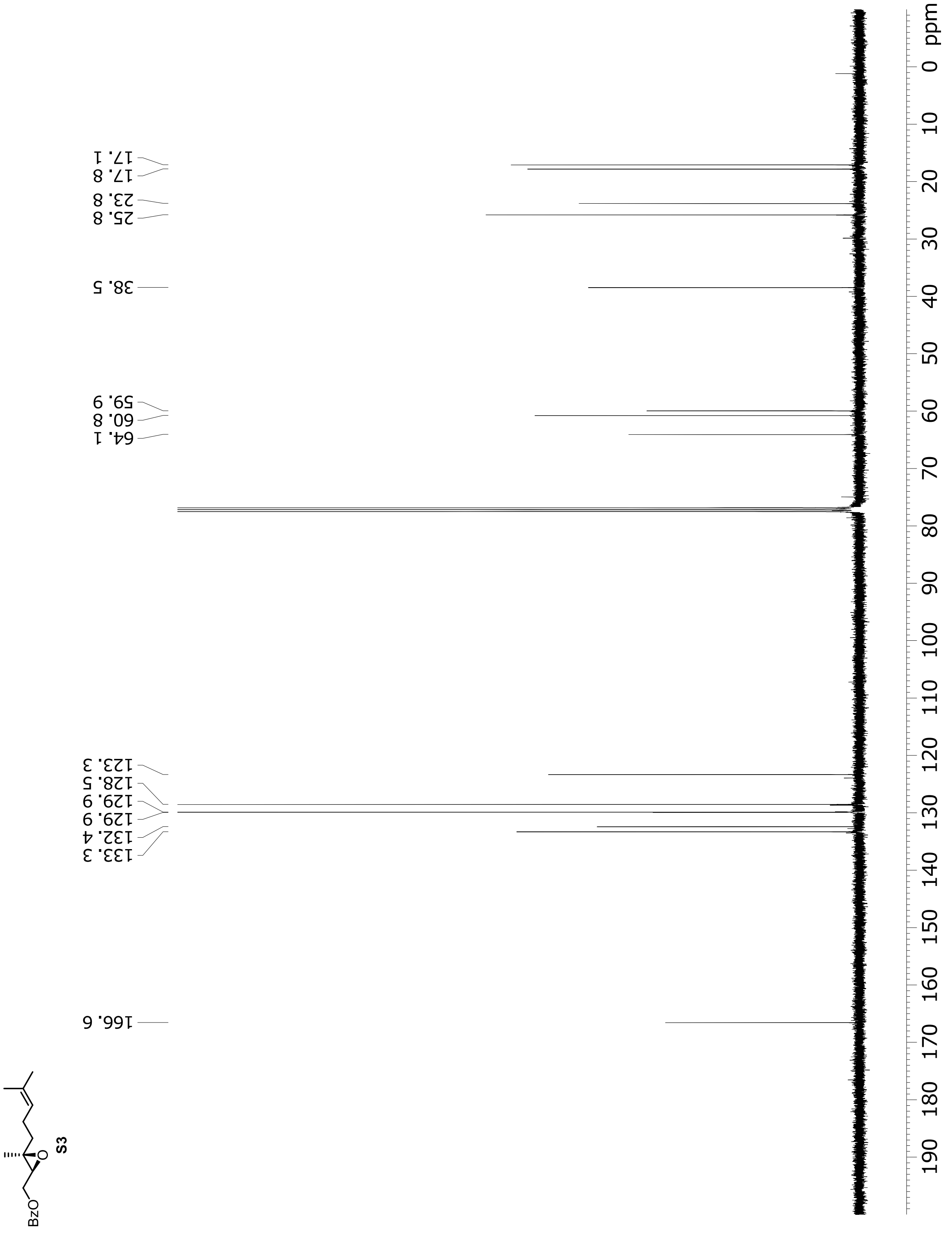

^{13}C NMR spectrum of benzoate **S3** measured in $CDCl_3$ at 101 MHz.

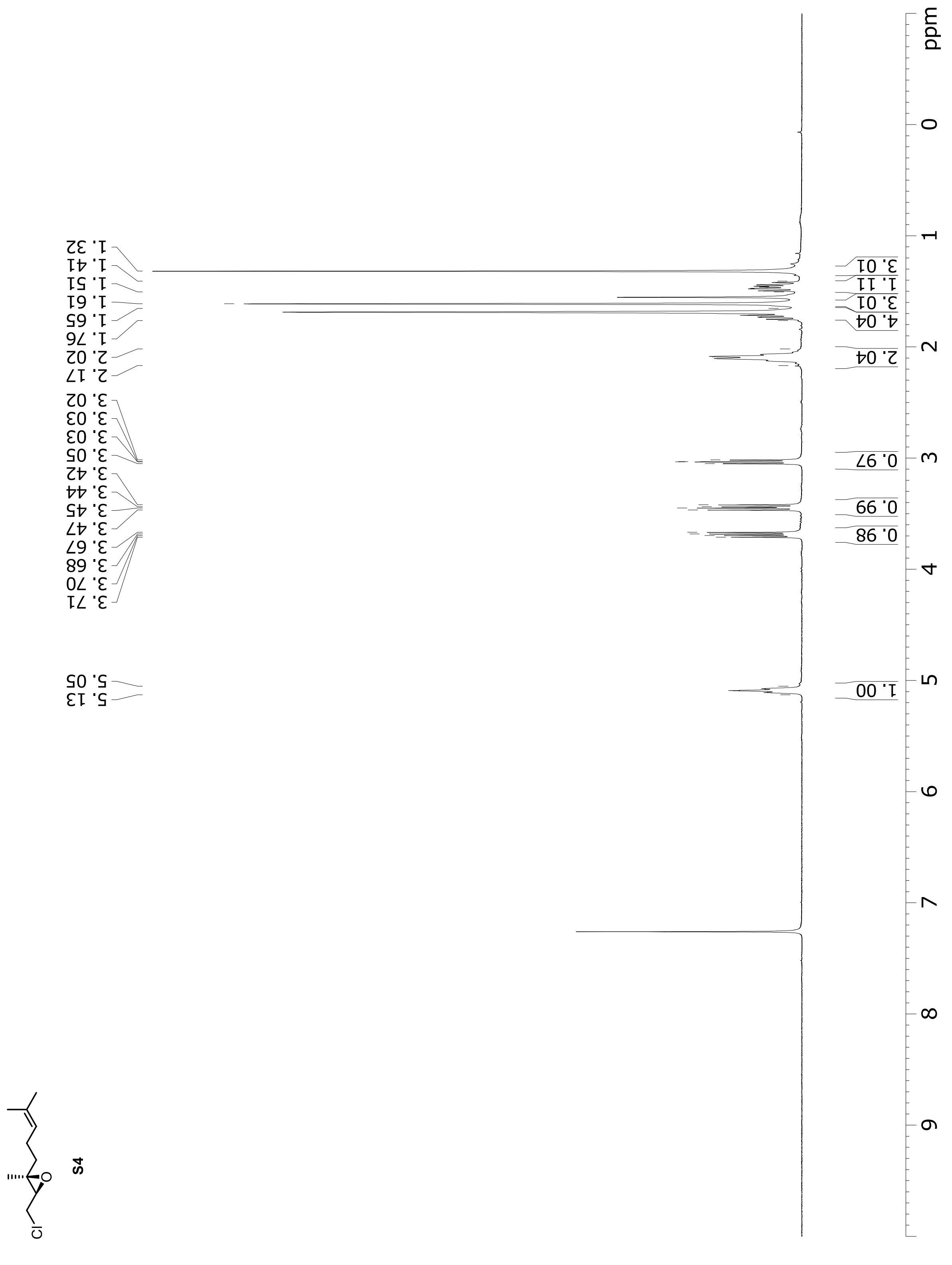

^{1}H NMR spectrum of epoxy chloride **S4** measured in $CDCl_3$ at 400 MHz.

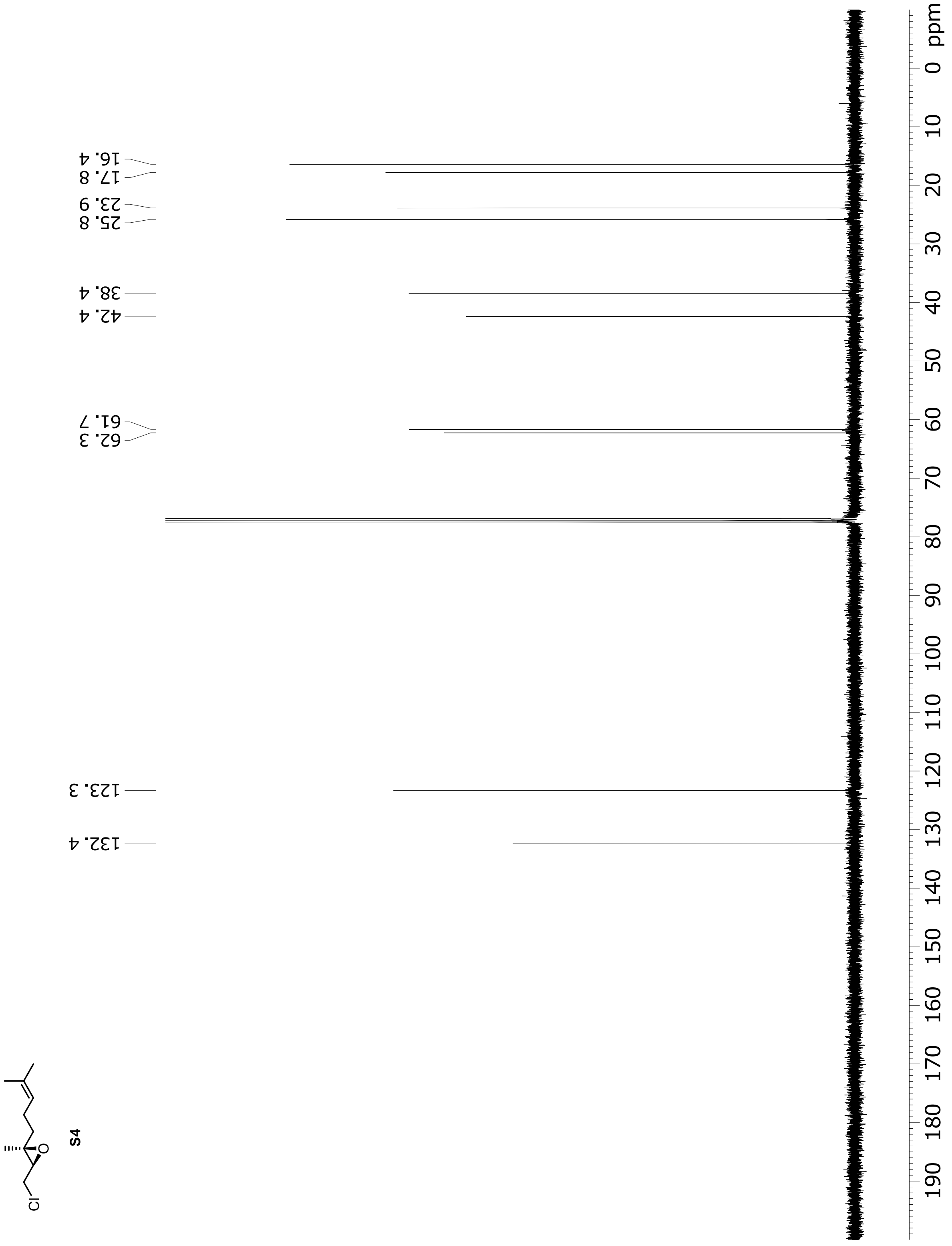

^{13}C NMR spectrum of epoxy chloride **S4** measured in $CDCl_3$ at 101 MHz.

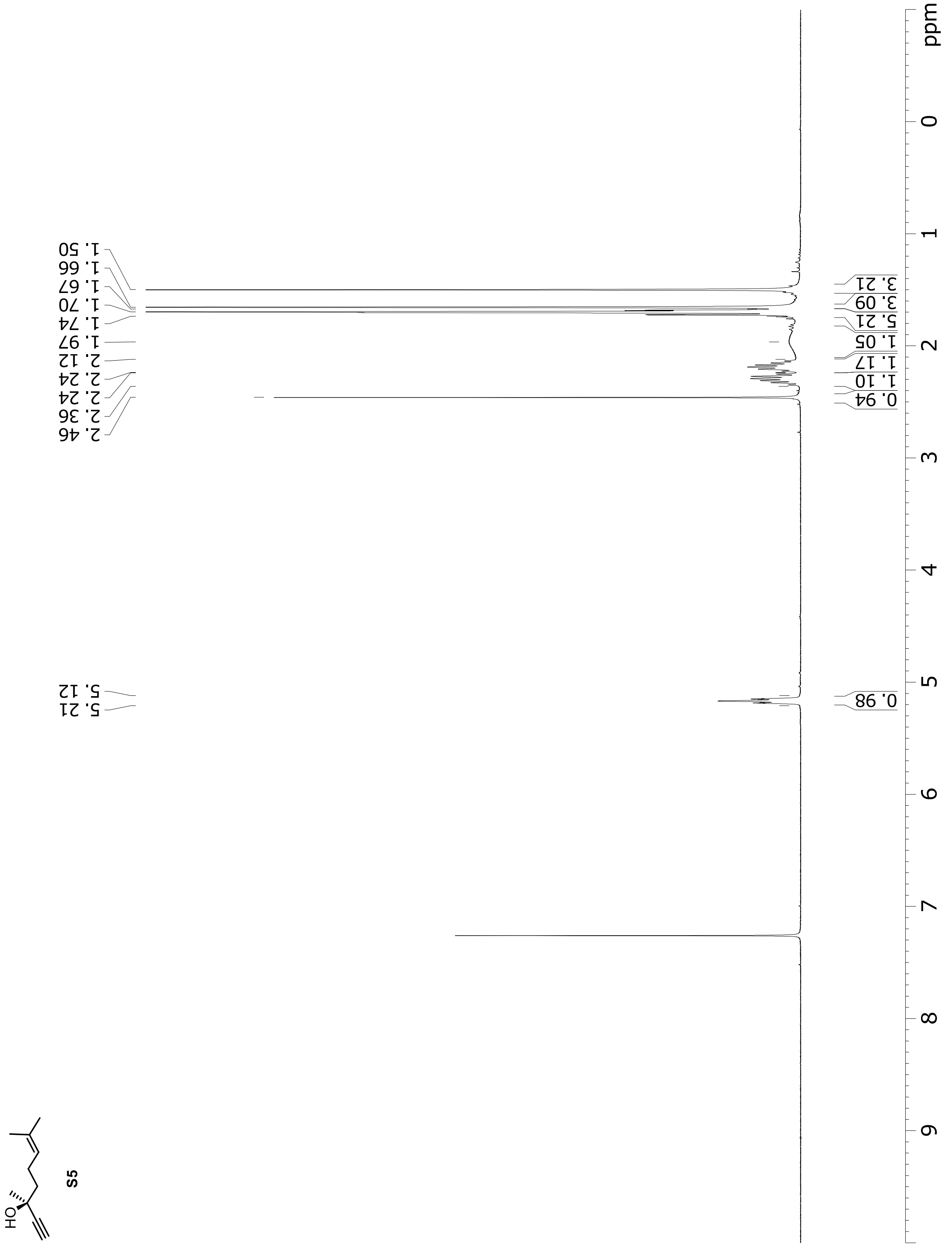

^{1}H NMR spectrum of propargyl alcohol **S5** measured in $CDCl_3$ at 400 MHz.

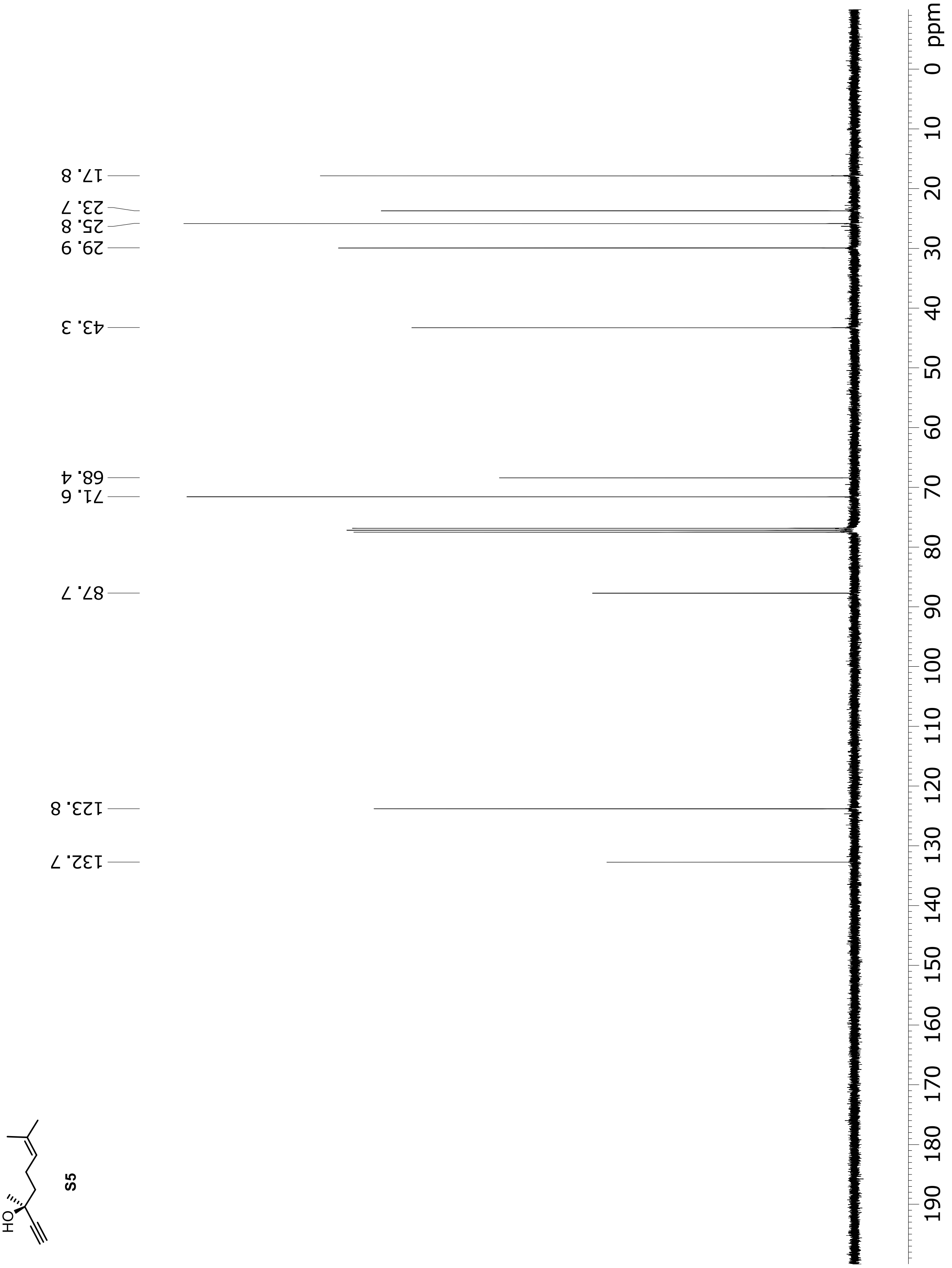

[13]**C NMR spectrum** of propargyl alcohol **S5** measured in $CDCl_3$ at 101 MHz.

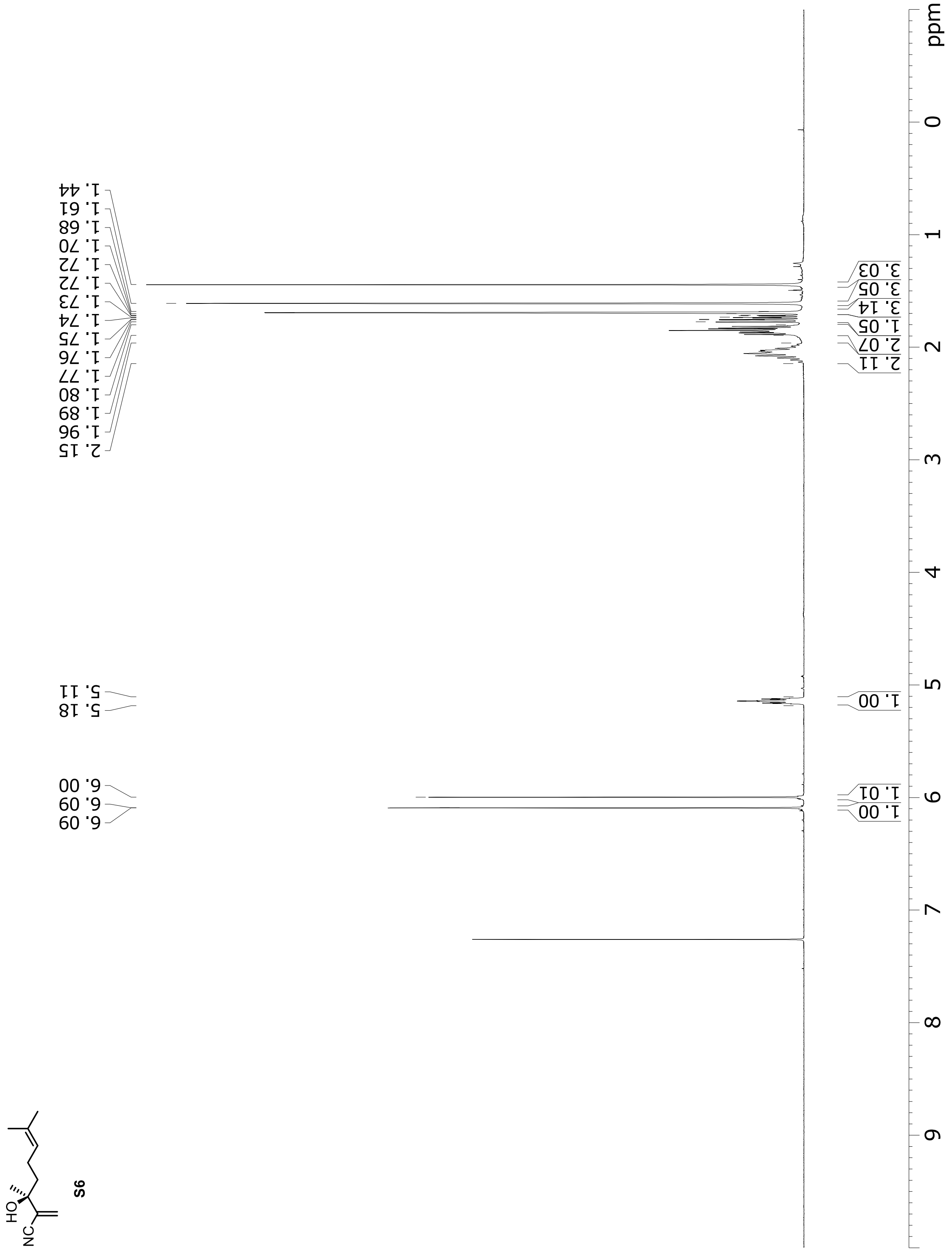

^{1}H NMR spectrum of nitrile **S6** measured in $CDCl_3$ at 400 MHz.

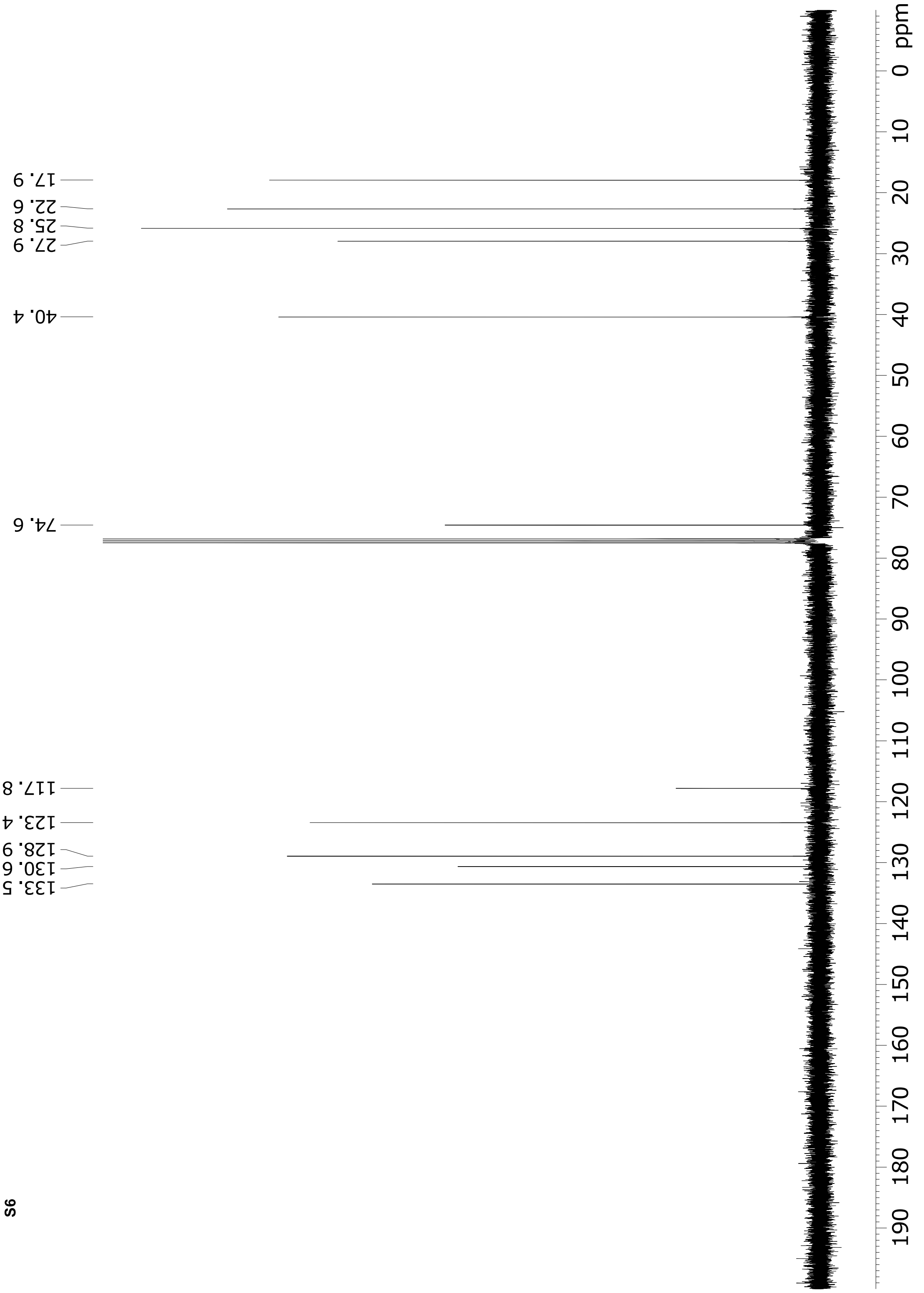

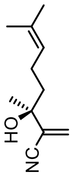

S6

^{13}C NMR spectrum of nitrile **S6** measured in $CDCl_3$ at 101 MHz.

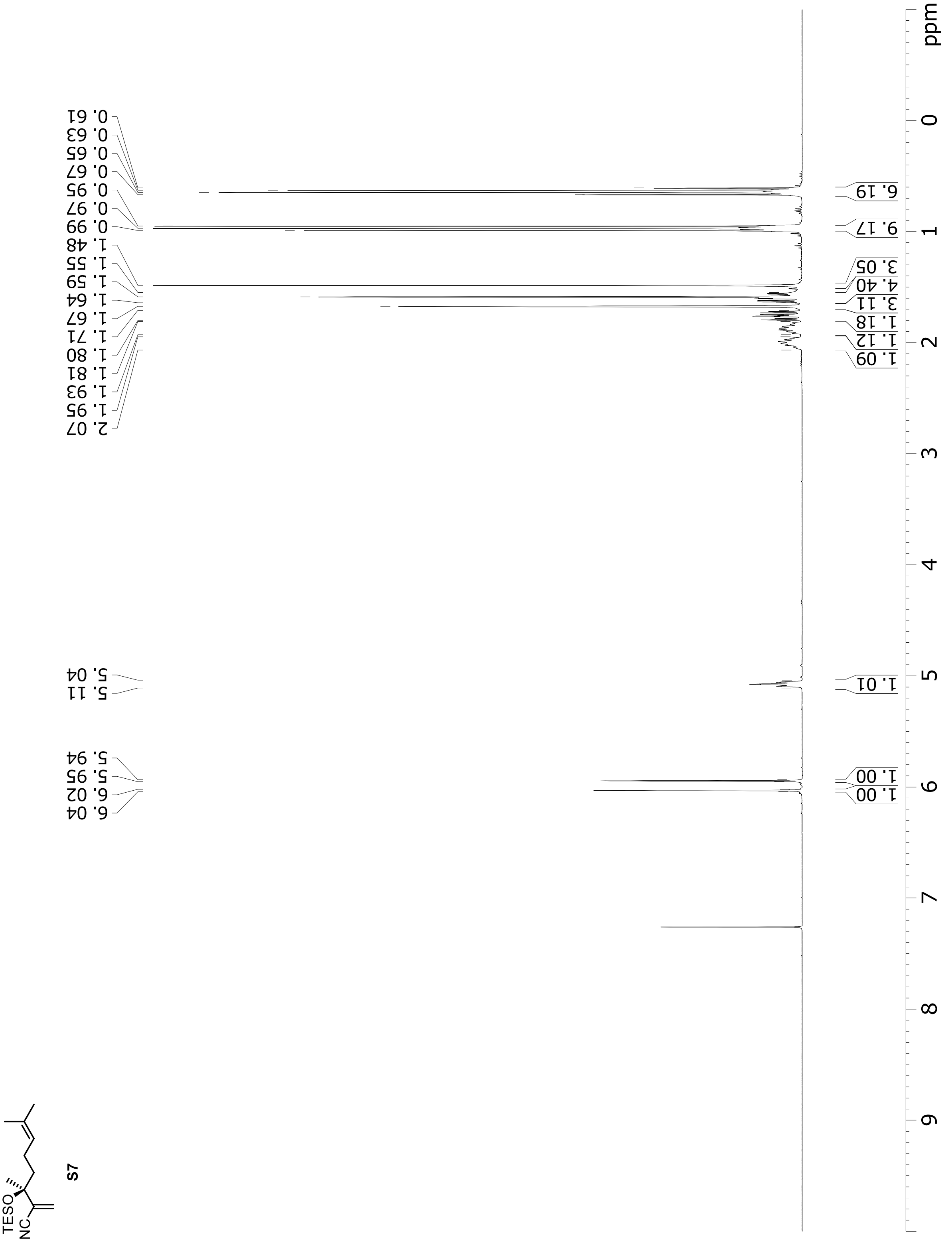

^{1}H NMR spectrum of TES-protected hydroxy nitrile **S7** measured in $CDCl_3$ at 400 MHz.

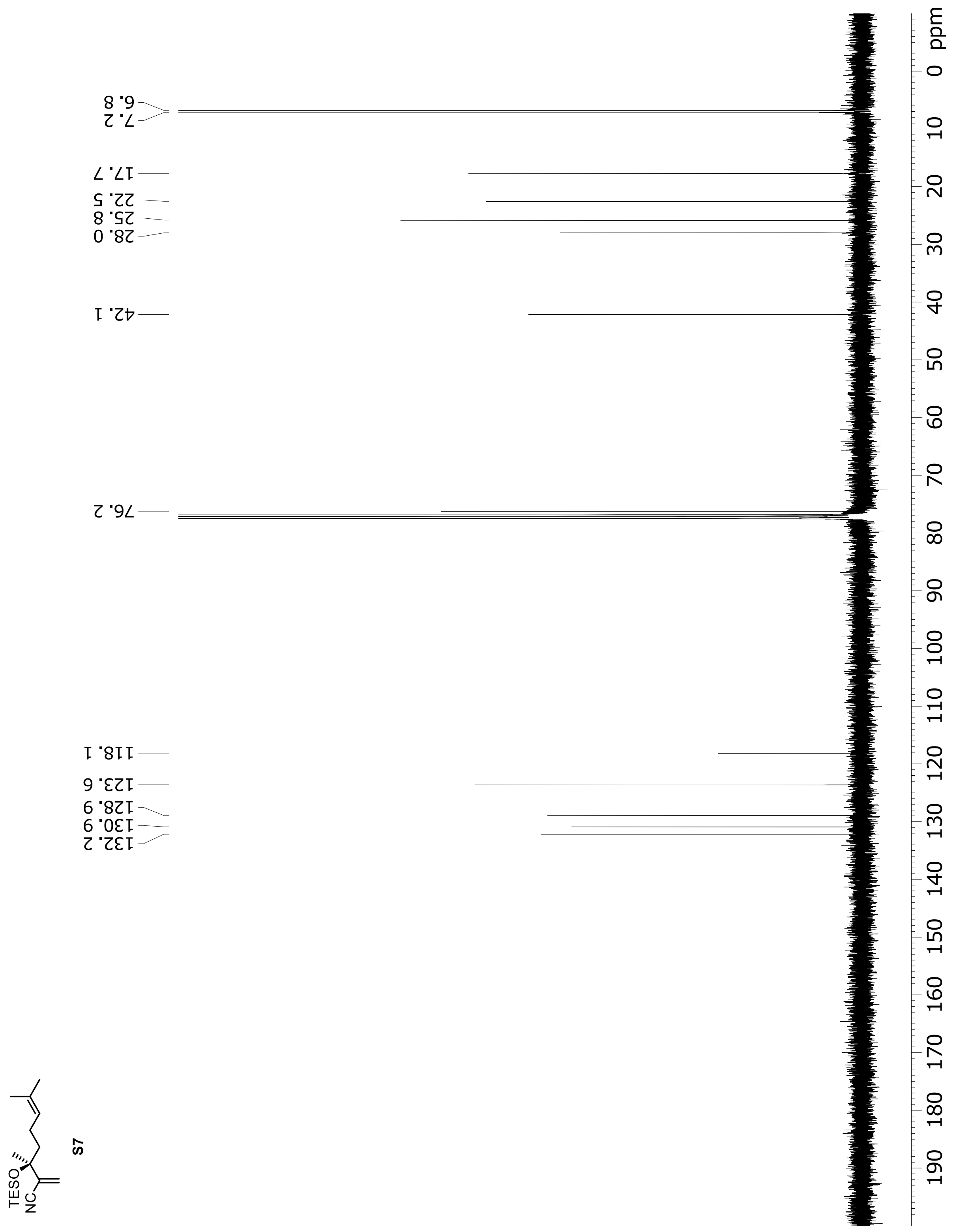

^{13}C NMR spectrum of TES-protected hydroxyl nitrile **S7** measured in $CDCl_3$ at 101 MHz.

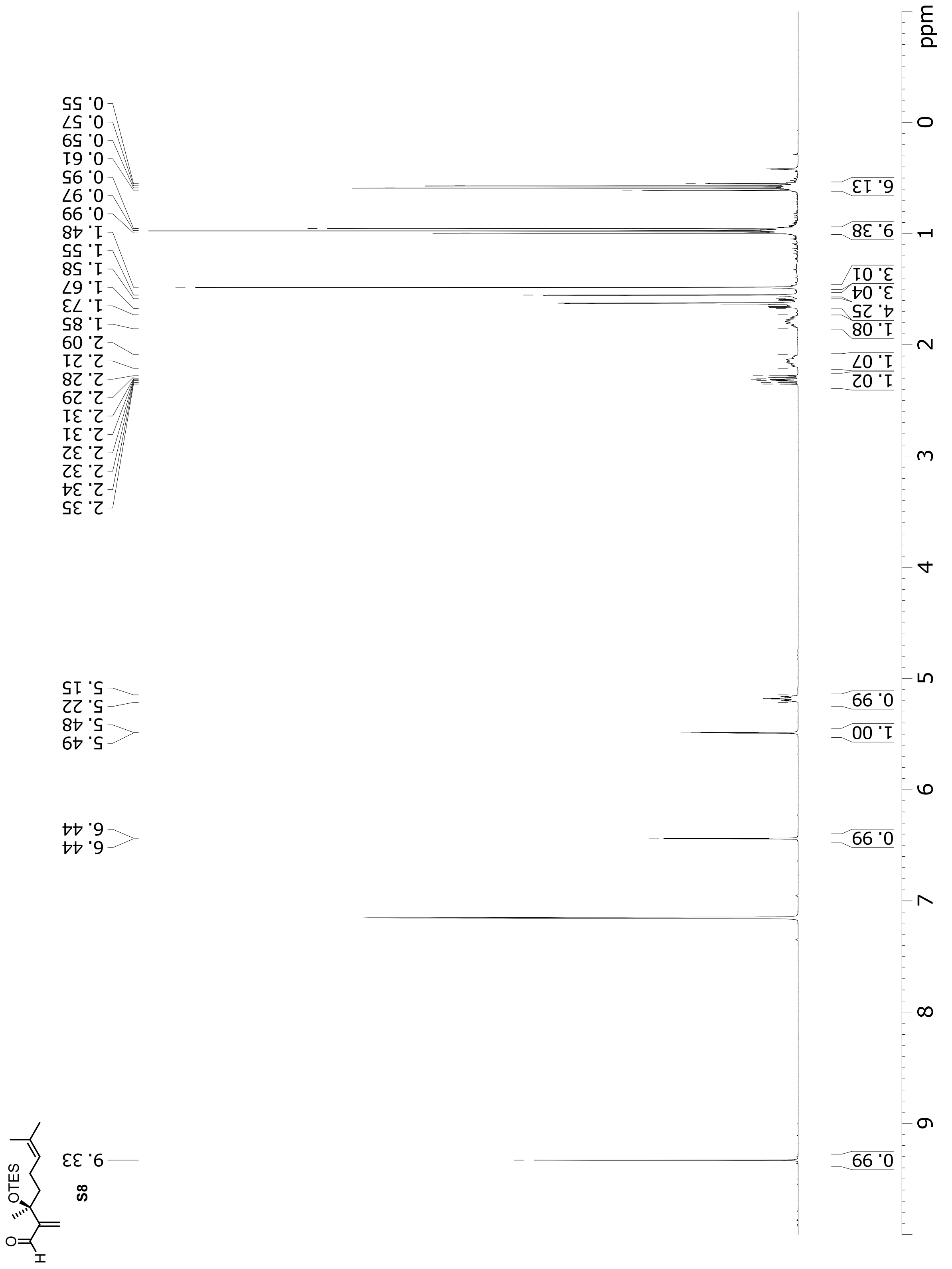

^{1}H NMR spectrum of aldehyde **S8** measured in C_6D_6 at 400 MHz.

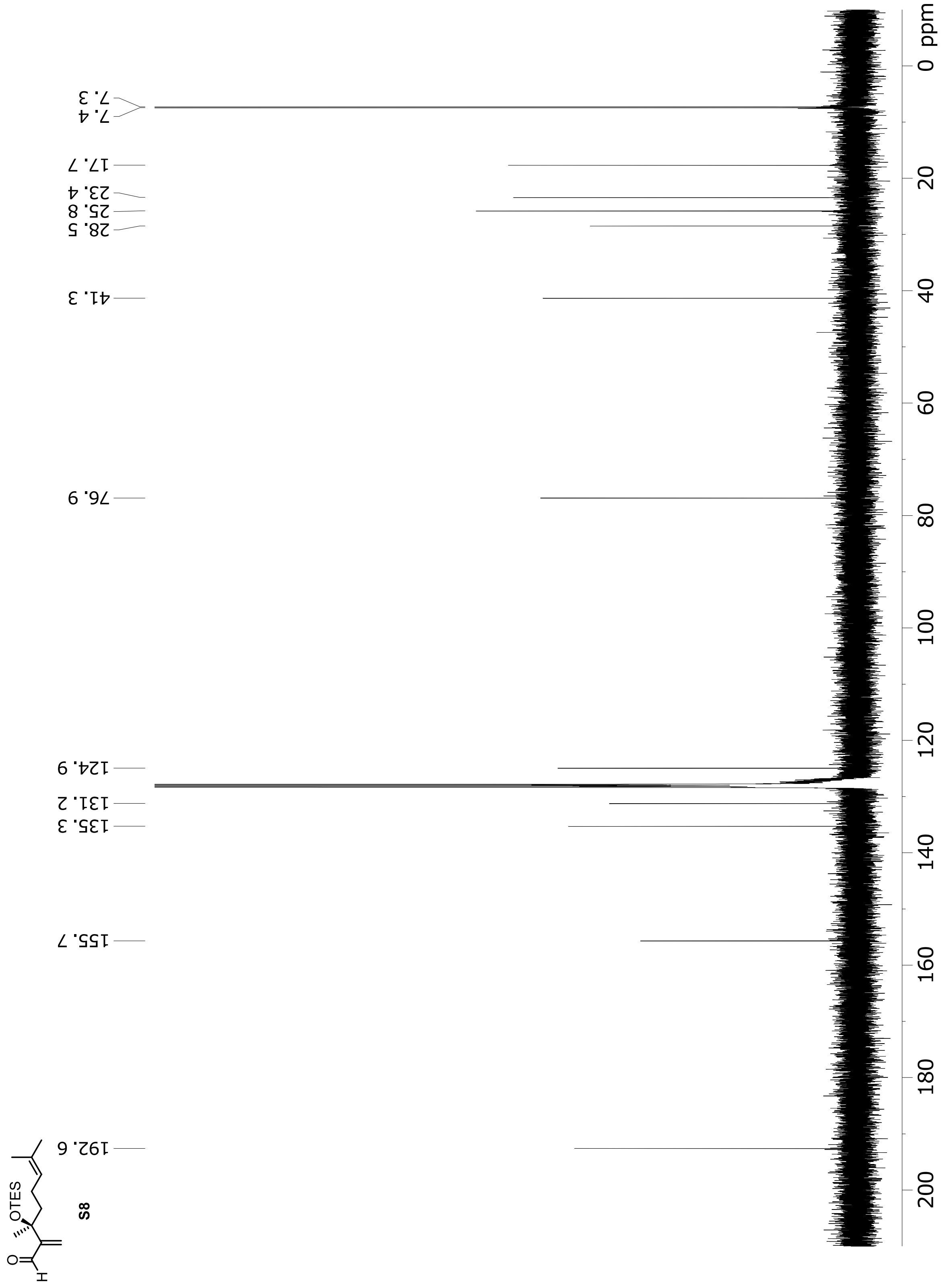

^{13}C NMR spectrum of aldehyde **S8** measured in C_6D_6 at 101 MHz.

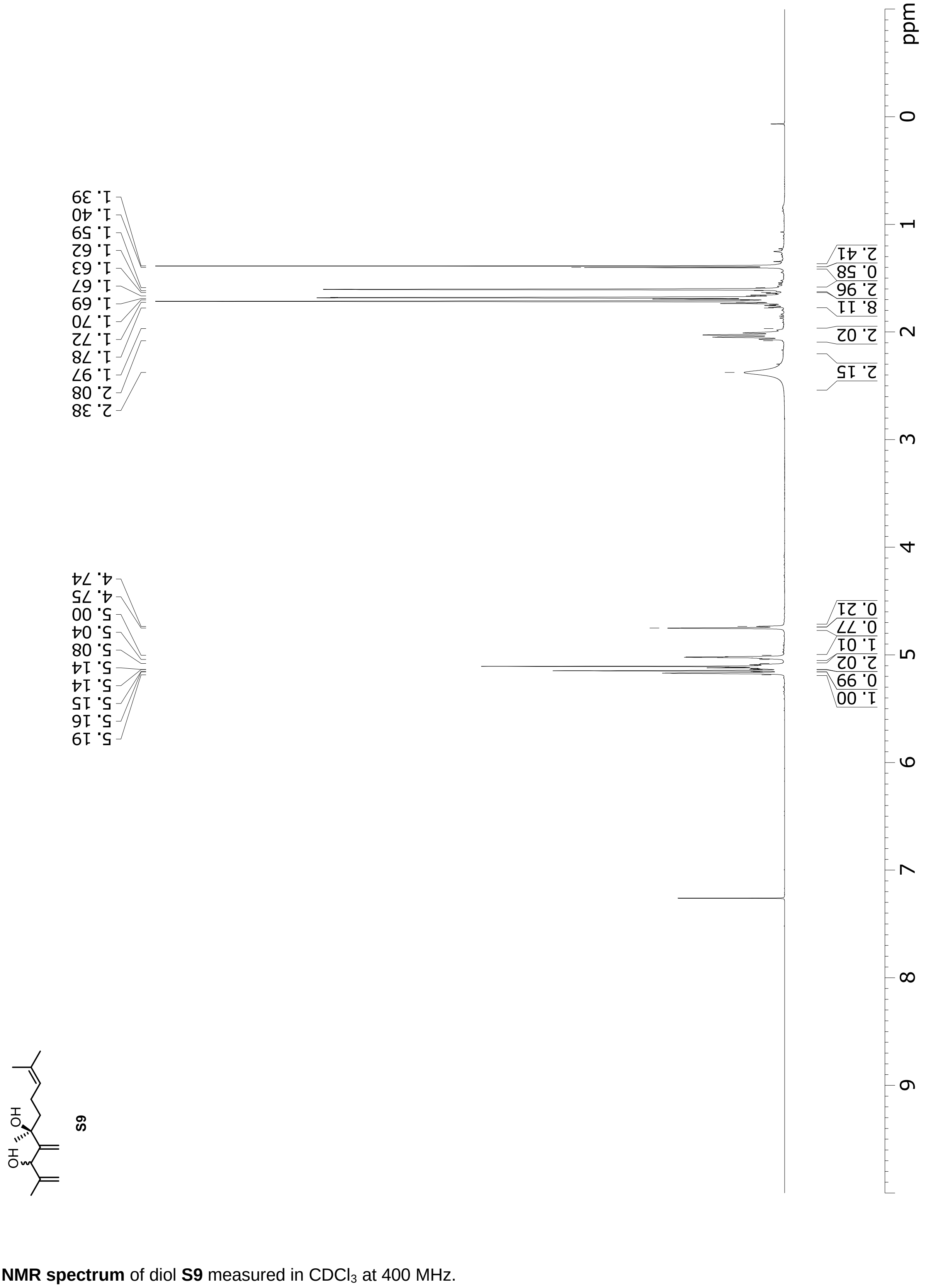

^{1}**H NMR spectrum** of diol **S9** measured in $CDCl_3$ at 400 MHz.

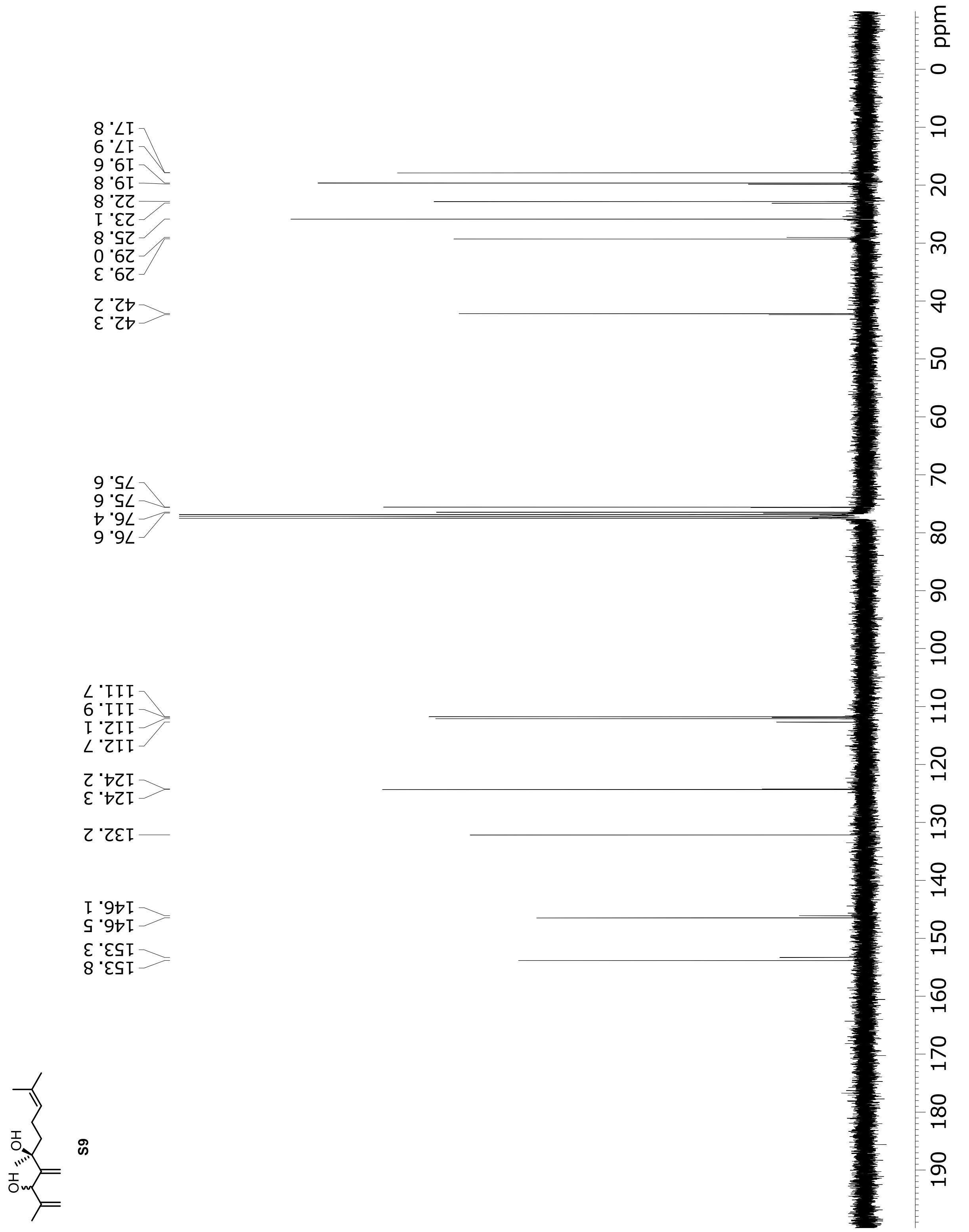

^{13}C NMR spectrum of diol **S9** measured in $CDCl_3$ at 101 MHz.

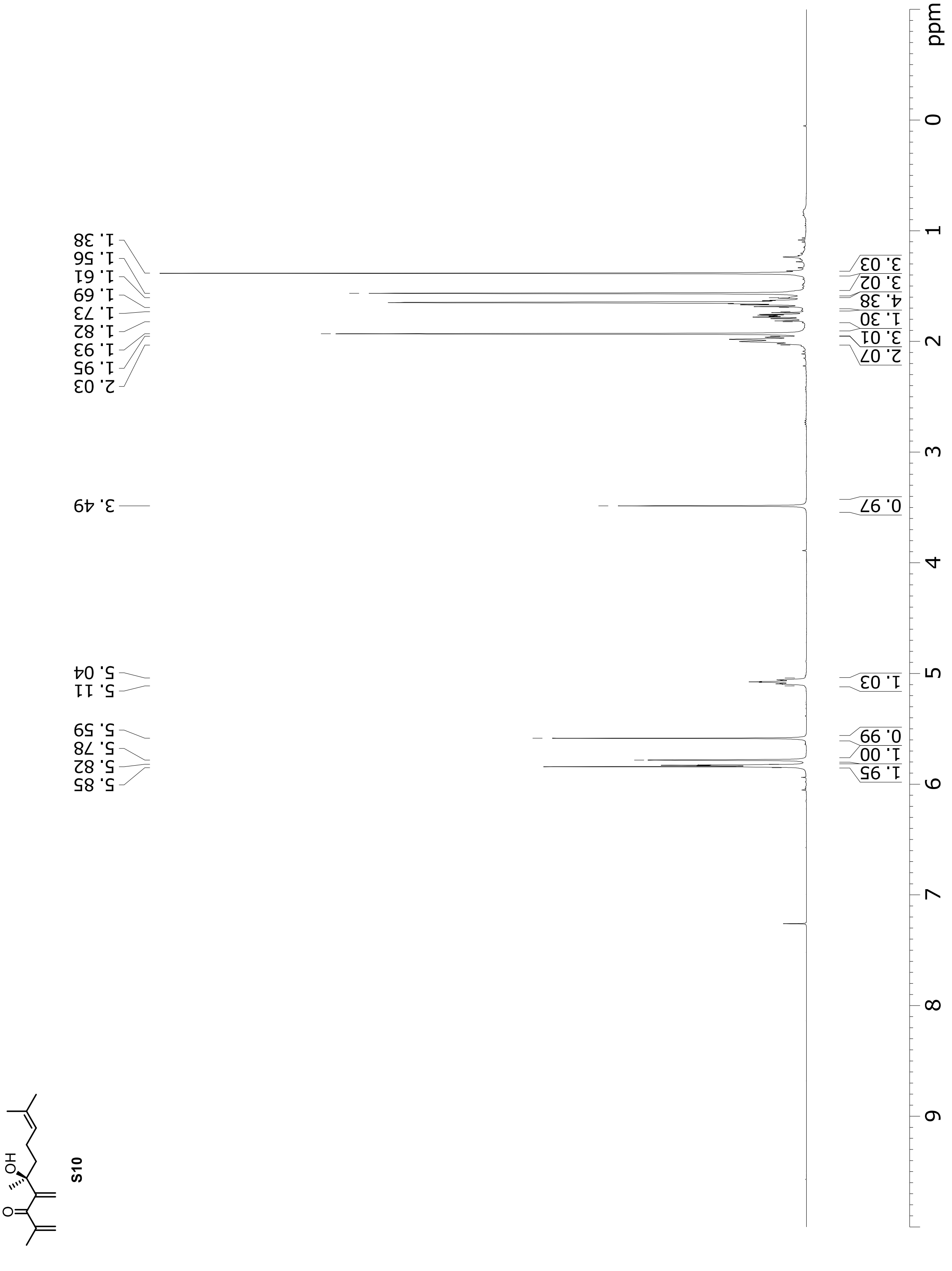

[1]**H NMR spectrum** of cross-conjugated ketone **S10** measured in $CDCl_3$ at 400 MHz.

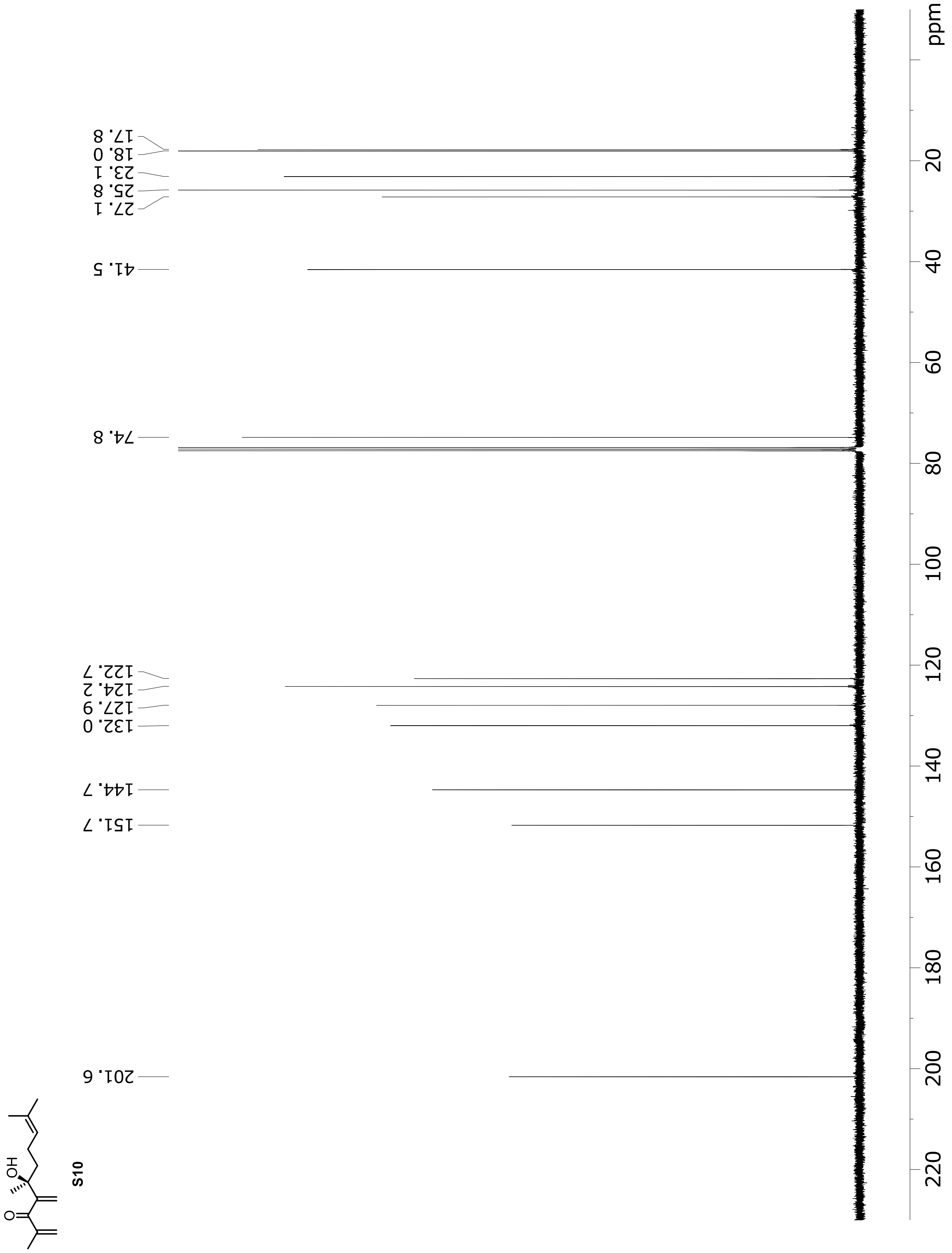

[13]**C NMR spectrum** of cross-conjugated ketone **S10** measured in $CDCl_3$ at 101 MHz.

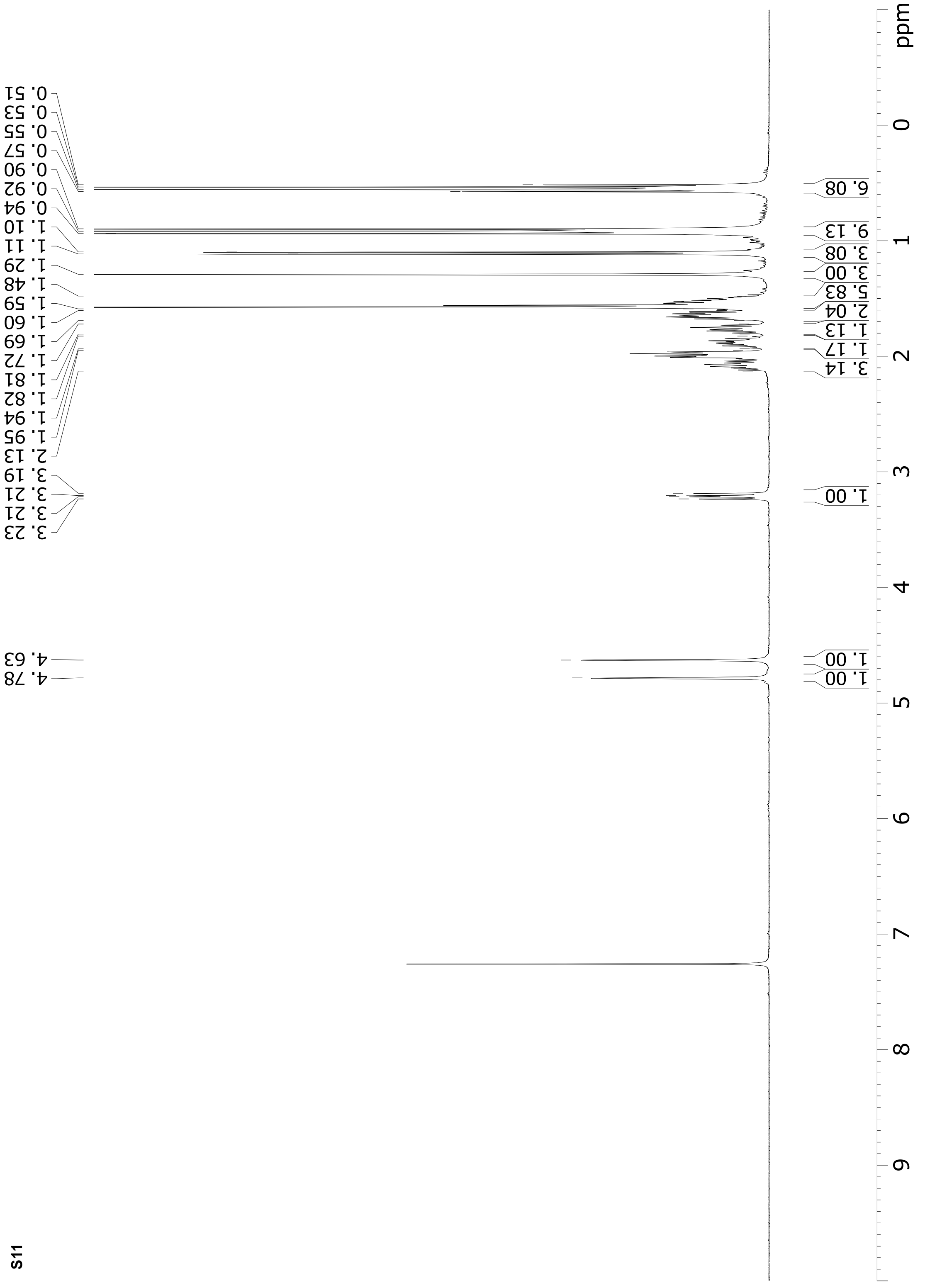

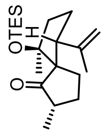

S11

[1]**H NMR spectrum** of TES-protected spiroketone **S11** measured in $CDCl_3$ at 400 MHz.

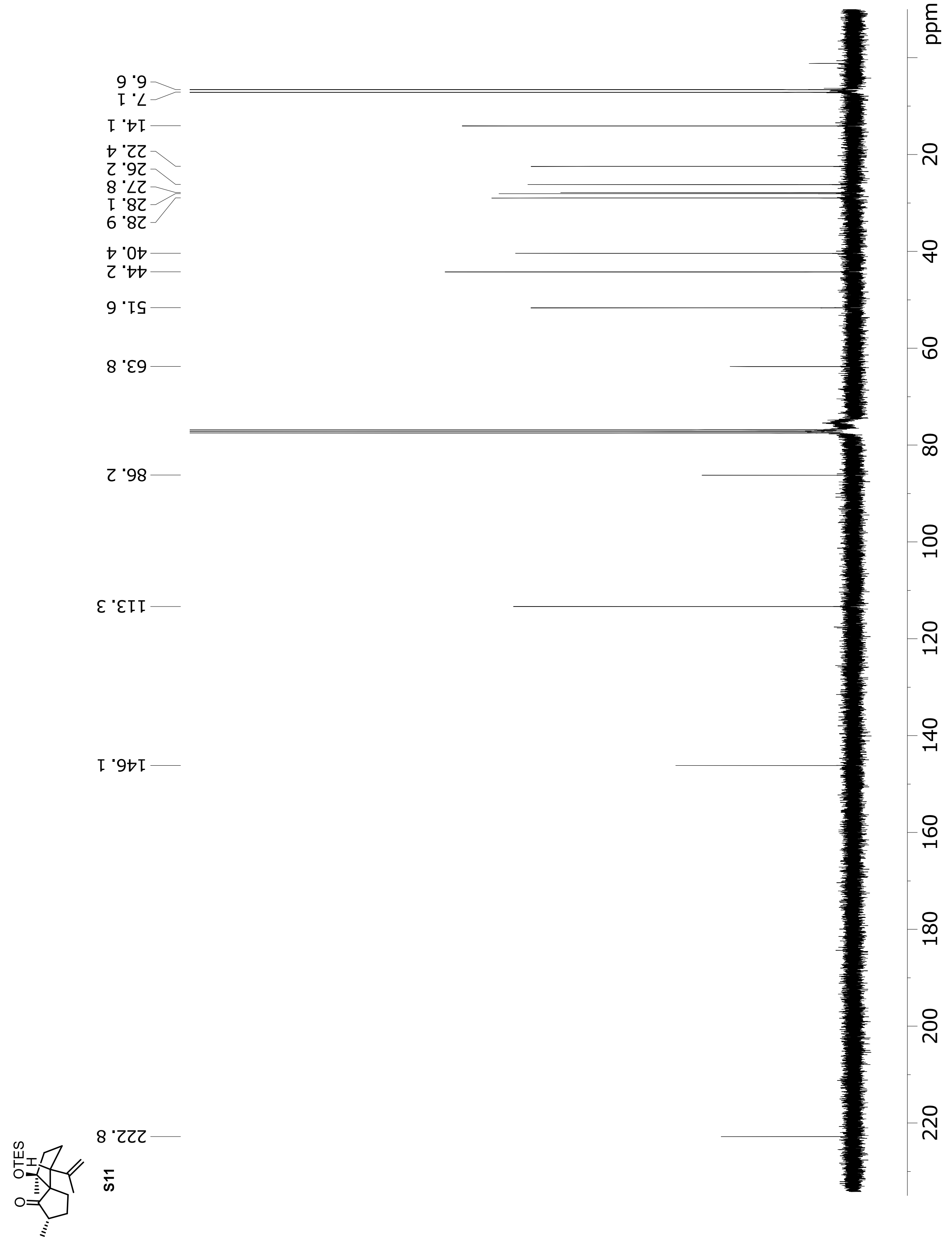

^{13}C NMR spectrum of TES-protected spiroketone **S11** measured in $CDCl_3$ at 101 MHz.

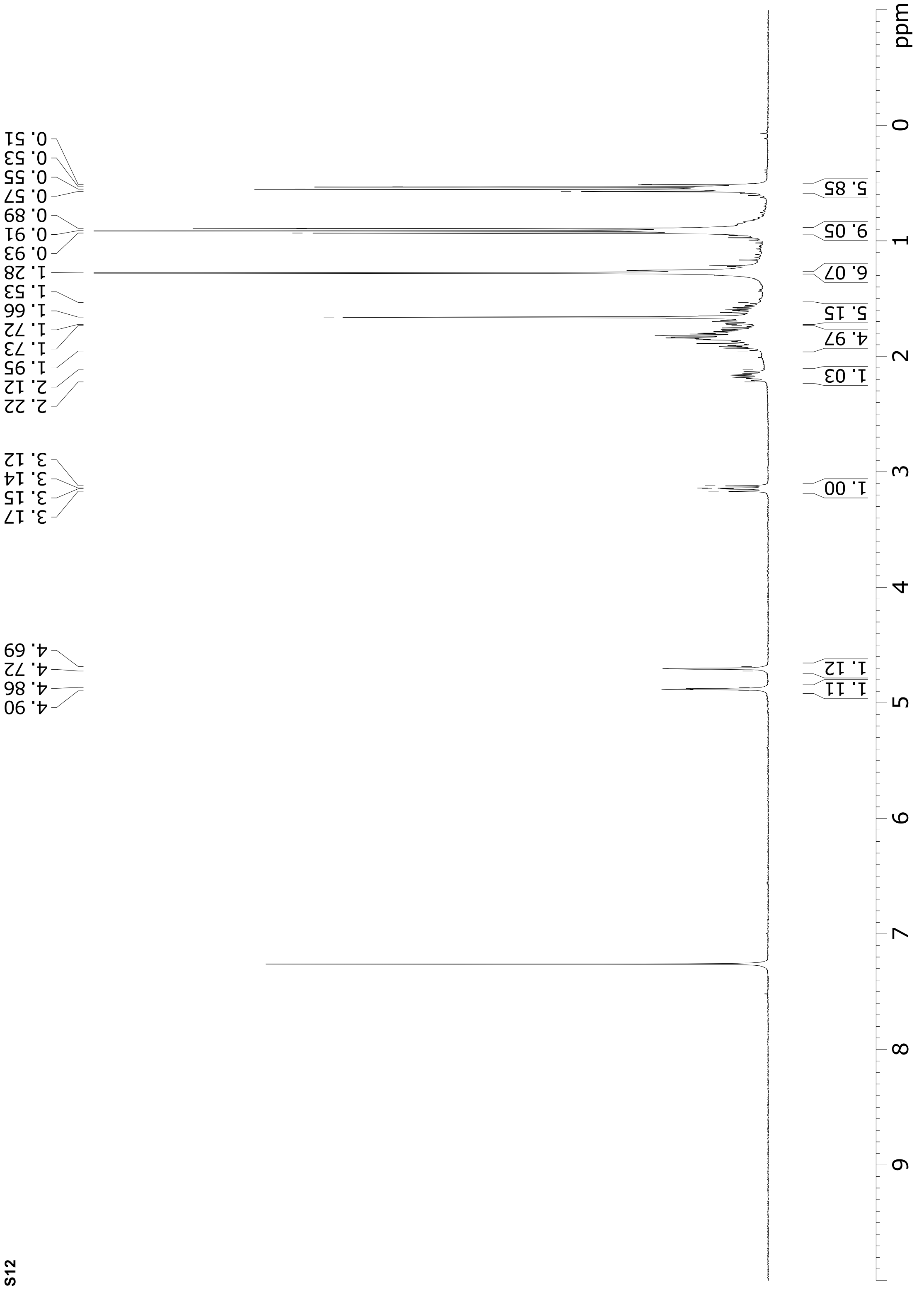

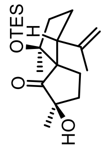

S12

^{1}**H NMR spectrum** of α-hydroxy ketone **S12** measured in $CDCl_3$ at 400 MHz.

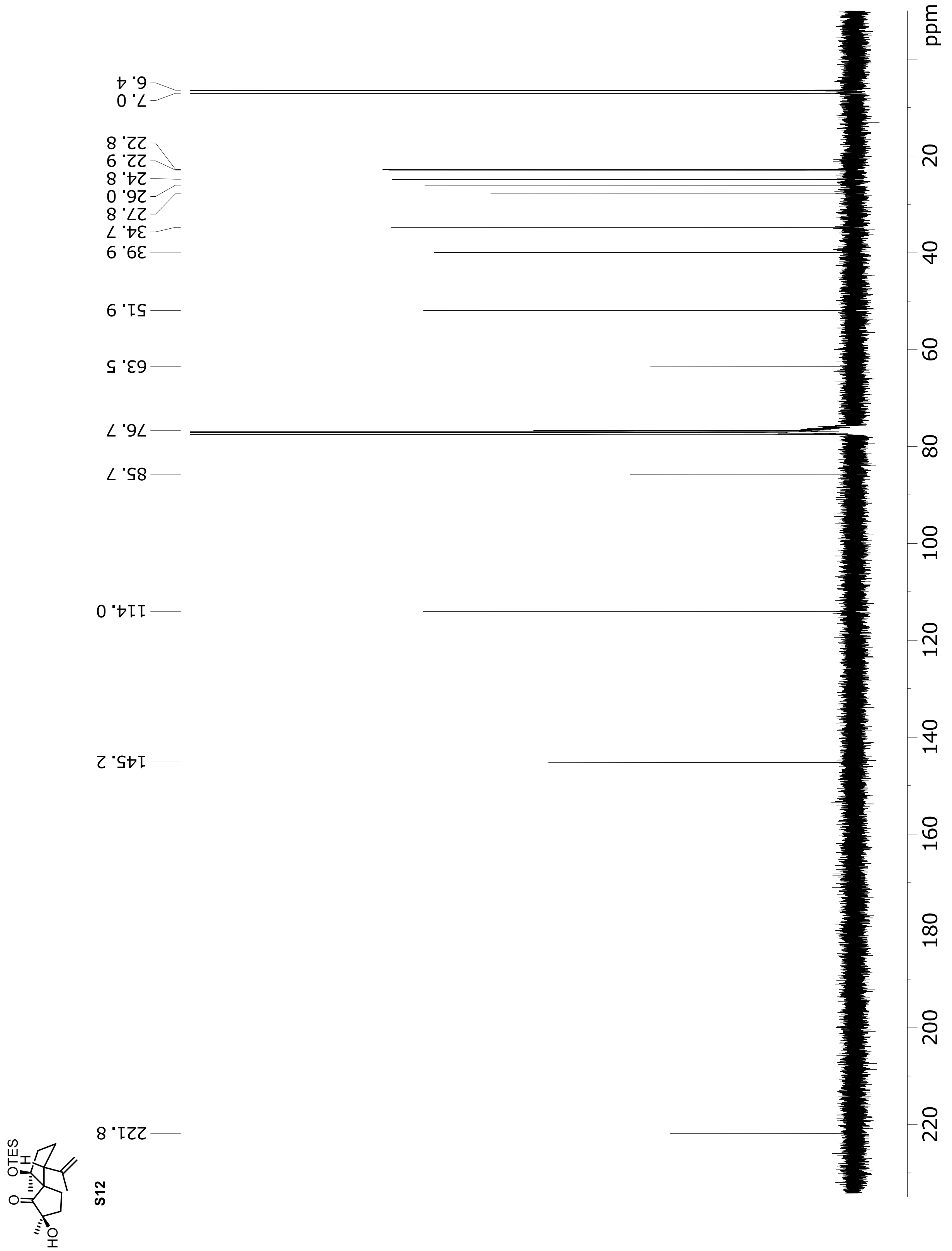

^{13}C NMR spectrum of α-hydroxy ketone **S12** measured in $CDCl_3$ at 101 MHz.

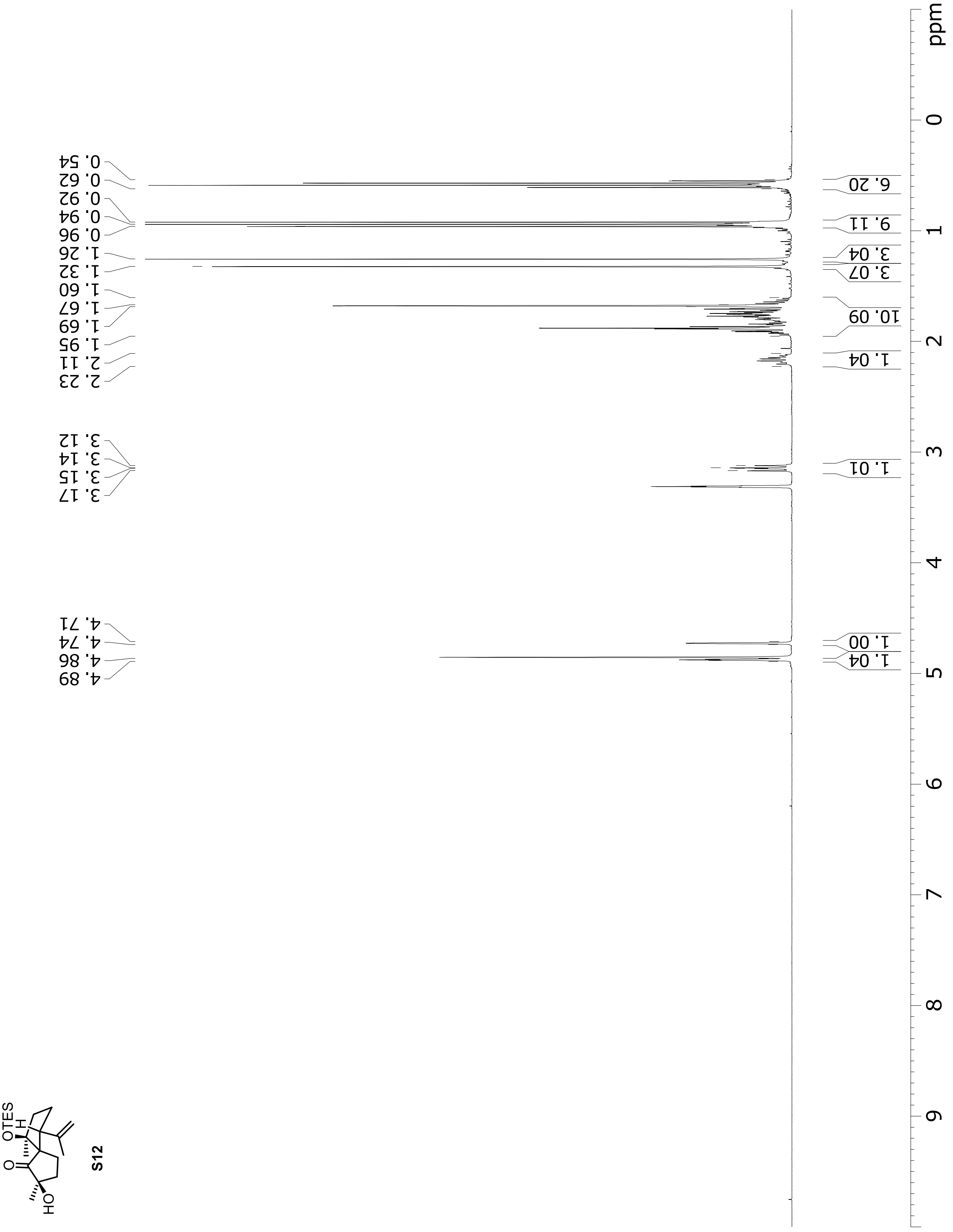

¹H NMR spectrum of α-hydroxy ketone **S12** measured in methanol-d_4 at 400 MHz

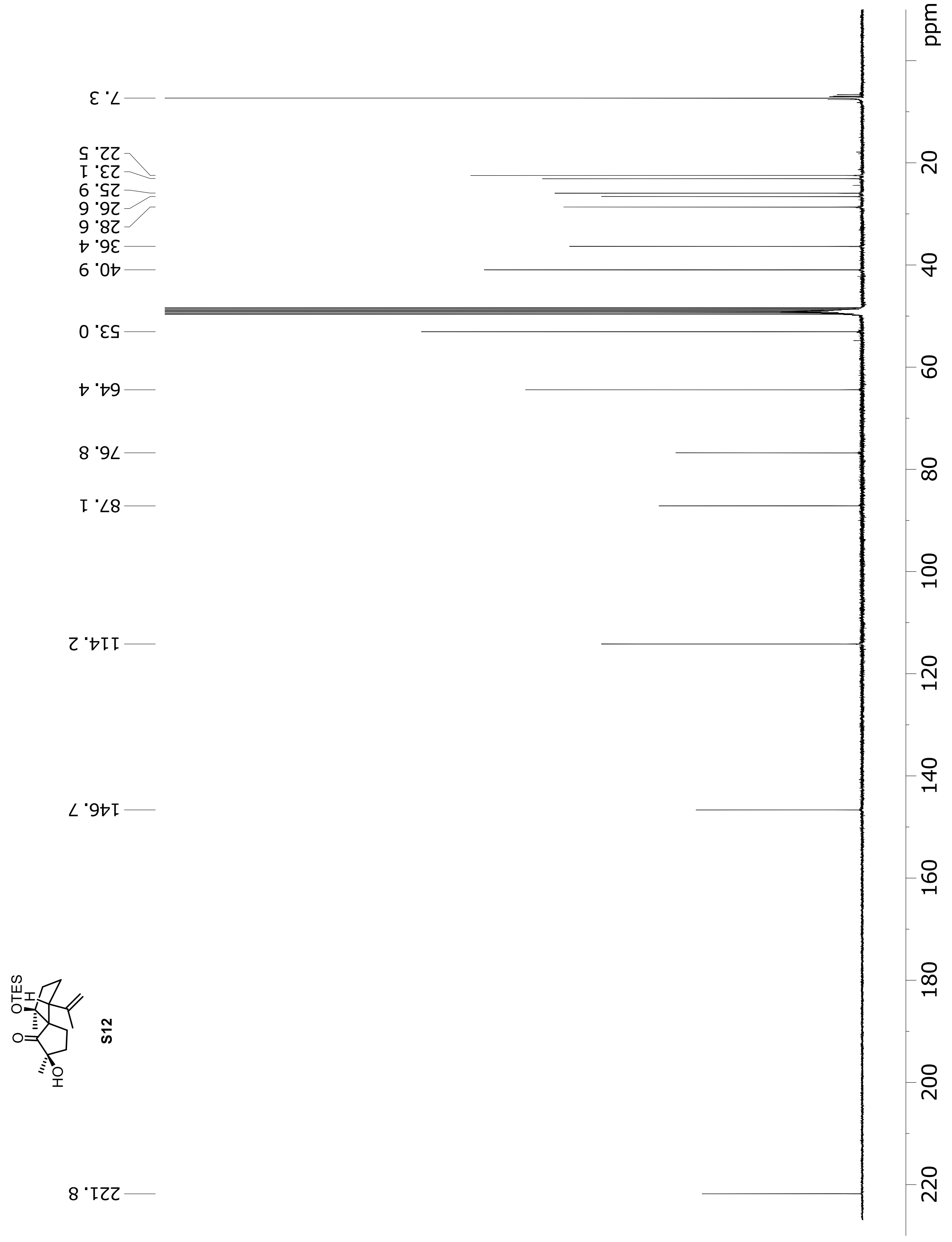

^{13}**C NMR spectrum** of α-hydroxy ketone **S12** measured in methanol-d_4 at 101 MHz.

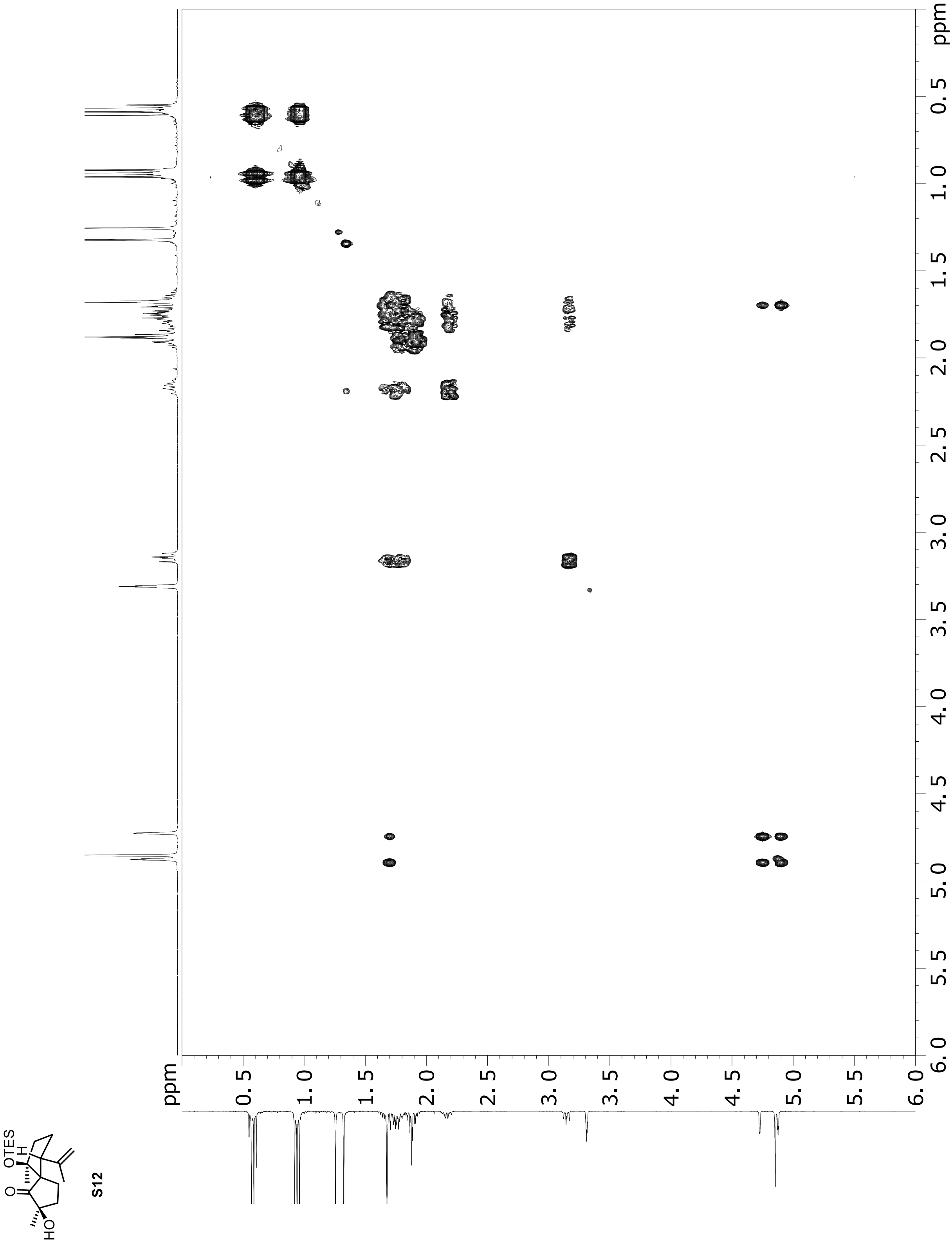

COSY NMR spectrum of α-hydroxy ketone **S12** measured in methanol-d_4 at 400 MHz.

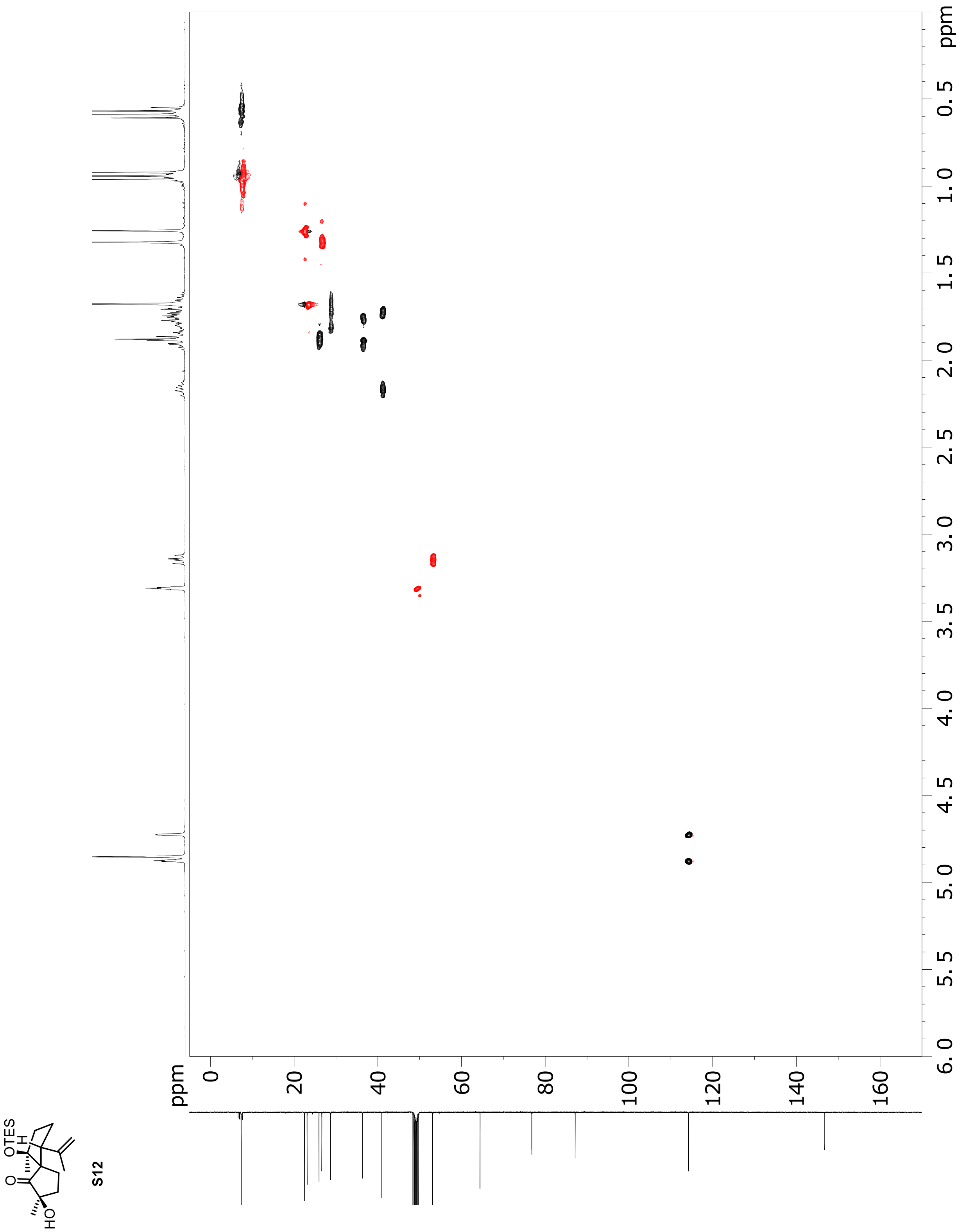

HSQC NMR spectrum of α-hydroxy ketone **S12** measured in methanol-d_4 at 400 MHz.

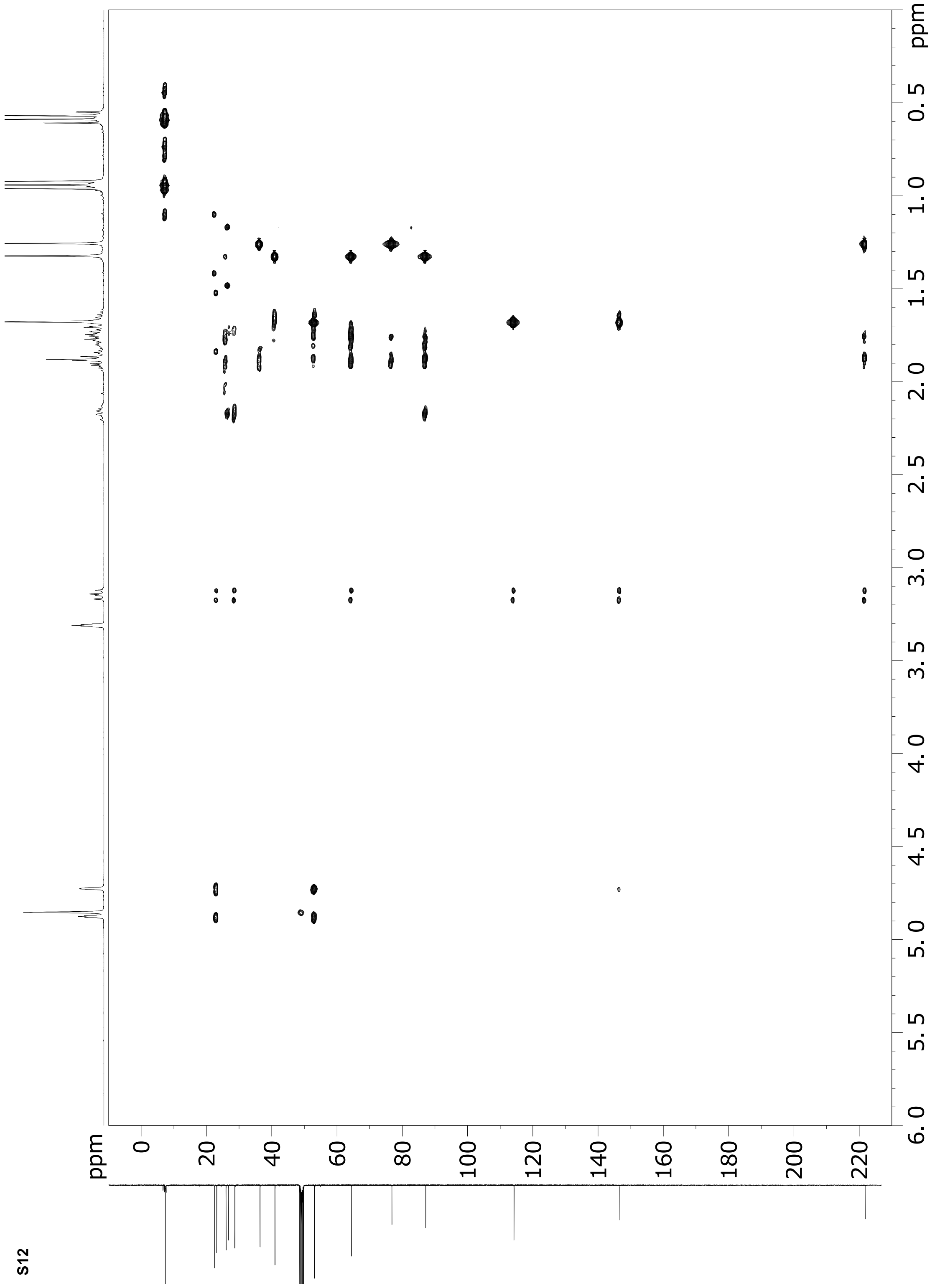

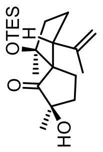

S12

HMBC NMR spectrum of α-hydroxy ketone **S12** measured in methanol-d_4 at 400 MHz.

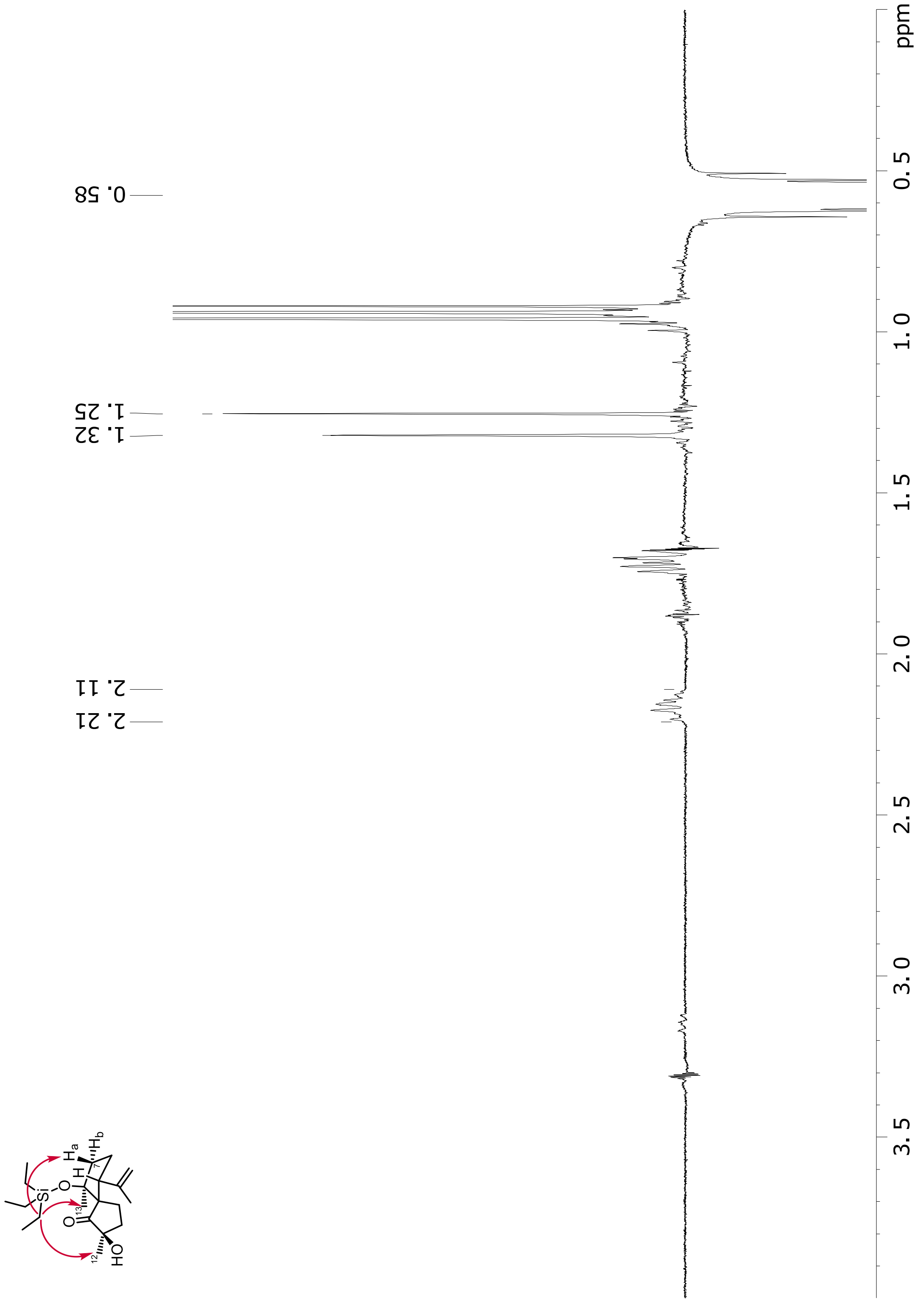

^{1}H-NOE spectrum of α-hydroxy ketone **S12** after irradiation at 0.58 ppm (TES, CH_2), measured in in methanol-d_4 at 400 MHz

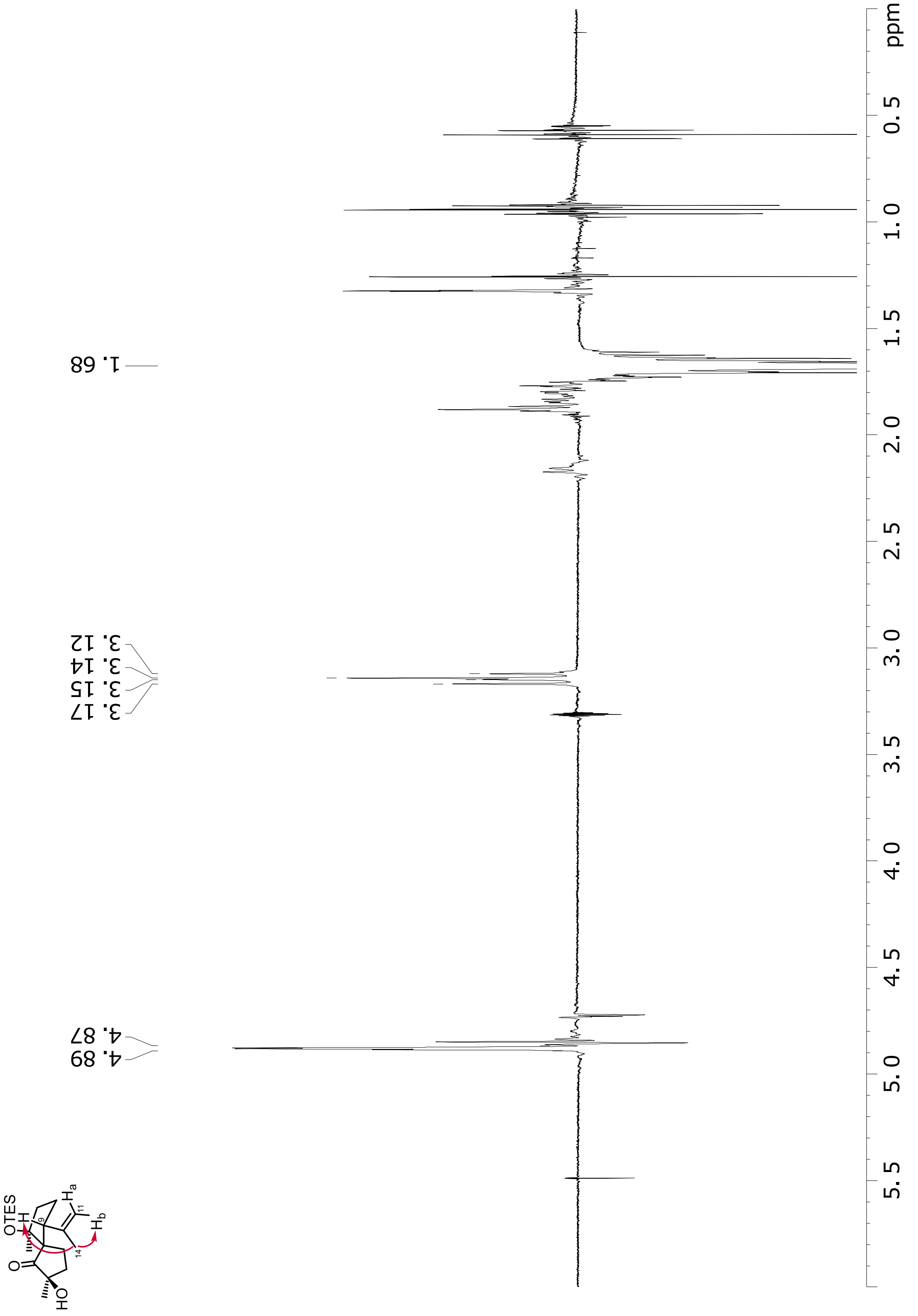

^{1}H-NOE spectrum of α-hydroxy ketone **S12** after irradiation at 1.68 ppm (CH_3-14), measured in in methanol-d_4 at 400 MHz

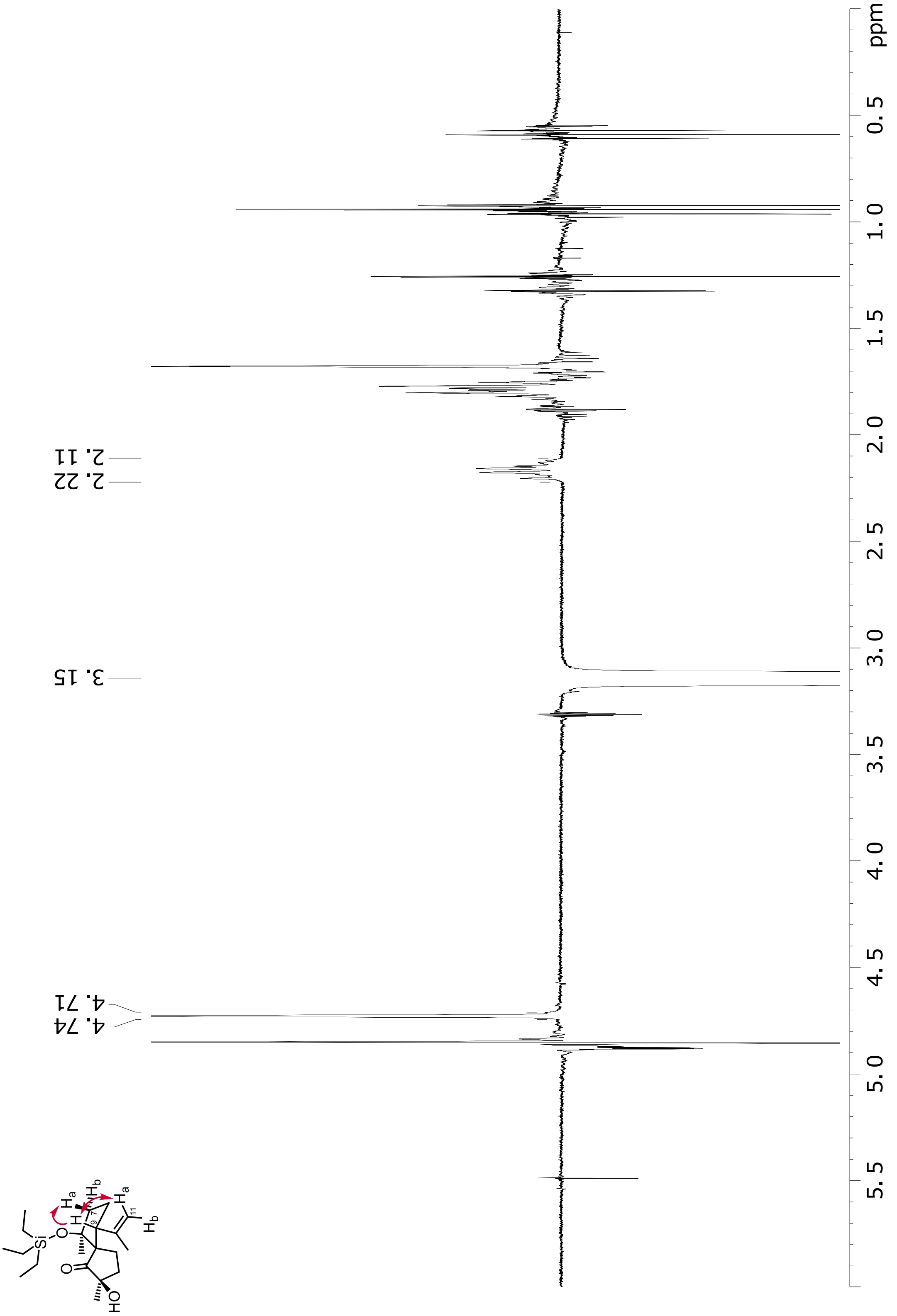

[1]**H-NOE spectrum** of α-hydroxy ketone **S12** after irradiation at 3.15 ppm (CH-9), measured in in methanol-d_4 at 400 MHz

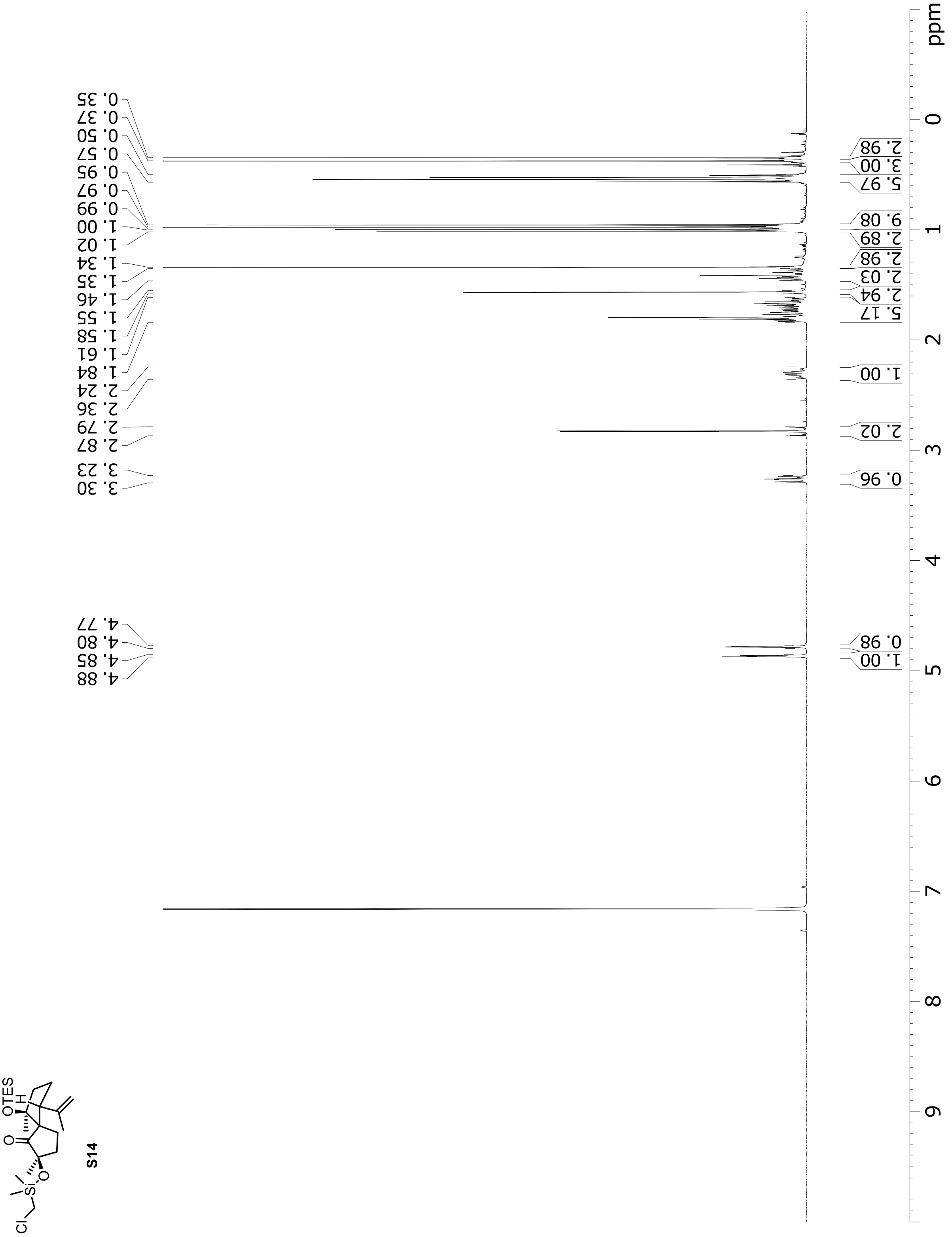

^{1}H NMR spectrum of silyl ether **S14** measured in C_6D_6 at 400 MHz.

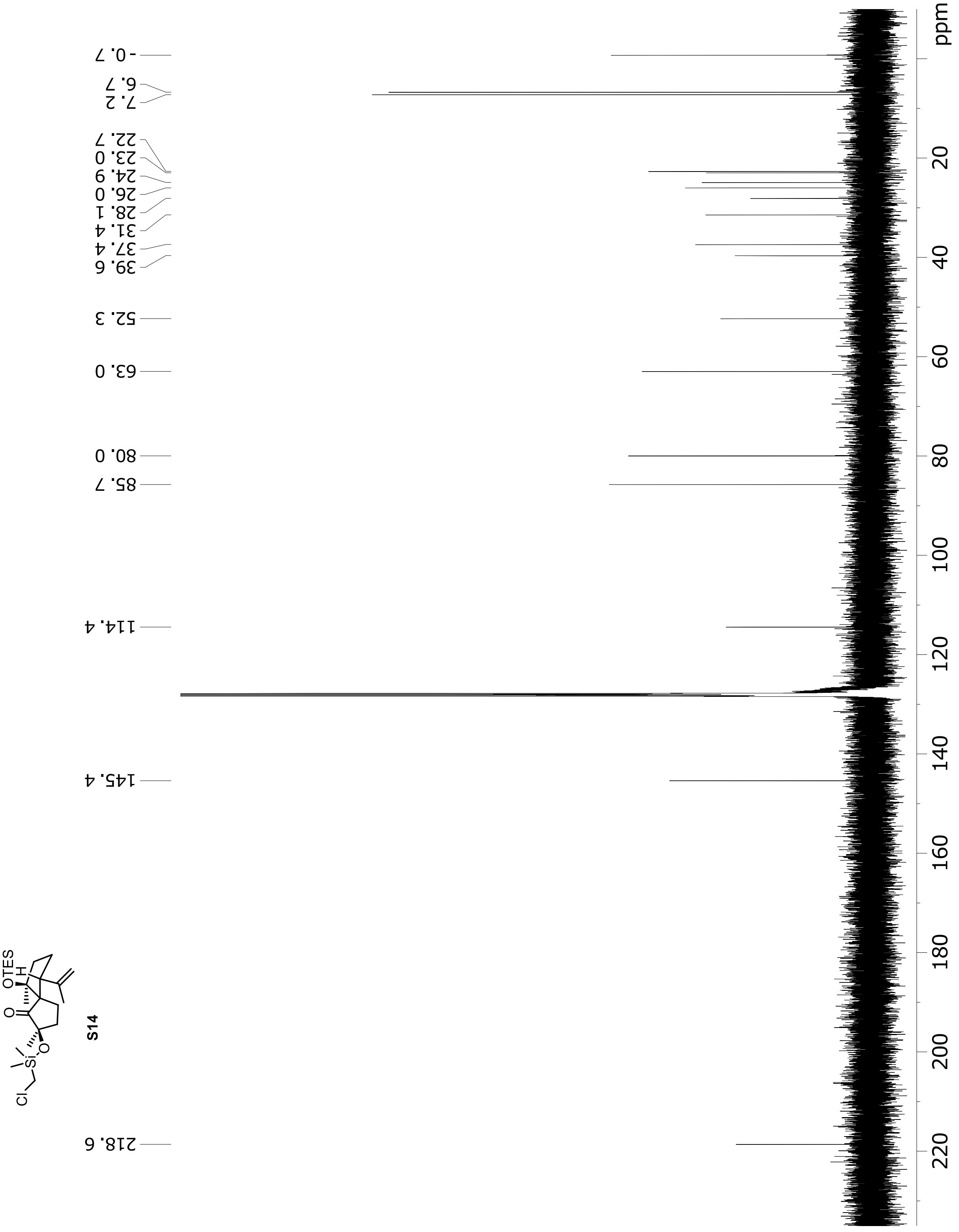

^{13}**C NMR spectrum** of silyl ether **S14** measured in C_6D_6 at 101 MHz.

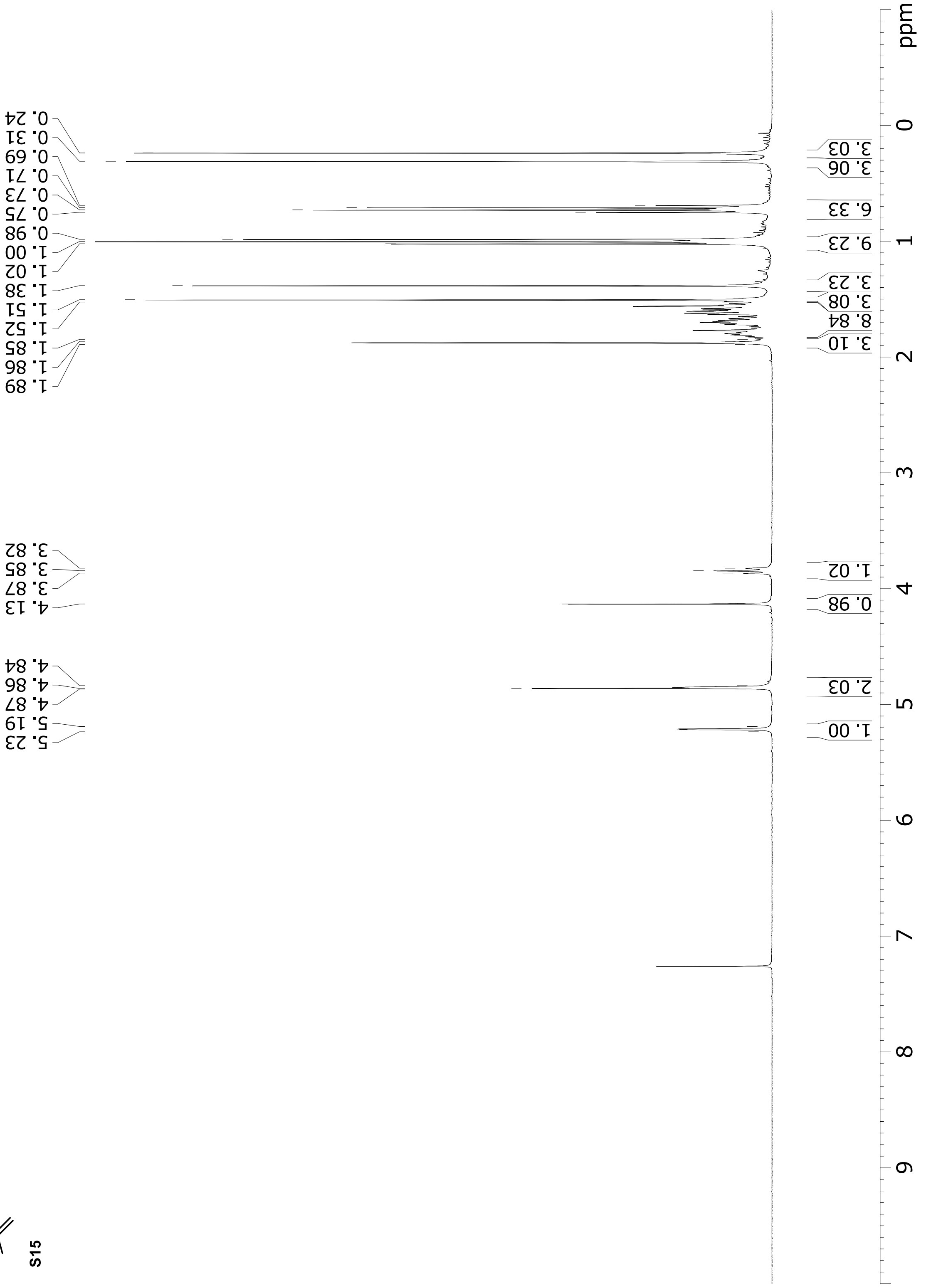

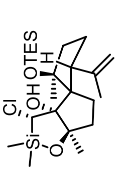

S15

[1]**H NMR spectrum** of oxasilolane **S15** measured in $CDCl_3$ at 400 MHz.

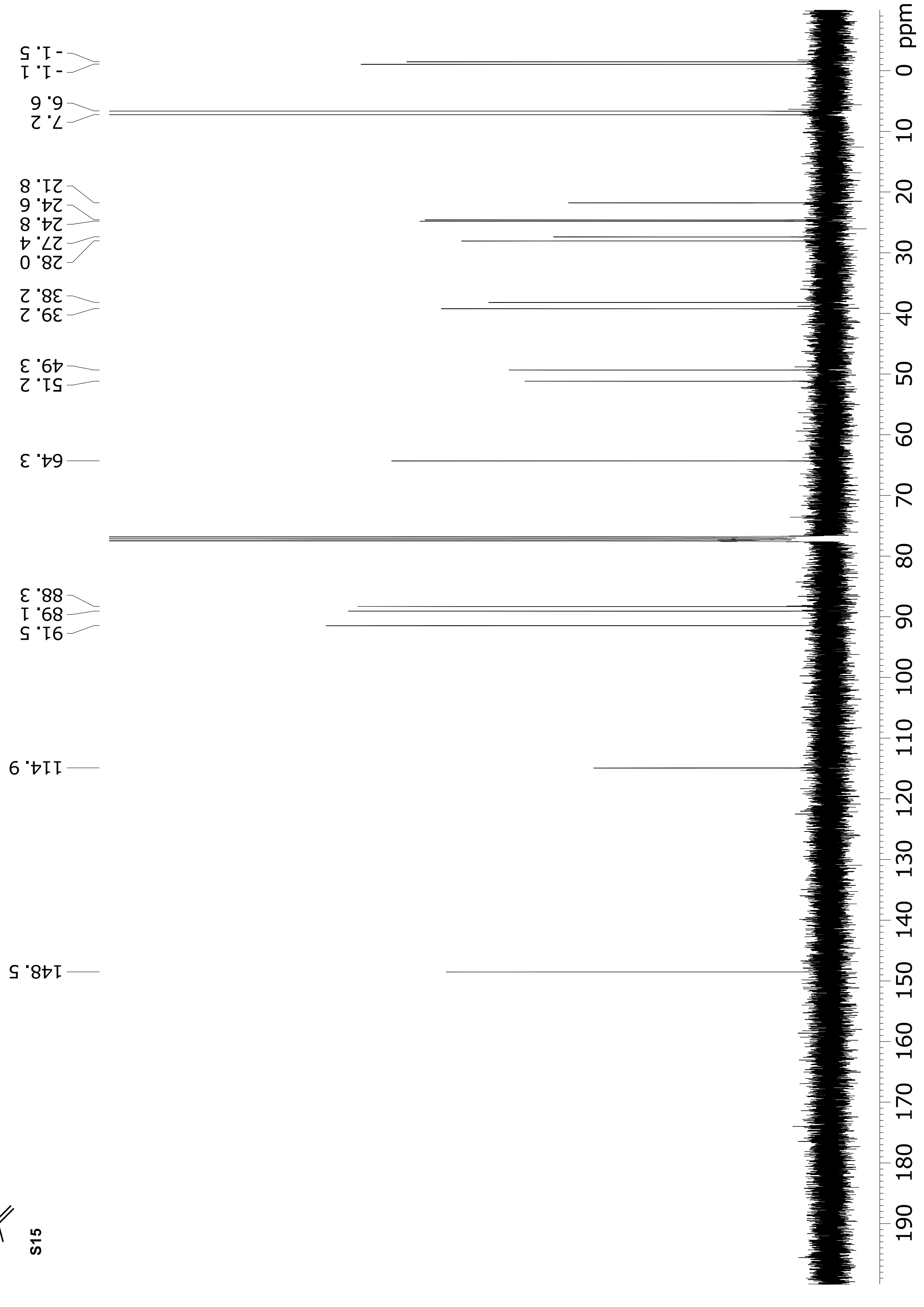

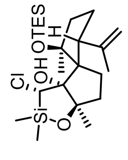

S15

^{13}C NMR spectrum of oxasilolane **S15** measured in $CDCl_3$ at 101 MHz.

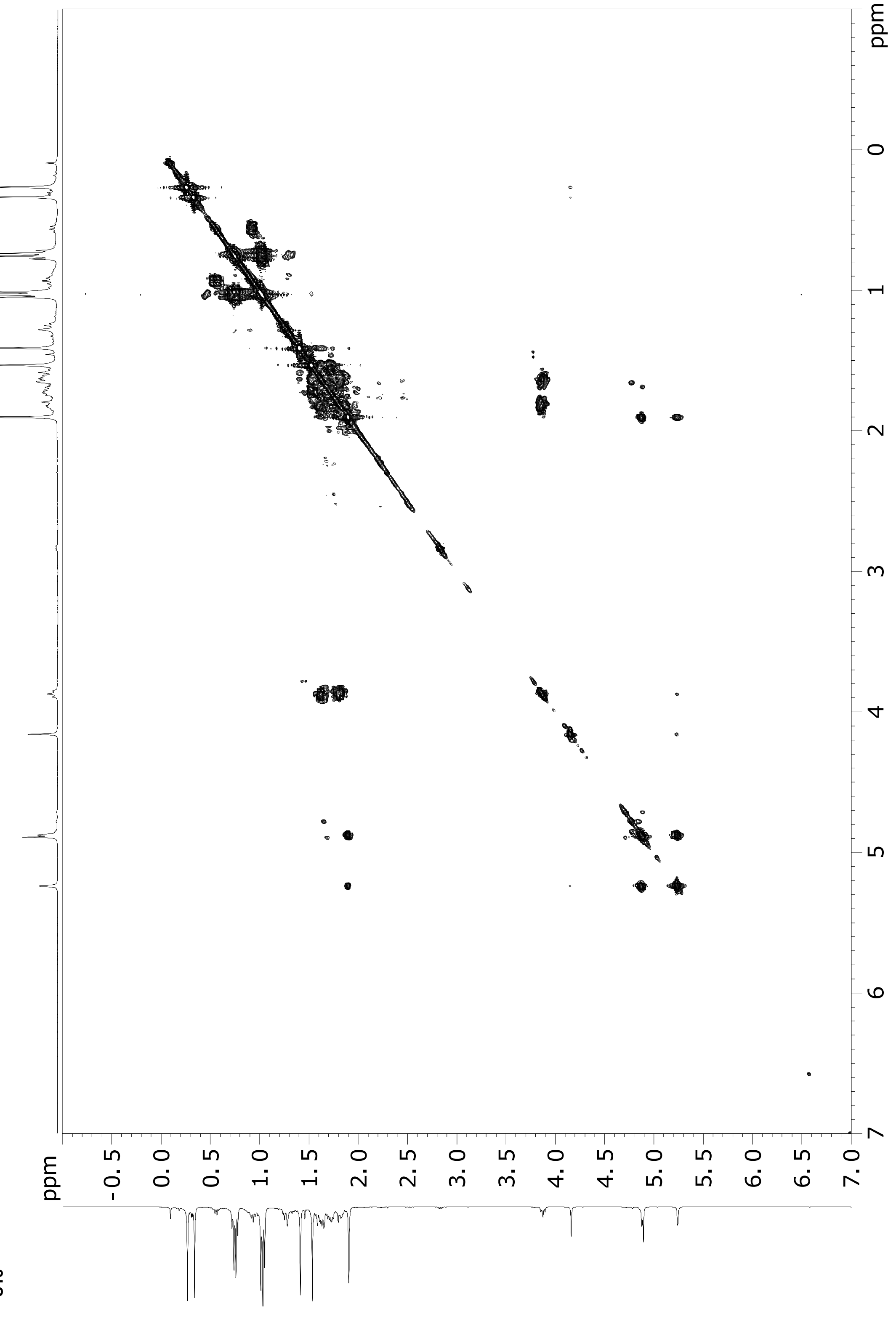

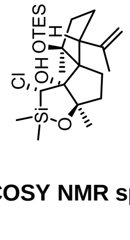

S15

COSY NMR spectrum of oxasilolane **S15** measured in $CDCl_3$ at 400 MHz.

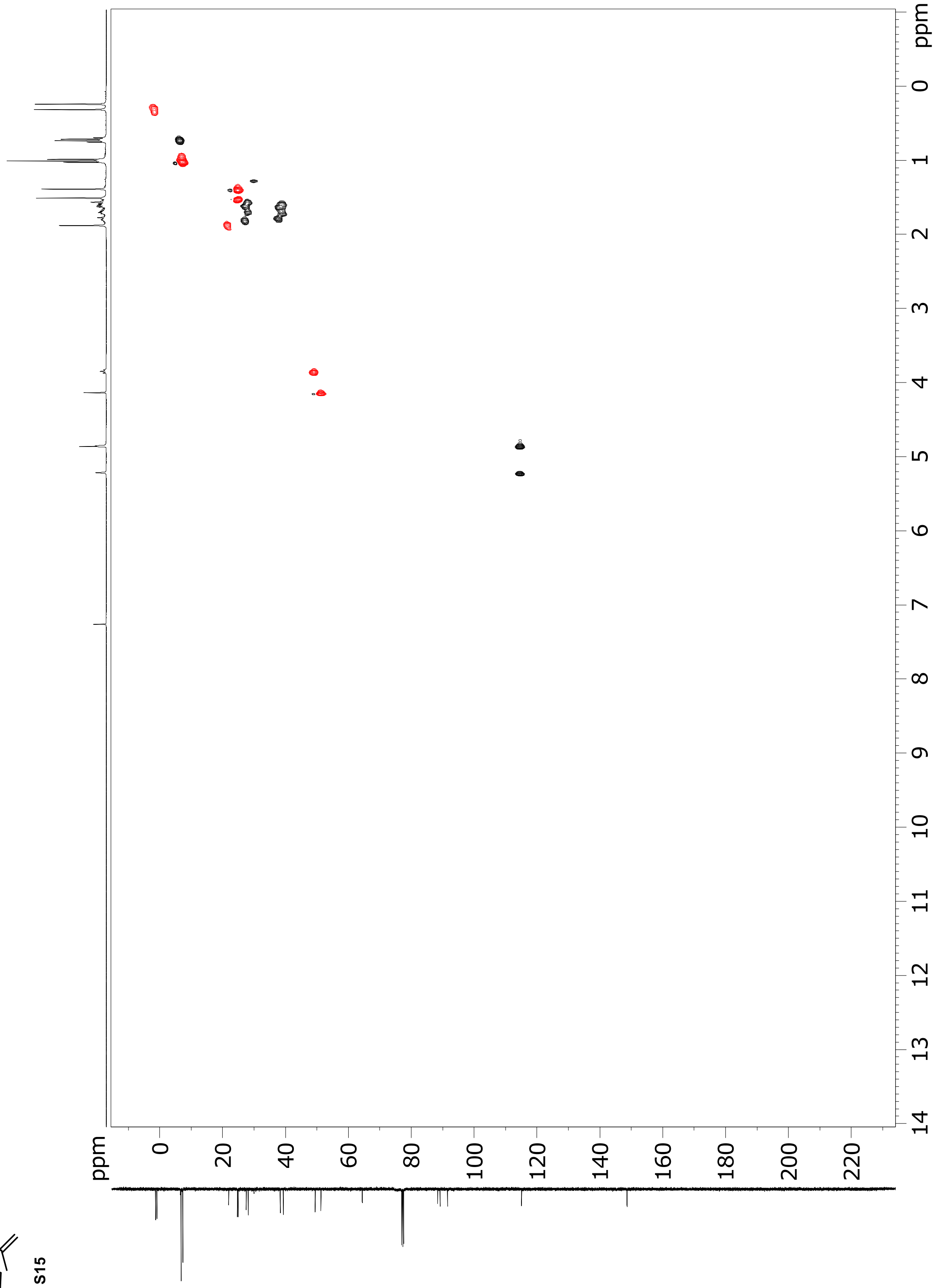

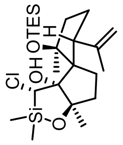

S15

HSQC NMR spectrum of oxasilolane **S15** measured in $CDCl_3$ at 400 MHz.

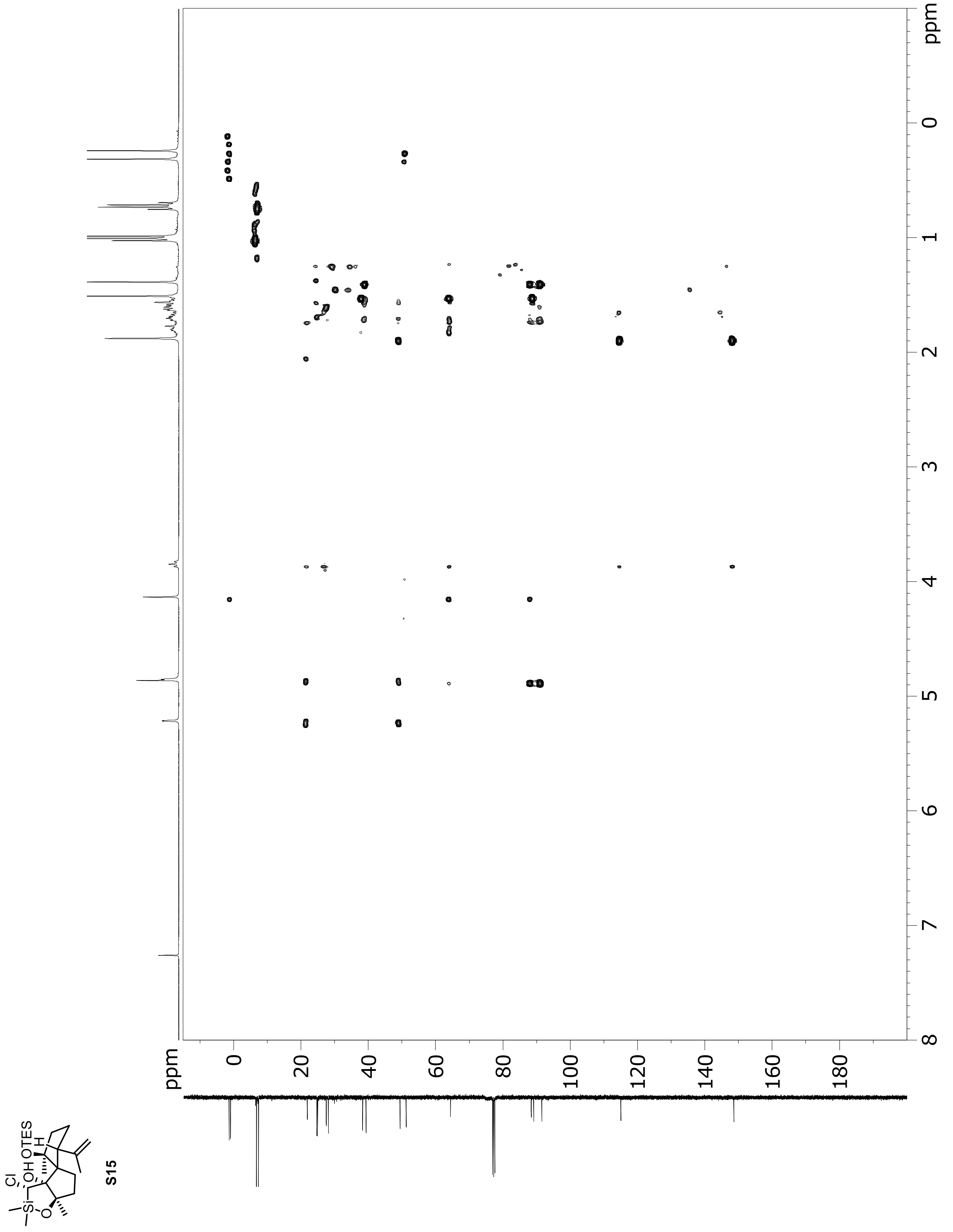

HMBC NMR spectrum of oxasilolane **S15** measured in $CDCl_3$ at 400 MHz.

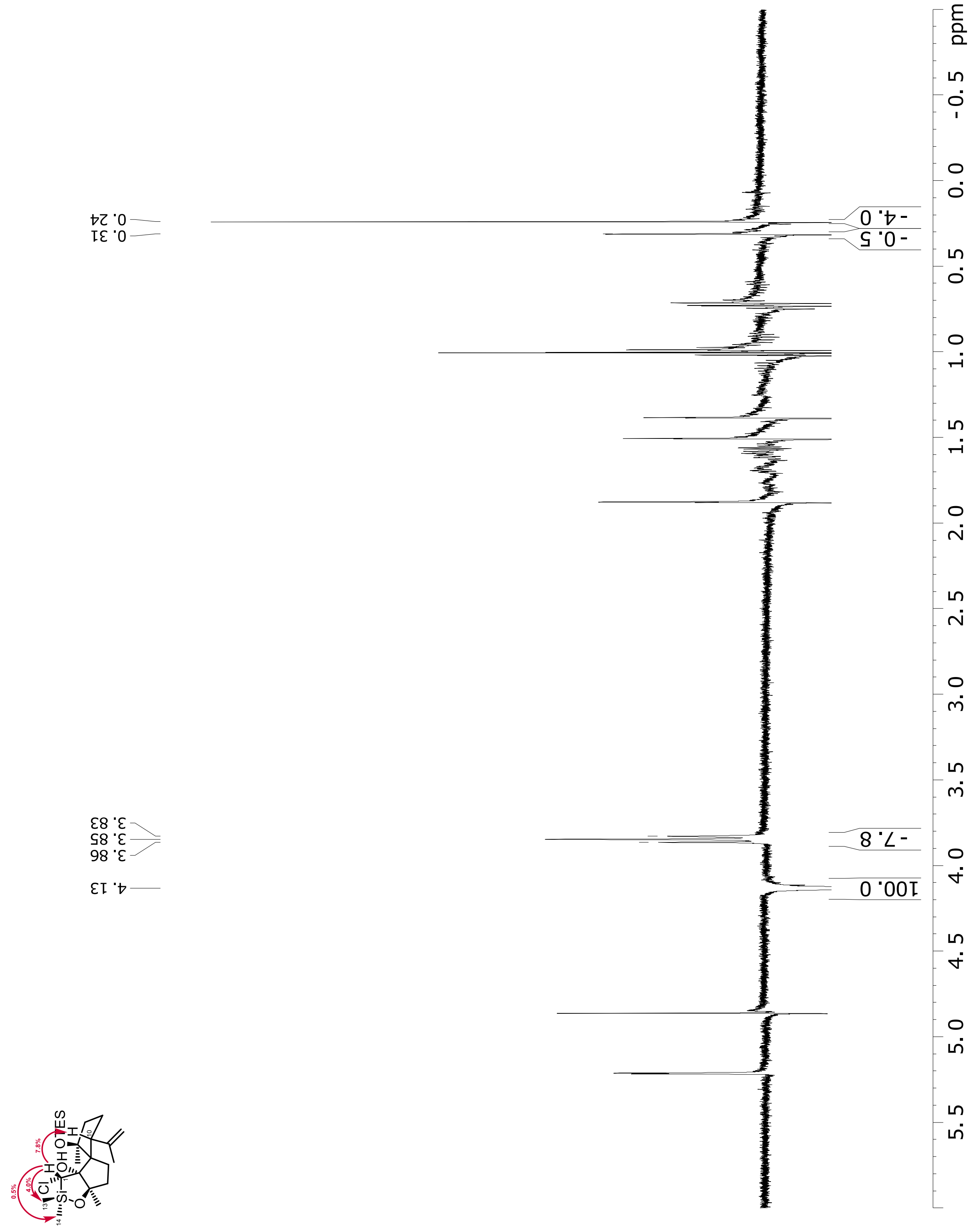

^{1}H-NOE spectrum of oxasilolane **S15** after irradiation at 4.13 ppm (CH-1), measured in $CDCl_3$ at 500 MHz

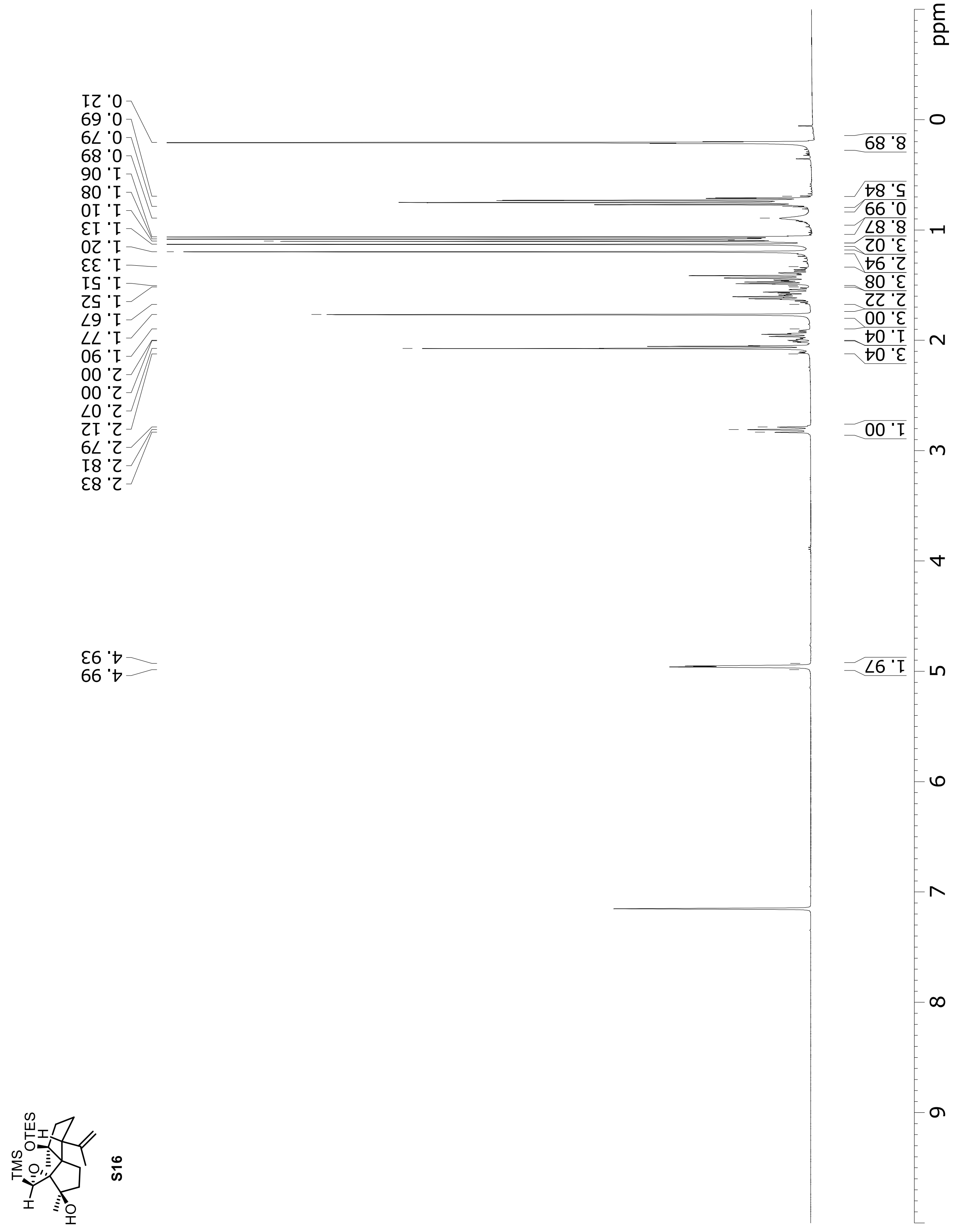

1H NMR spectrum of TMS-epoxide **S16** measured in C_6D_6 at 400 MHz.

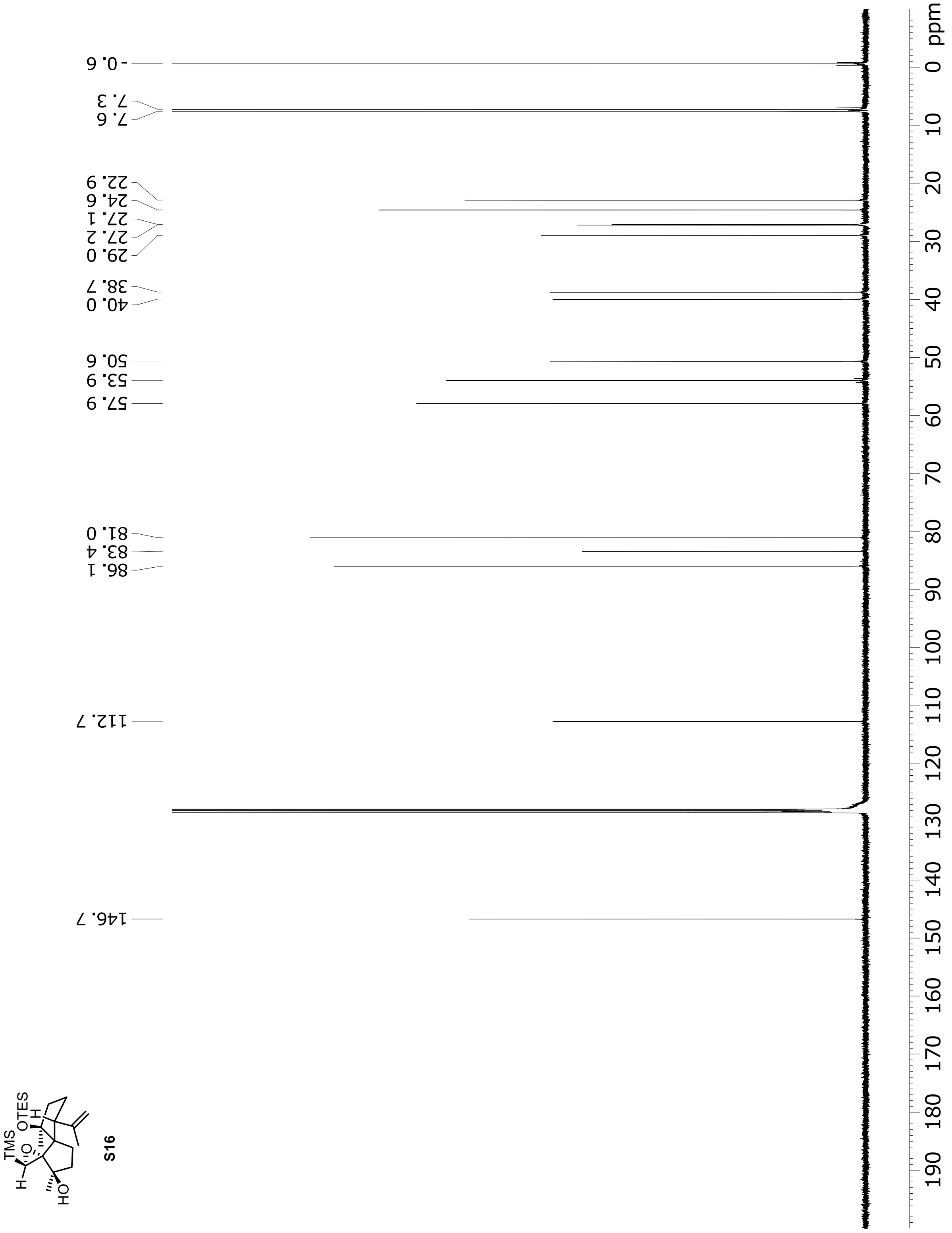

^{13}C NMR spectrum of TMS-epoxide **S16** measured in C_6D_6 at 101 MHz.

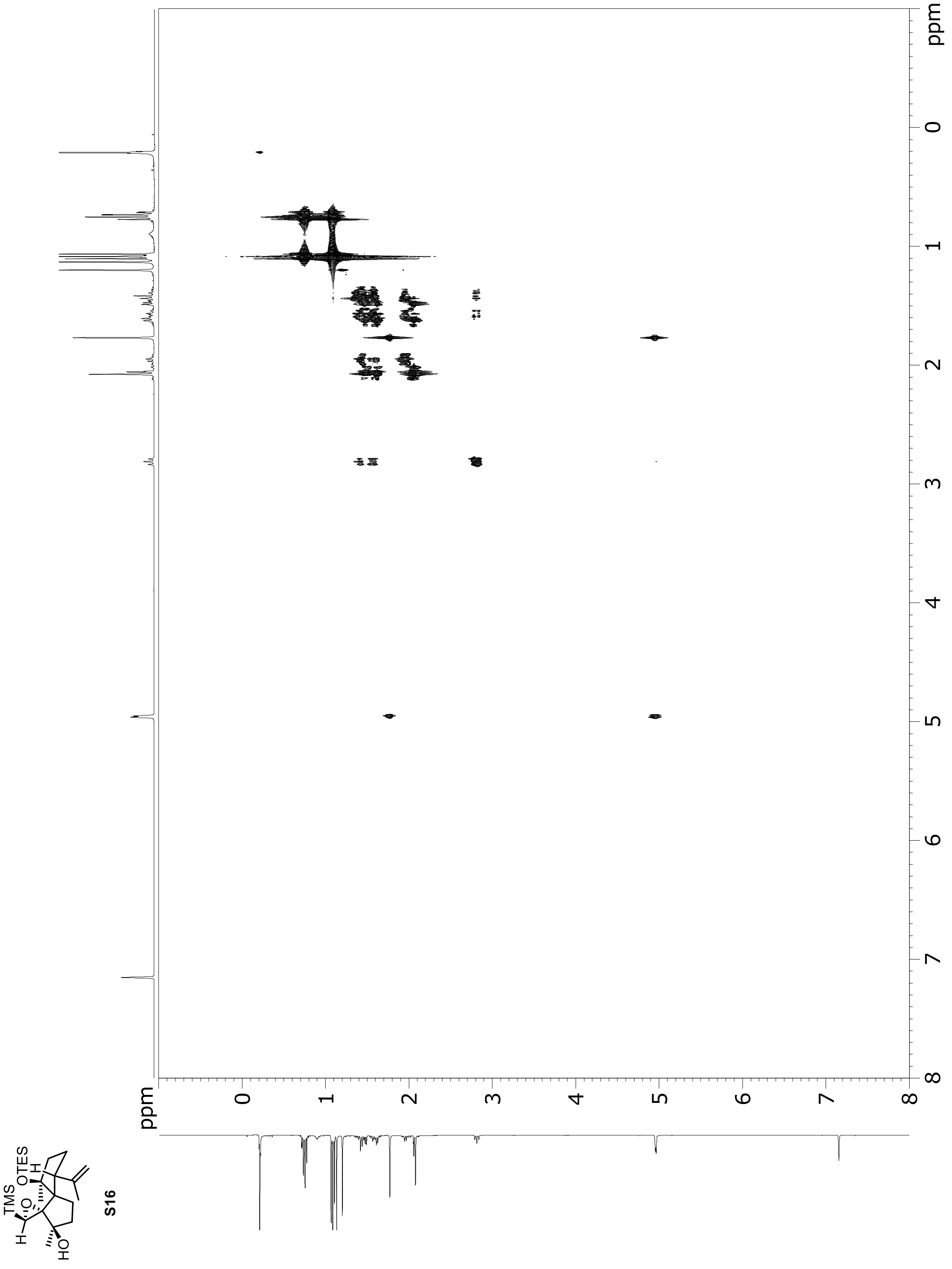

COSY NMR spectrum of TMS-epoxide **S16** measured in C_6D_6 at 400 MHz.

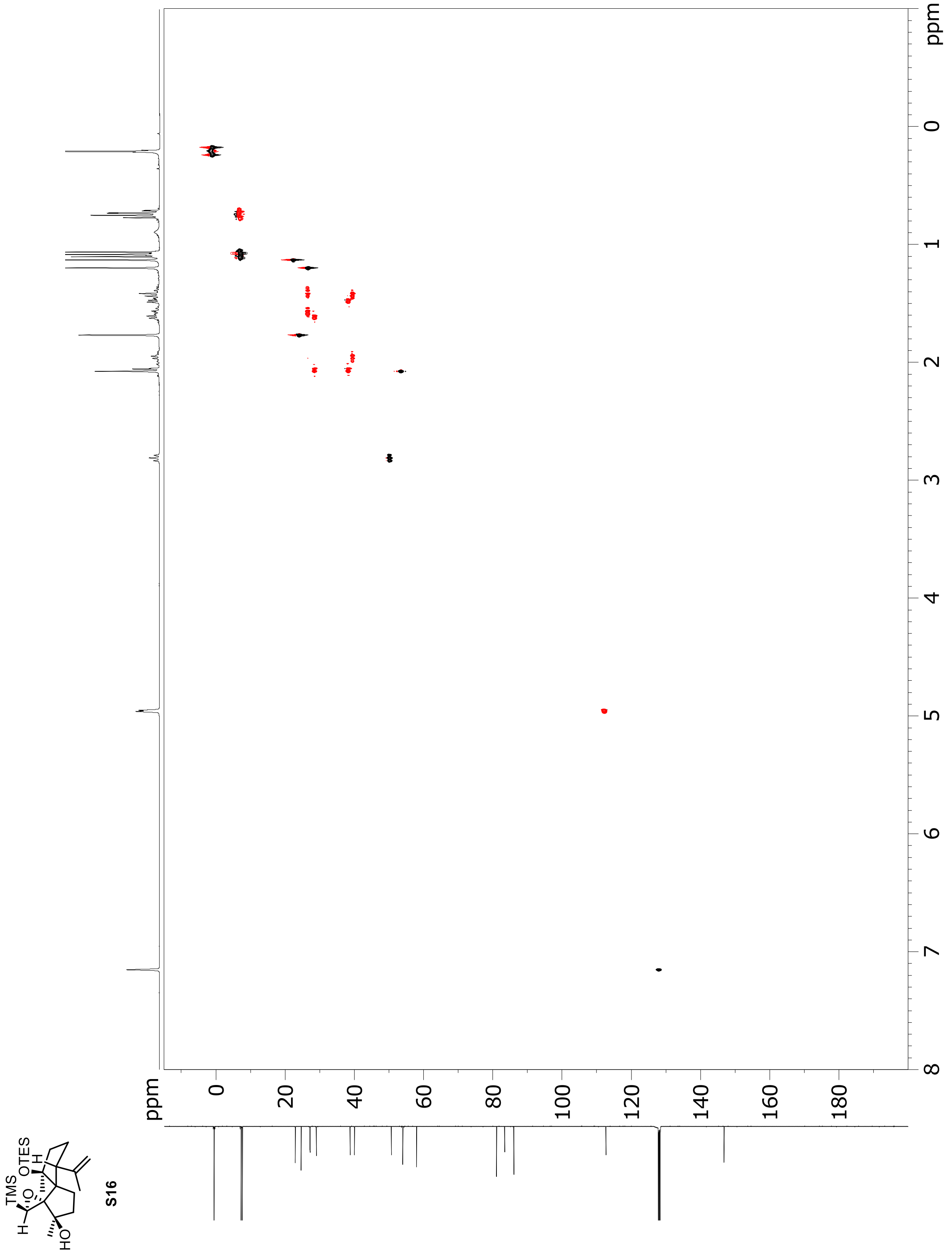

HSQC NMR spectrum of TMS-epoxide **S16** measured in C_6D_6 at 400 MHz.

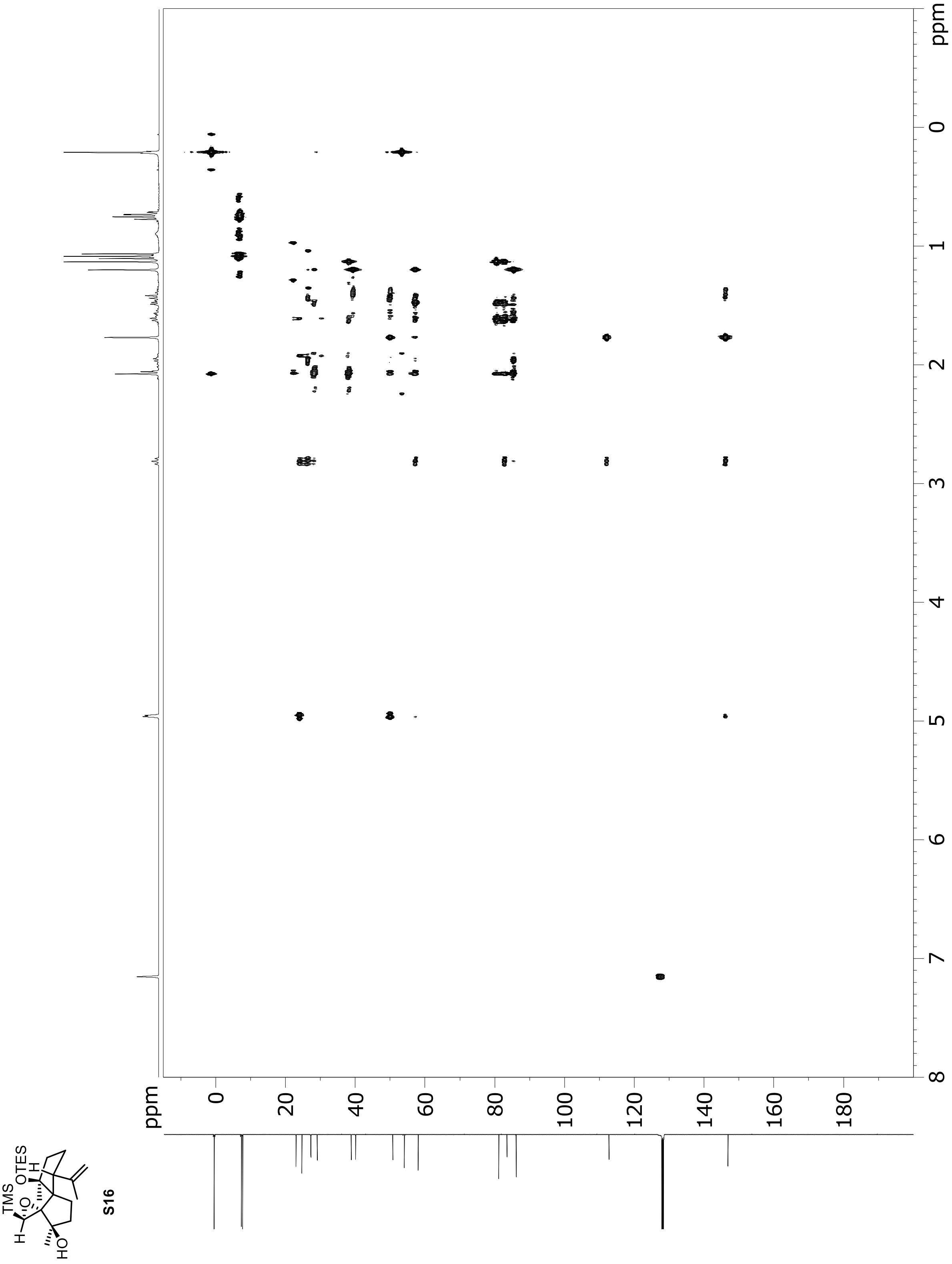

HMBC NMR spectrum of TMS-epoxide **S16** measured in C_6D_6 at 400 MHz.

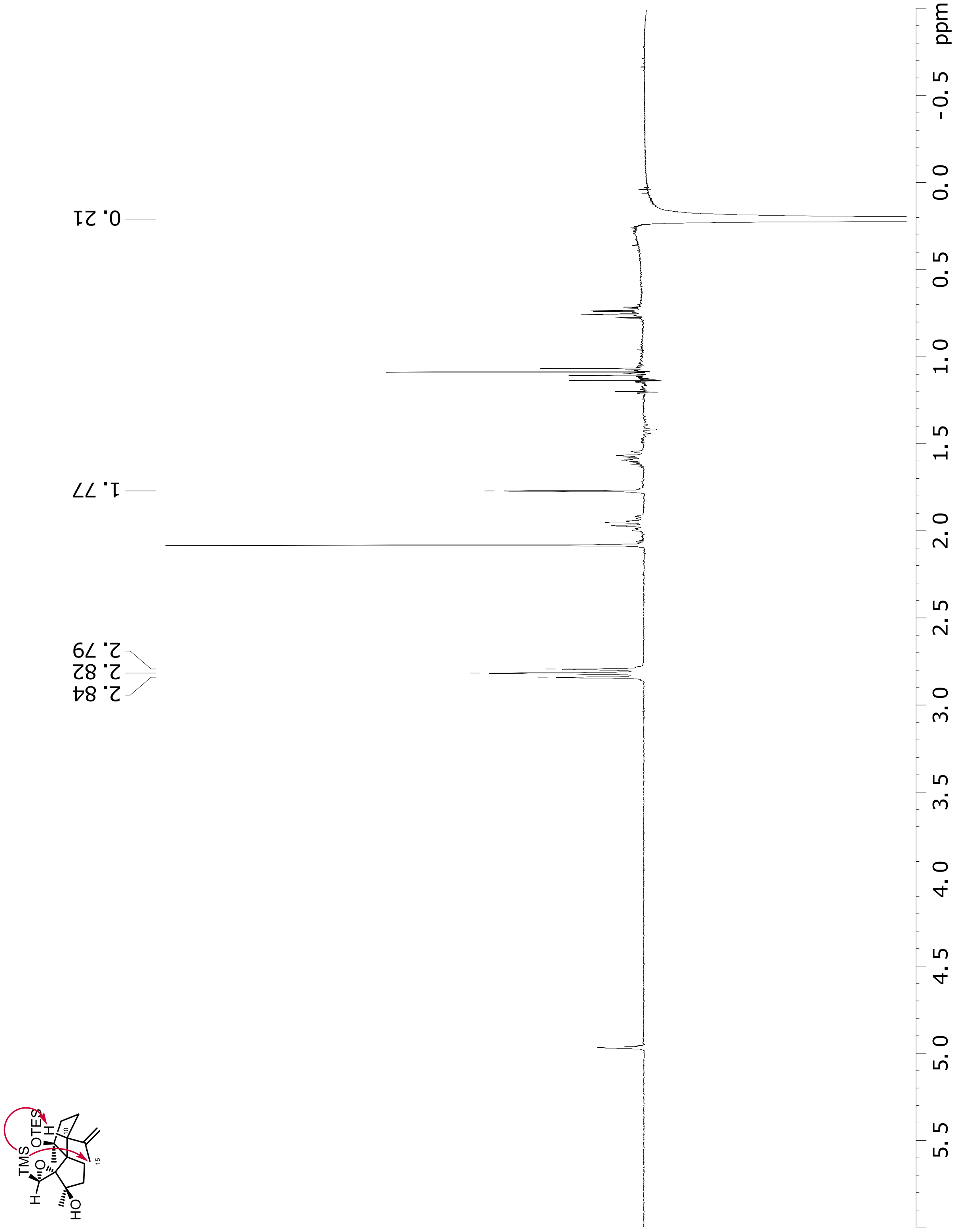

¹H-NOE spectrum of TMS-epoxide **S16** after irradiation at 0.21 ppm (TMS), measured in C_6D_6 at 400 MHz.

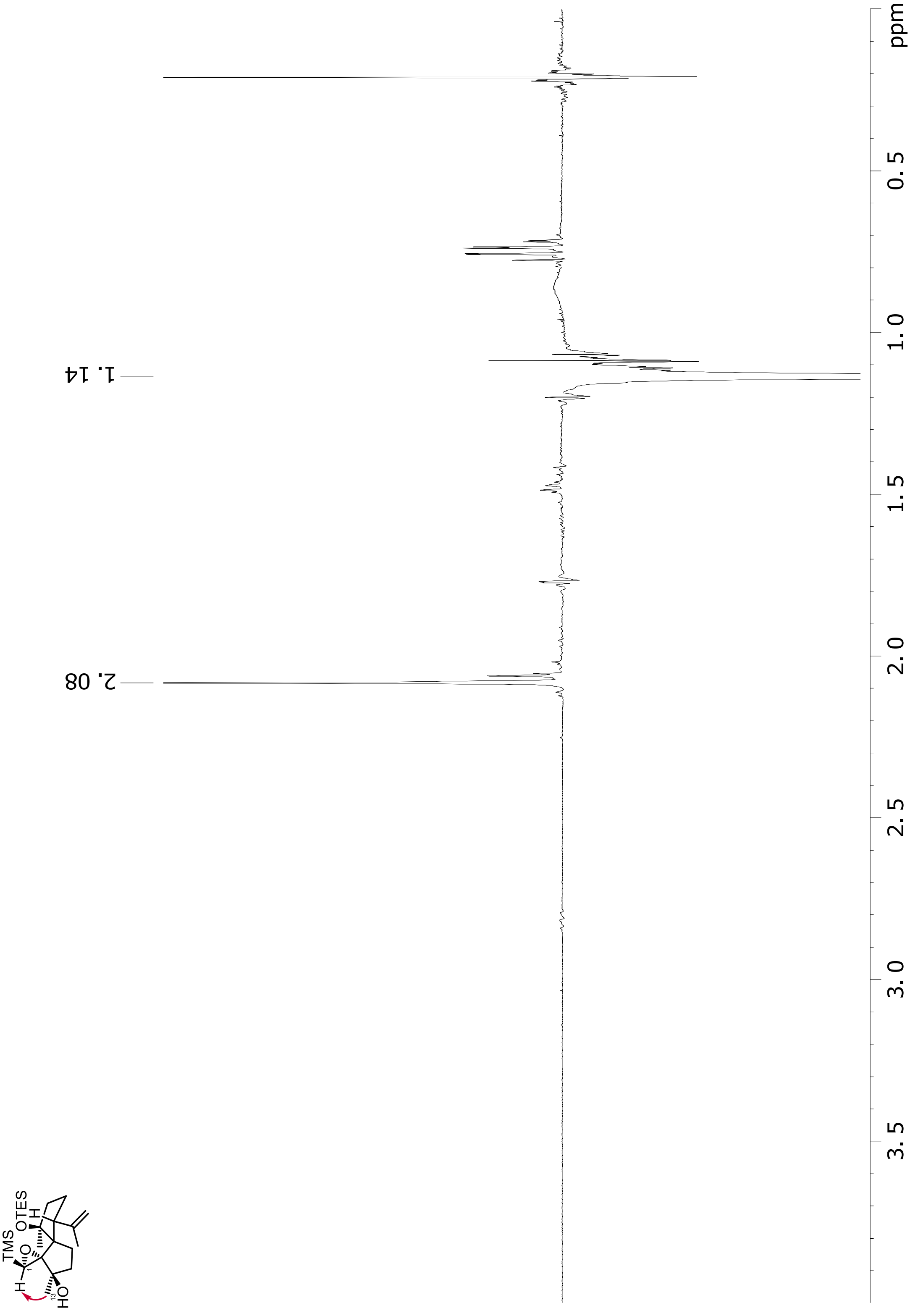

^{1}H-NOE spectrum of TMS-epoxide **S16** after irradiation at 1.14 ppm (CH_3-13), measured in C_6D_6 at 400 MHz.

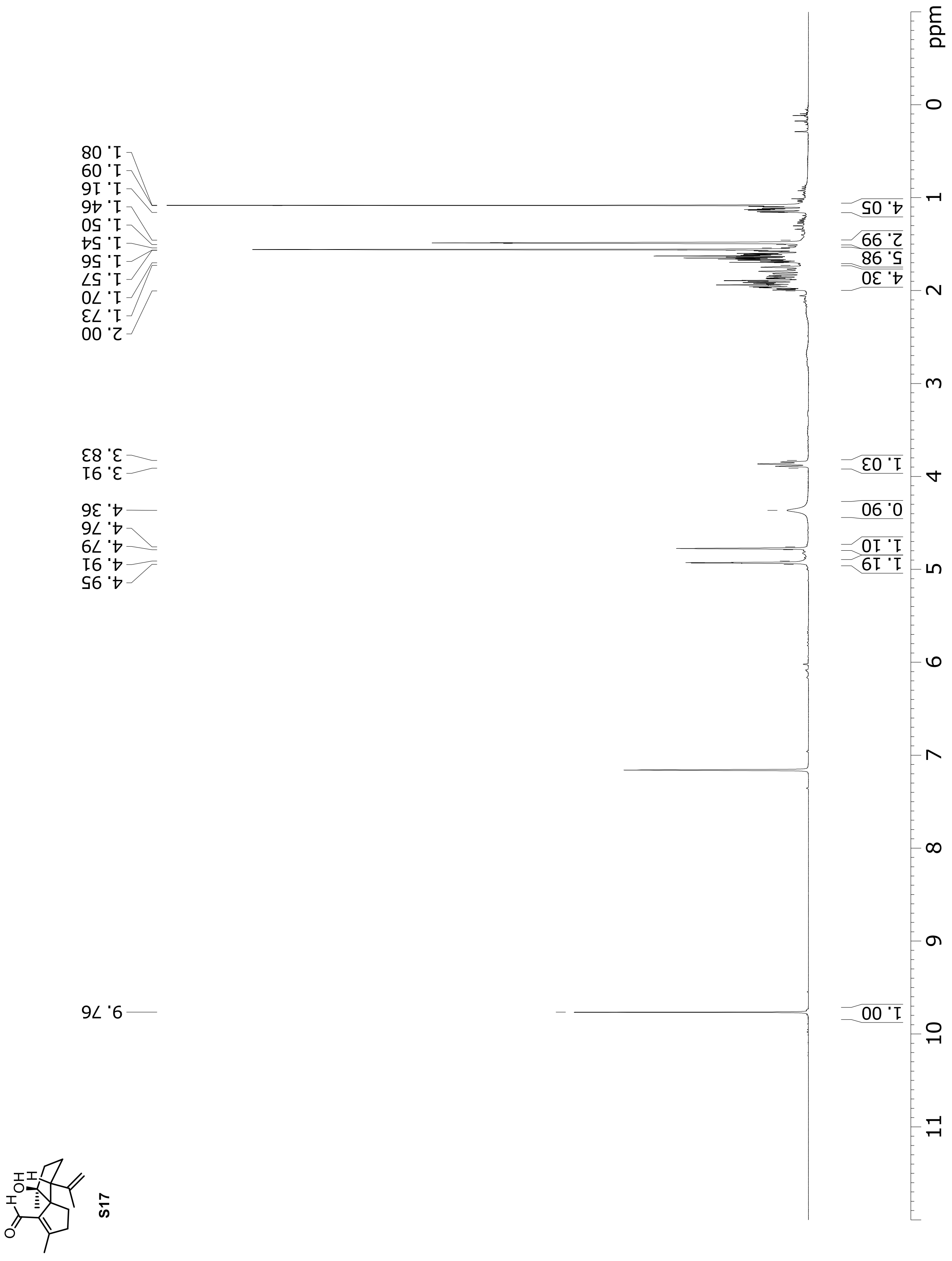

^{1}H NMR spectrum of aldehyde **S17** measured in C_6D_6 at 400 MHz

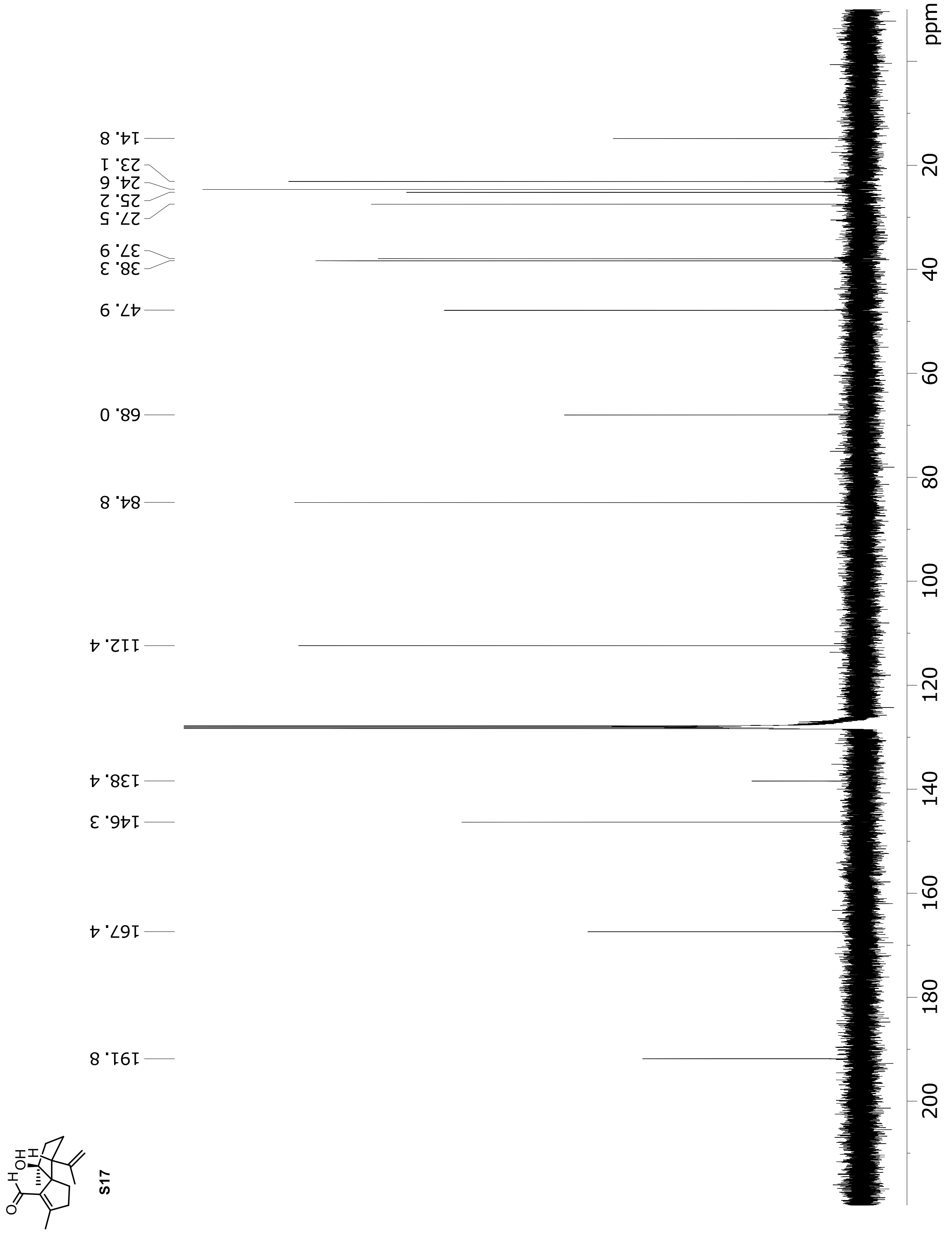

^{13}C NMR spectrum of aldehyde **S17** measured in C_6D_6 at 101 MHz..

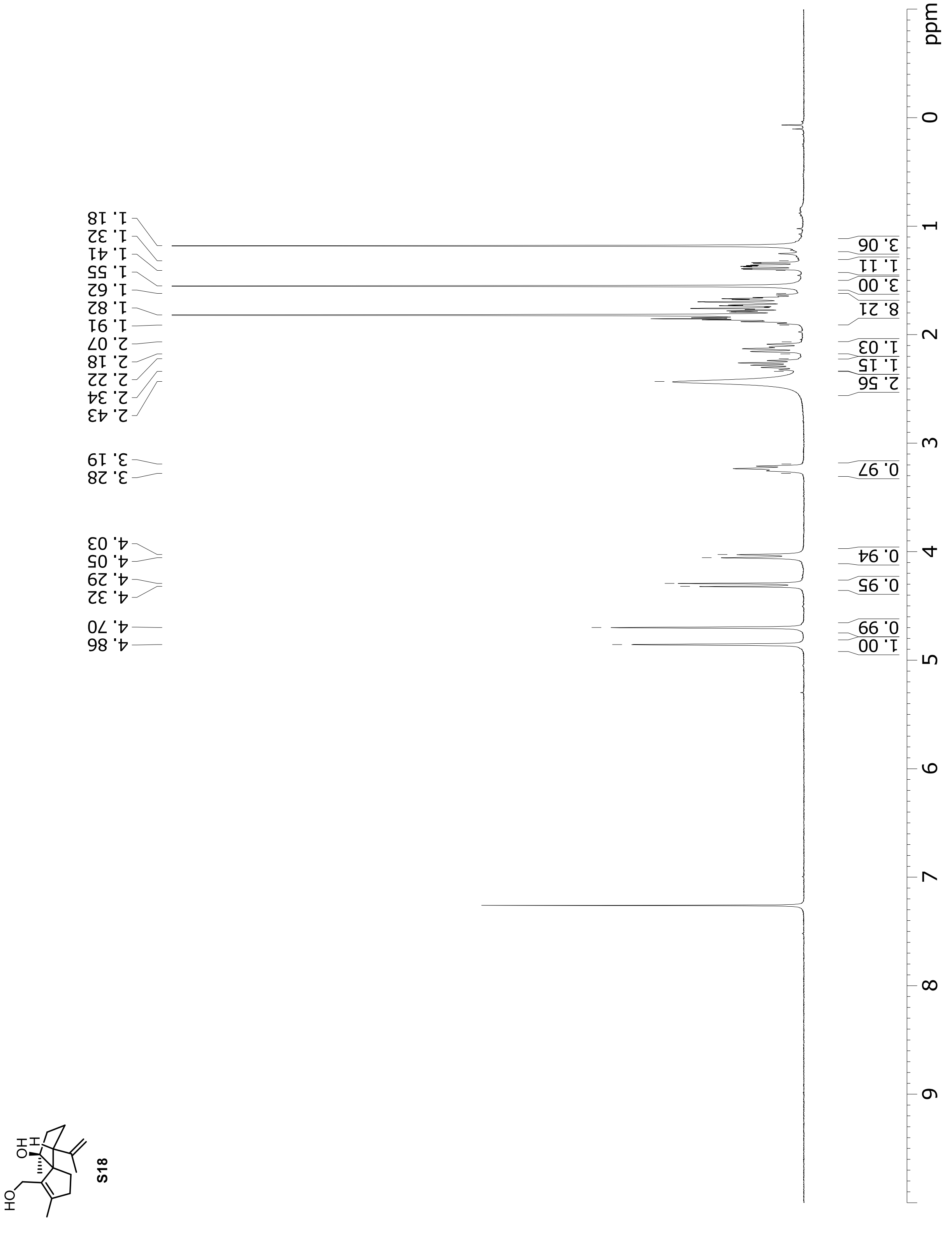

^{1}H NMR spectrum of diol **S18** measured in $CDCl_3$ at 400 MHz

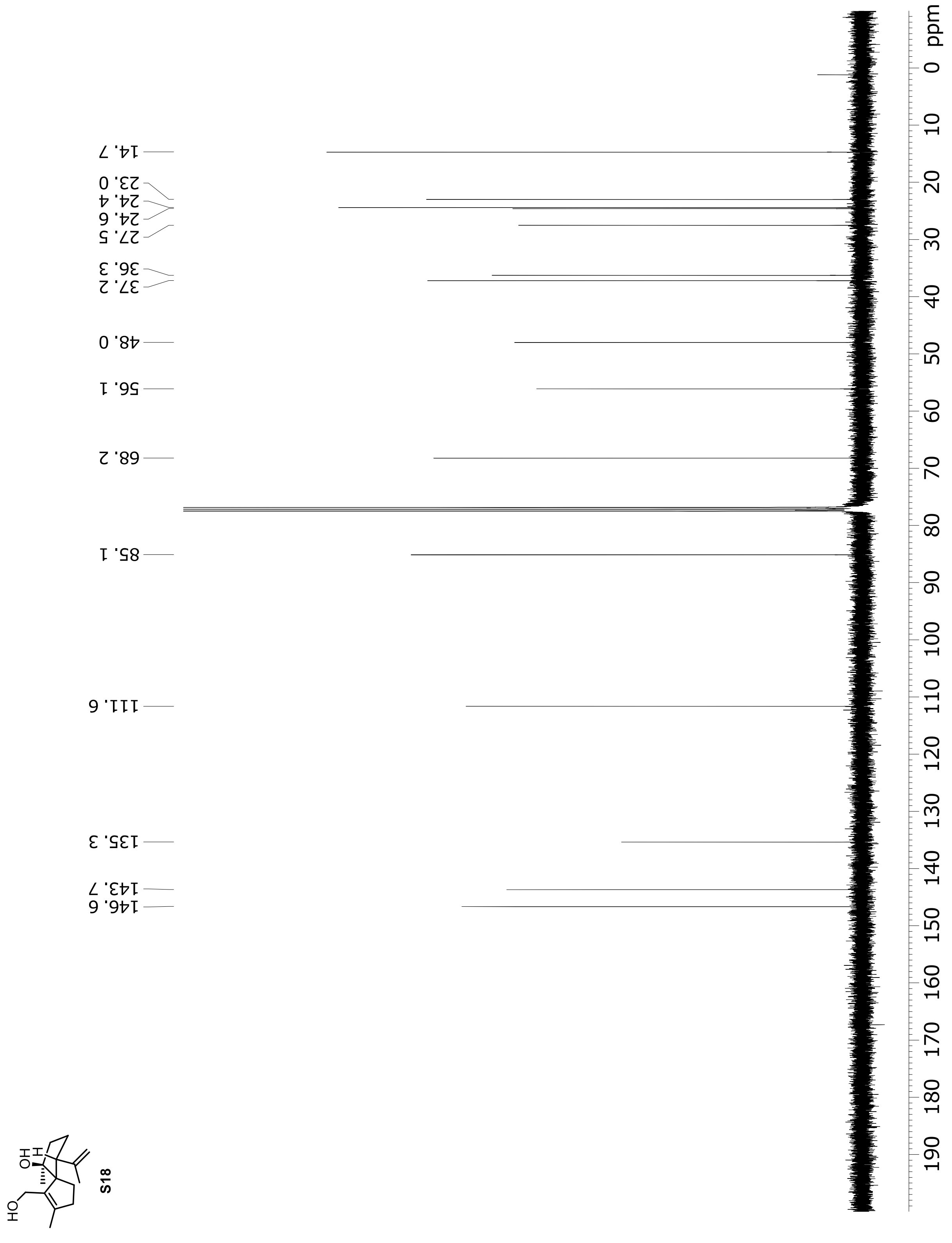

^{13}C NMR spectrum of diol S18 measured in $CDCl_3$ at 101 MHz.

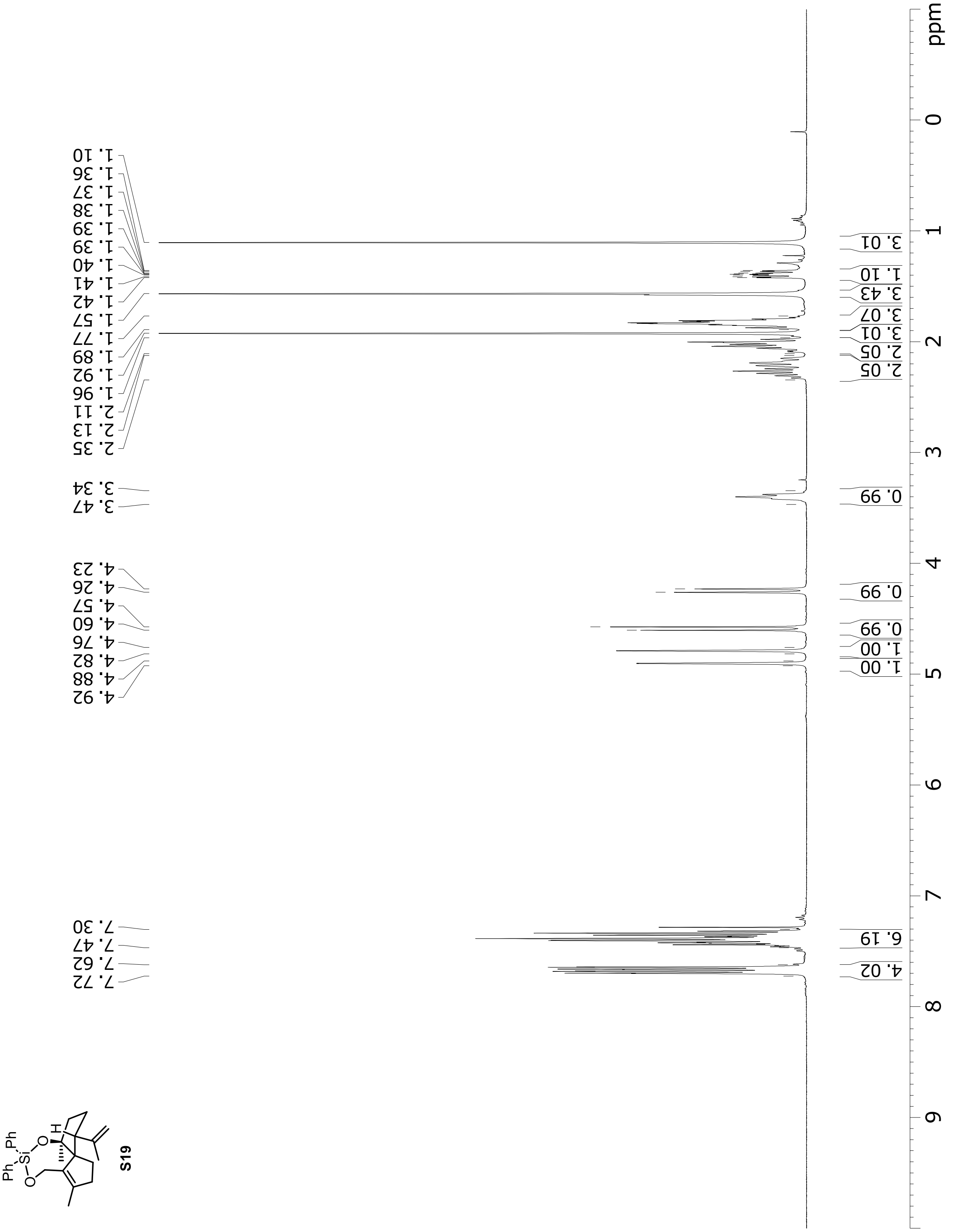

^{1}H NMR spectrum of Ph_2Si-protected diol **S19** measured in $CDCl_3$ at 400 MHz

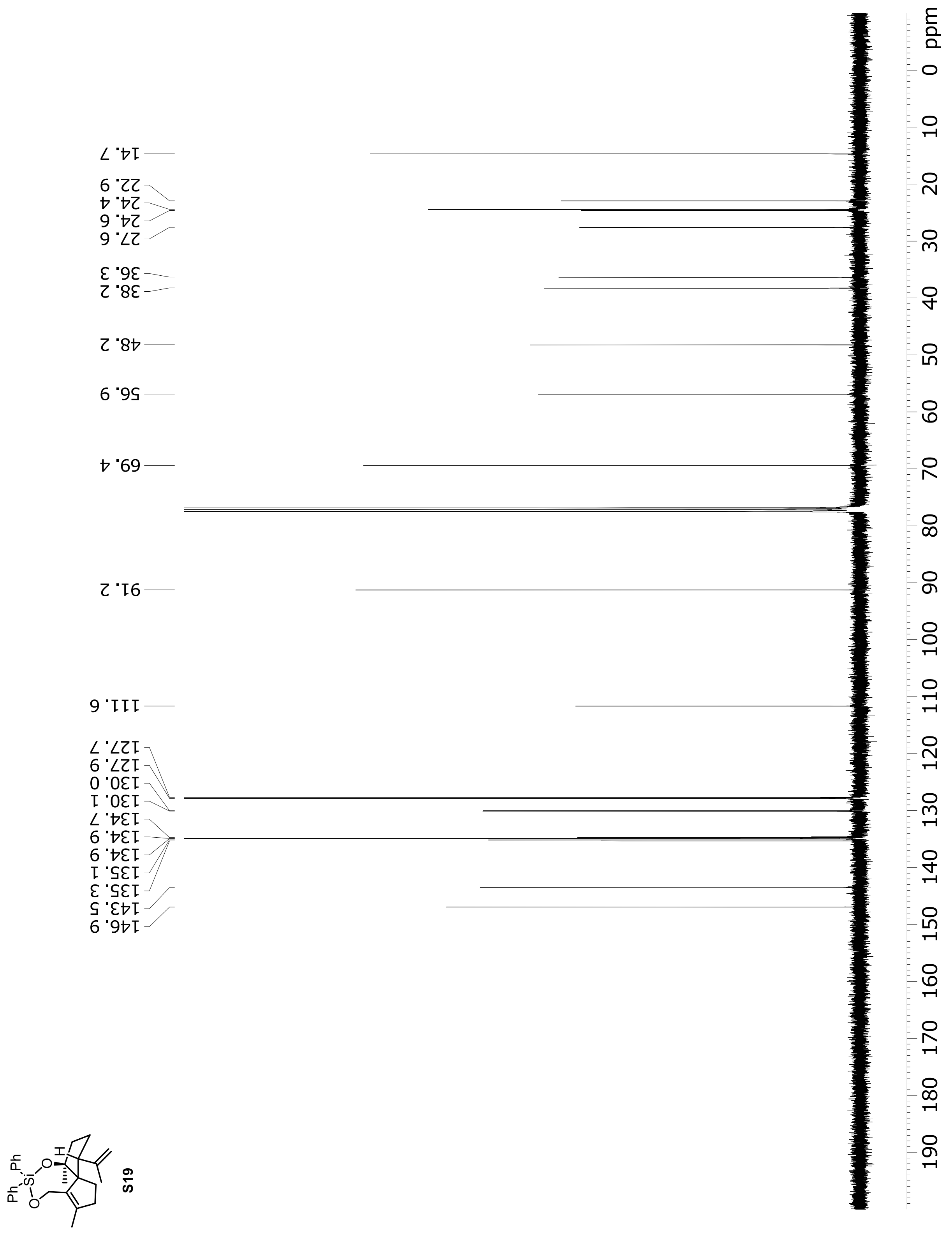

^{13}C NMR spectrum of Ph_2Si-protected diol **S19** measured in $CDCl_3$ at 101 MHz.

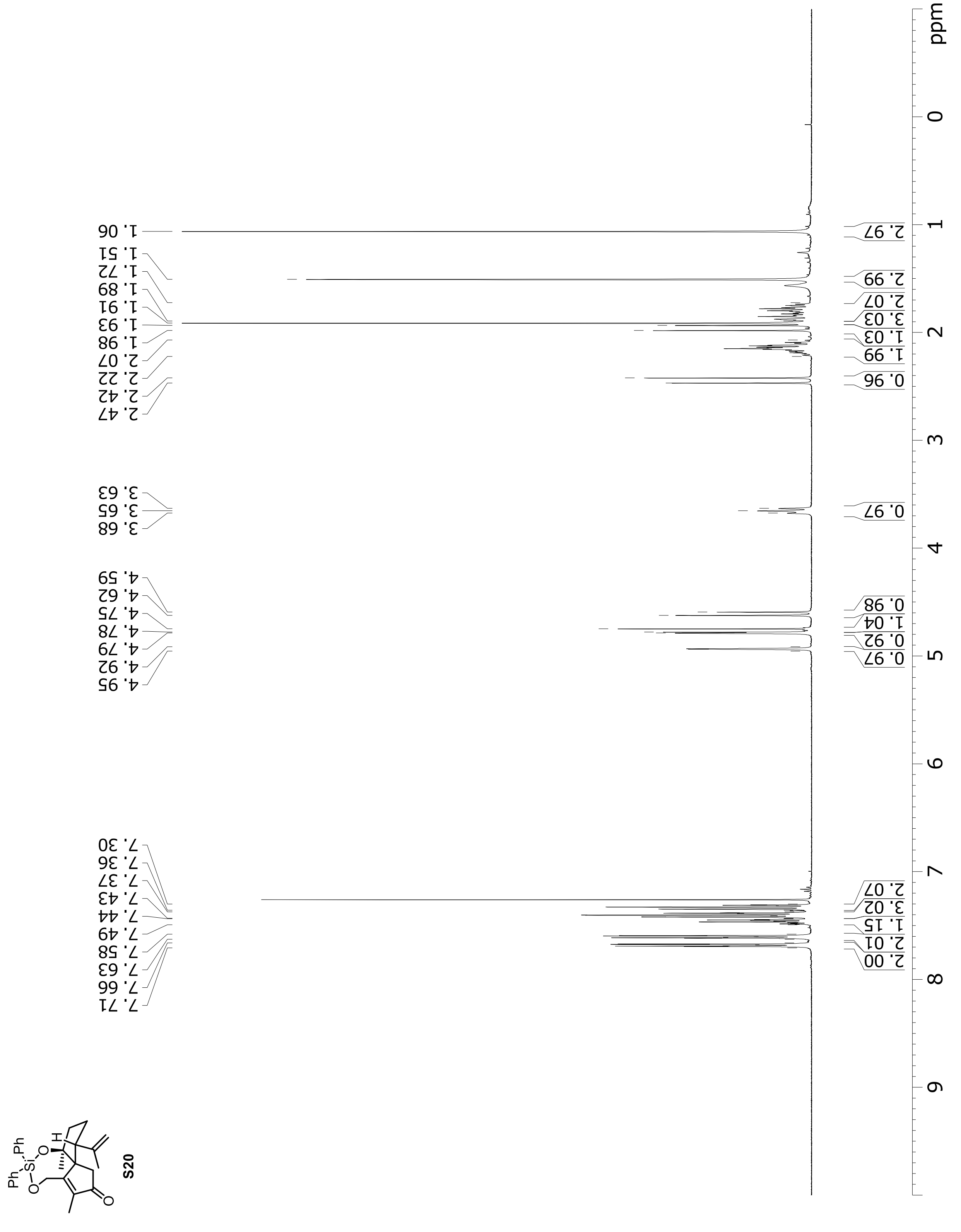

^{1}H NMR spectrum of enone **S20** measured in $CDCl_3$ at 400 MHz

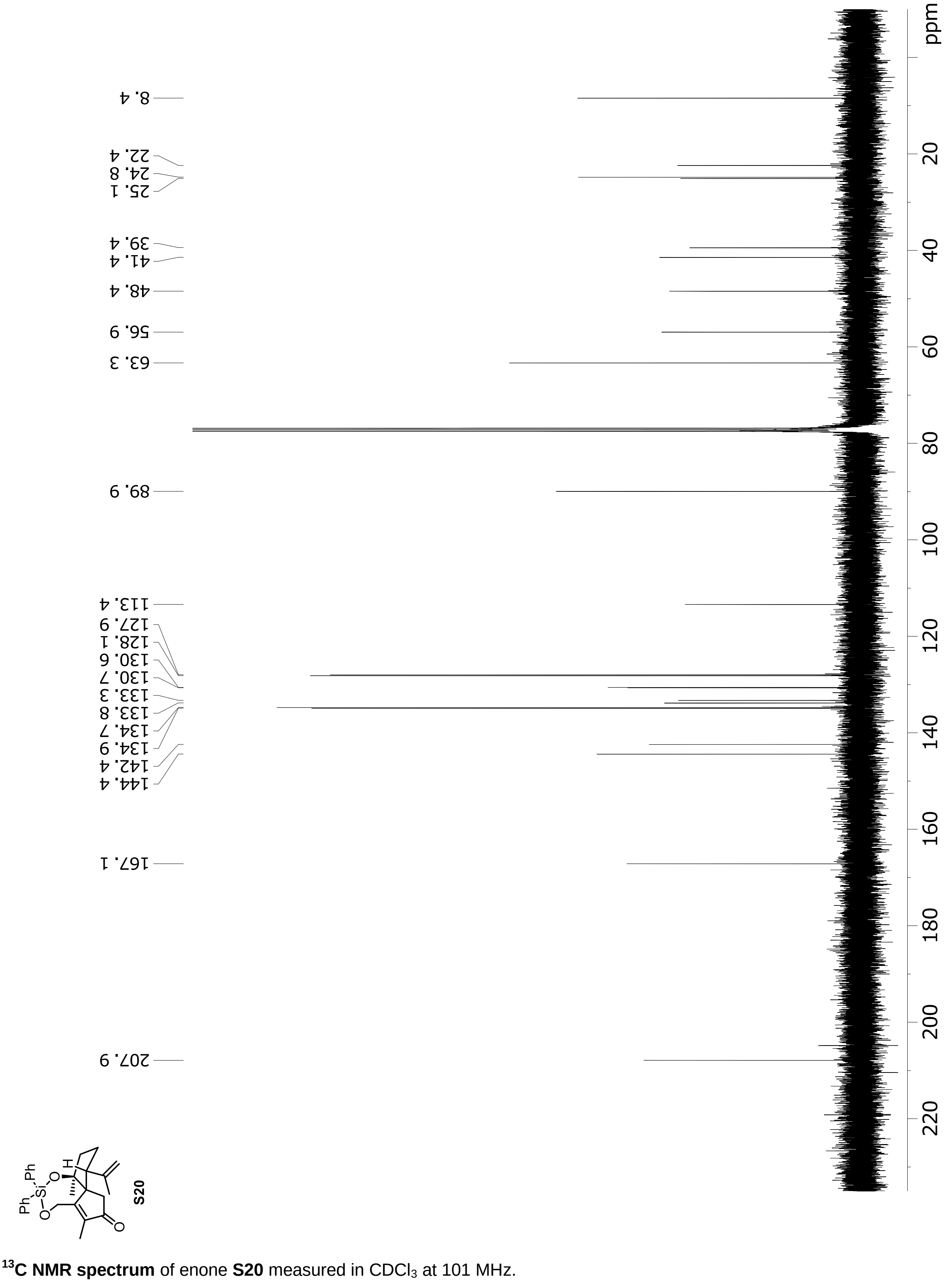

^{13}C NMR spectrum of enone **S20** measured in $CDCl_3$ at 101 MHz.

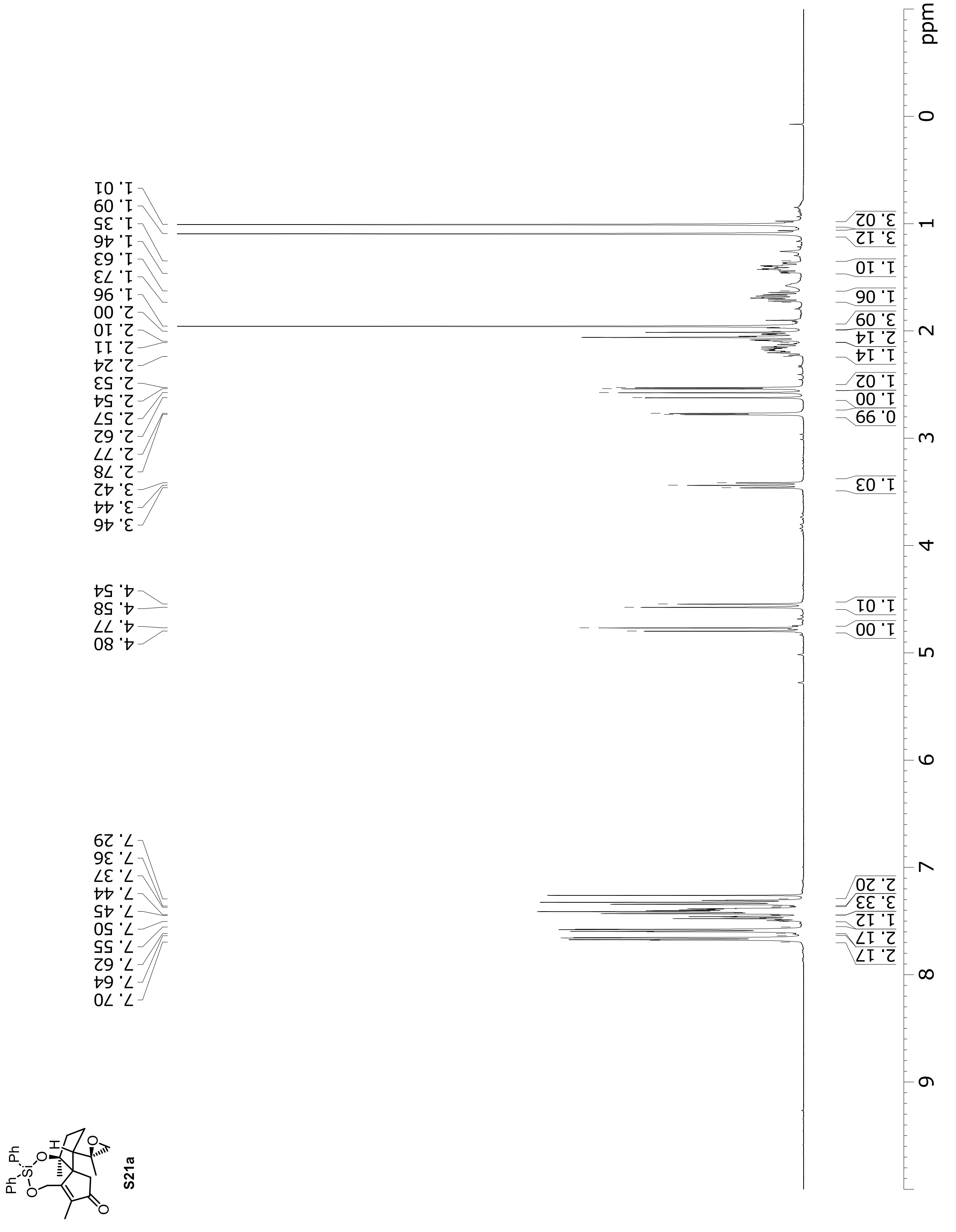

^{1}H NMR spectrum of epoxy enone **S21a** (major diastereomer) measured in $CDCl_3$ at 400 MHz

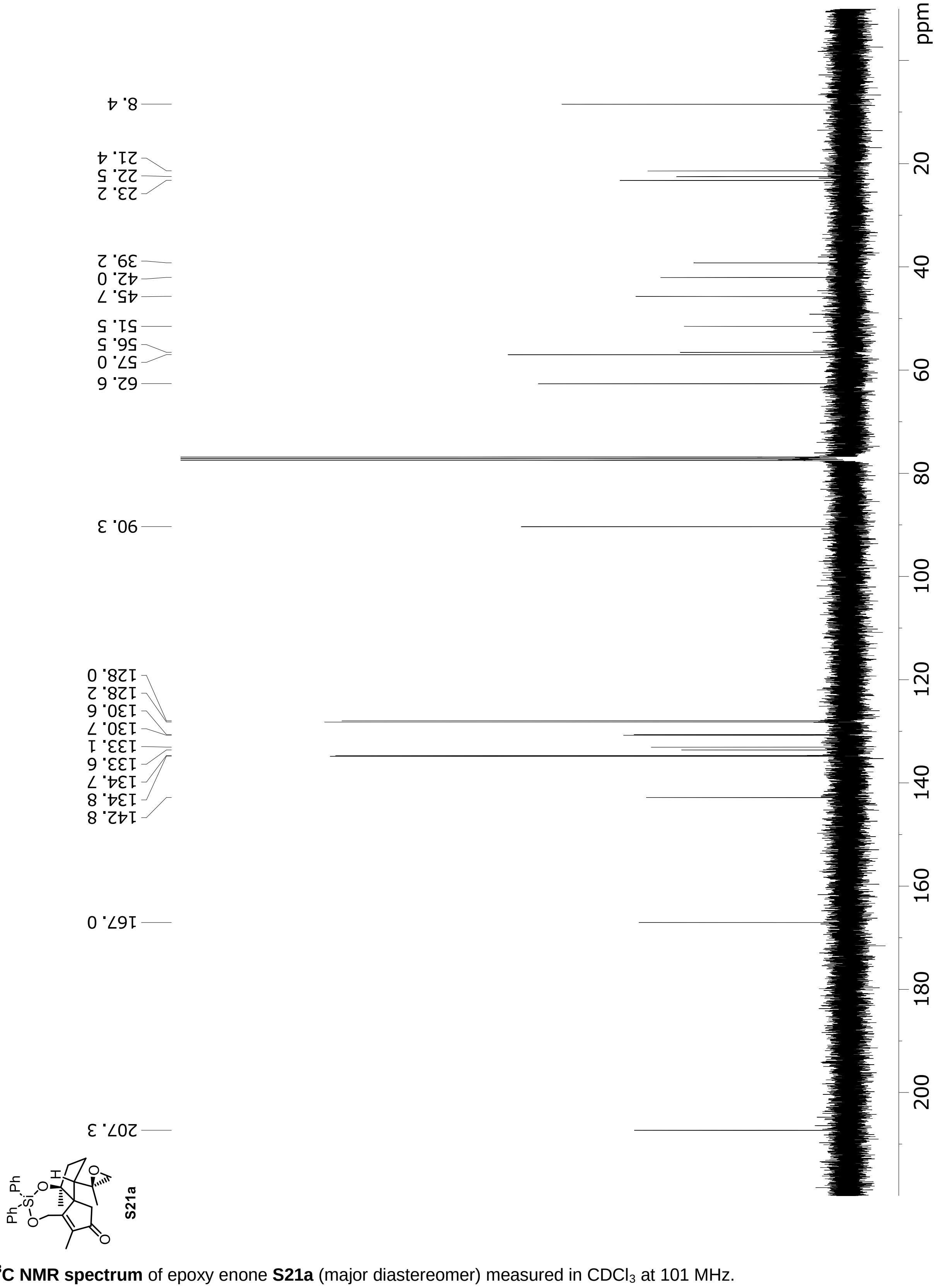

^{13}C NMR spectrum of epoxy enone **S21a** (major diastereomer) measured in $CDCl_3$ at 101 MHz.

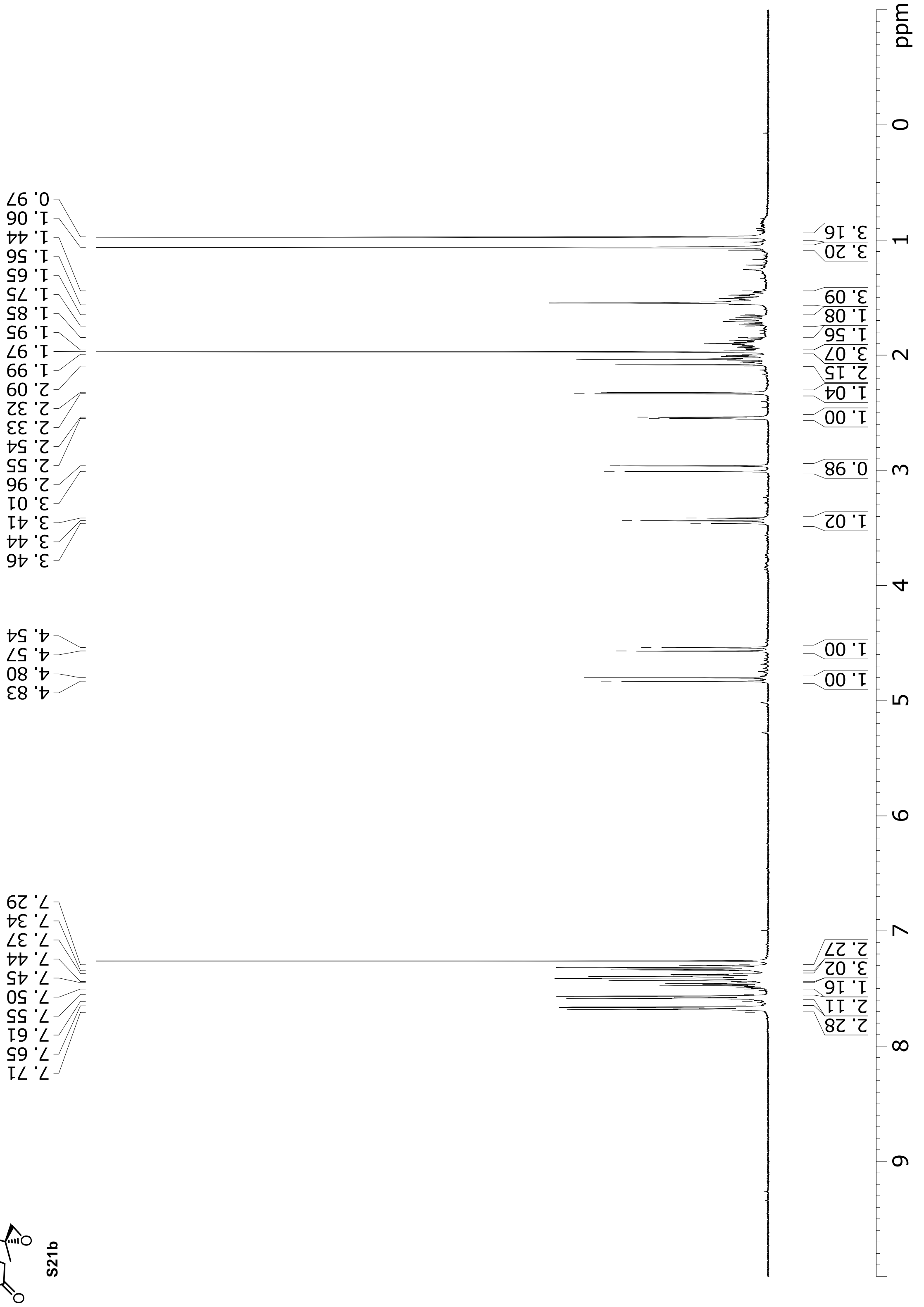

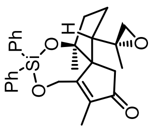

S21b

^{1}H NMR spectrum of epoxy enone S21b (minor diastereomer) measured in $CDCl_3$ at 400 MHz

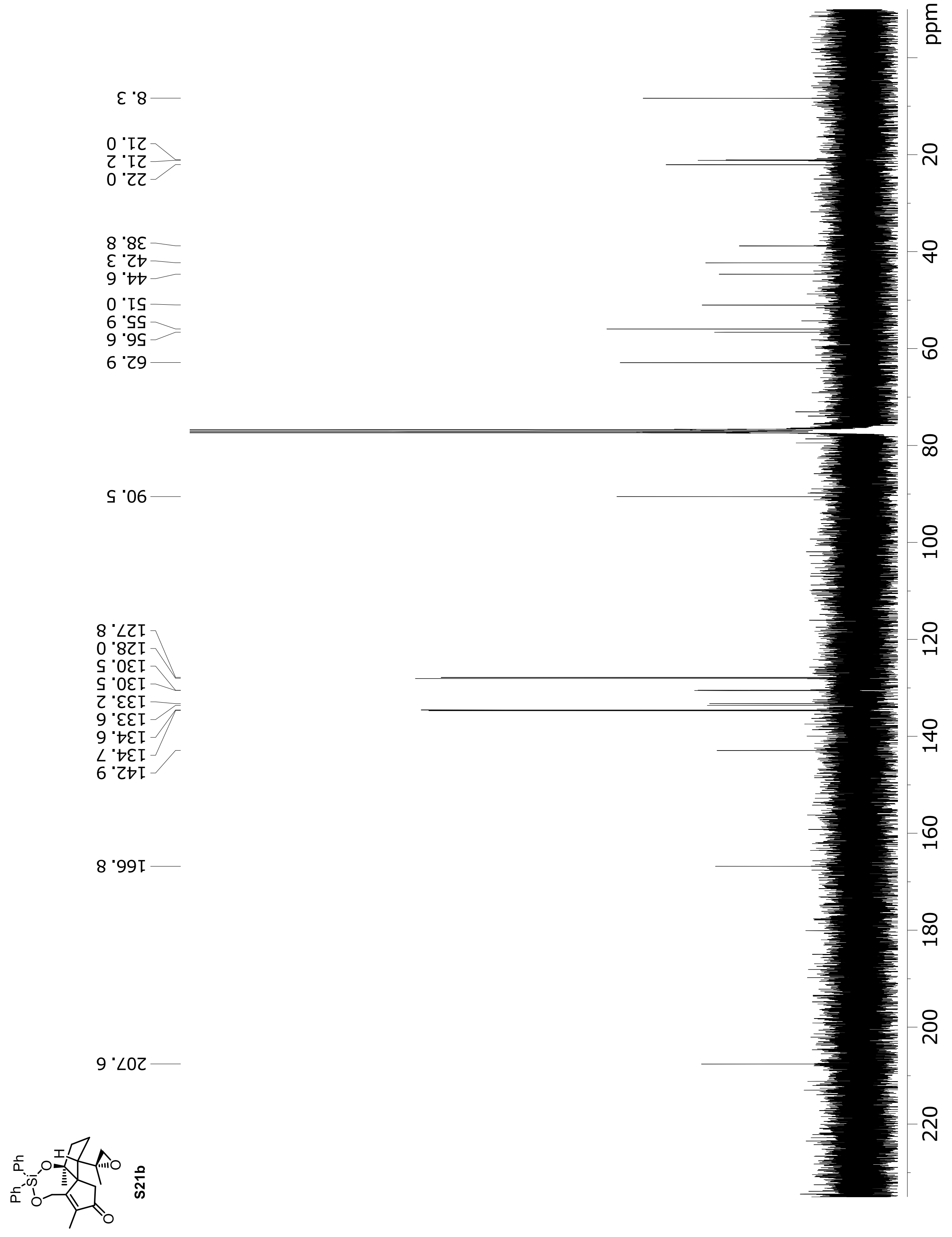

^{13}C NMR spectrum of epoxy enone **S21b** (minor diastereomer) measured in $CDCl_3$ at 101 MHz.

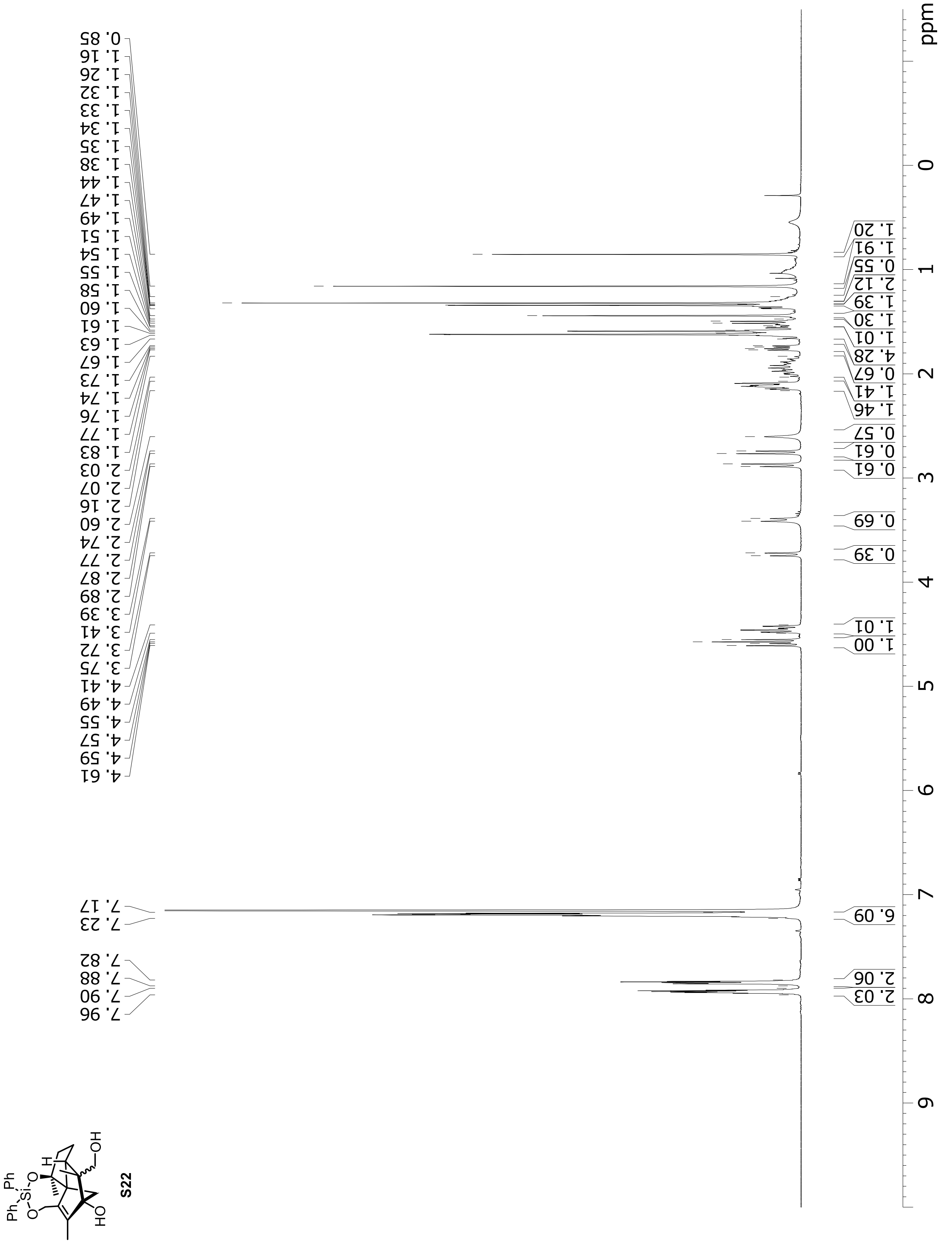

^{1}H NMR spectrum of cyclization product **S22** (diastereomeric mixture) measured in C_6D_6 at 400 MHz

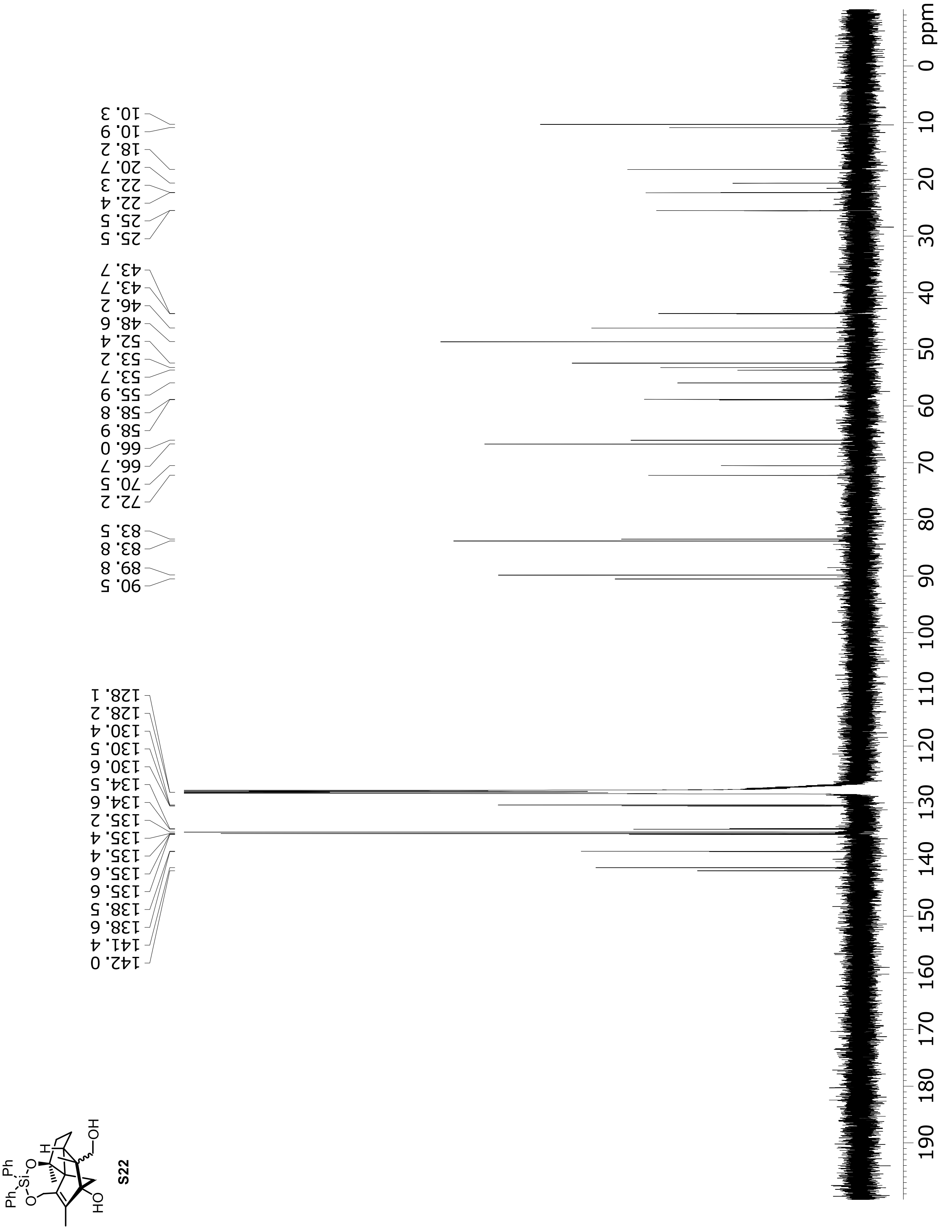

^{13}C NMR spectrum of cyclization product **S22** (diastereomeric mixture) measured in C_6D_6 at 101 MHz

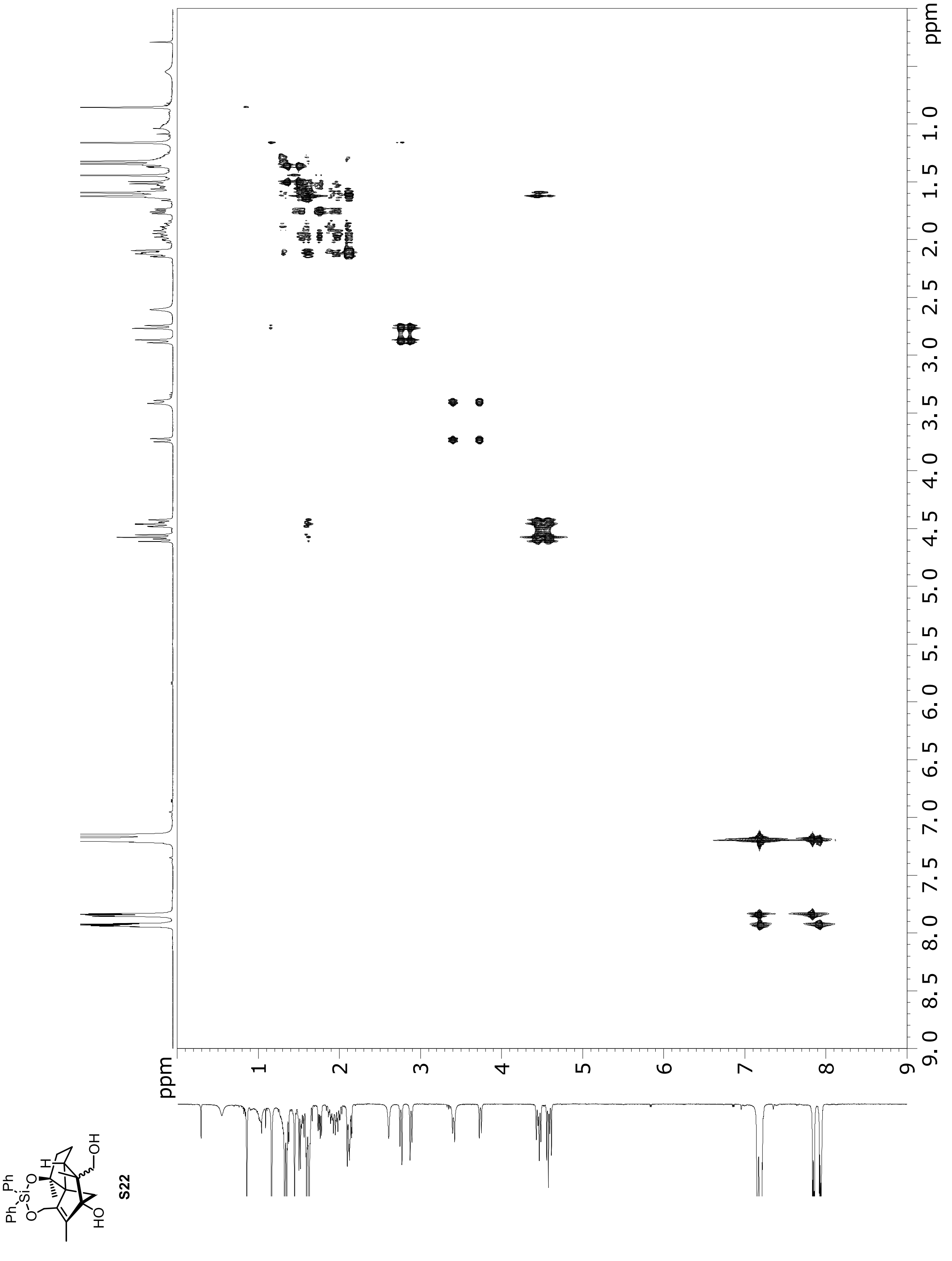

COSY spectrum of cyclization product **S22** (diastereomeric mixture) measured in C_6D_6 at 400 MHz.

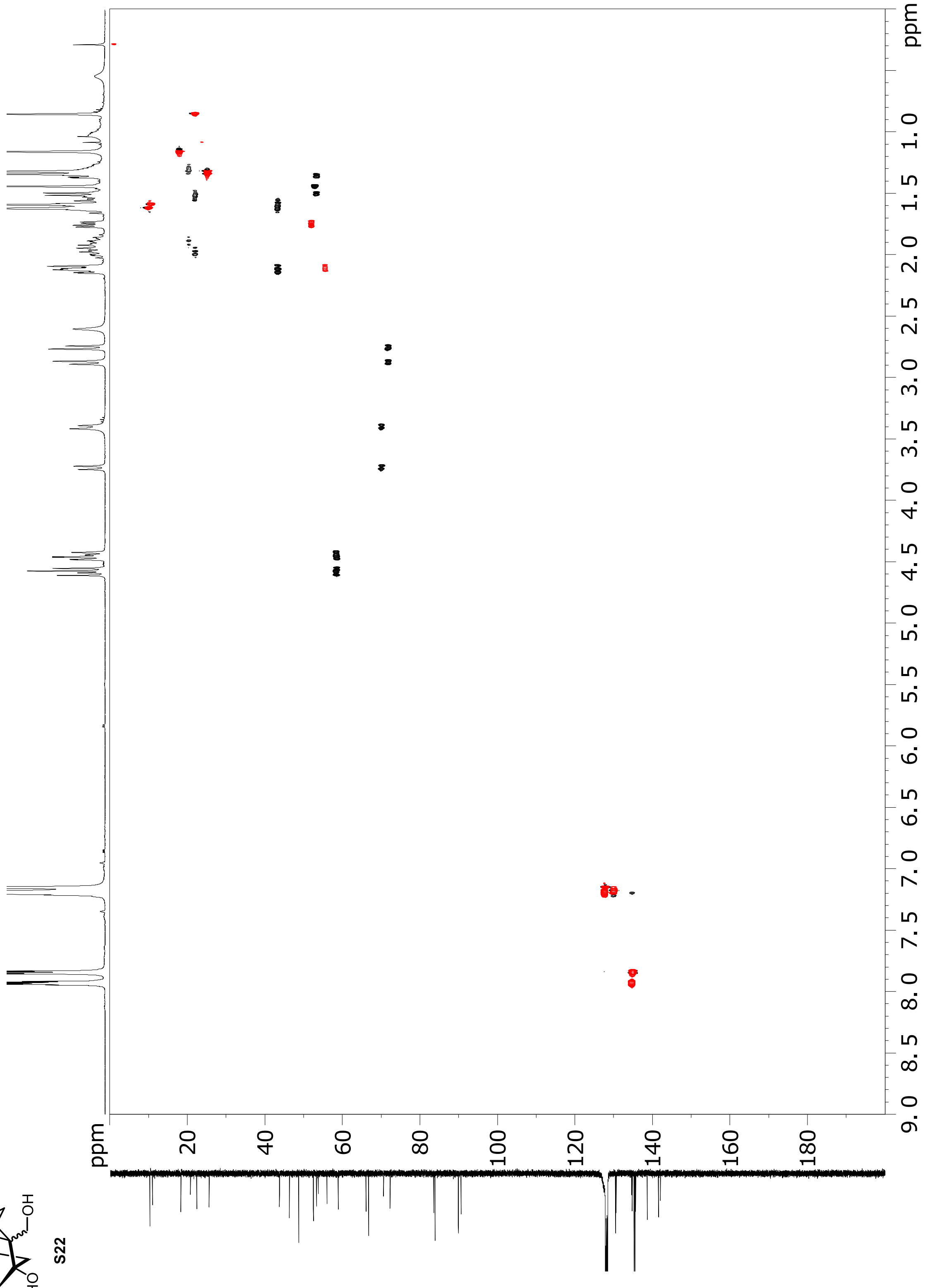

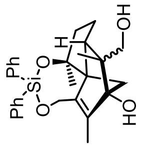

S22

HSQC spectrum of cyclization product **S22** (diastereomeric mixture) measured in C_6D_6 at 400 MHz.

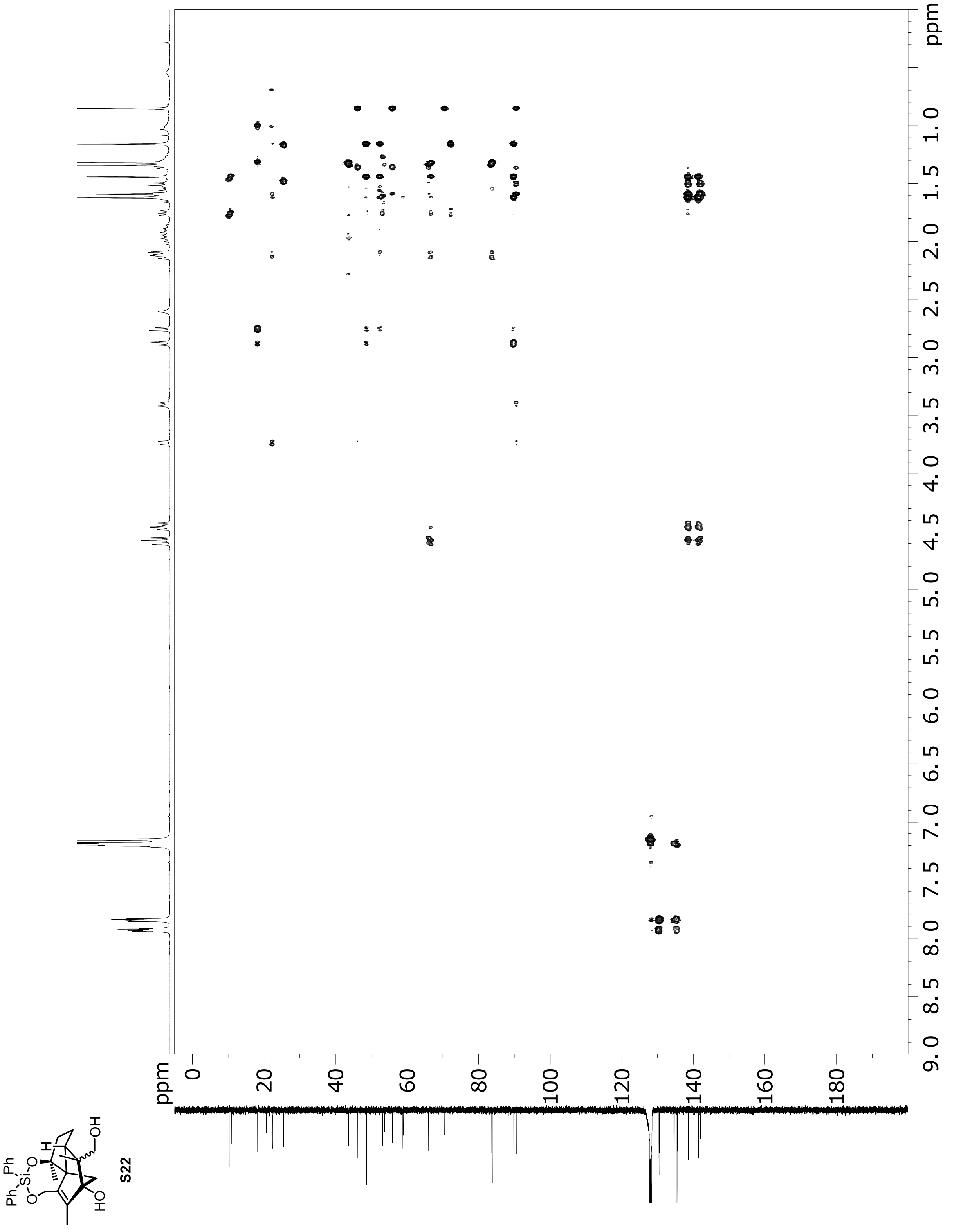

HMBC spectrum of cyclization product **S22** (diastereomeric mixture) measured in C_6D_6 at 400 MHz.

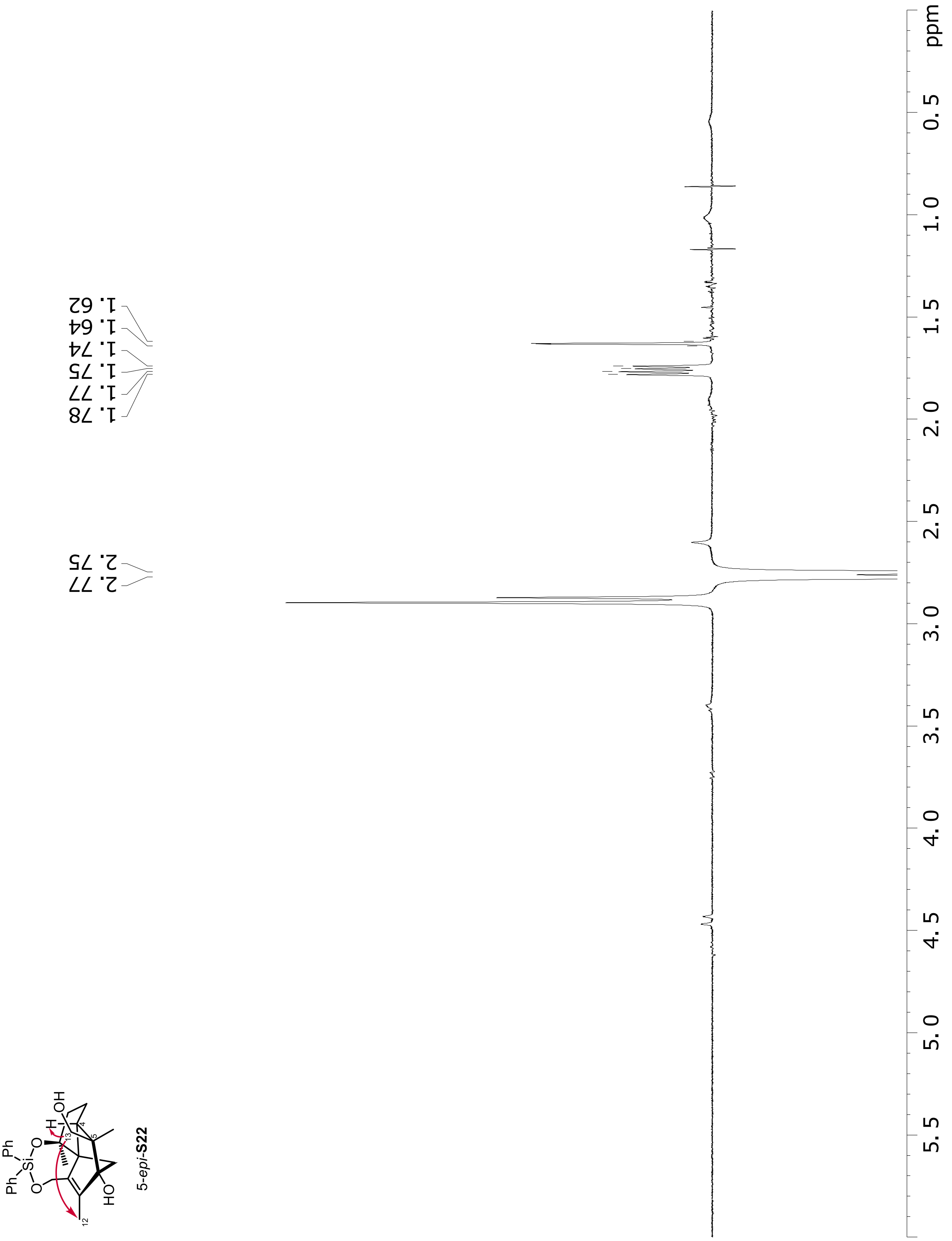

^{1}H-NOE spectrum of cyclization product **S22** (diastereomeric mixture) after irradiation at 2.75 ppm (major H-13b), showing the diagnostic correlations for the proposed stereoconfiguration of the major diastereomer. Spectrum measured in C_6D_6 at 400 MHz.

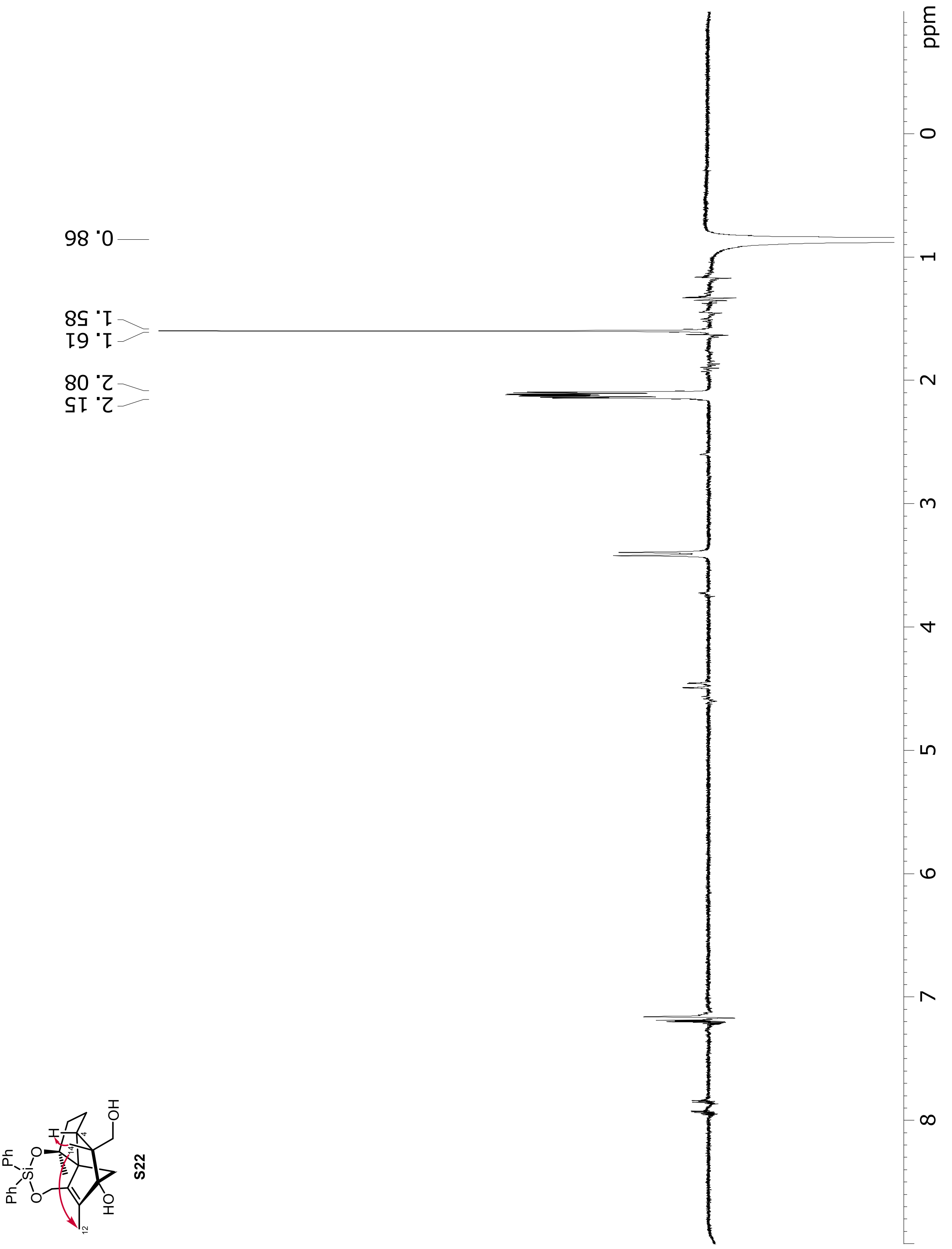

^{1}H-NOE spectrum of cyclization product **S22** (diastereomeric mixture) after irradiation at 0.86 ppm (minor CH_3-14), showing the diagnostic correlations for the proposed stereoconfiguration of the minor diastereomer. Spectrum measured in C_6D_6 at 400 MHz.

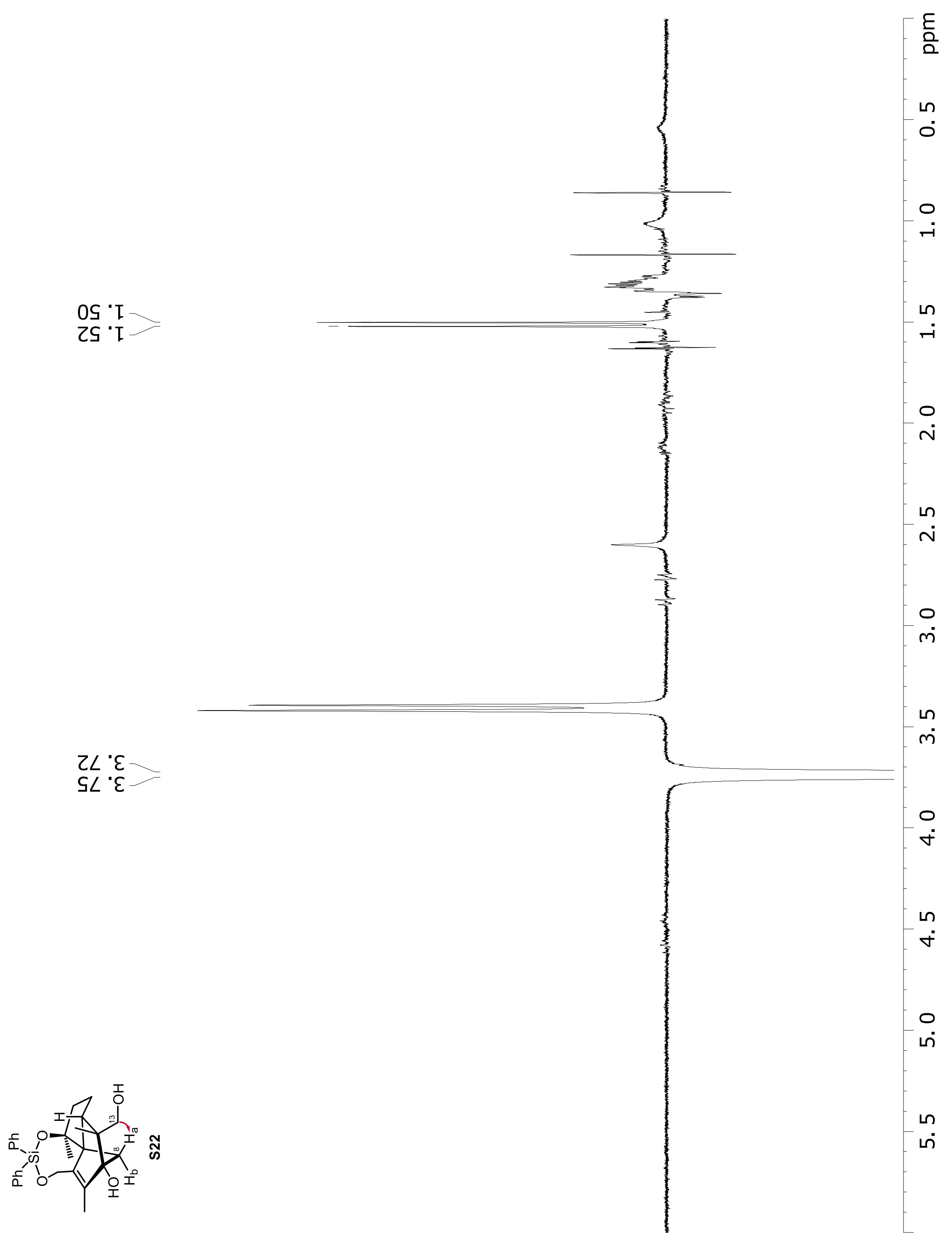

^{1}H-NOE spectrum of cyclization product **S22** (diastereomeric mixture) after irradiation at 3.73 ppm (minor H-13a), showing the diagnostic correlations for the proposed stereoconfiguration of the minor diastereomer. Spectrum measured in C_6D_6 at 400 MHz.

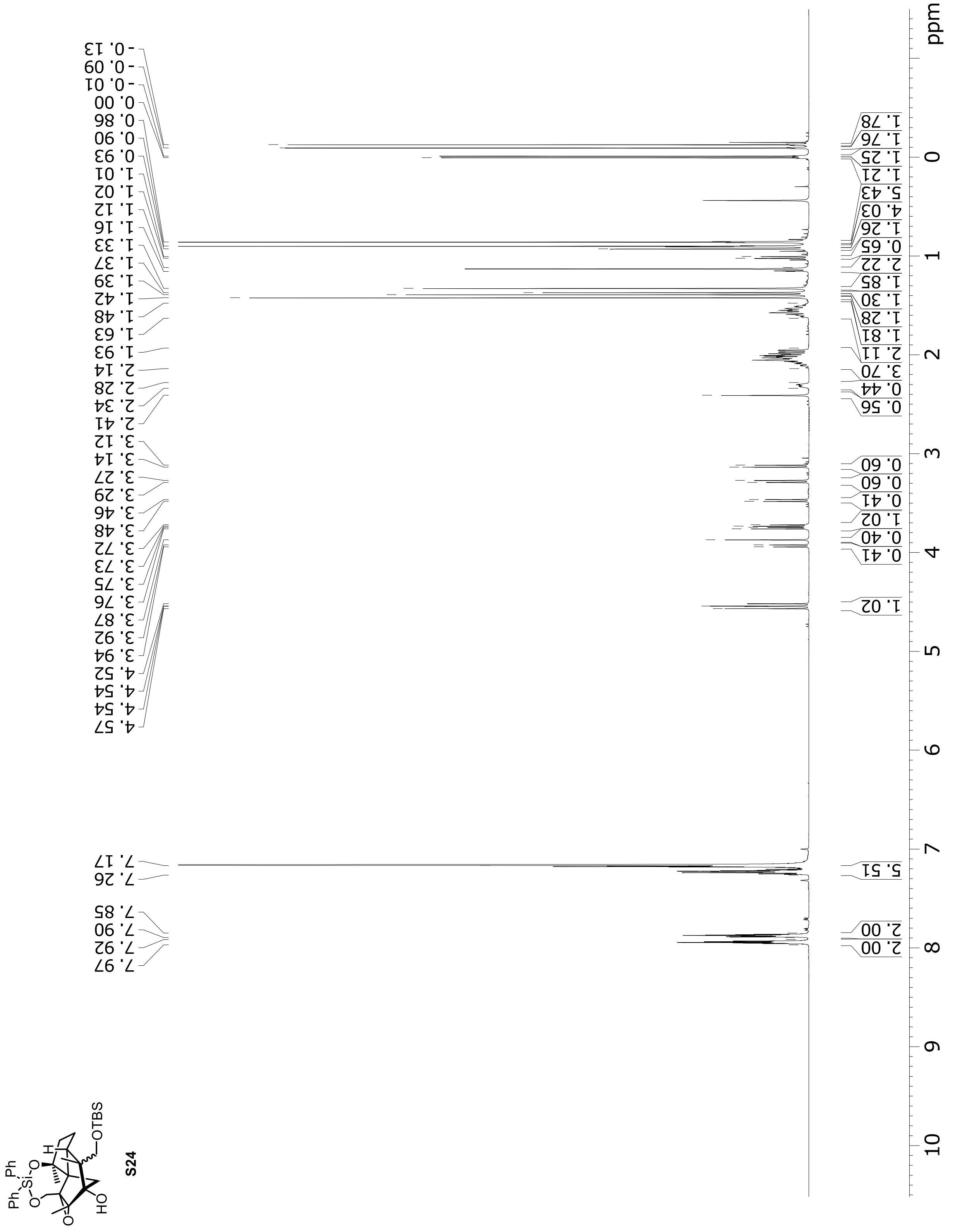

^{1}H NMR spectrum of epoxide **S24** measured in C_6D_6 at 500 MHz. In the spectra of **S24** always a small amount of rearranged product 5-*epi*-**S25** was visible. We were not able to suppress its formation.

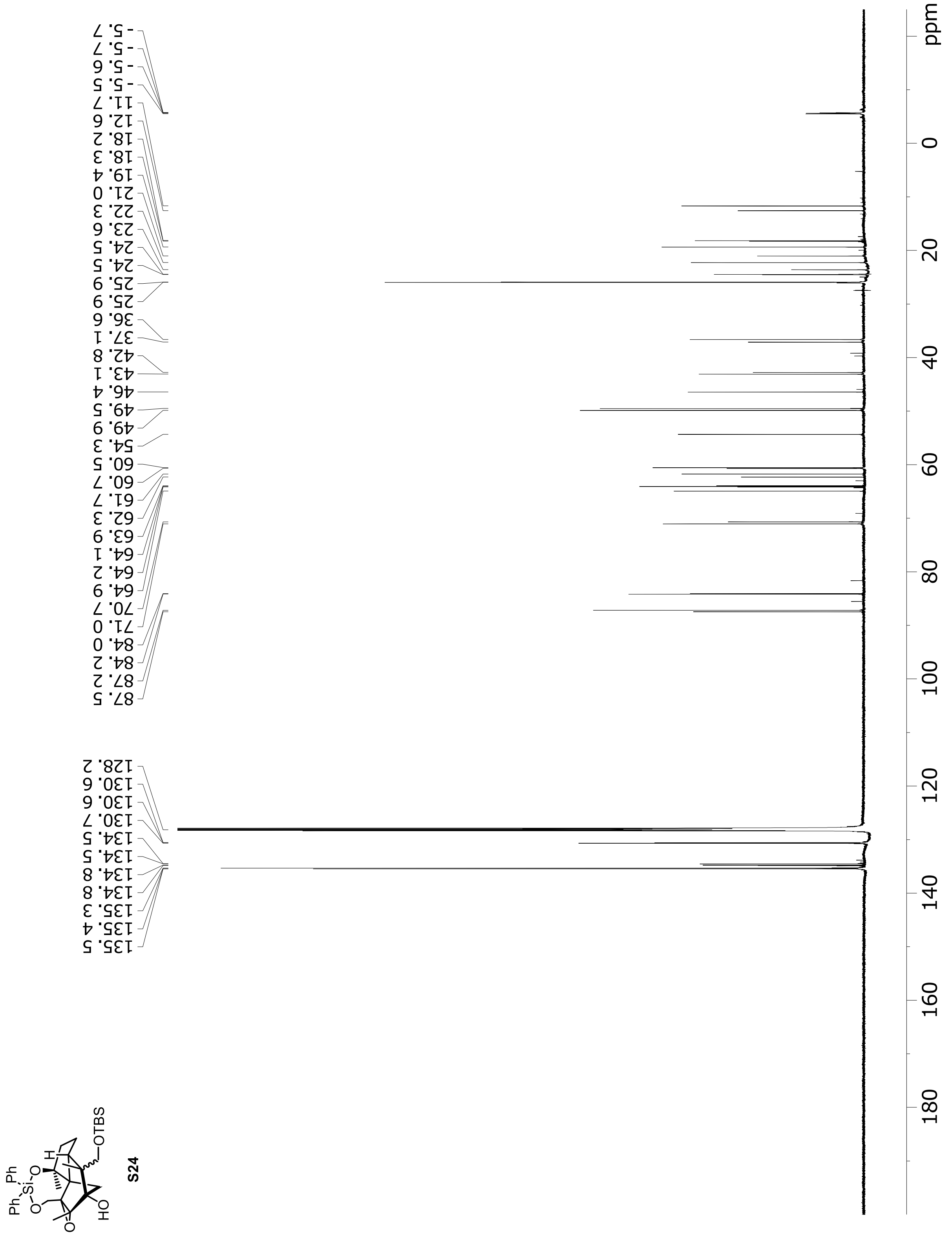

^{13}C NMR spectrum of epoxide **S24** measured in C_6D_6 at 126 MHz. In the spectra of **S24** always a small amount of rearranged product 5-*epi*-**S25** was visible. We were not able to suppress its formation.

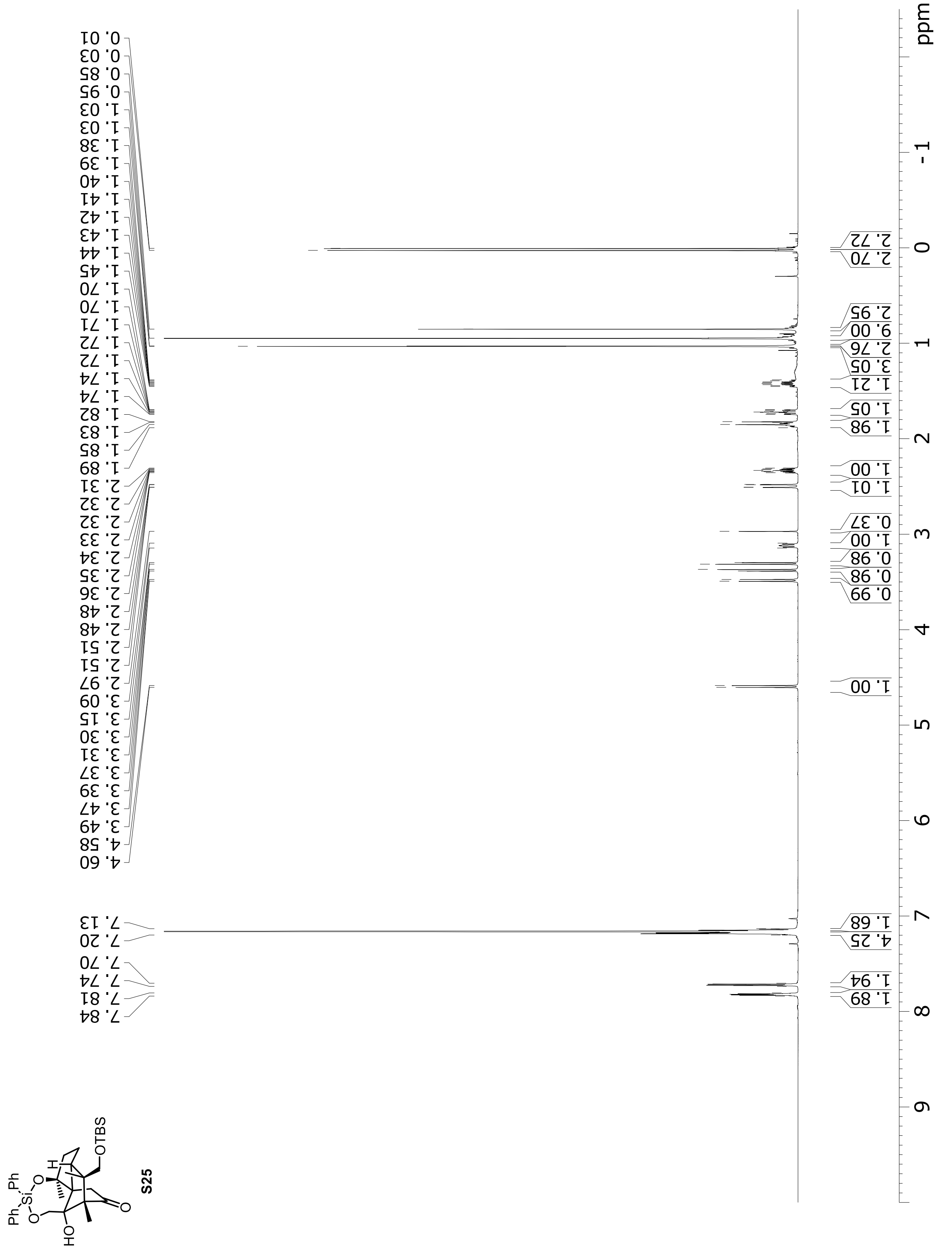

^{1}H NMR spectrum of semipinacol rearrangement product **S25** (desired diastereomer) measured in C_6D_6 at 600 MHz.

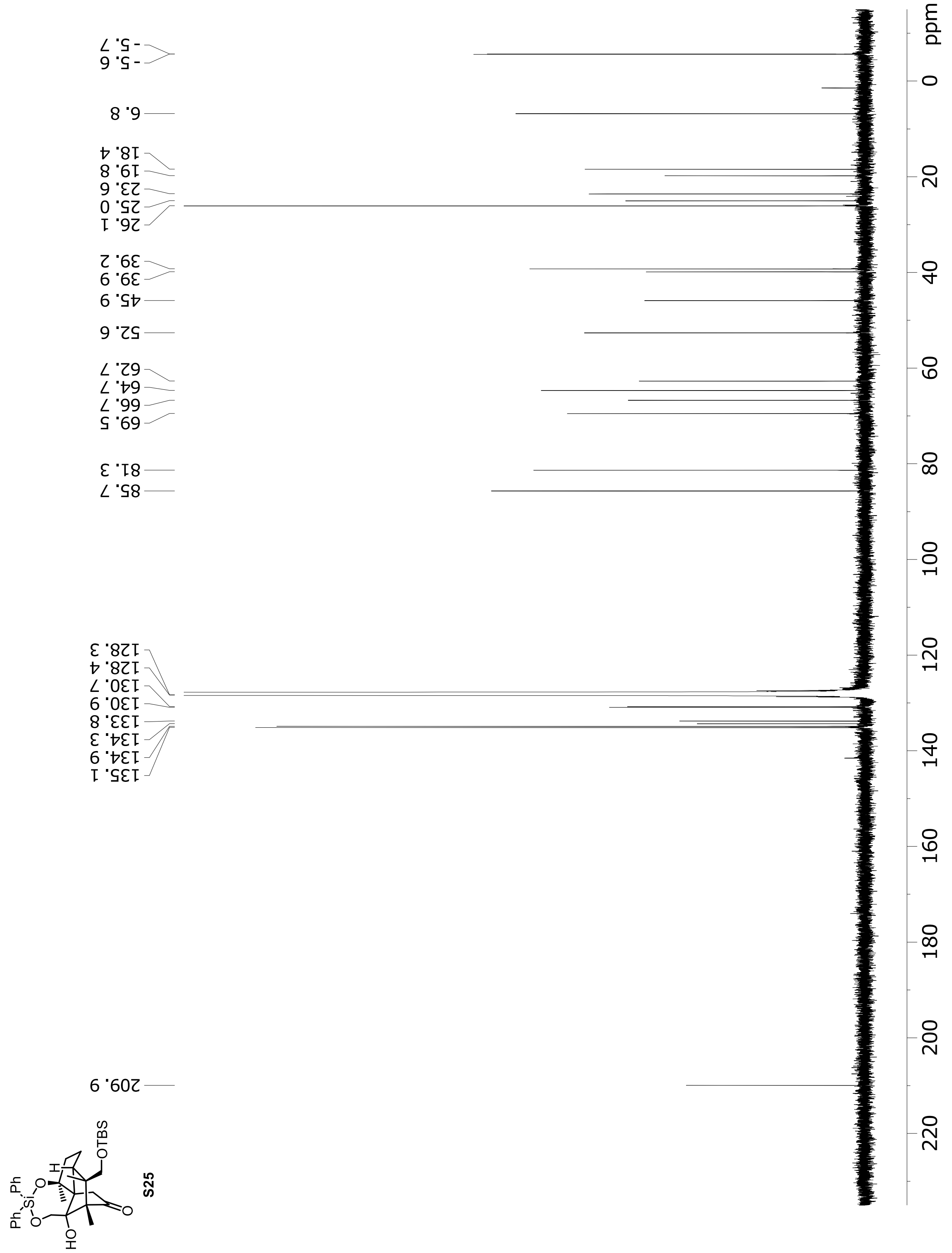

^{13}C NMR spectrum of semipinacol rearrangement product **S25** (desired diastereomer) measured in C_6D_6 at 151 MHz.

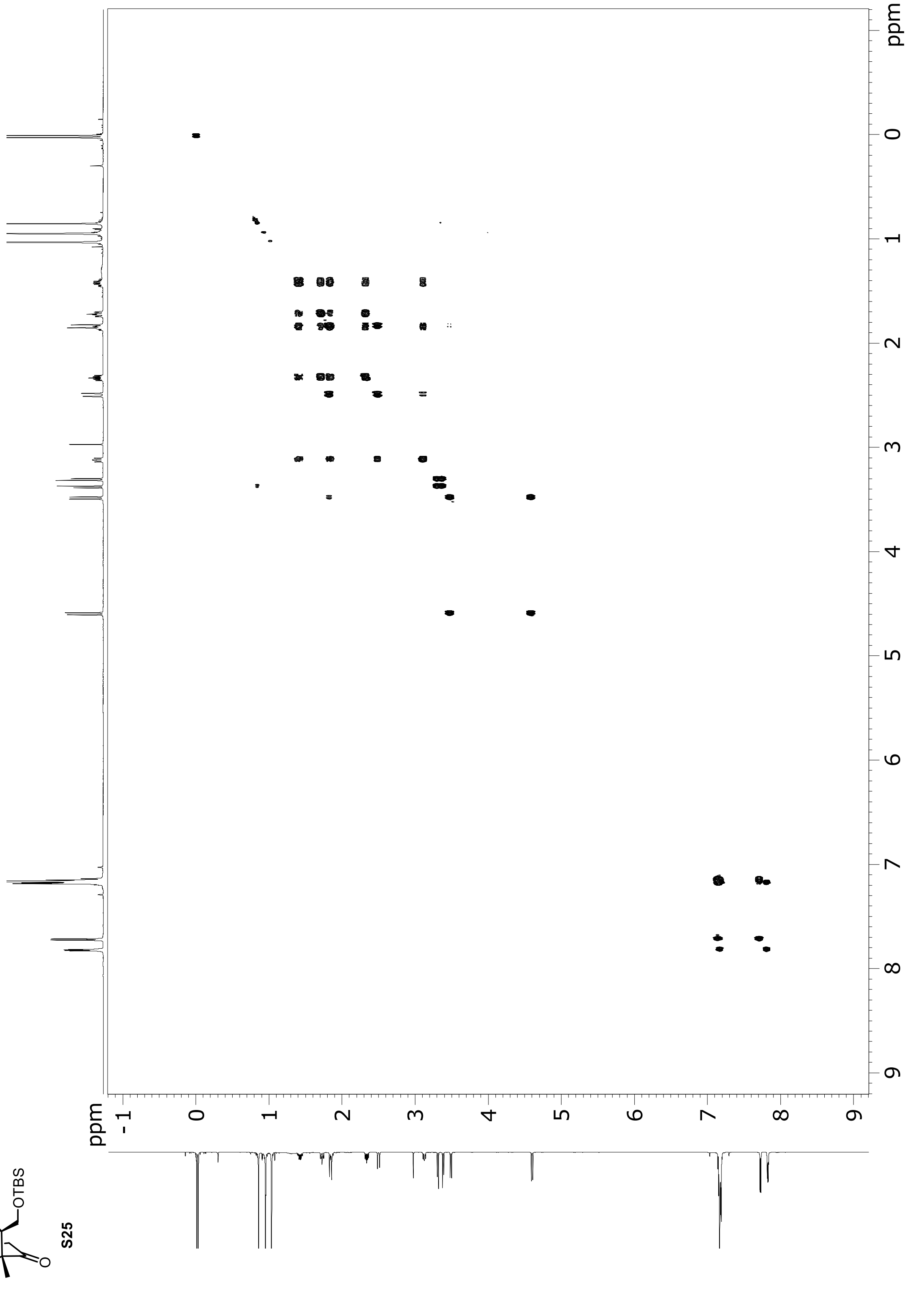

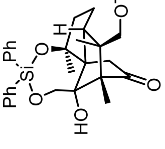

S25

COSY spectrum of semipinacol rearrangement product **S25** (desired diastereomer) measured in C_6D_6 at 600 MHz.

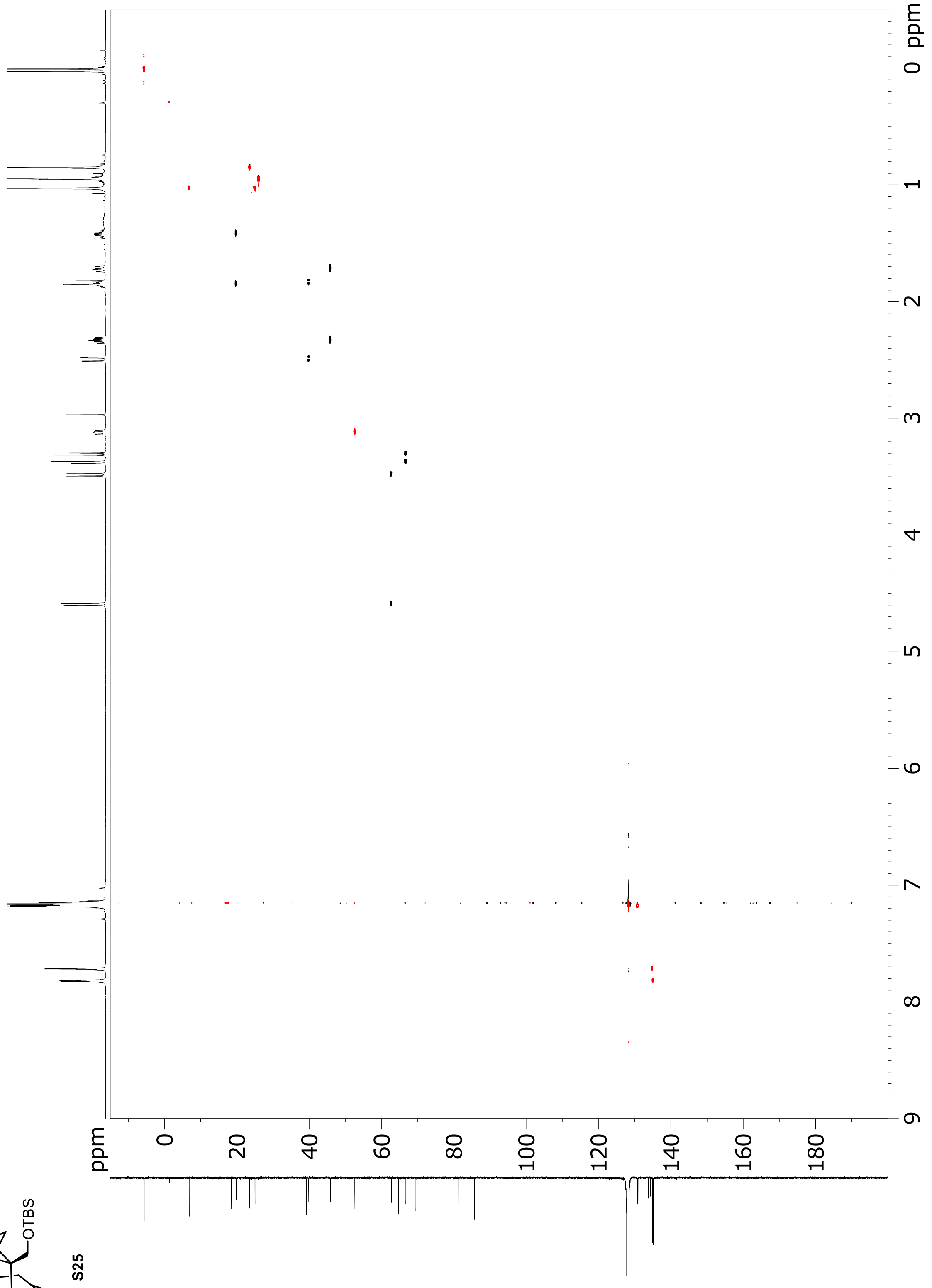

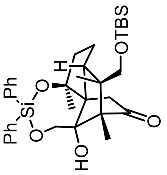

S25

HSQC spectrum of semipinacol rearrangement product **S25** (desired diastereomer) measured in C_6D_6 at 600 MHz.

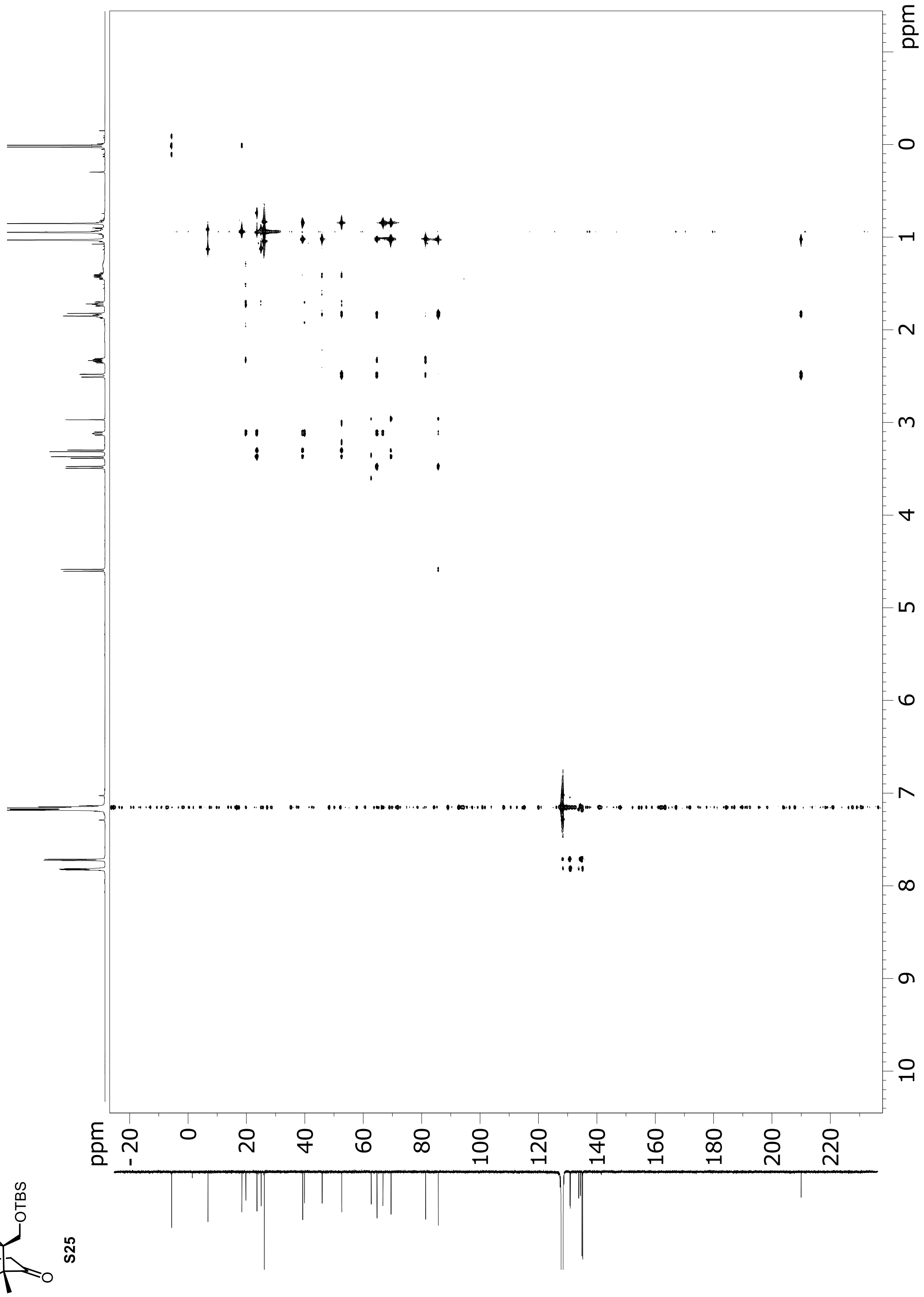

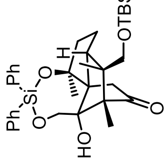

S25

HMBC spectrum of semipinacol rearrangement product **S25** (desired diastereomer) measured in C_6D_6 at 600 MHz.

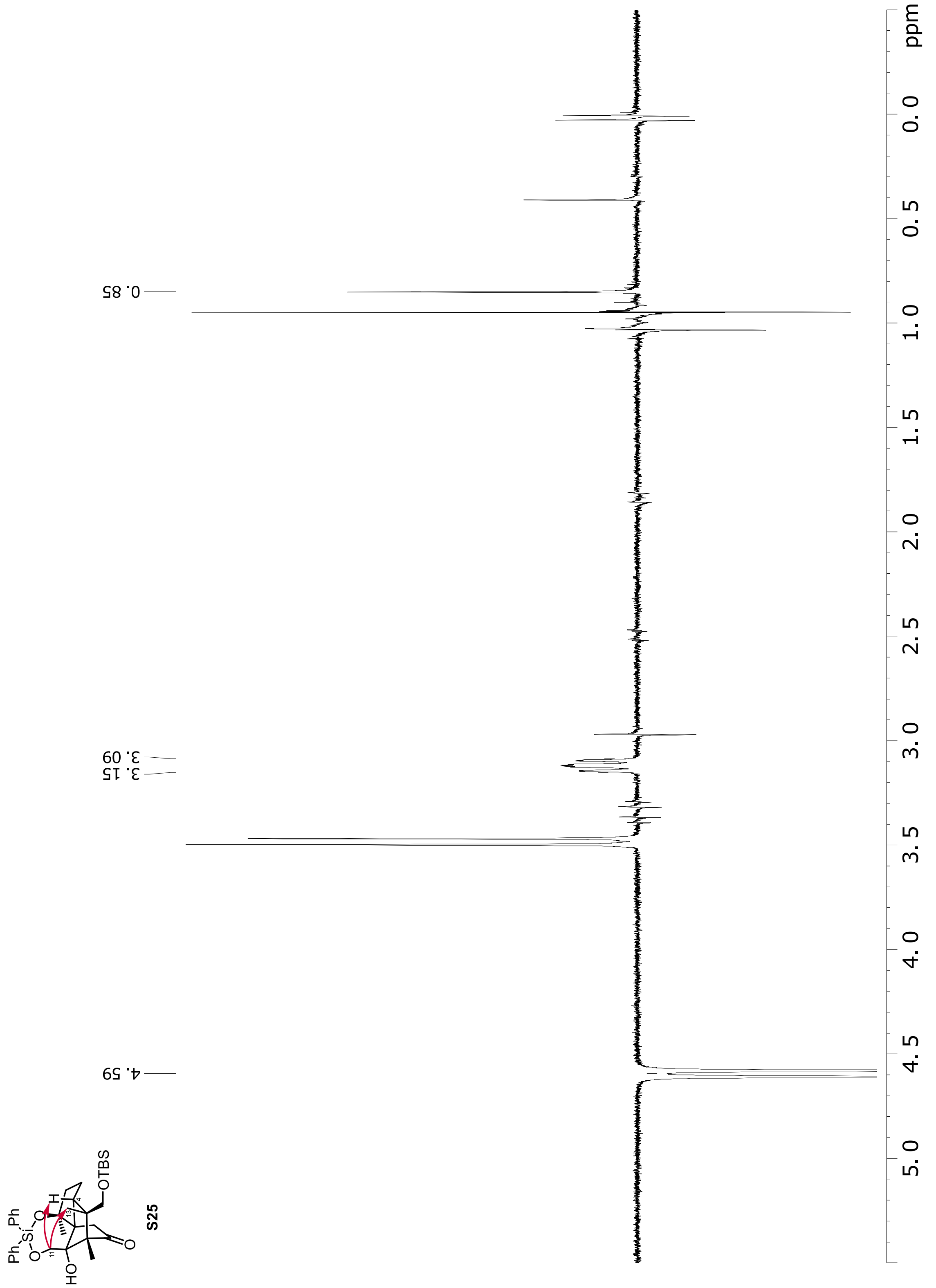

^{1}H-NOE spectrum of semipinacol rearrangement product **S25** after irradiation at 4.59 ppm (CH_2-11a), measured in C_6D_6 at 400 MHz.

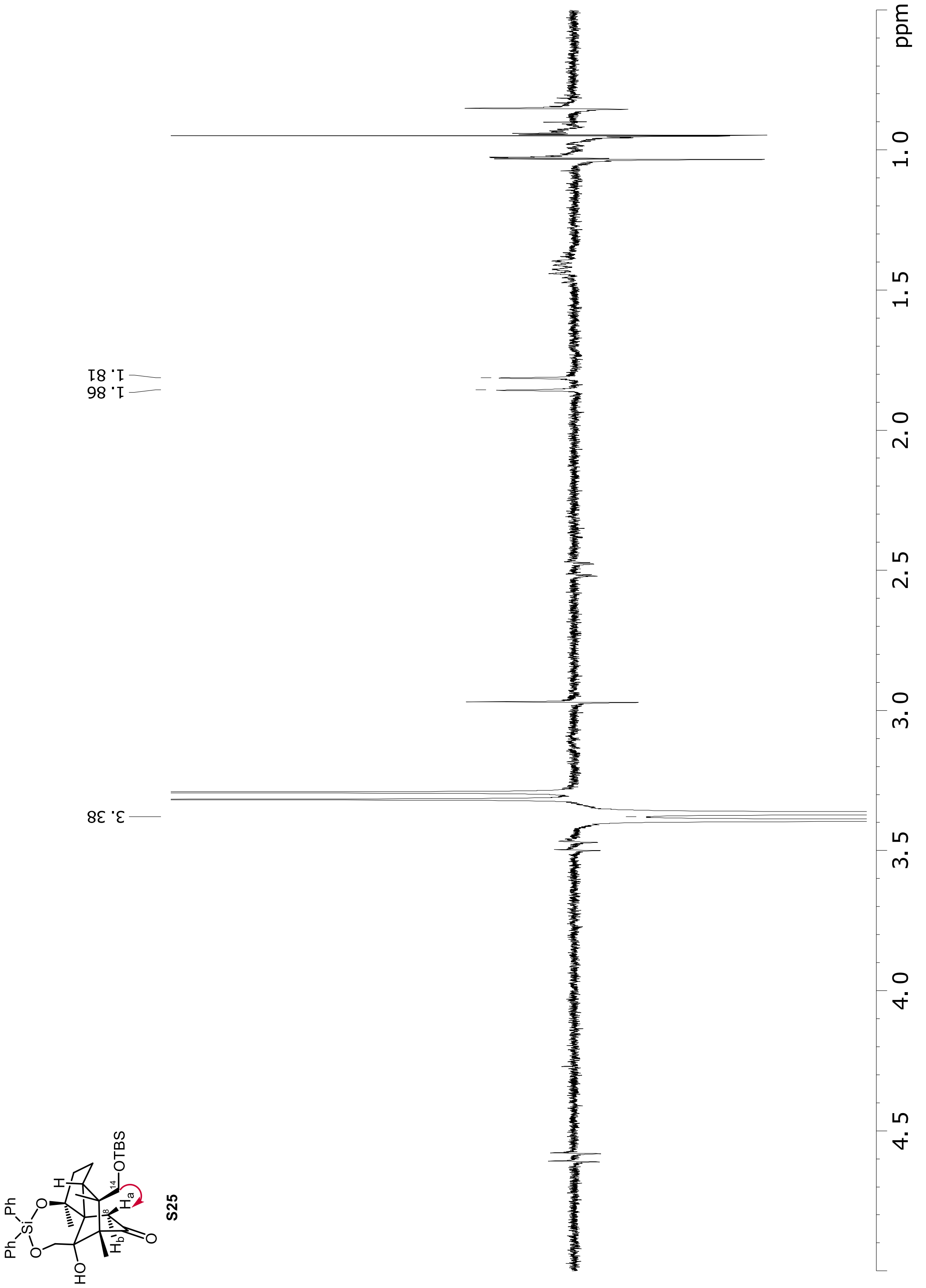

^{1}H-NOE spectrum of semipinacol rearrangement product **S25** after irradiation at 3.38 ppm (CH_2-14a), measured in C_6D_6 at 400 MHz.

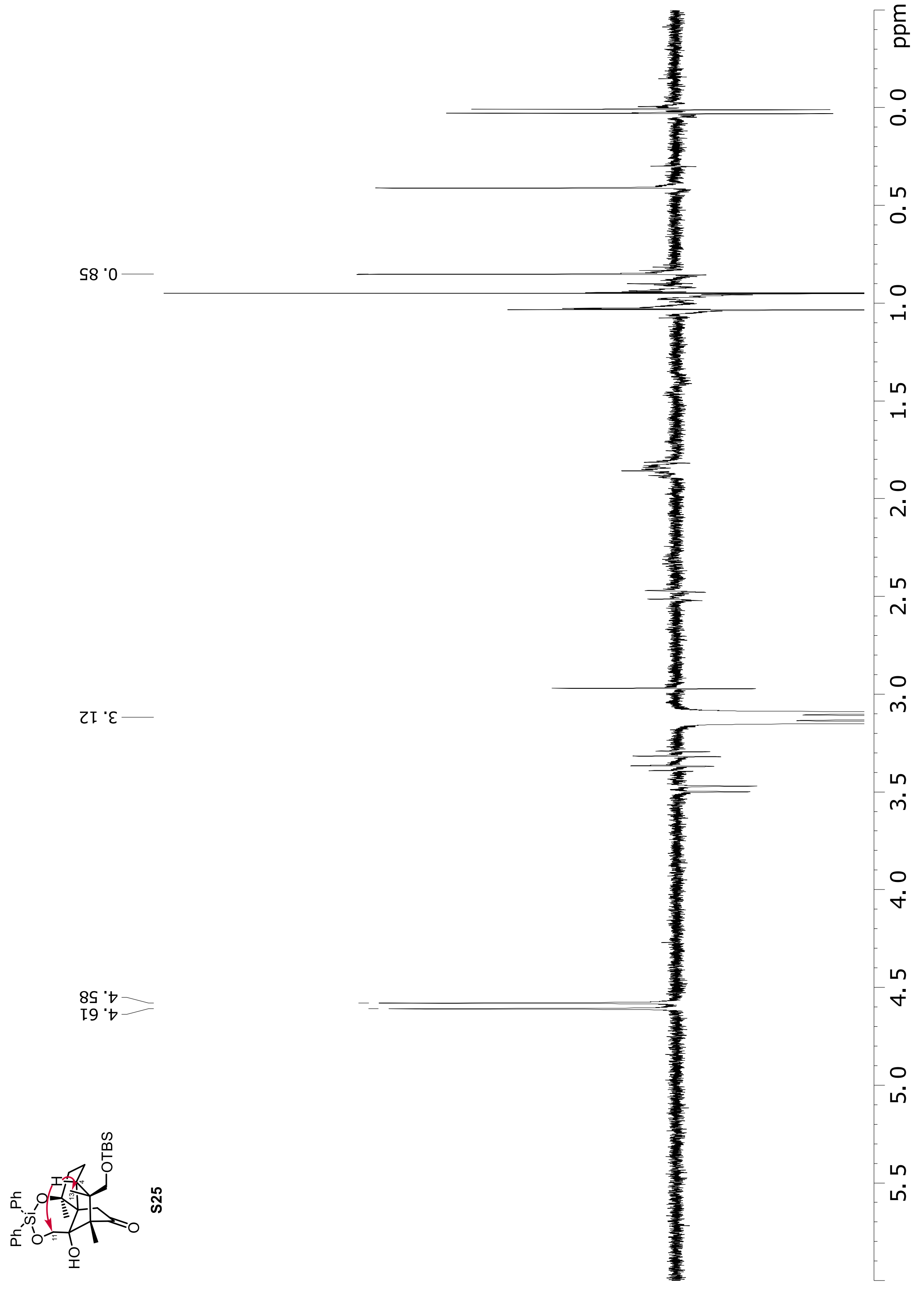

^{1}H-NOE spectrum of semipinacol rearrangement product **S25** after irradiation at 3.12 ppm (CH-4), measured in C_6D_6 at 400 MHz.

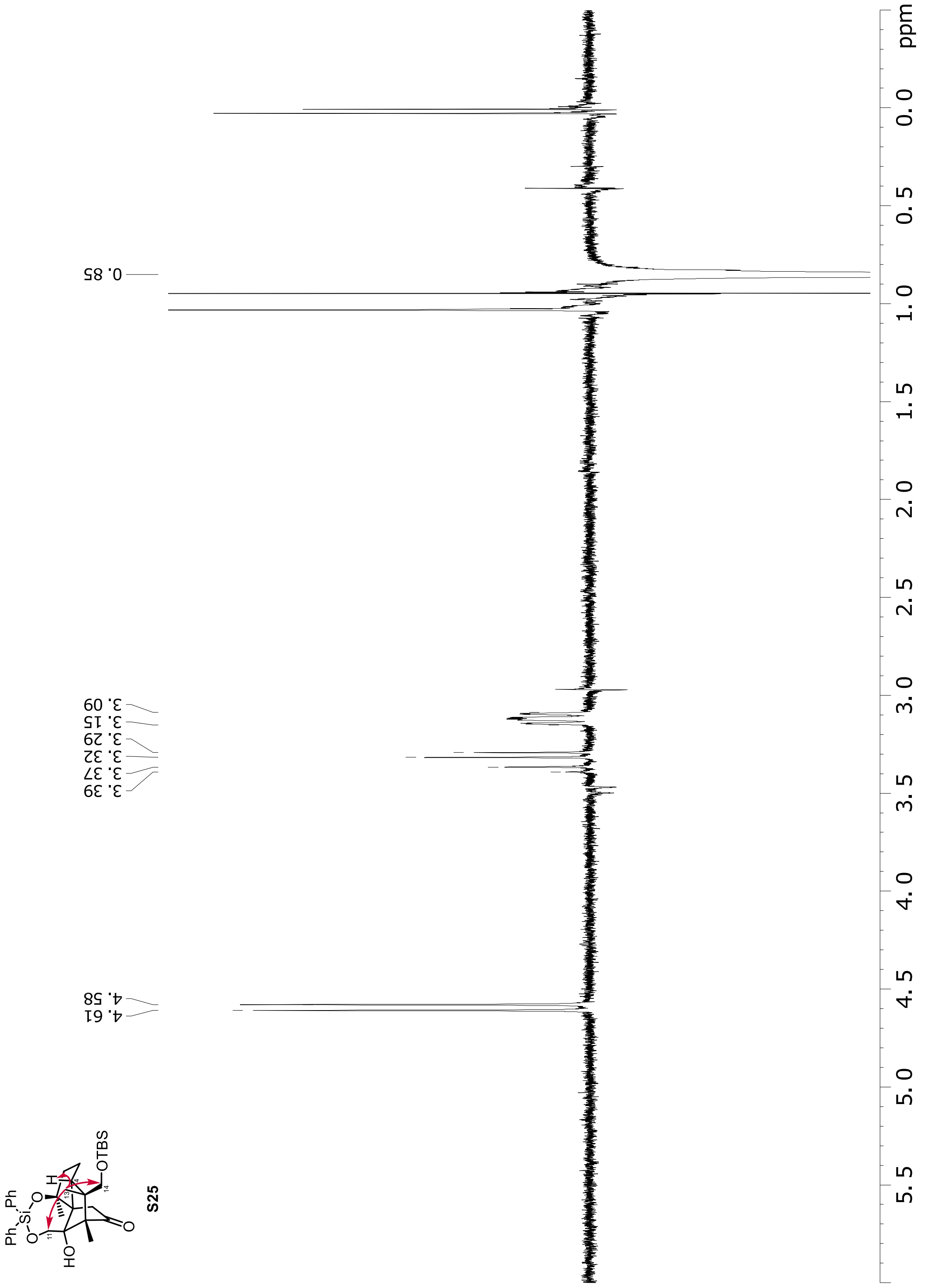

[1]**H-NOE spectrum** of semipinacol rearrangement product **S25** after irradiation at 0.85 ppm (CH_3-13), measured in C_6D_6 at 400 MHz.

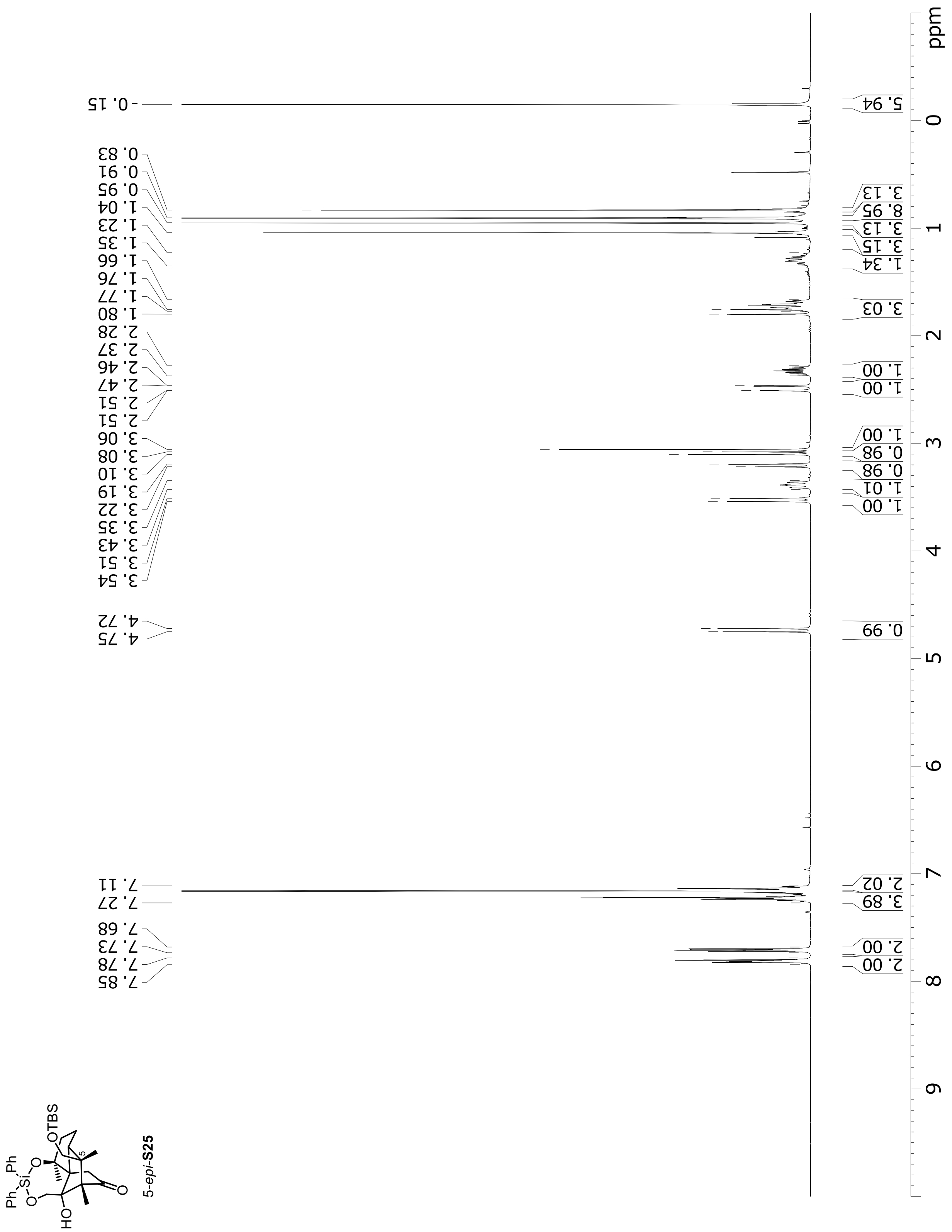

^{1}H NMR spectrum of semipinacol rearrangement product 5-*epi*-**S25** (undesired diastereomer) measured in C_6D_6 at 400 MHz.

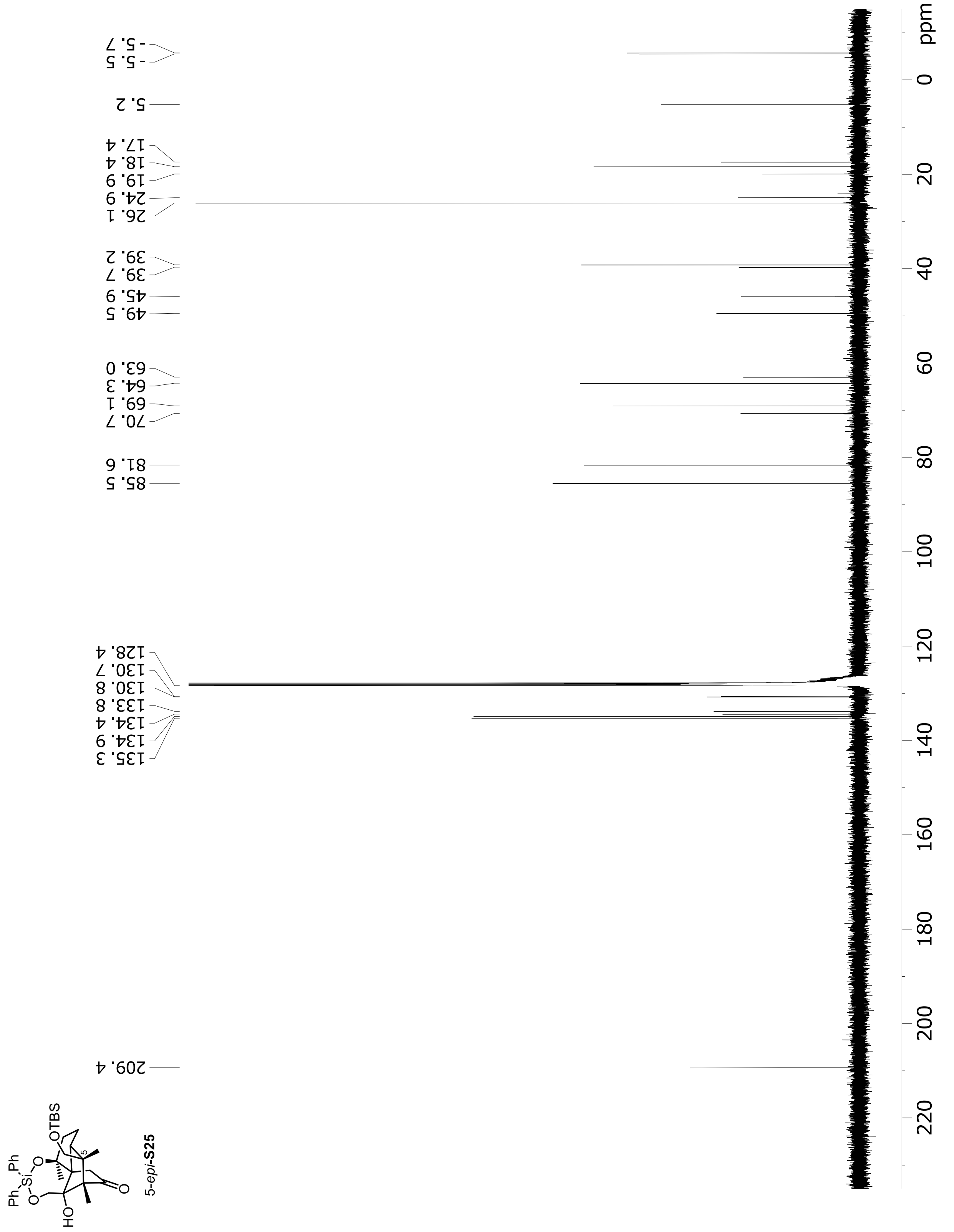

^{13}C NMR spectrum of semipinacol rearrangement product 5-epi-**S25** (undesired diastereomer) measured in C_6D_6 at 101 MHz.

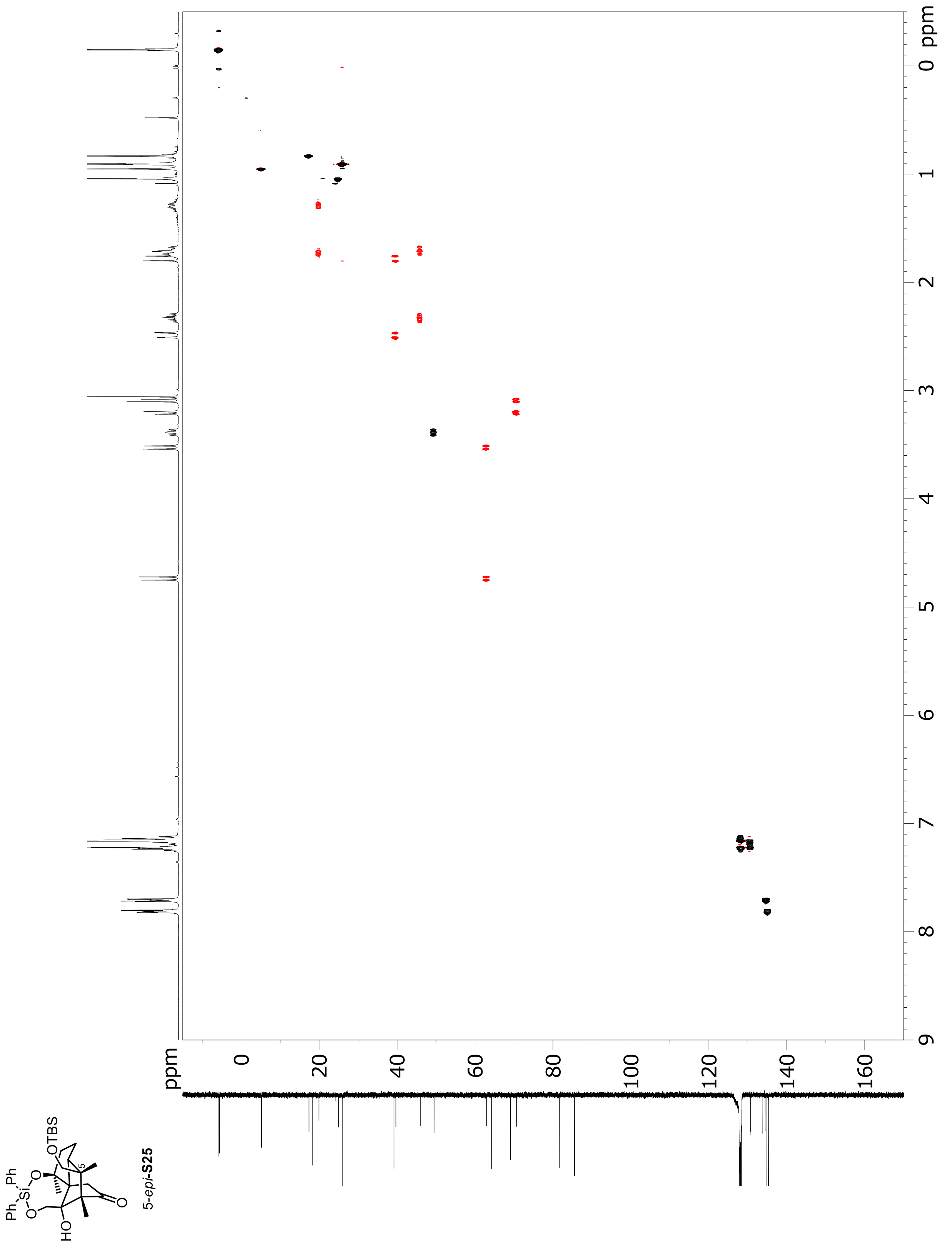

HSQC NMR spectrum of semipinacol rearrangement product 5-*epi*-**S25** (undesired diastereomer) measured in C_6D_6 at 400 MHz.

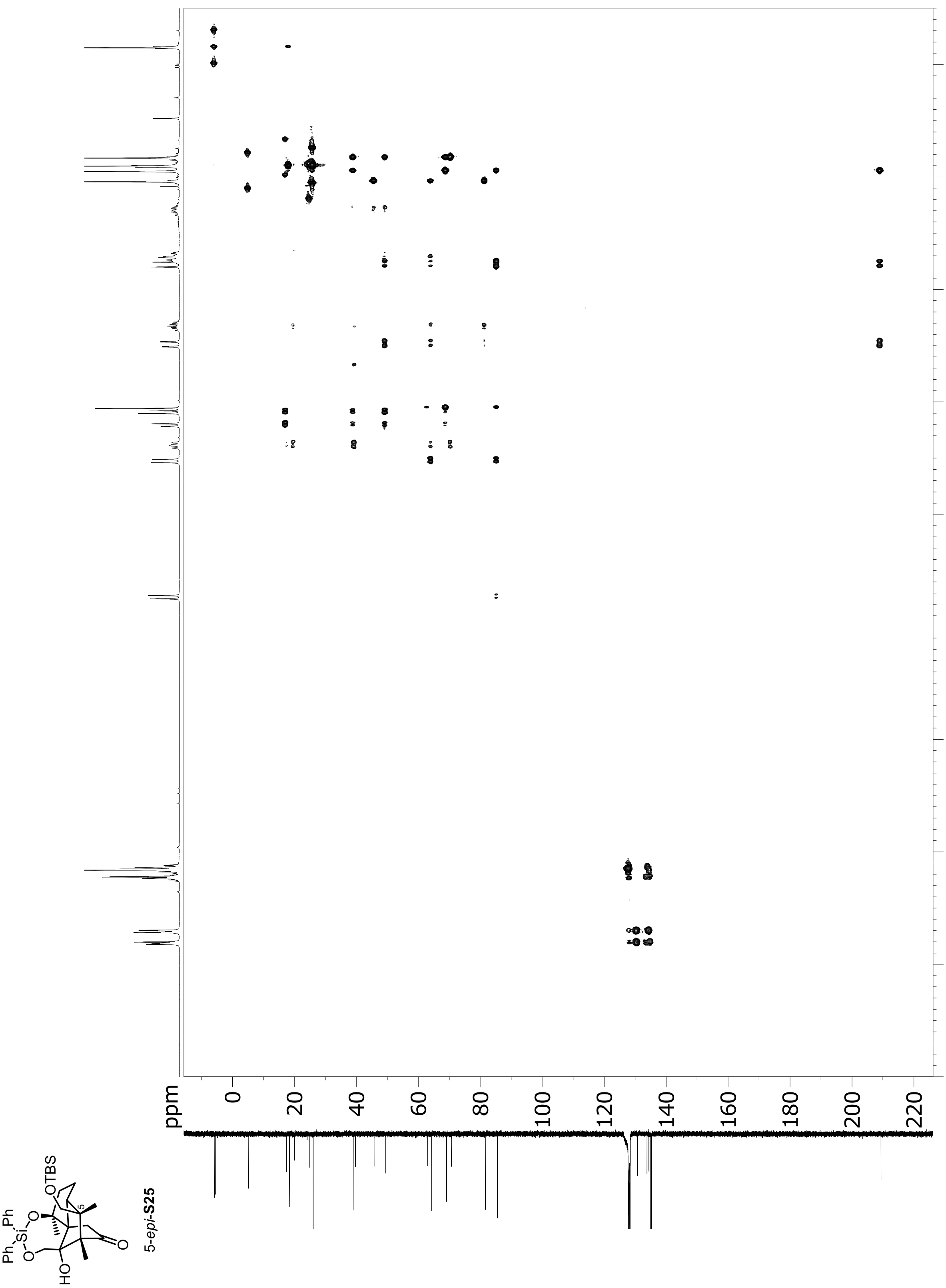

HMBC NMR spectrum of semipinacol rearrangement product 5-*epi*-**S25** (undesired diastereomer) measured in C_6D_6 at 400 MHz.

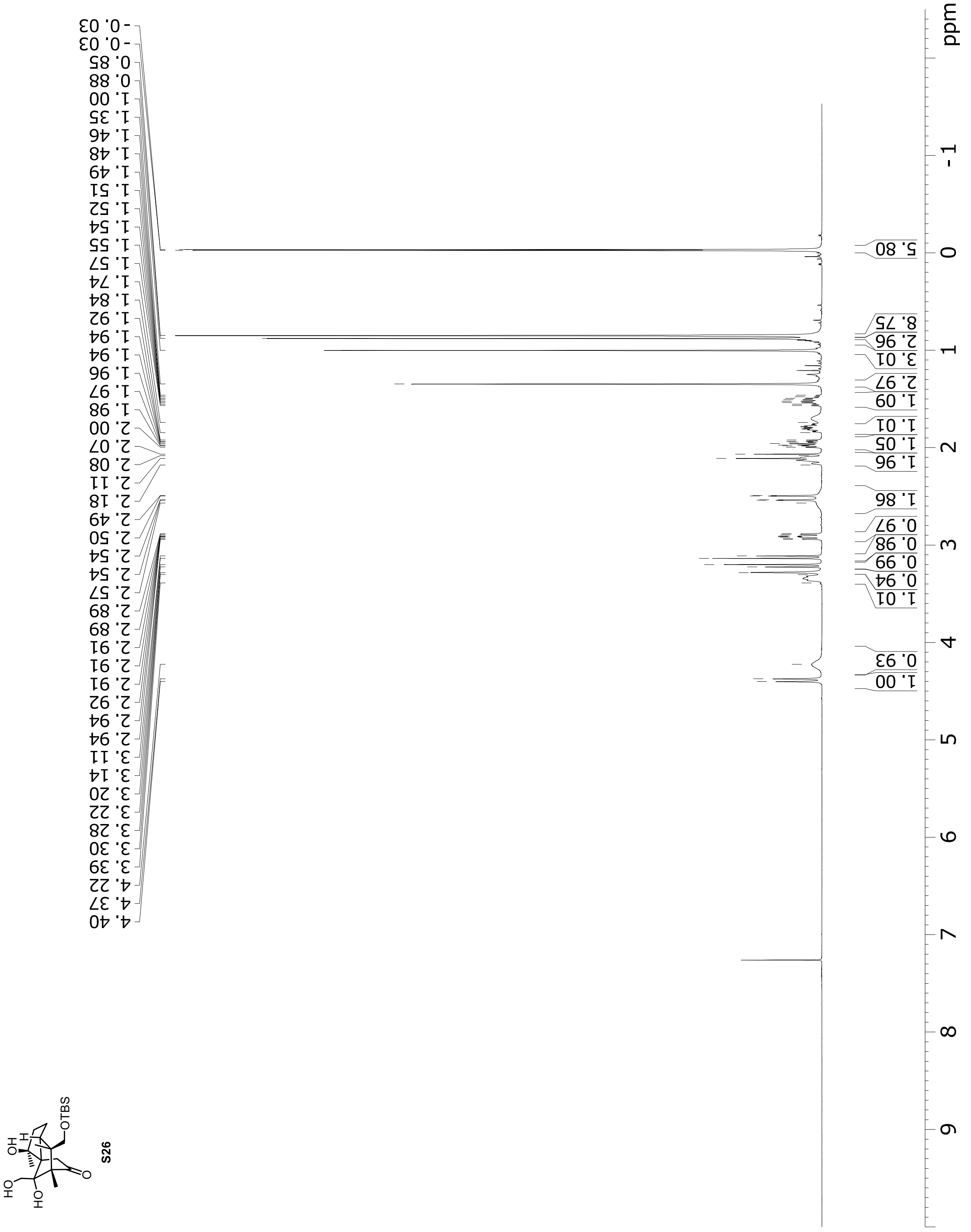

^{1}**H NMR spectrum** of Rychnovsky and Burns’ intermediate **S26** measured in $CDCl_3$ at 400 MHz.

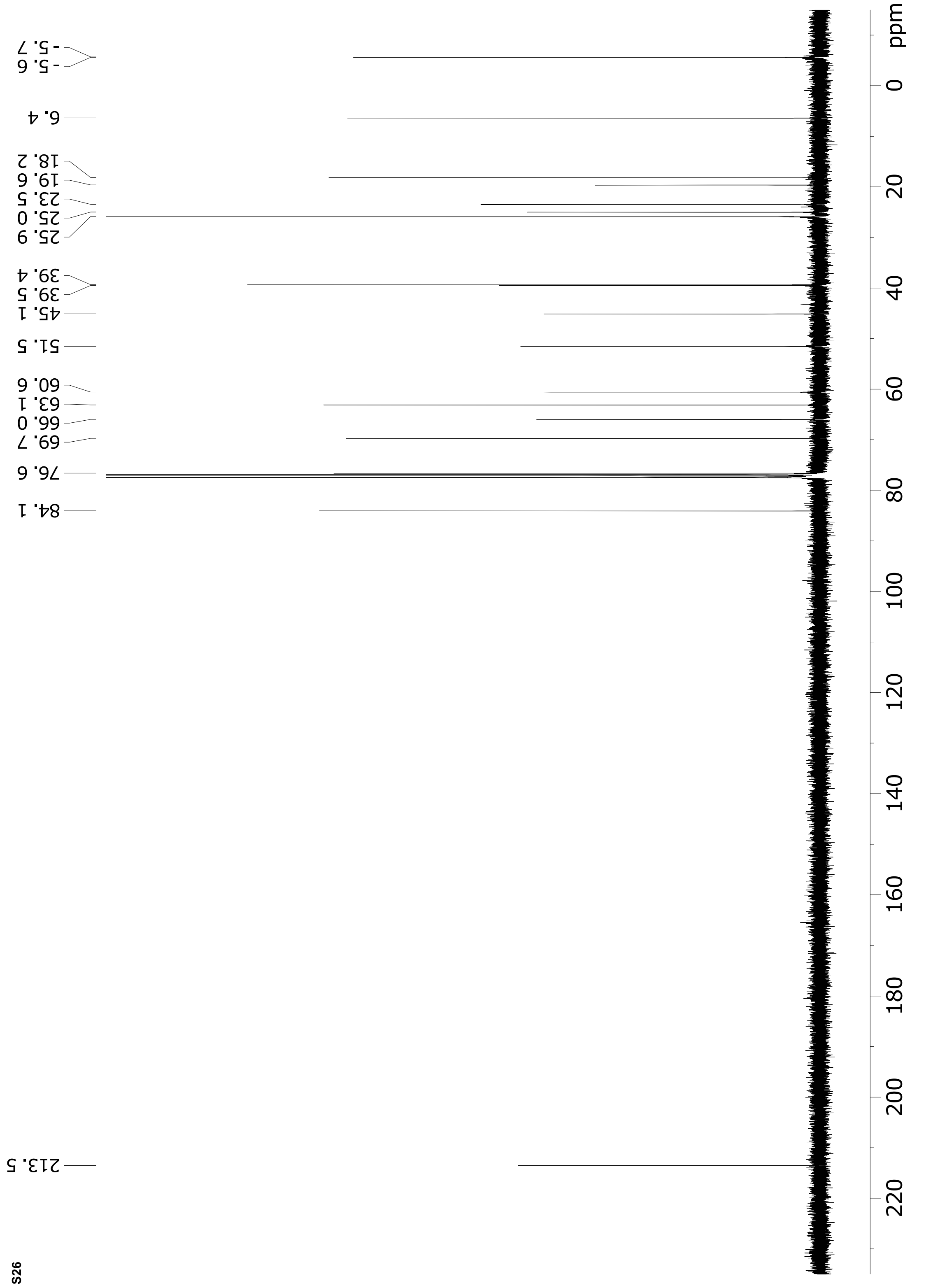

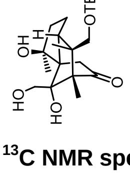

S26

^{13}C NMR spectrum of Rychnovsky and Burns' intermediate **S26** measured in $CDCl_3$ at 101 MHz.

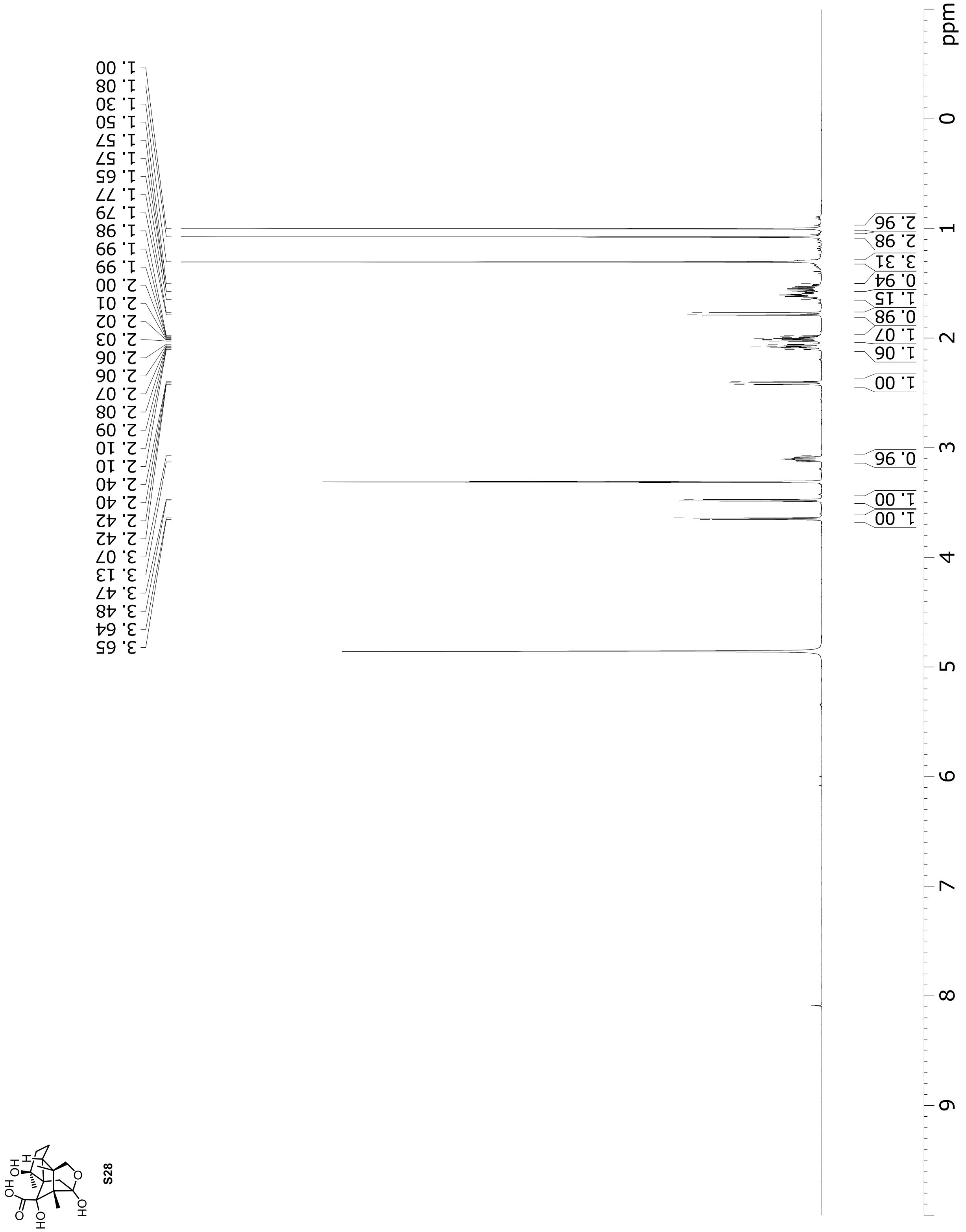

^{1}H NMR spectrum of carboxylic acid **S28** measured in methanol-d_4 at 600 MHz.

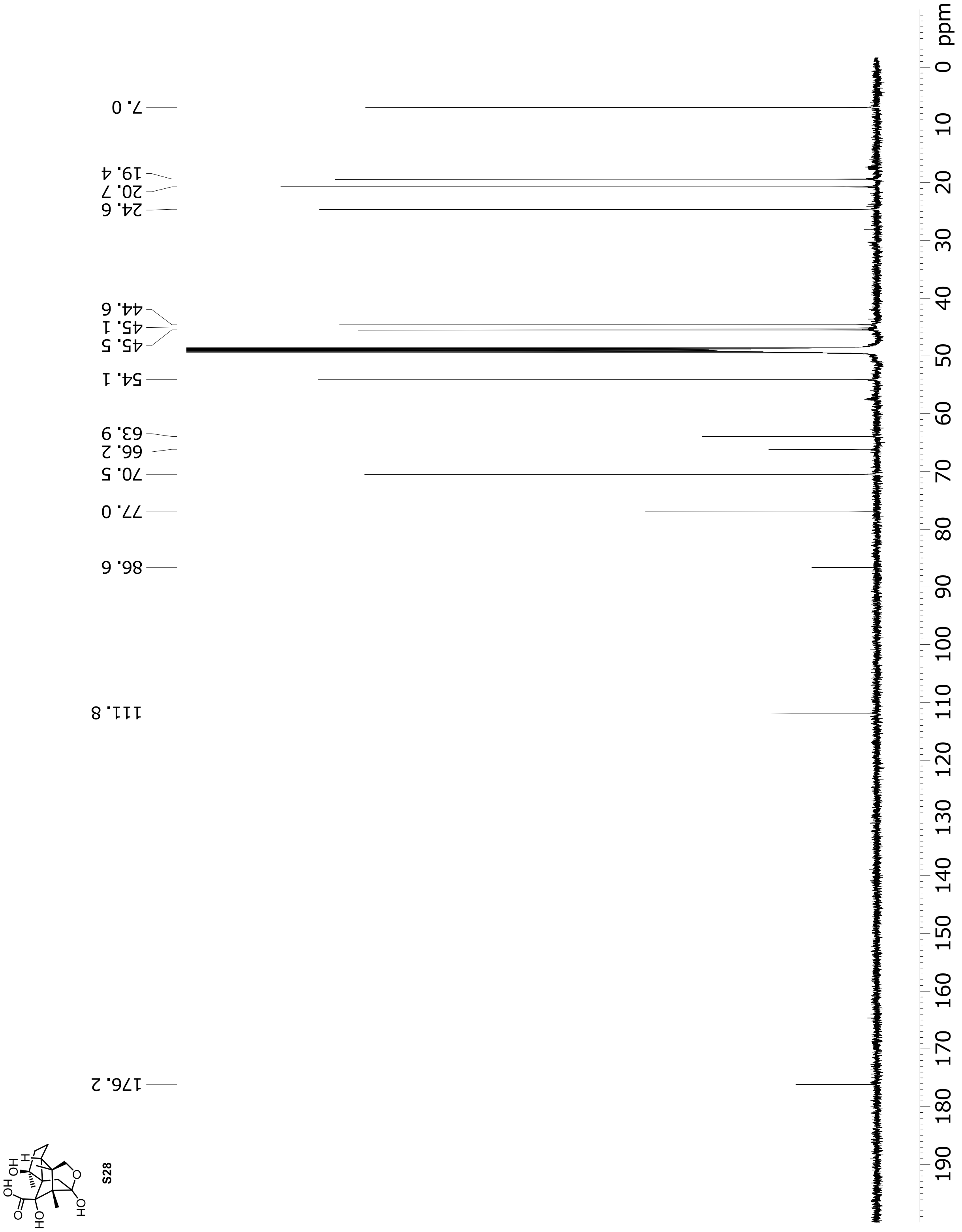

^{13}**C NMR spectrum** of carboxylic acid **S28** measured in methanol-d_4 at 151 MHz.

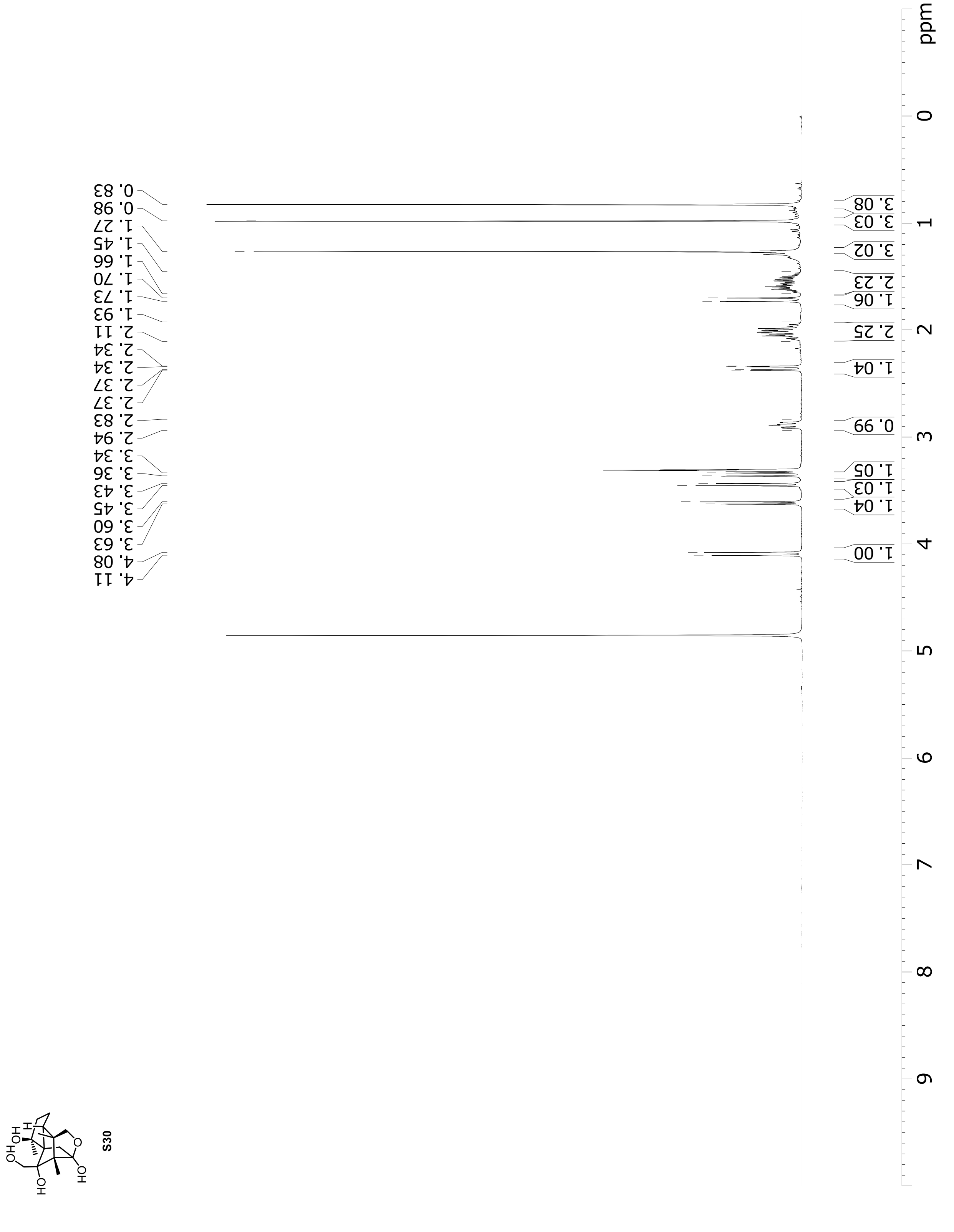

^{1}H NMR spectrum of lactol **S30** measured in methanol-d_4 at 400 MHz.

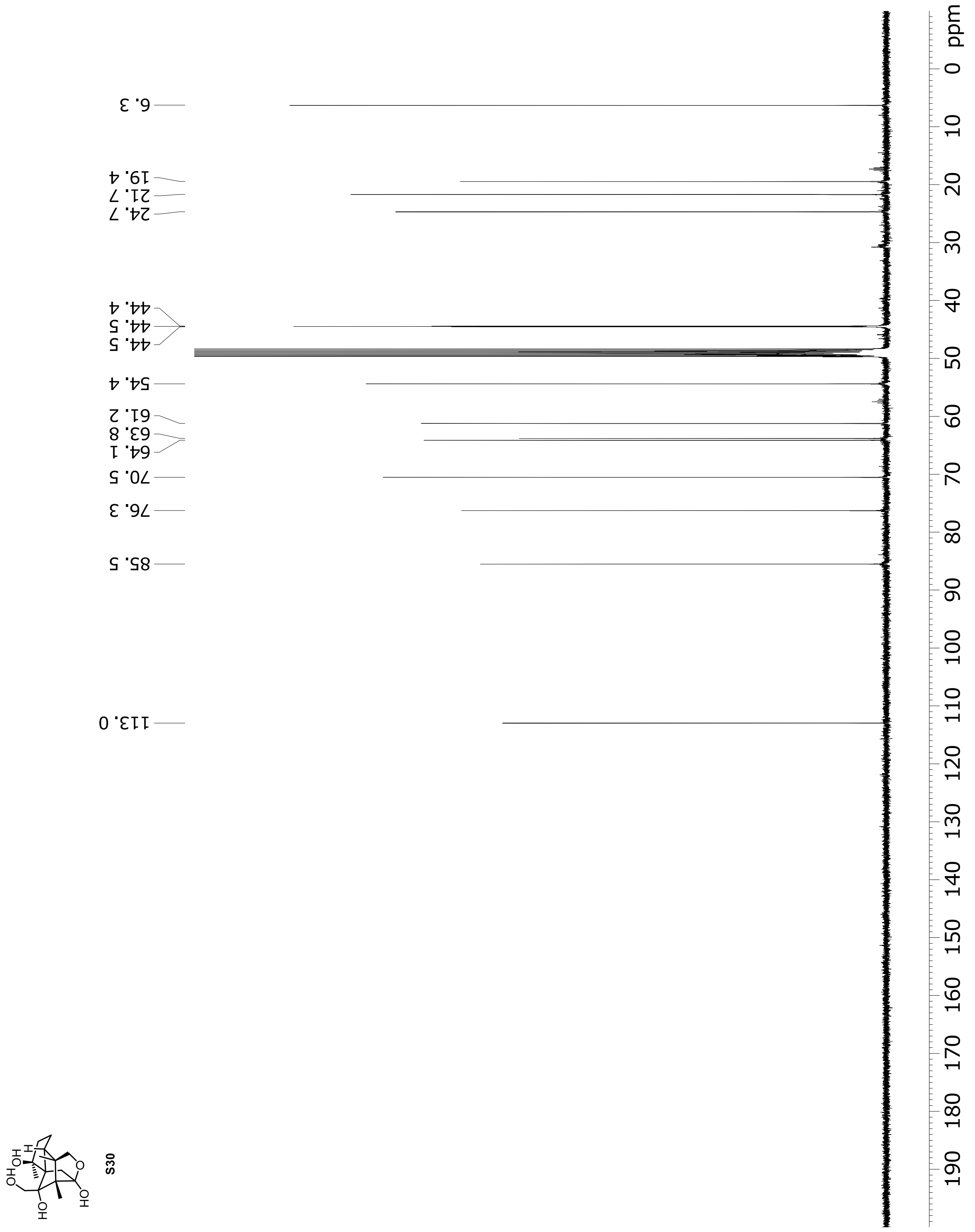

^{13}C NMR spectrum of lactol **S30** measured in methanol-d_4 at 101 MHz.

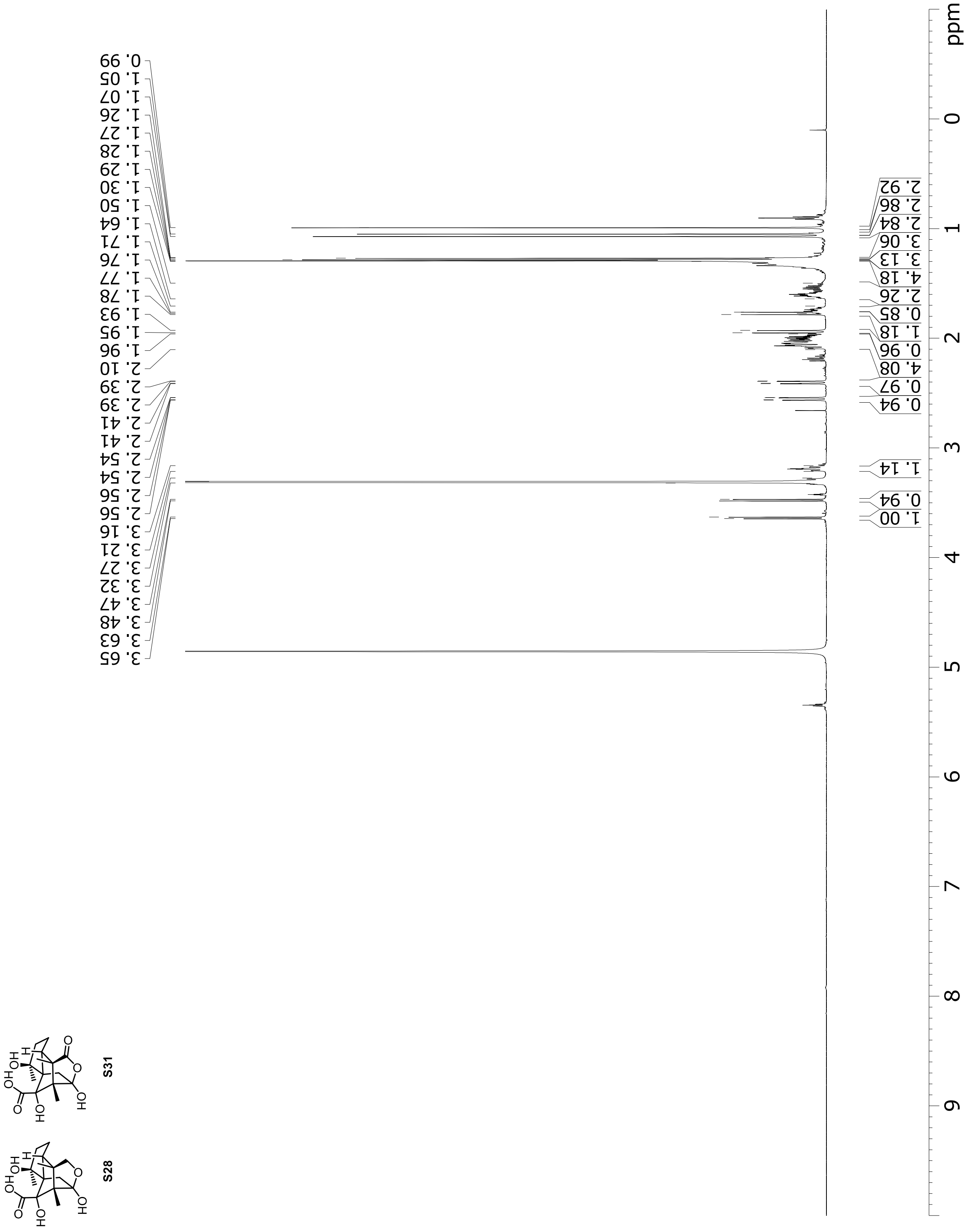

^{1}**H NMR spectrum** of carboxylic acid **S28** and over-oxidized carboxylic acid **S31** measured in methanol-d_4 at 600 MHz. The spectrum shows impurities that can be traced back to the batch of methanol-d_4 used. These signals show significant intensities due to the low amount of substance that was available for the measurement. To clarify the circumstance, a spectrum of the used solvent is given below.

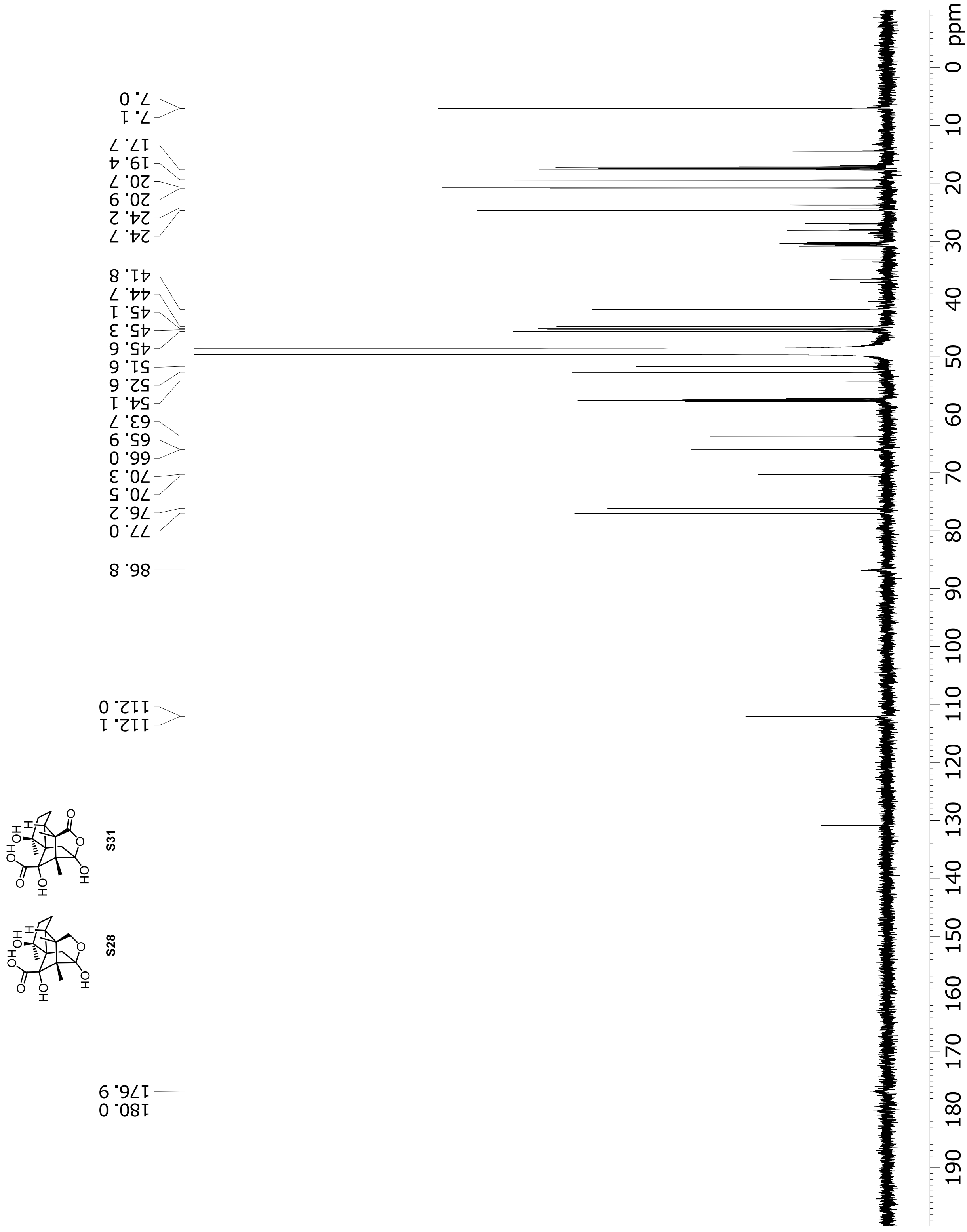

^{13}**C NMR spectrum** of carboxylic acid **S28** and over-oxidized carboxylic acid **S31** measured in methanol-d_4 at 151 MHz. The spectrum shows impurities that can be traced back to the batch of methanol-d_4 used. These signals show significant intensities due to the low amount of substance that was available for the measurement. To clarify the circumstance, a spectrum of the used solvent is given below.

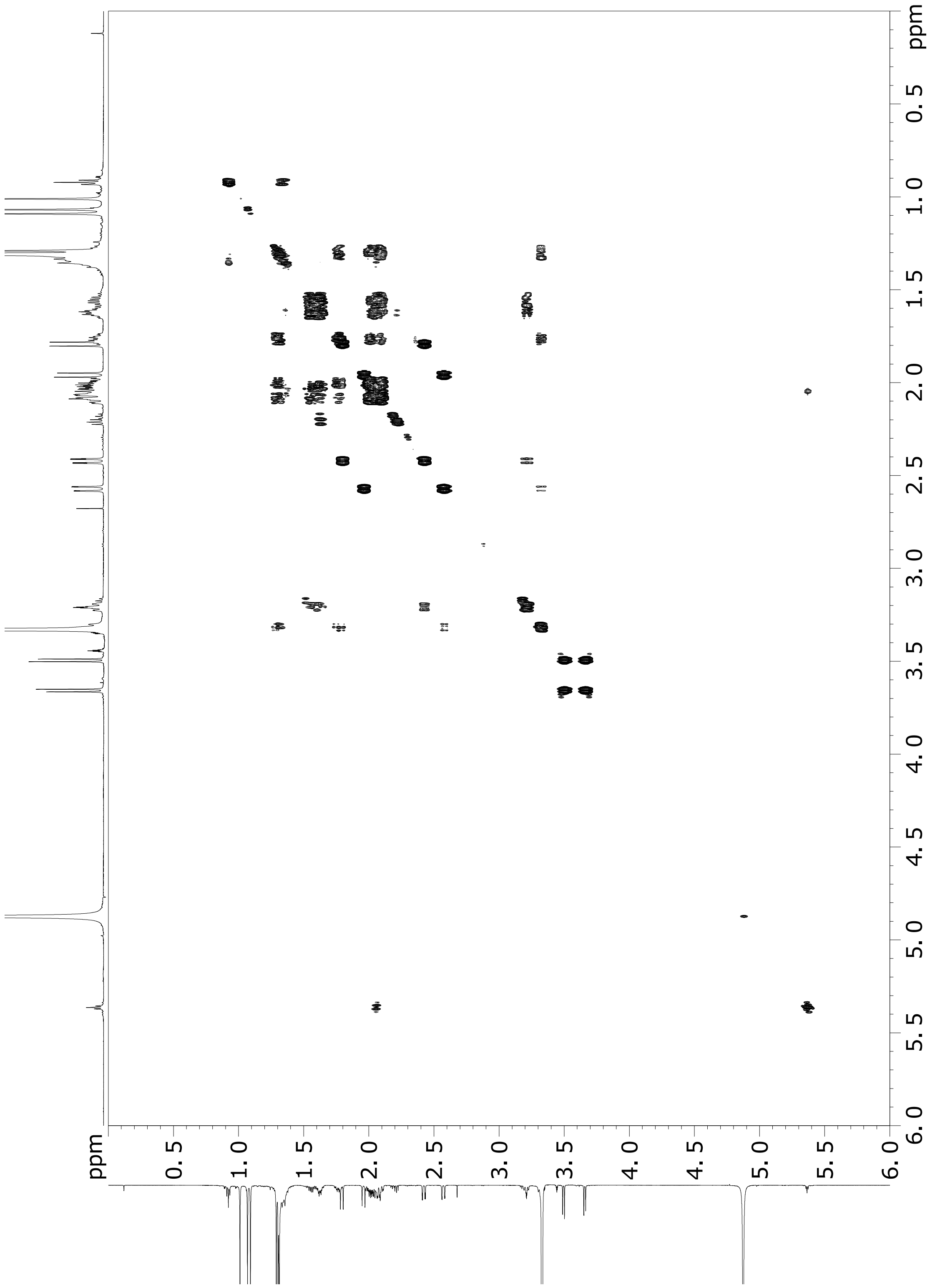

COSY spectrum of carboxylic acid **S28** and over-oxidized carboxylic acid **S31** measured in methanol-d_4 at 600 MHz.

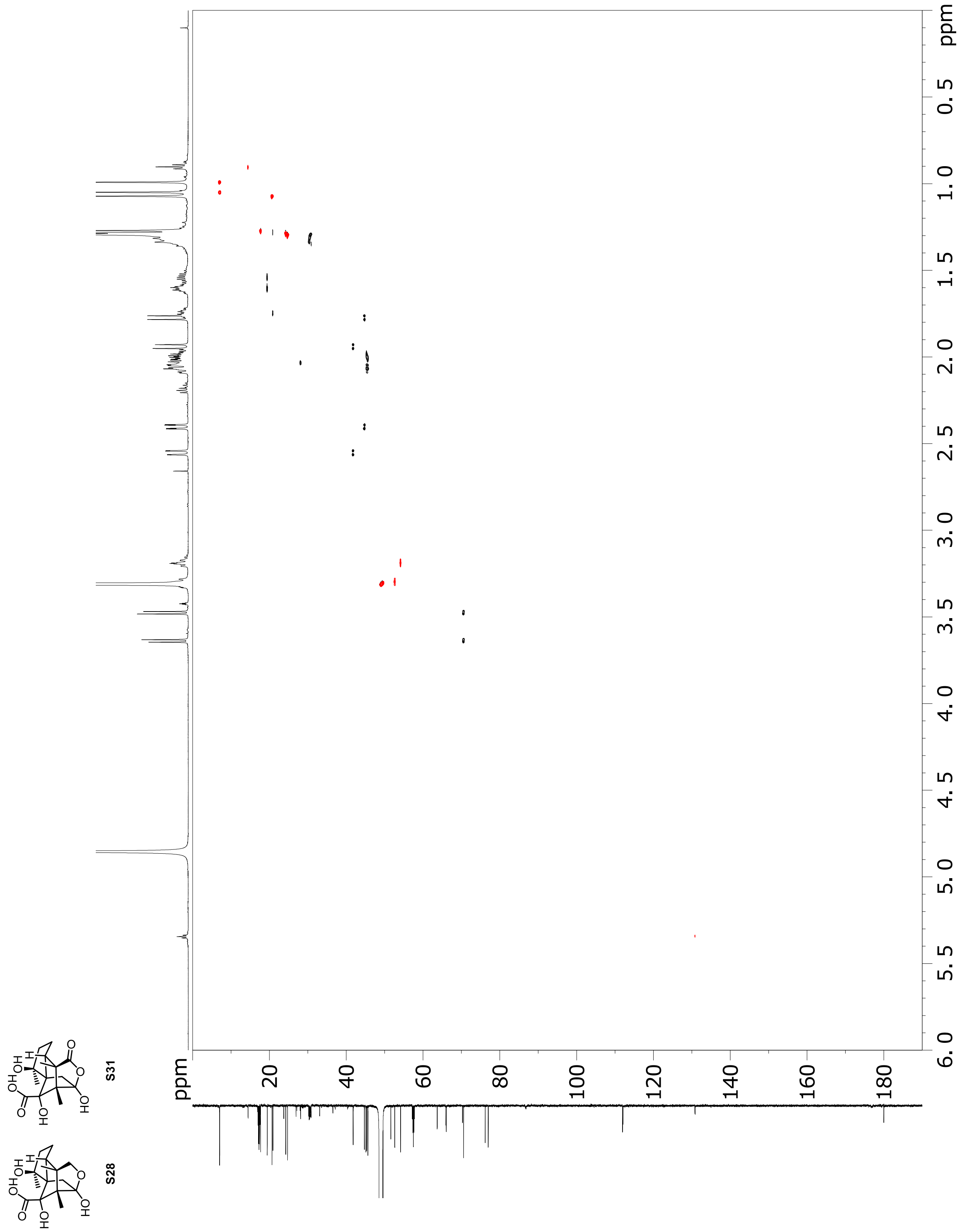

HSQC spectrum of carboxylic acid **S28** and over-oxidized carboxylic acid **S31** measured in methanol-d_4 at 600 MHz.

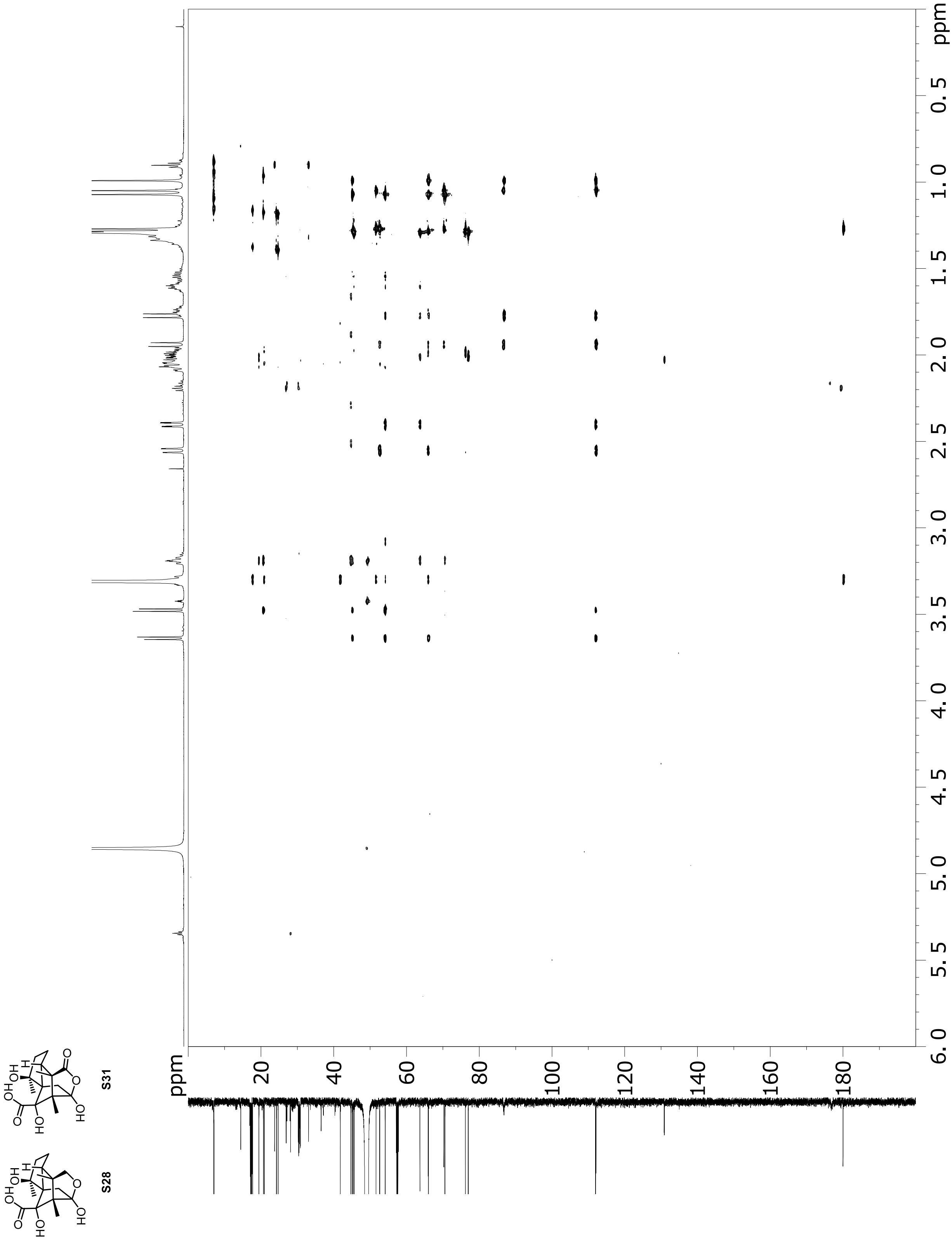

HMBC spectrum of carboxylic acid **S28** and over-oxidized carboxylic acid **S31** measured in methanol-d_4 at 600 MHz.

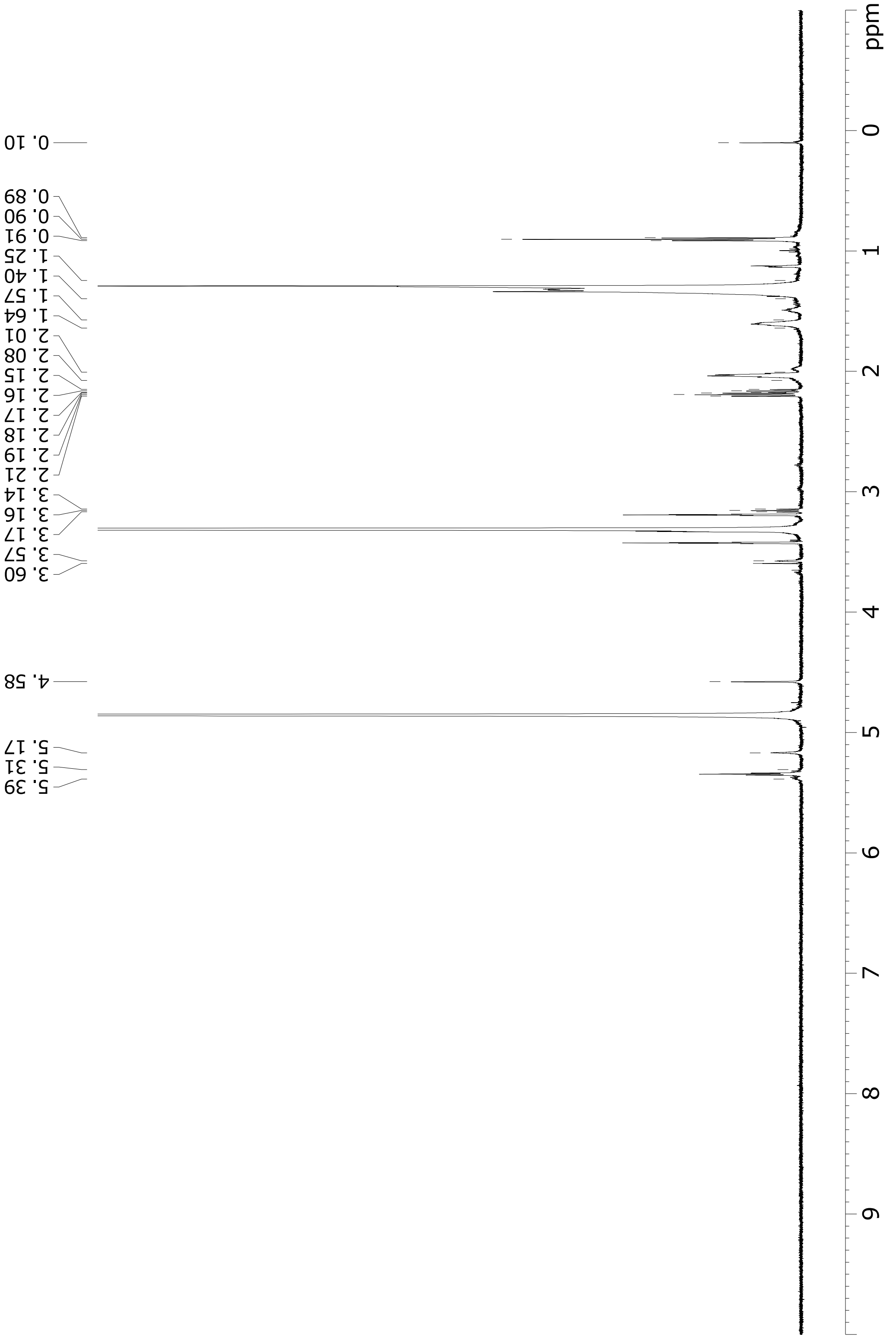

^{1}H NMR spectrum of the **methanol-*d*$_4$** used to measure the NMR spectra of carboxylic acid **S28** and over-oxidized carboxylic acid **S31.** The spectrum was measured at 600 MHz.

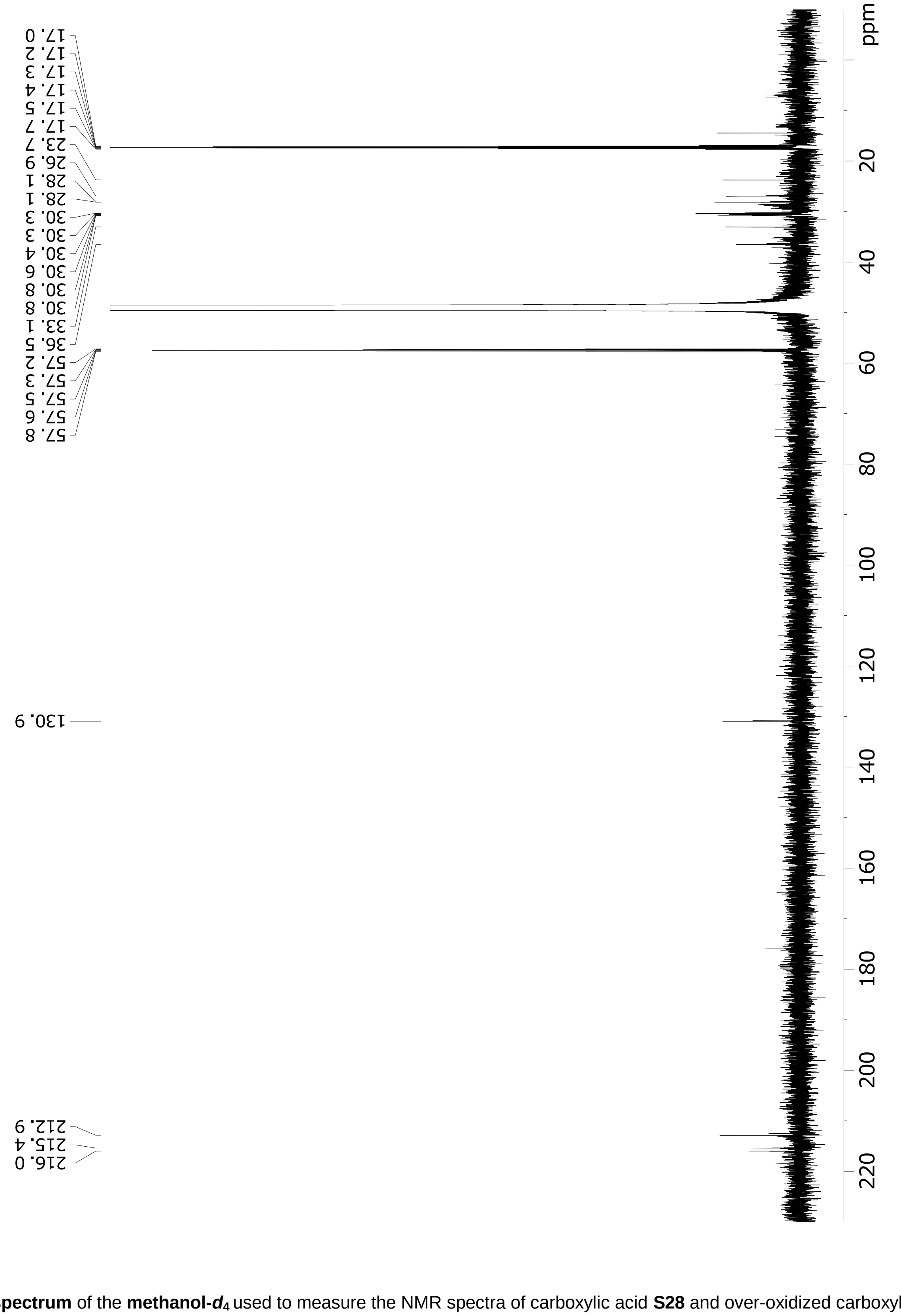

[13]**C NMR spectrum** of the **methanol-*d*$_4$** used to measure the NMR spectra of carboxylic acid **S28** and over-oxidized carboxylic acid **S31.** The spectrum was measured at 151 MHz.

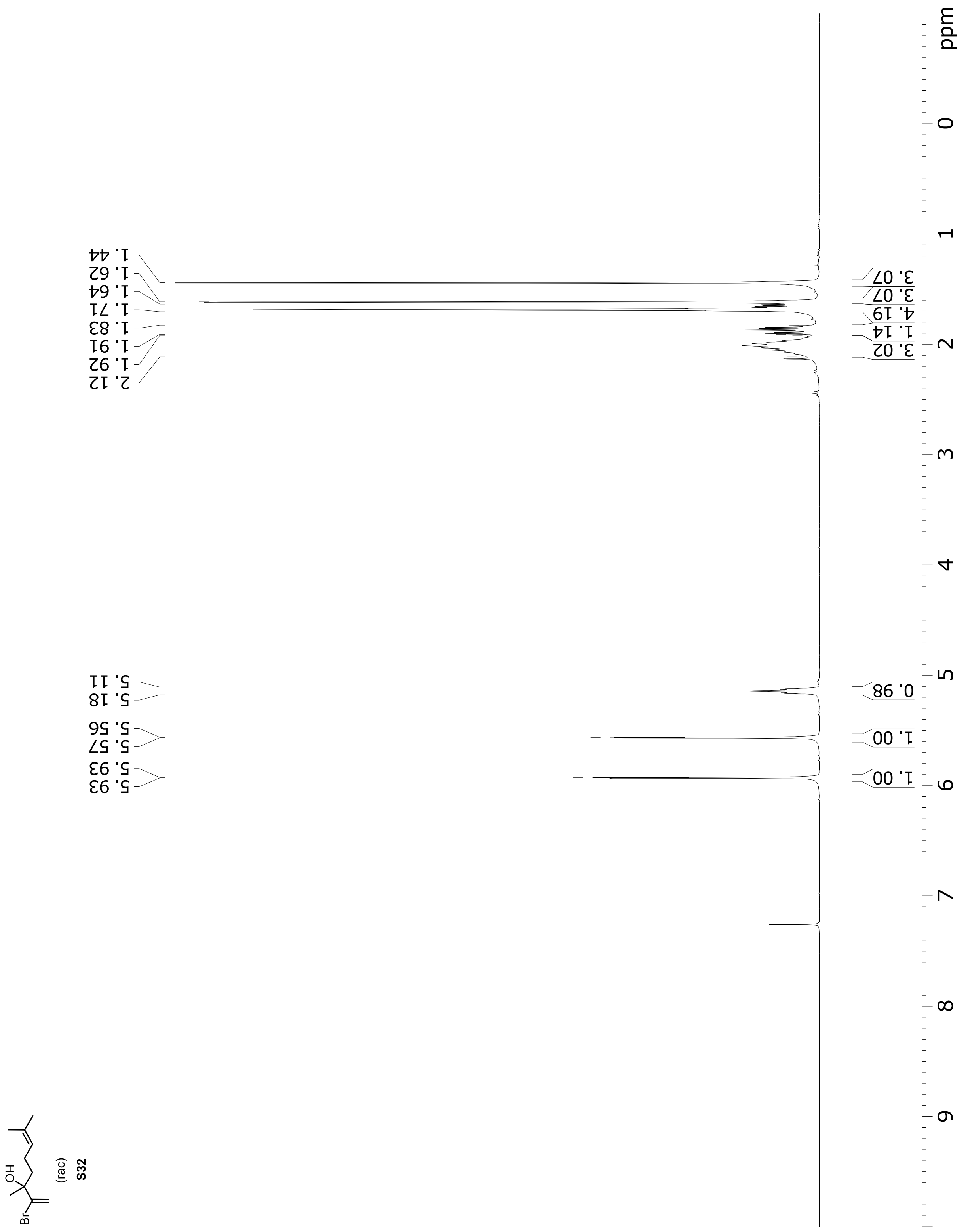

^{1}H NMR spectrum of vinylbromide **S32** measured in $CDCl_3$ at 400 MHz.

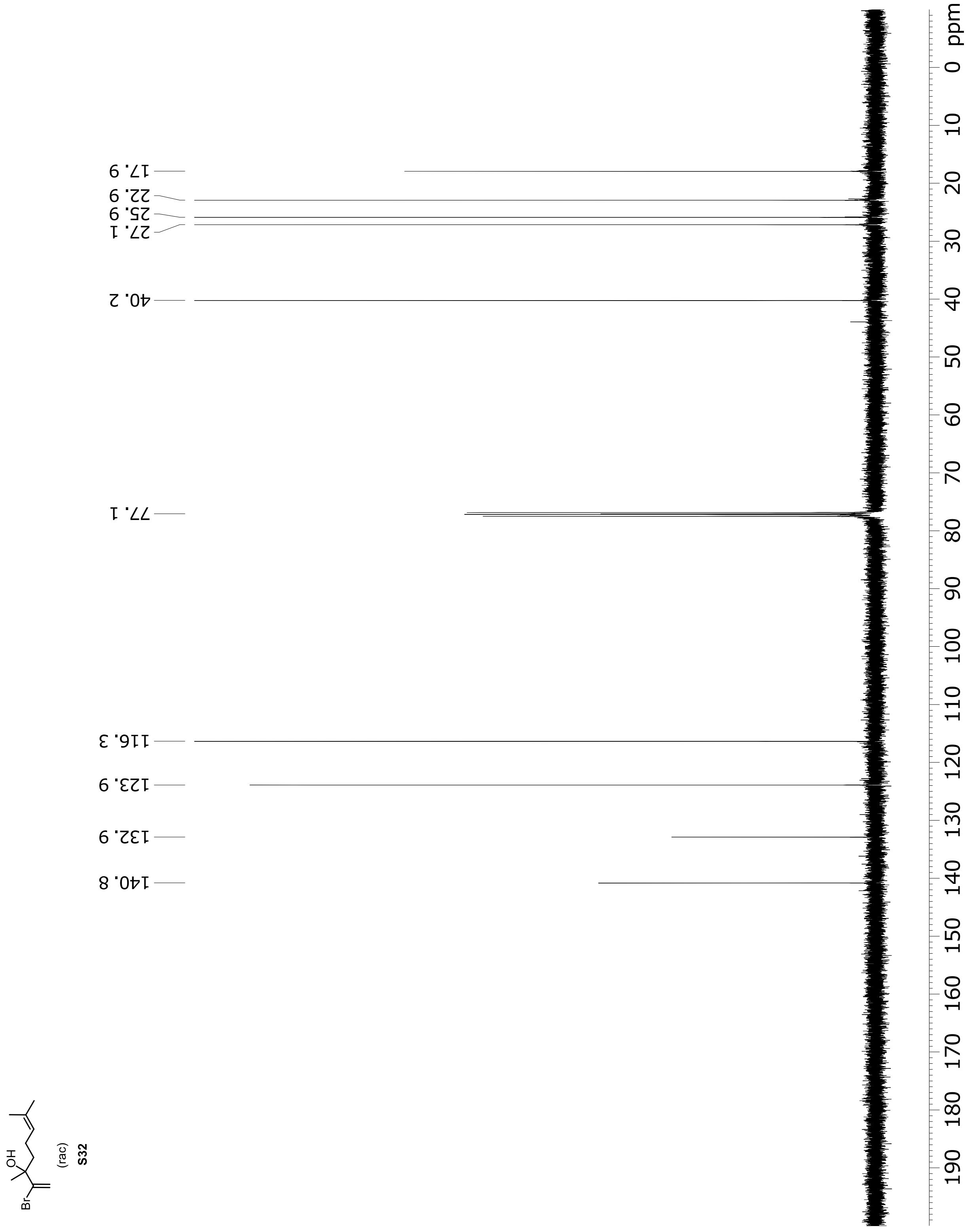

^{13}C NMR spectrum of vinylbromide **S32** measured in $CDCl_3$ at 101 MHz.

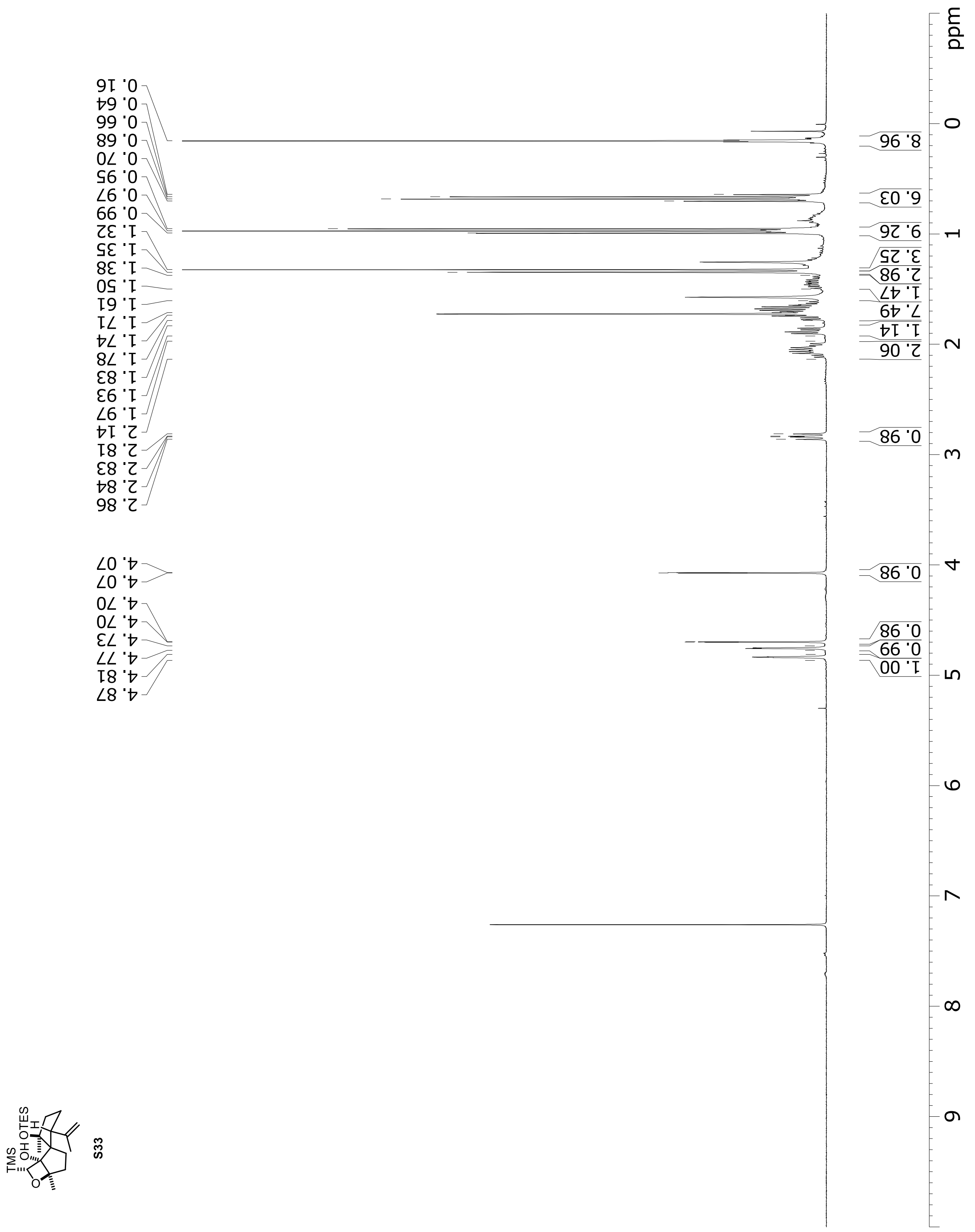

^{1}**H NMR spectrum** of oxetane **S33** measured in $CDCl_3$ at 400 MHz.

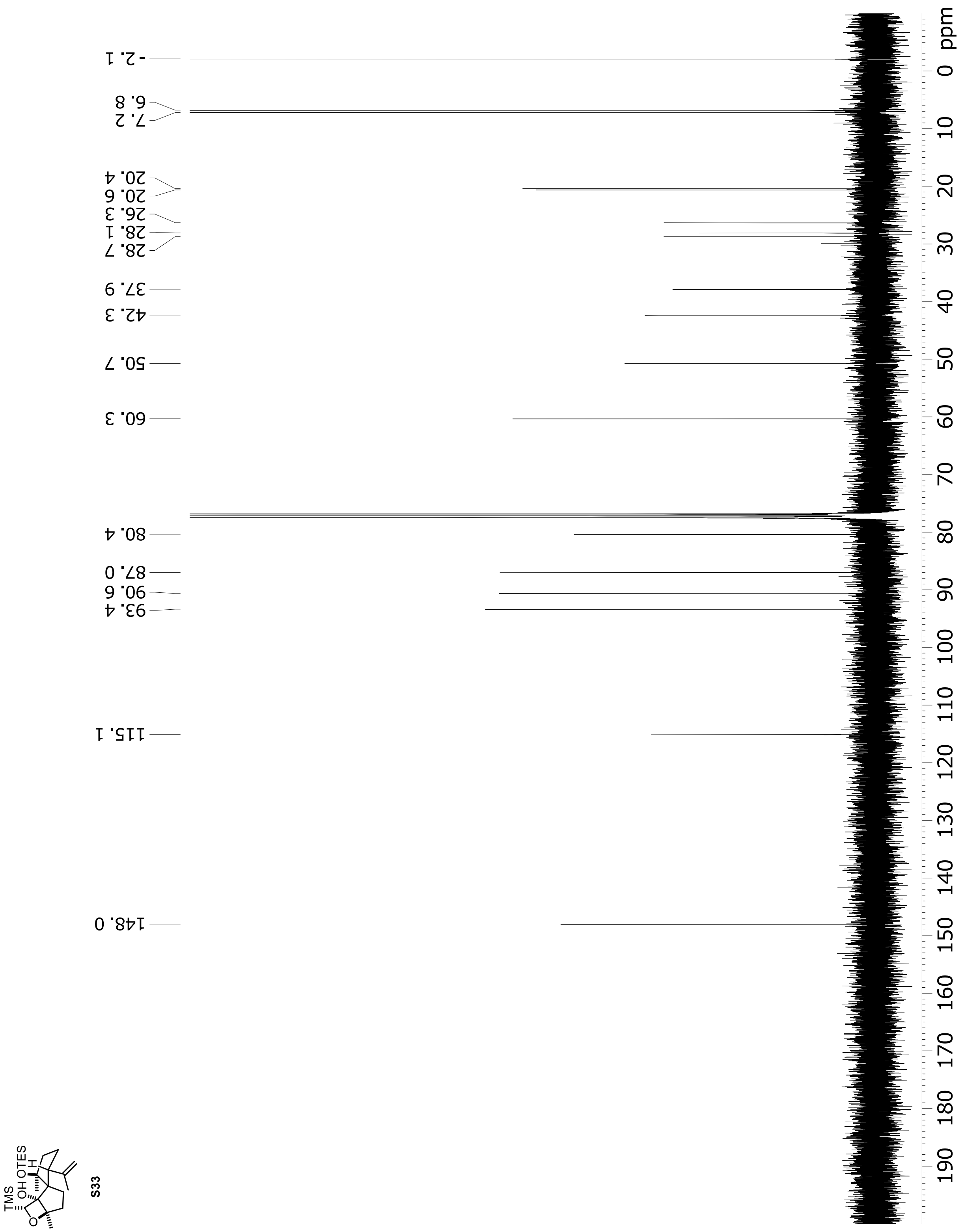

^{13}C NMR spectrum of oxetane **S33** measured in $CDCl_3$ at 101 MHz.

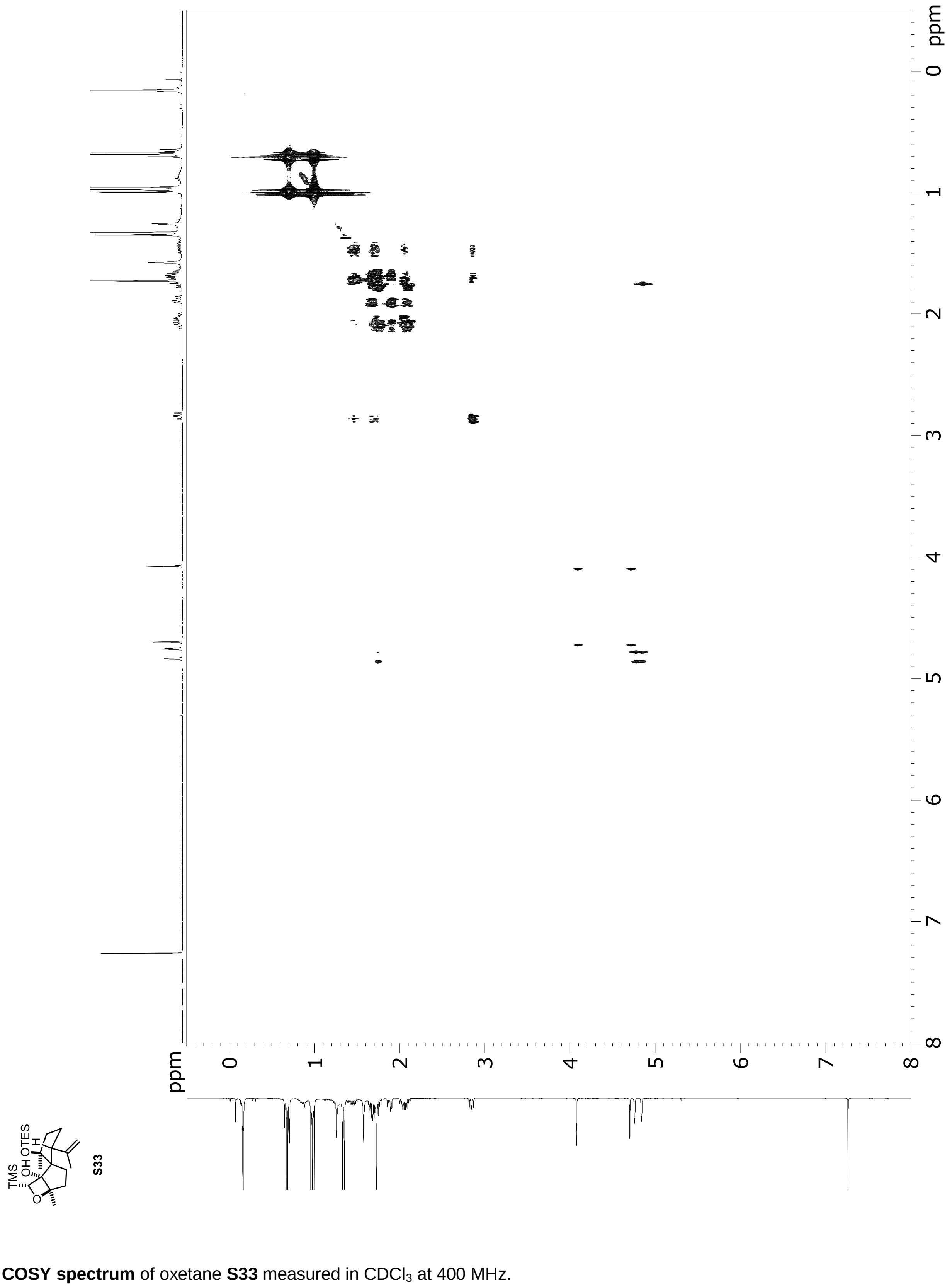

COSY spectrum of oxetane **S33** measured in $CDCl_3$ at 400 MHz.

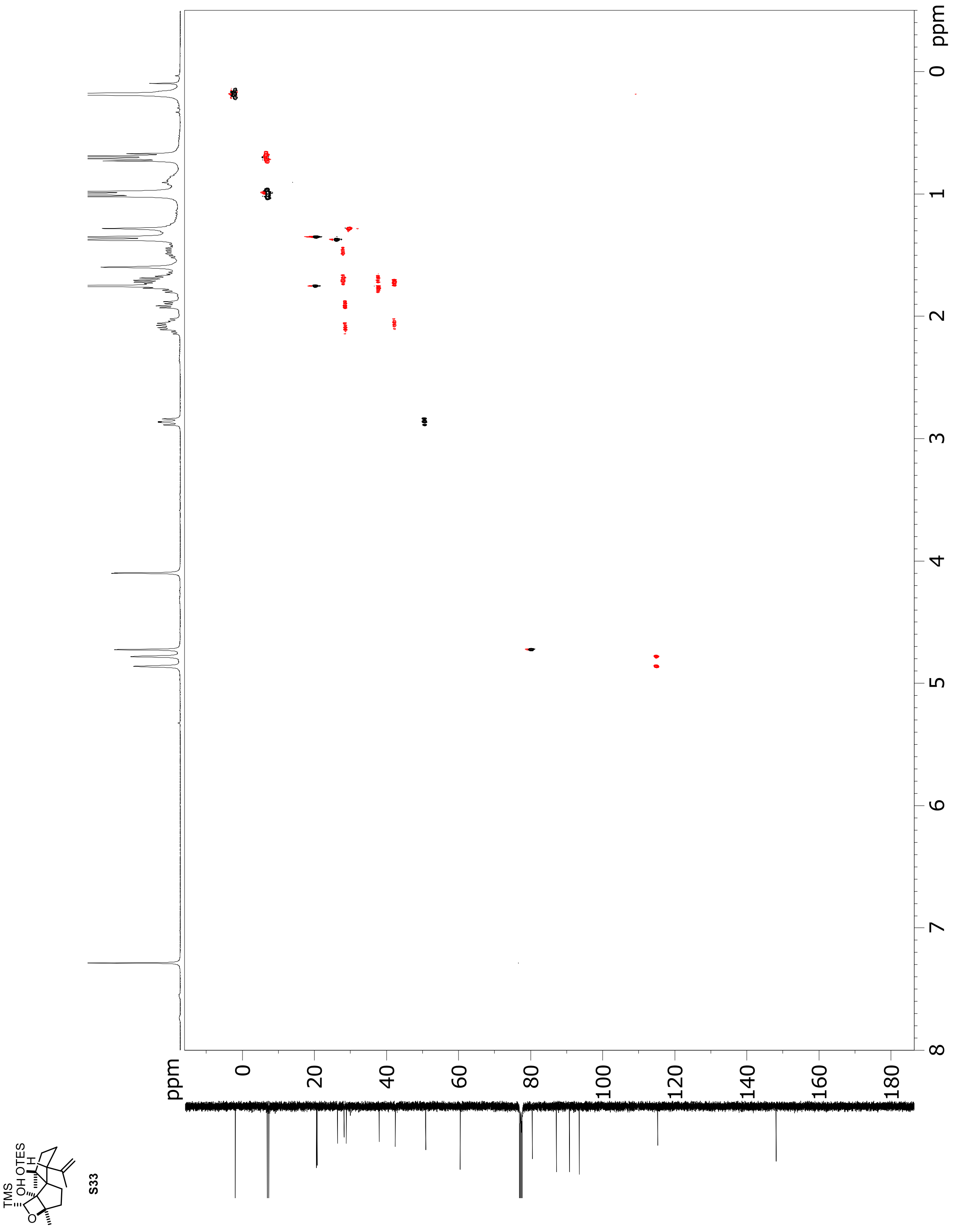

HSQC spectrum of oxetane **S33** measured in $CDCl_3$ at 400 MHz.

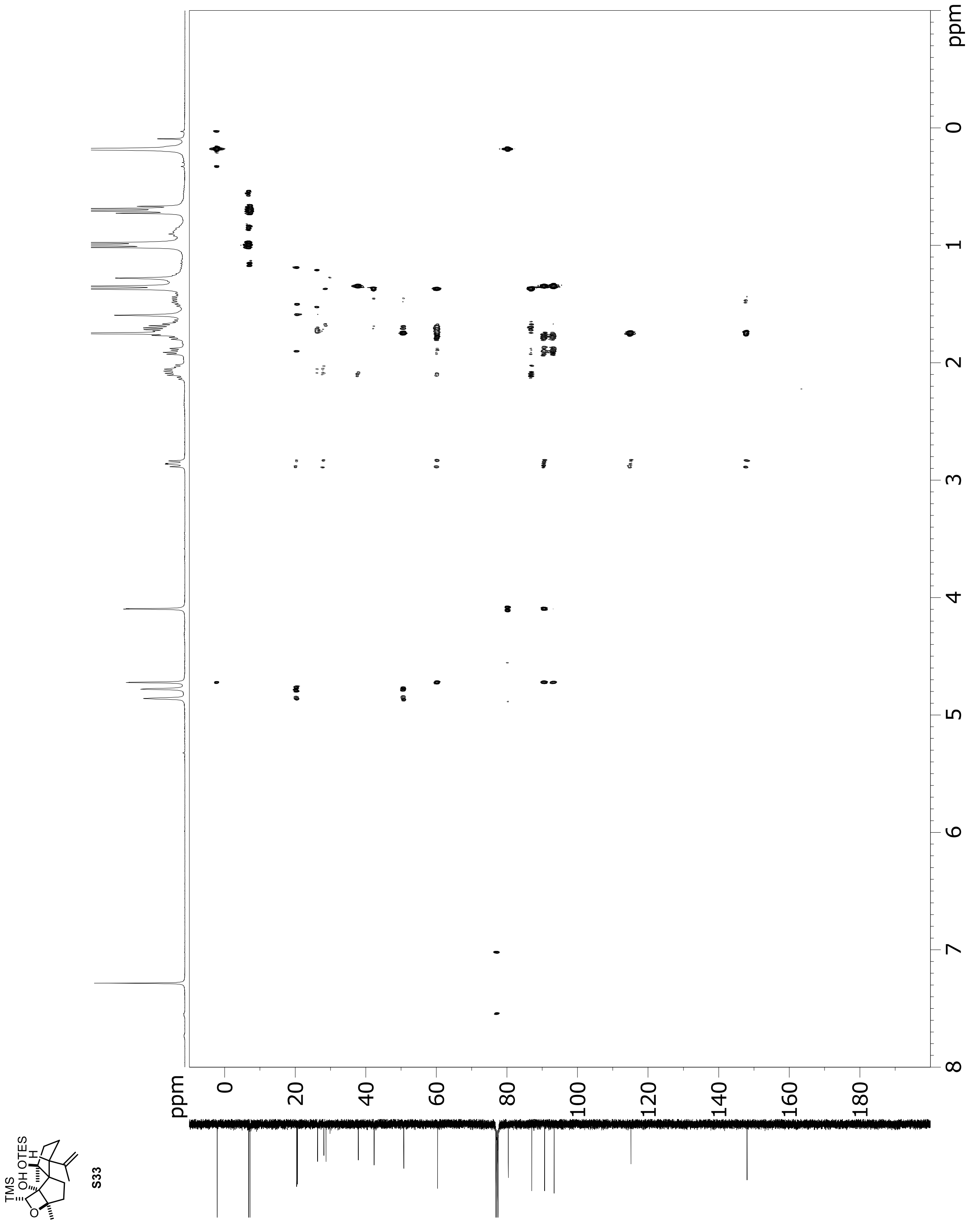

HMBC spectrum of oxetane **S33** measured in $CDCl_3$ at 400 MHz.

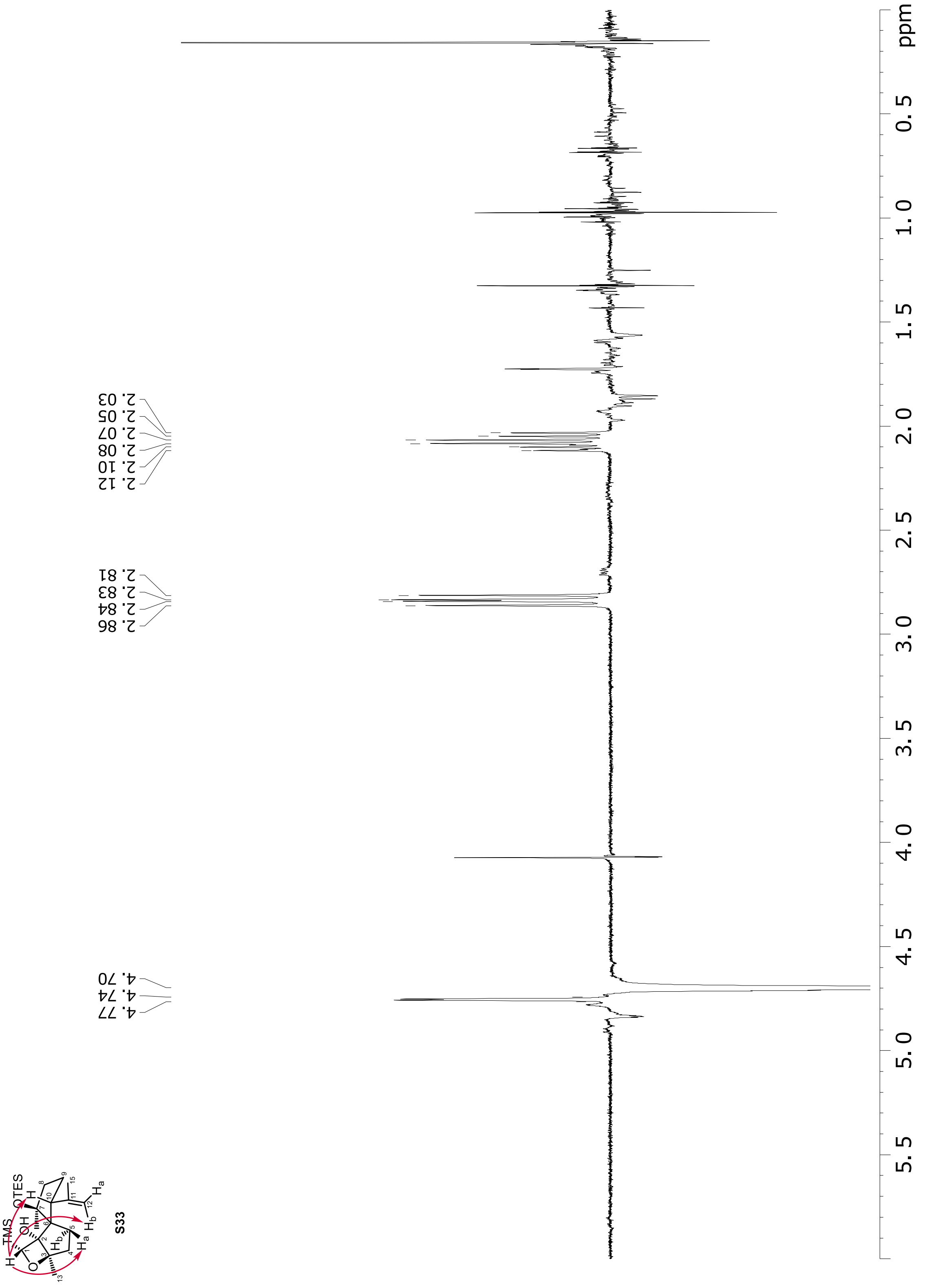

^{1}H-NOE spectrum of oxetane **S33** after irradiation at 4.70 ppm (CH-1), measured in $CDCl_3$ at 400 MHz.

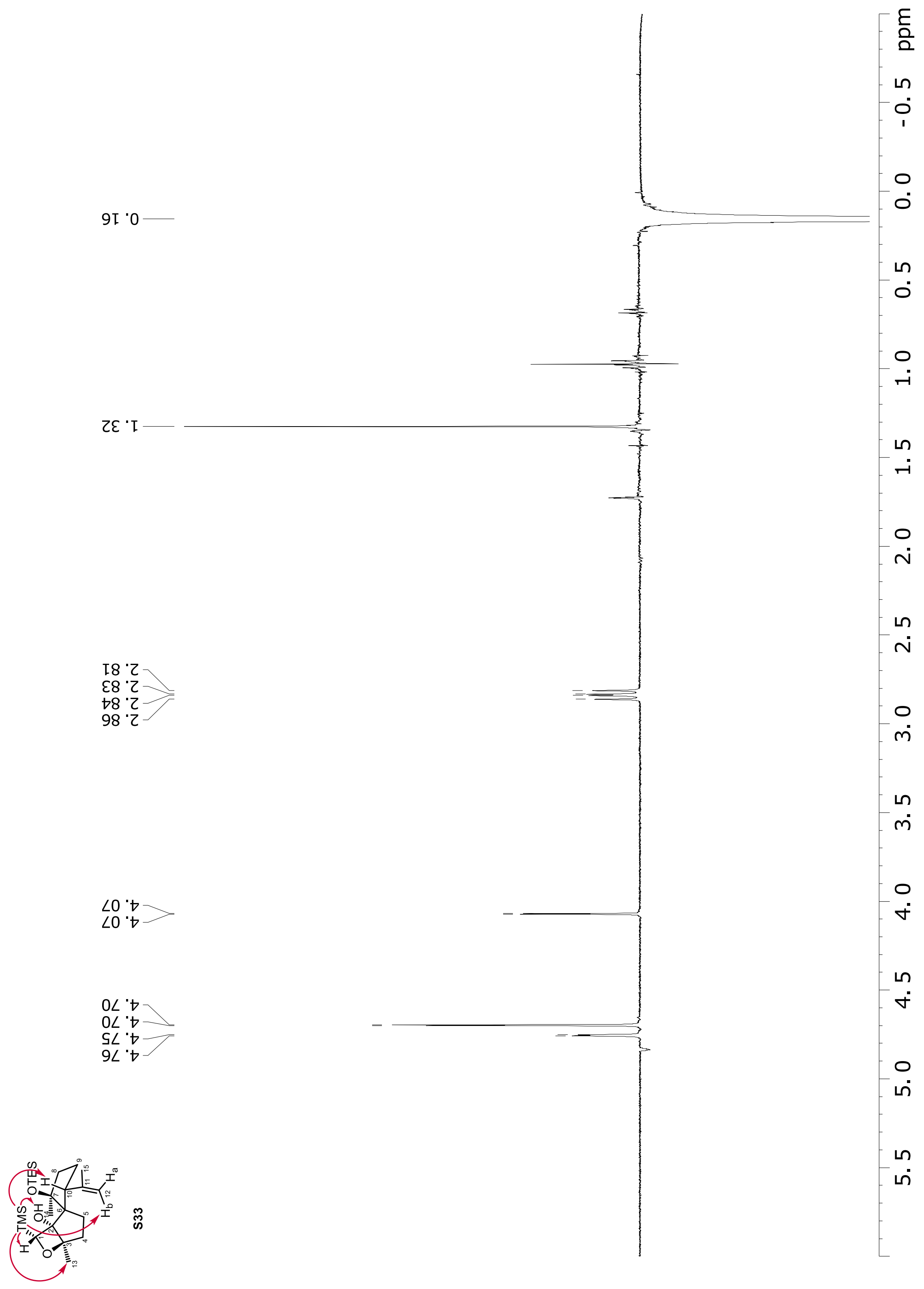

¹H-NOE spectrum of oxetane **S33** after irradiation at 0.16 ppm (TMS), measured in $CDCl_3$ at 400 MHz.

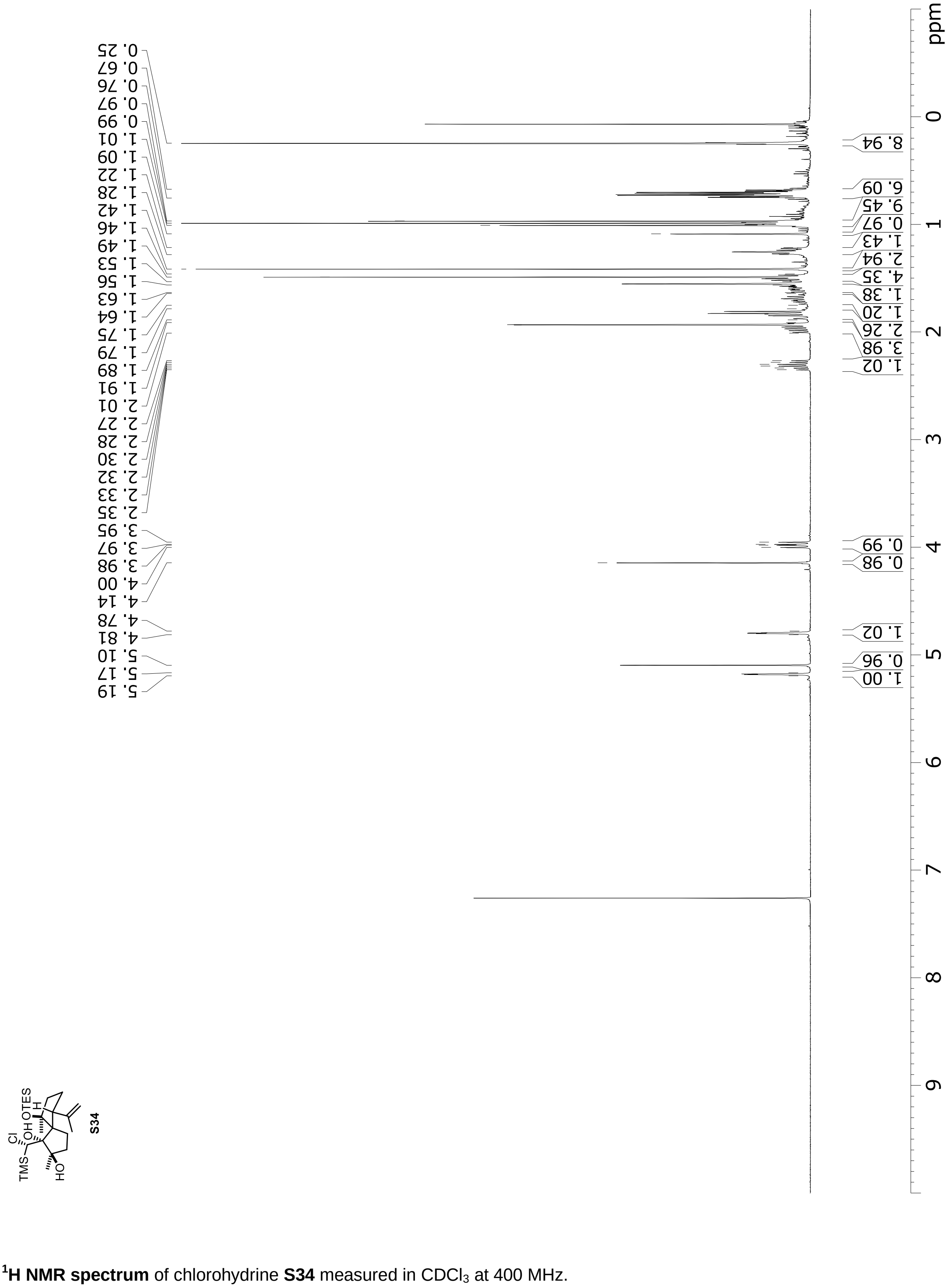

^{1}H NMR spectrum of chlorohydrine **S34** measured in $CDCl_3$ at 400 MHz.

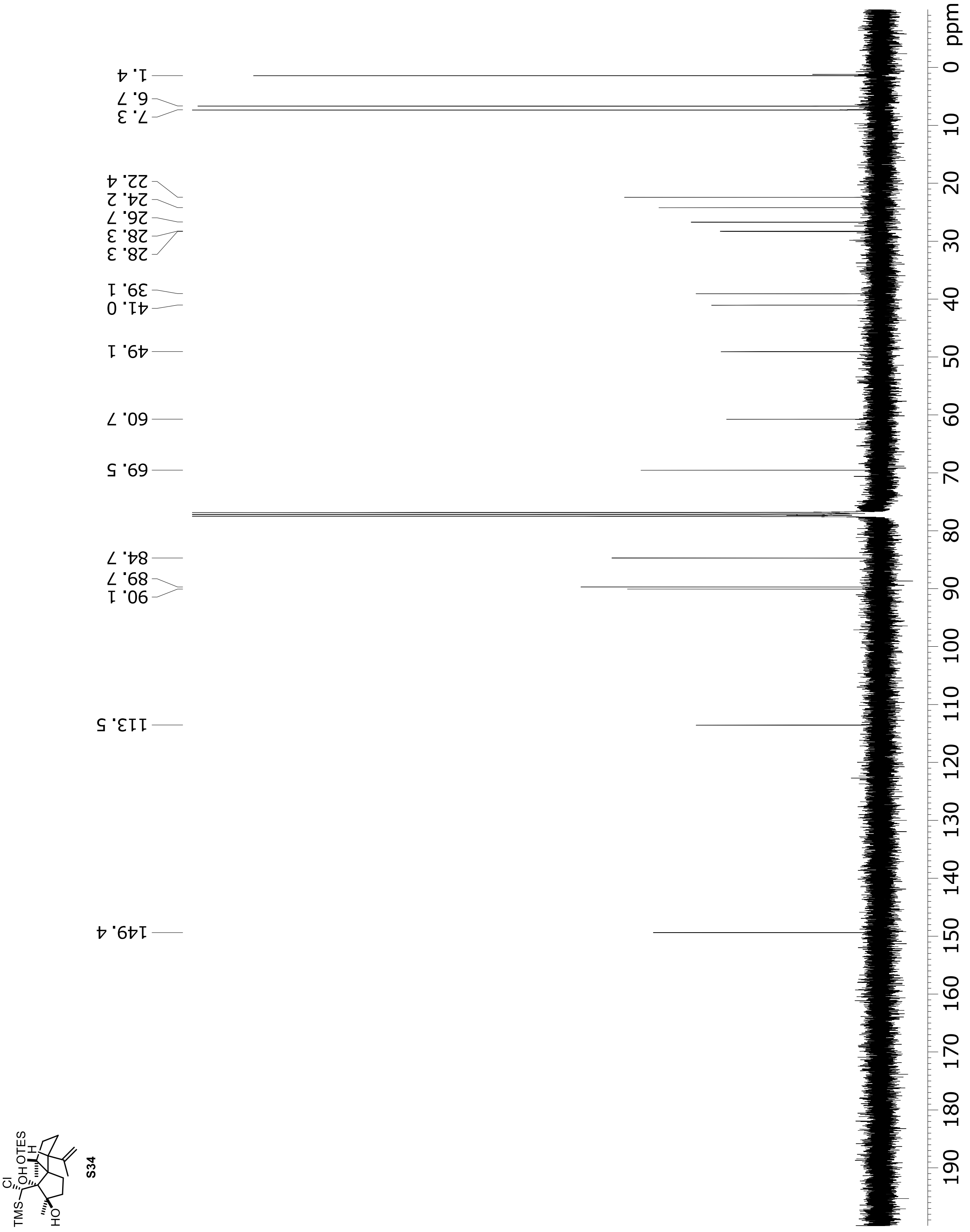

^{13}C NMR spectrum of chlorohydrine **S34** measured in $CDCl_3$ at 101 MHz.

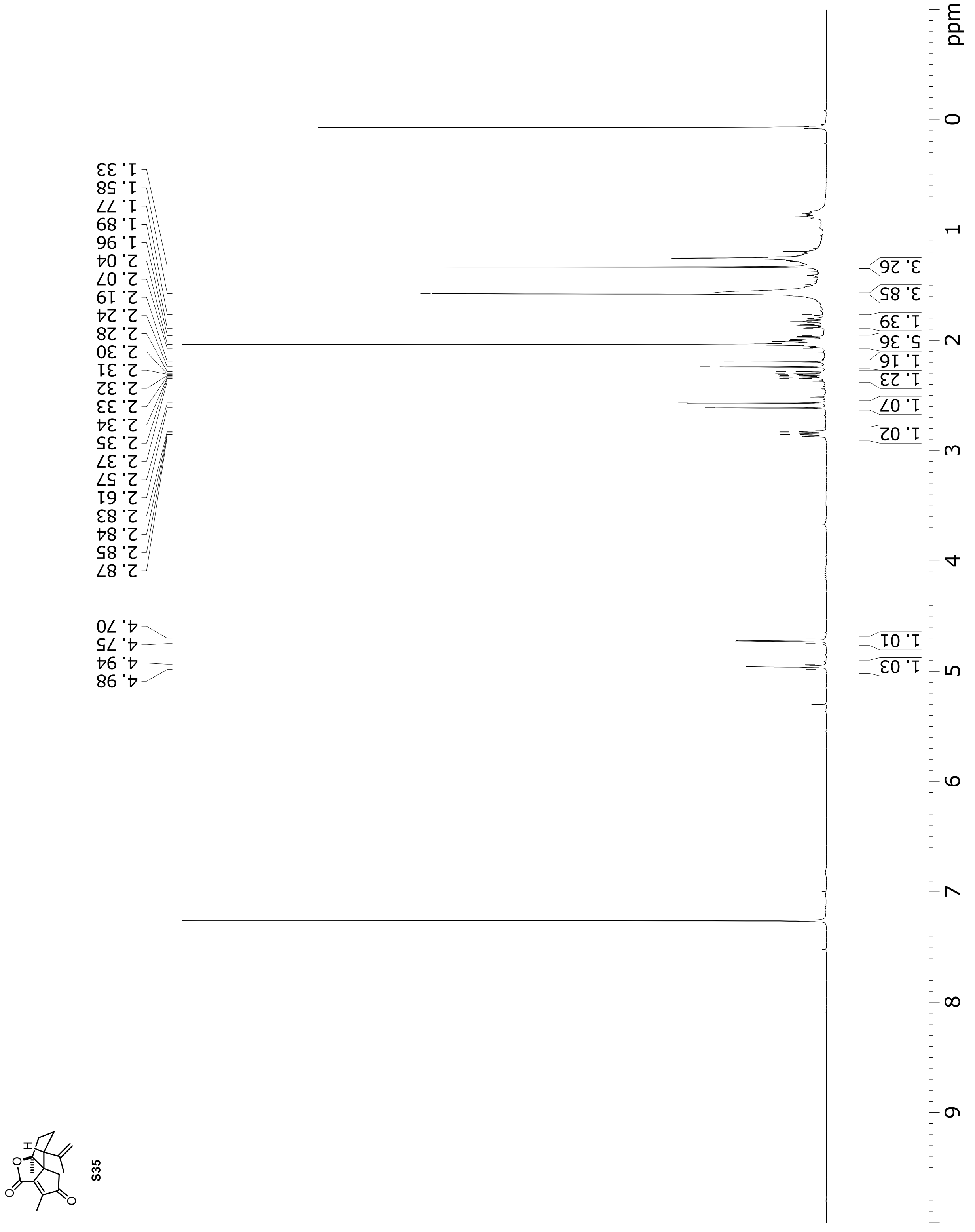

^{1}H NMR spectrum of lactone **S35** measured in $CDCl_3$ at 400 MHz.

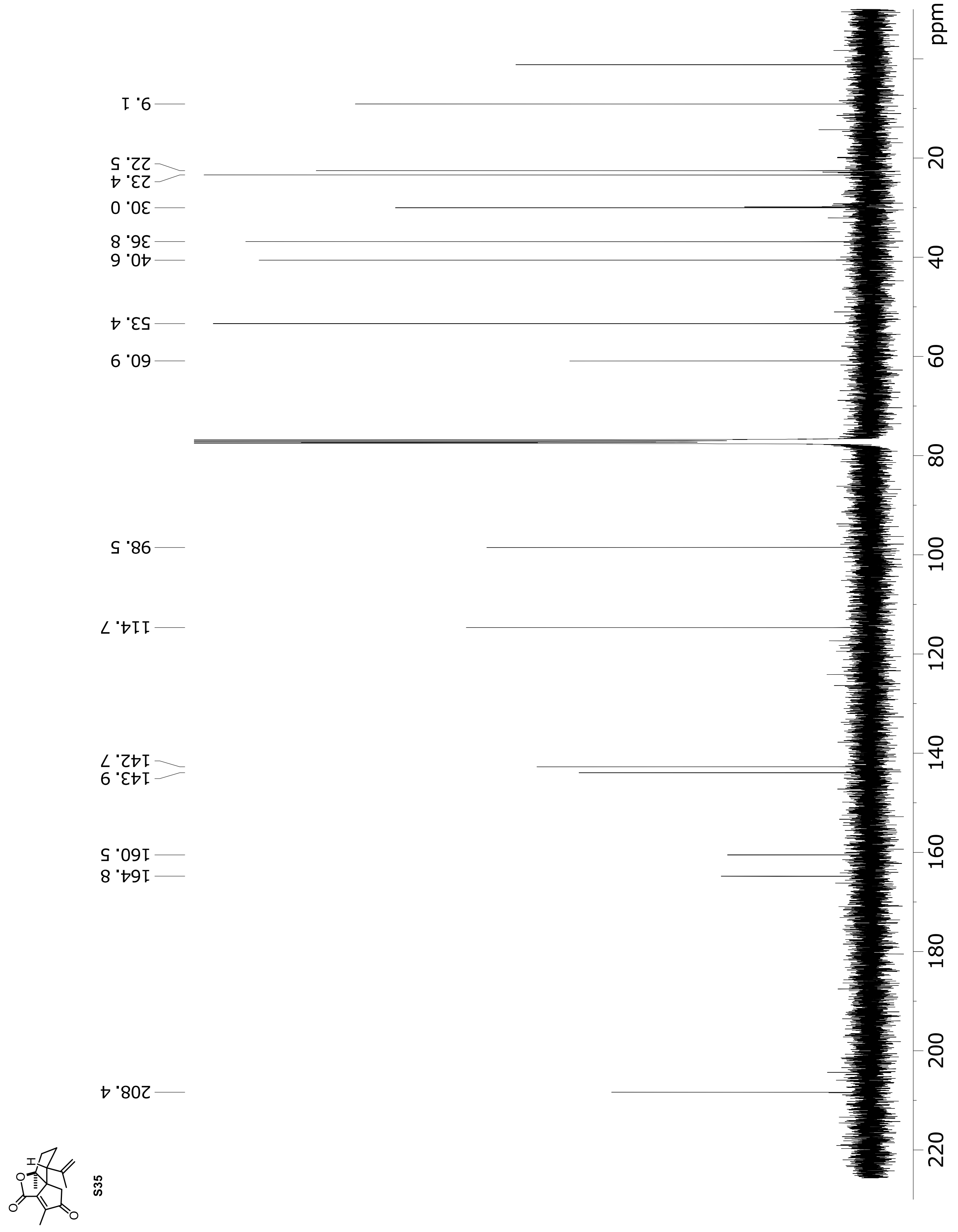

^{13}C NMR spectrum of lactone **S35** measured in $CDCl_3$ at 101 MHz.

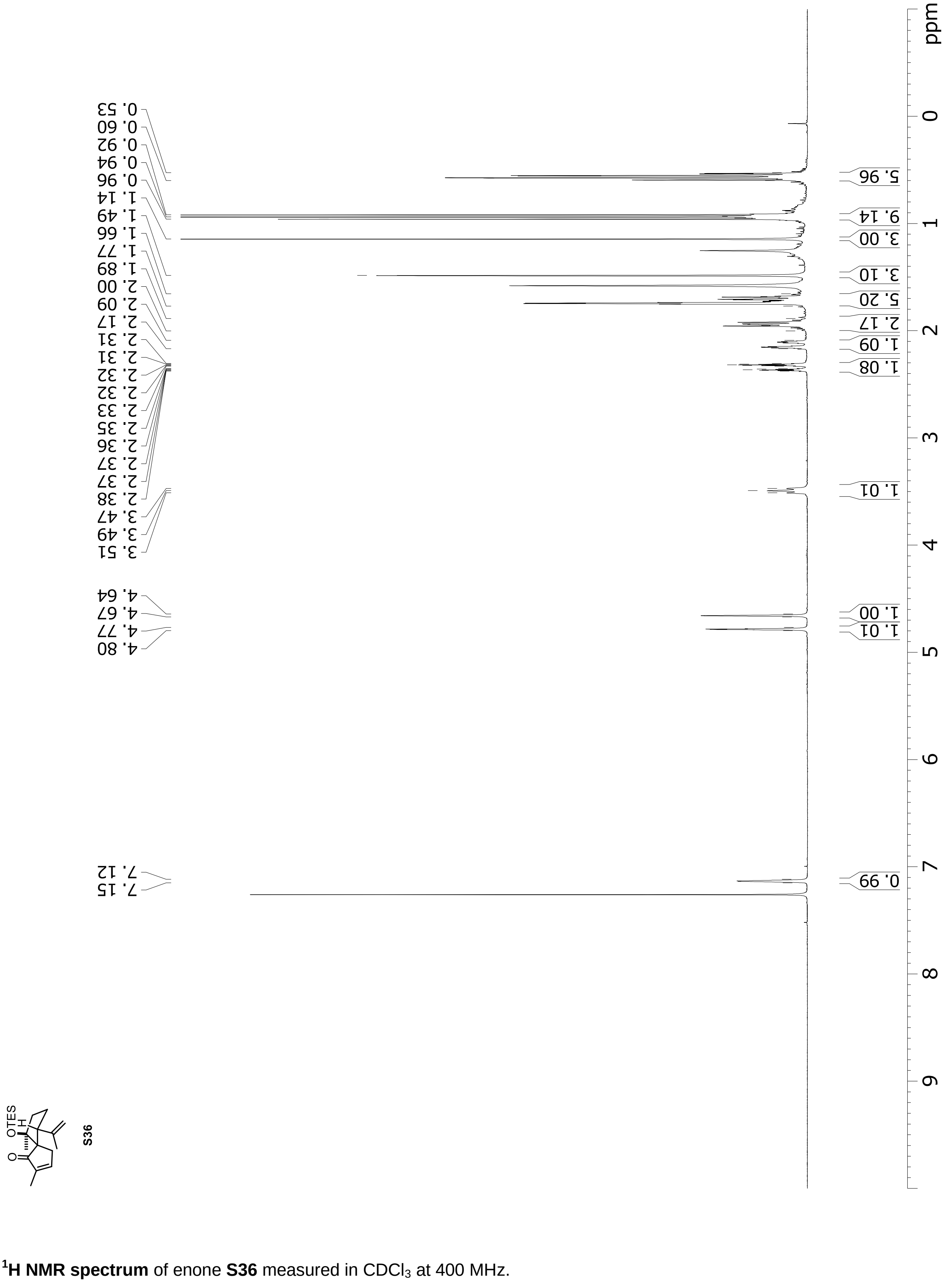

^{1}H NMR spectrum of enone **S36** measured in $CDCl_3$ at 400 MHz.

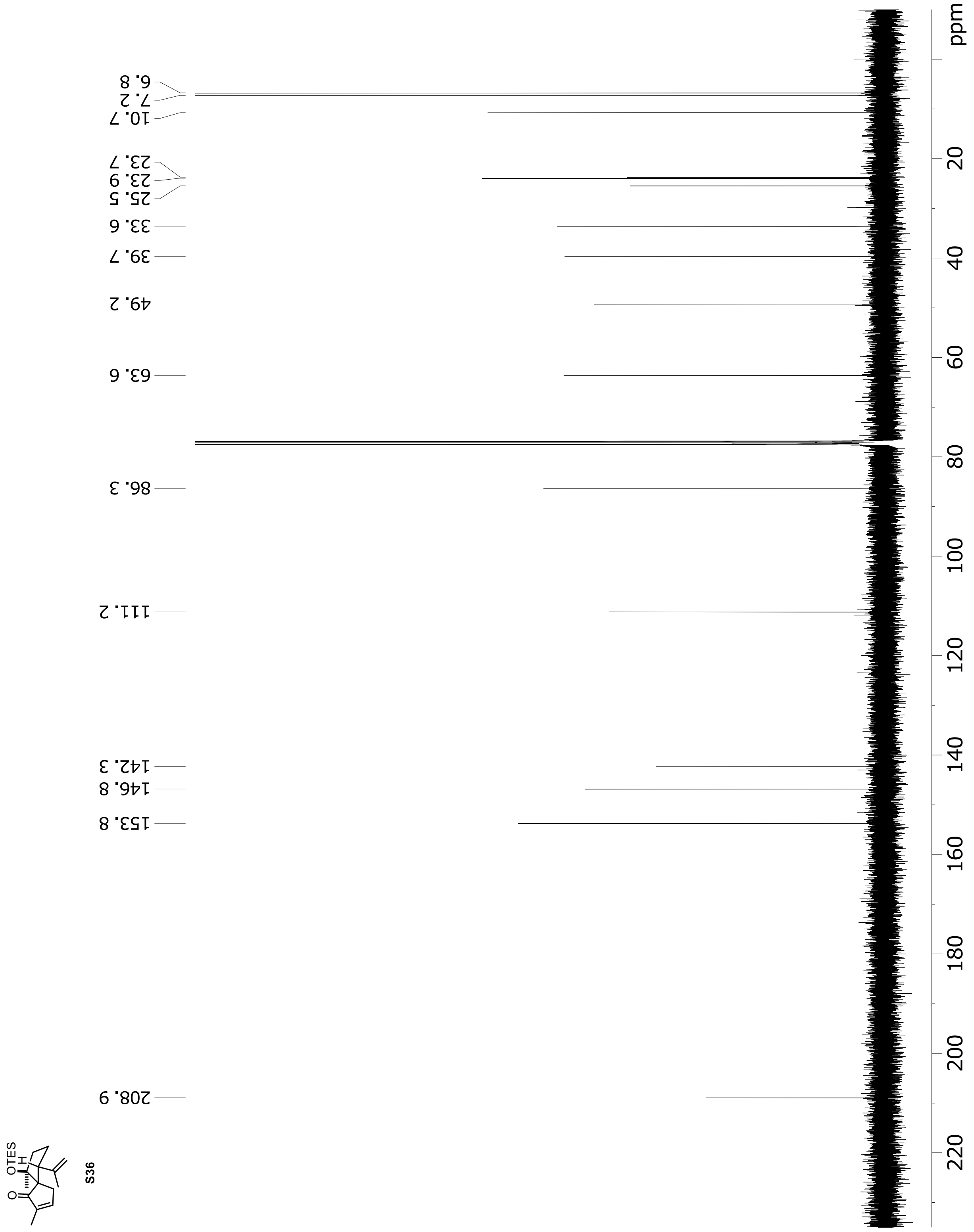

^{1}H NMR spectrum of enone **S36** measured in $CDCl_3$ at 400 MHz.

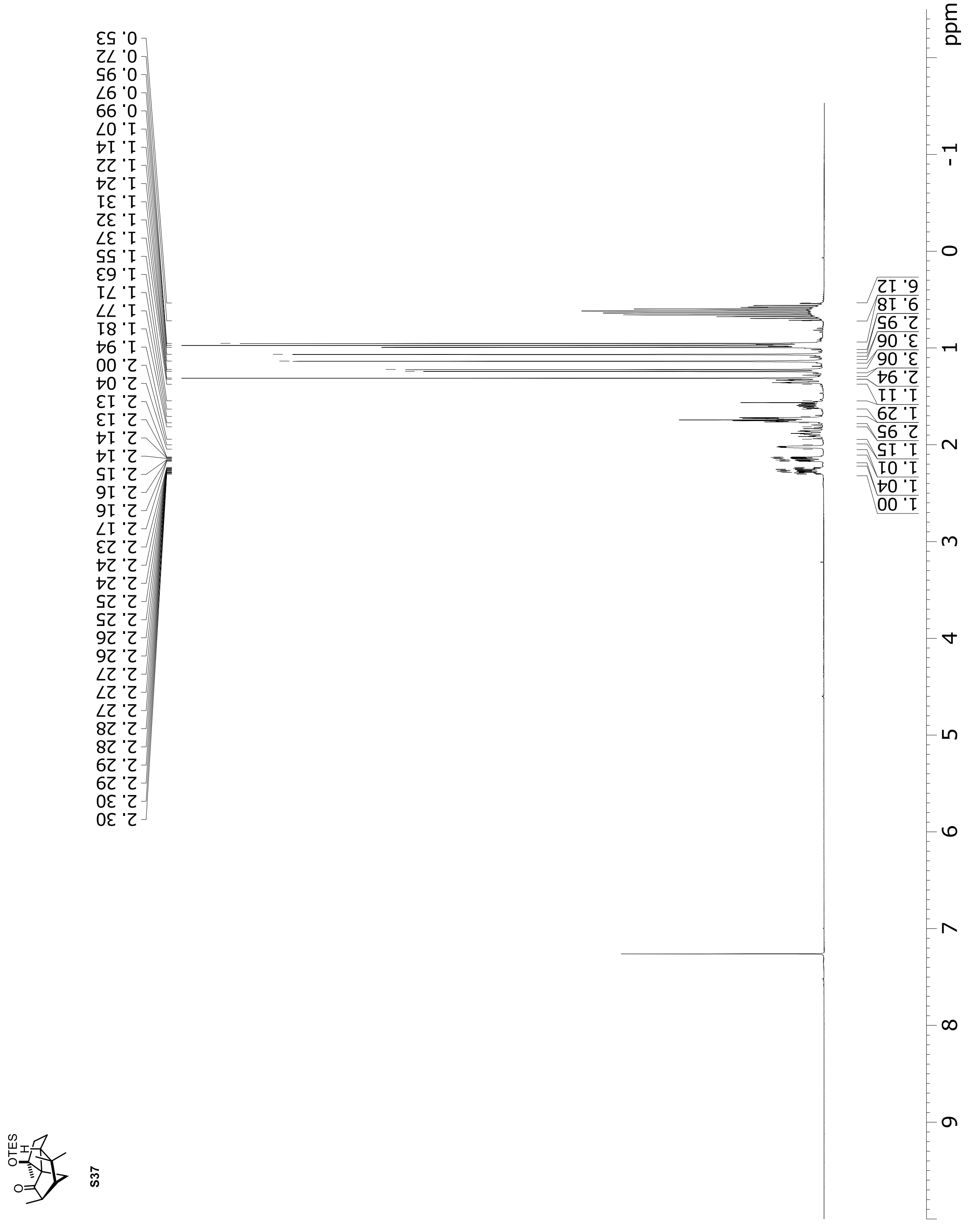

¹H NMR spectrum of tricycle **S37** measured in $CDCl_3$ at 400 MHz.

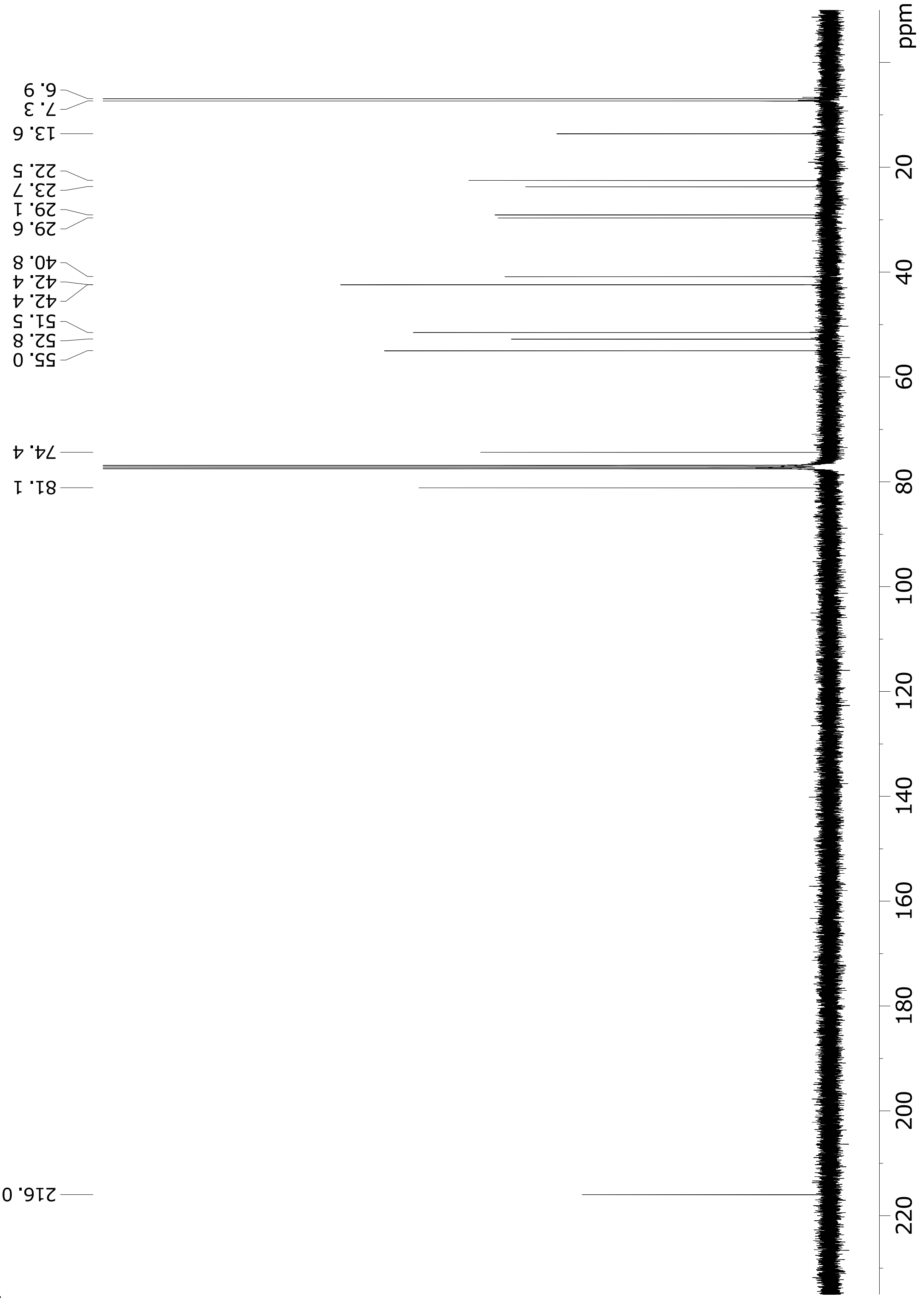

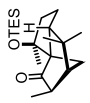

S37

^{13}C NMR spectrum of tricycle **S37** measured in $CDCl_3$ at 101 MHz.

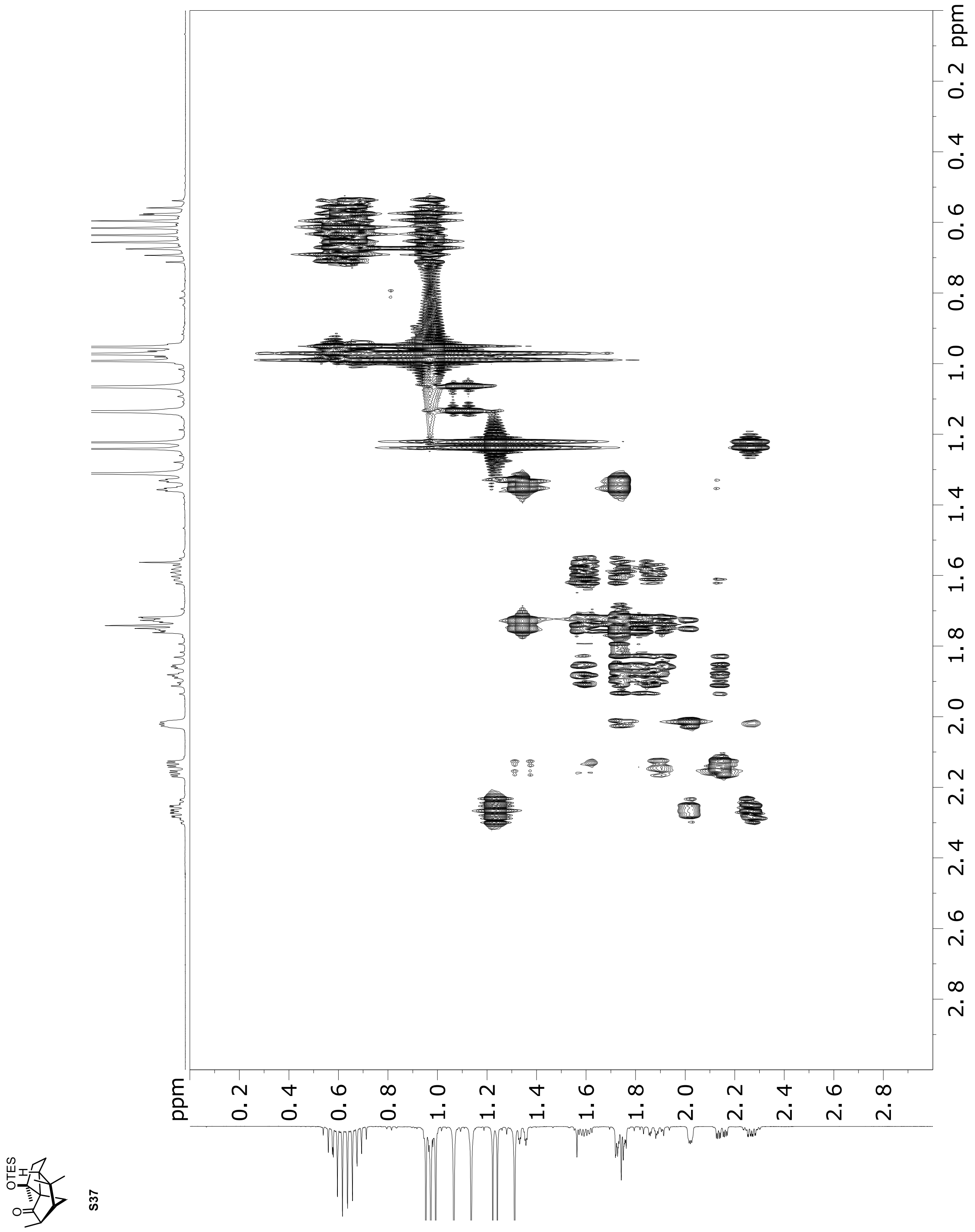

COSY NMR spectrum of tricycle **S37** measured in $CDCl_3$ at 400 MHz.

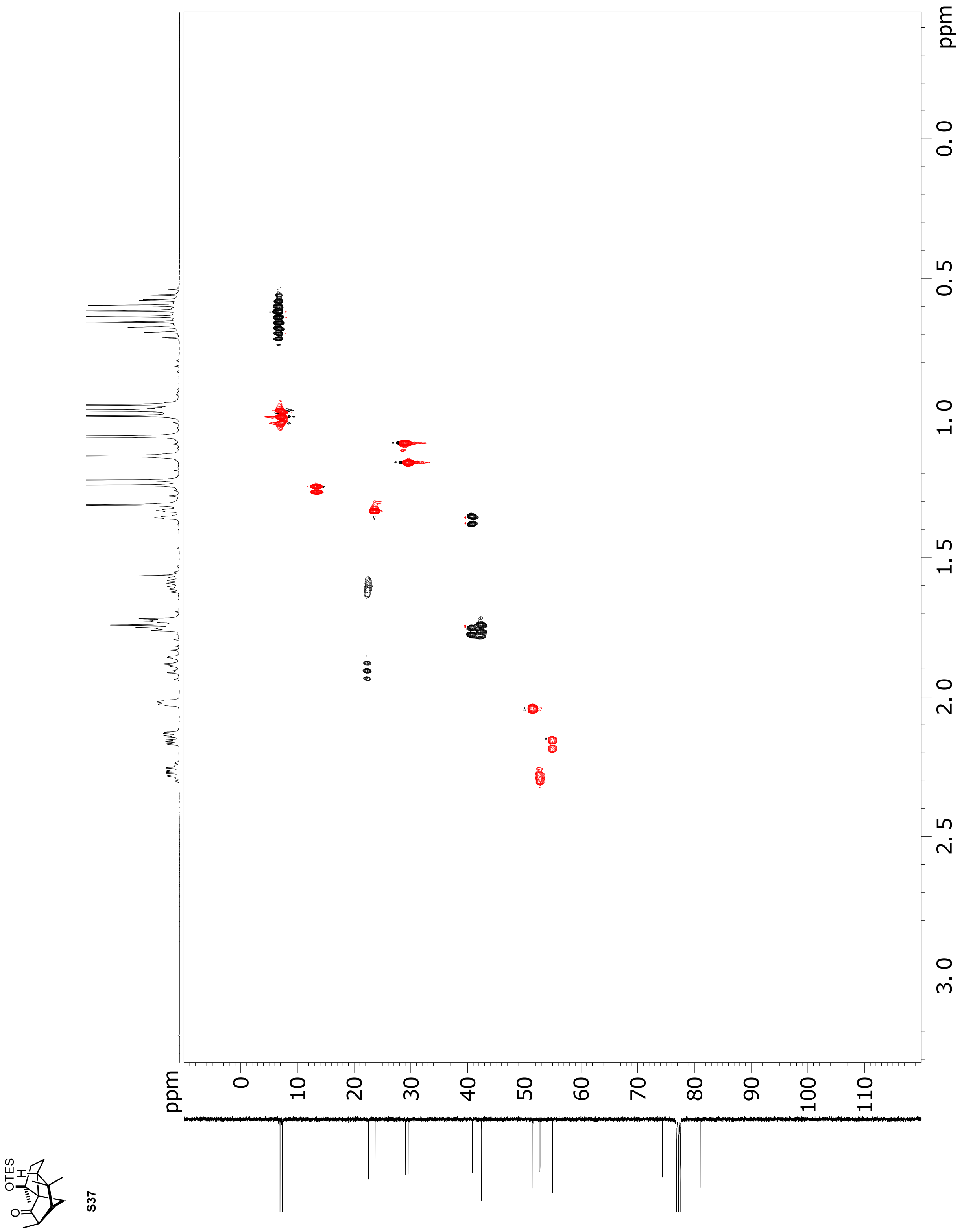

HSQC NMR spectrum of tricycle **S37** measured in $CDCl_3$ at 400 MHz.

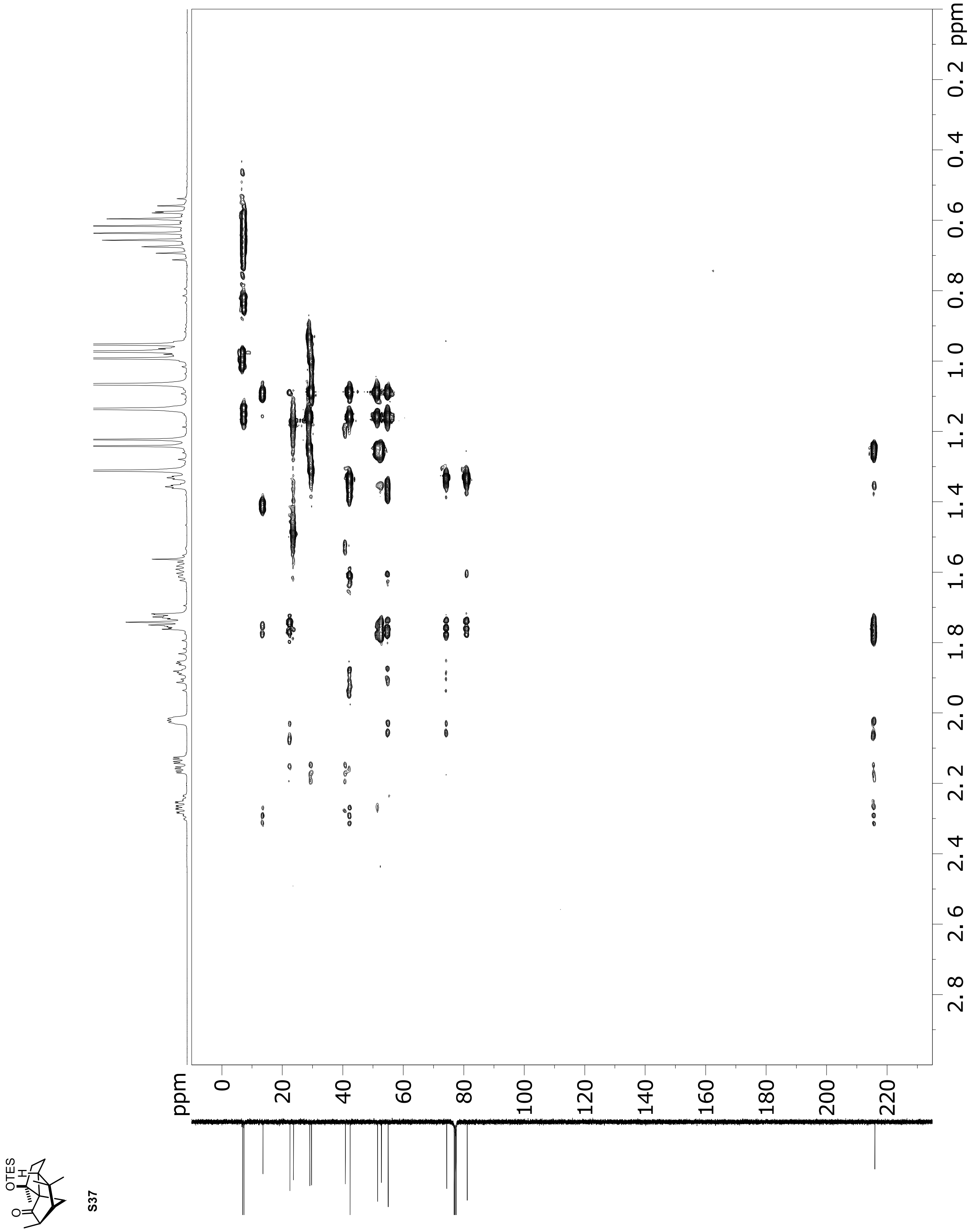

HMBC NMR spectrum of tricycle **S37** measured in $CDCl_3$ at 400 MHz.

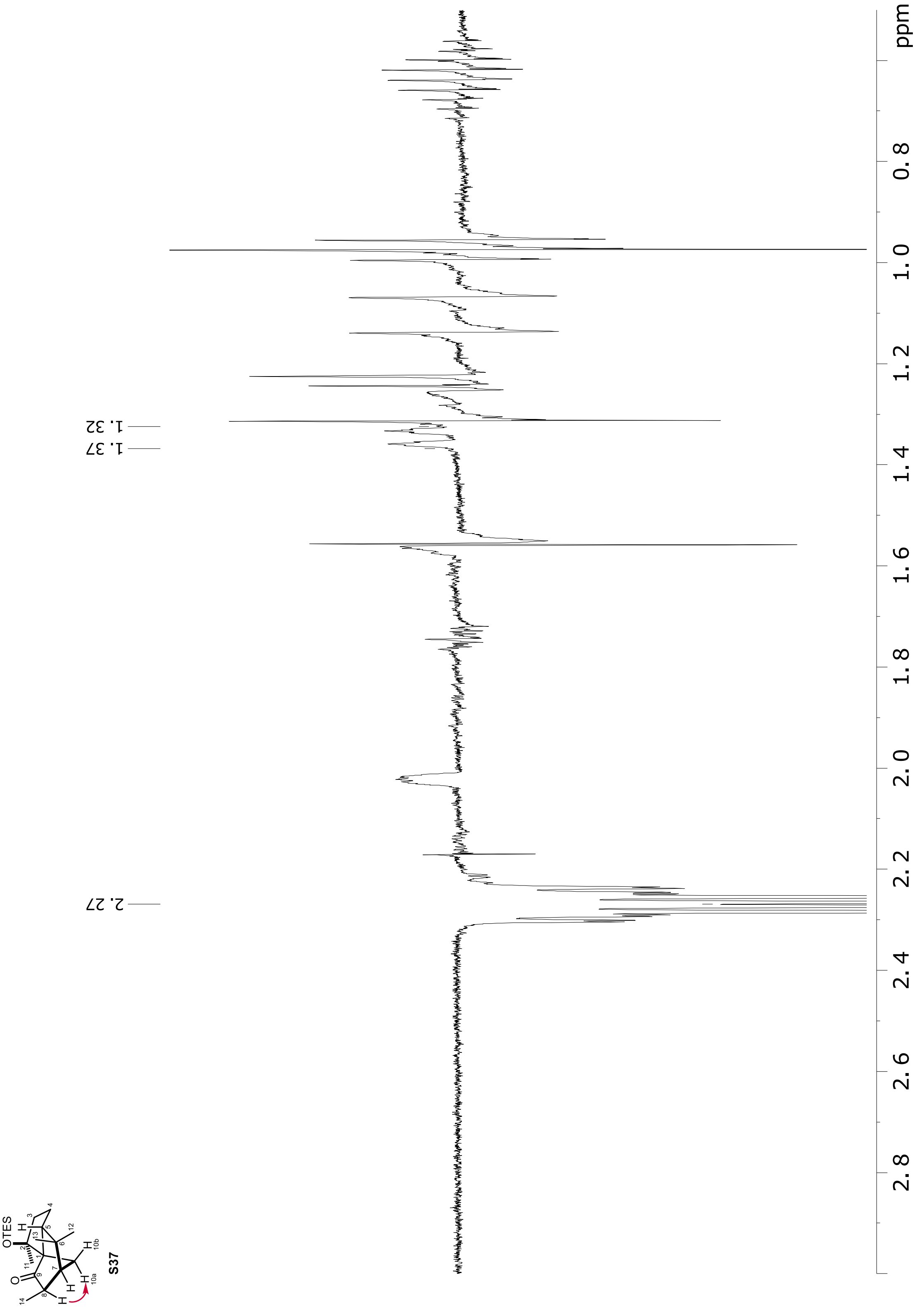

^{1}H-NOE spectrum of tricycle **S37** after irradiation at 2.27 ppm (CH-8), measured in $CDCl_3$ at 400 MHz.

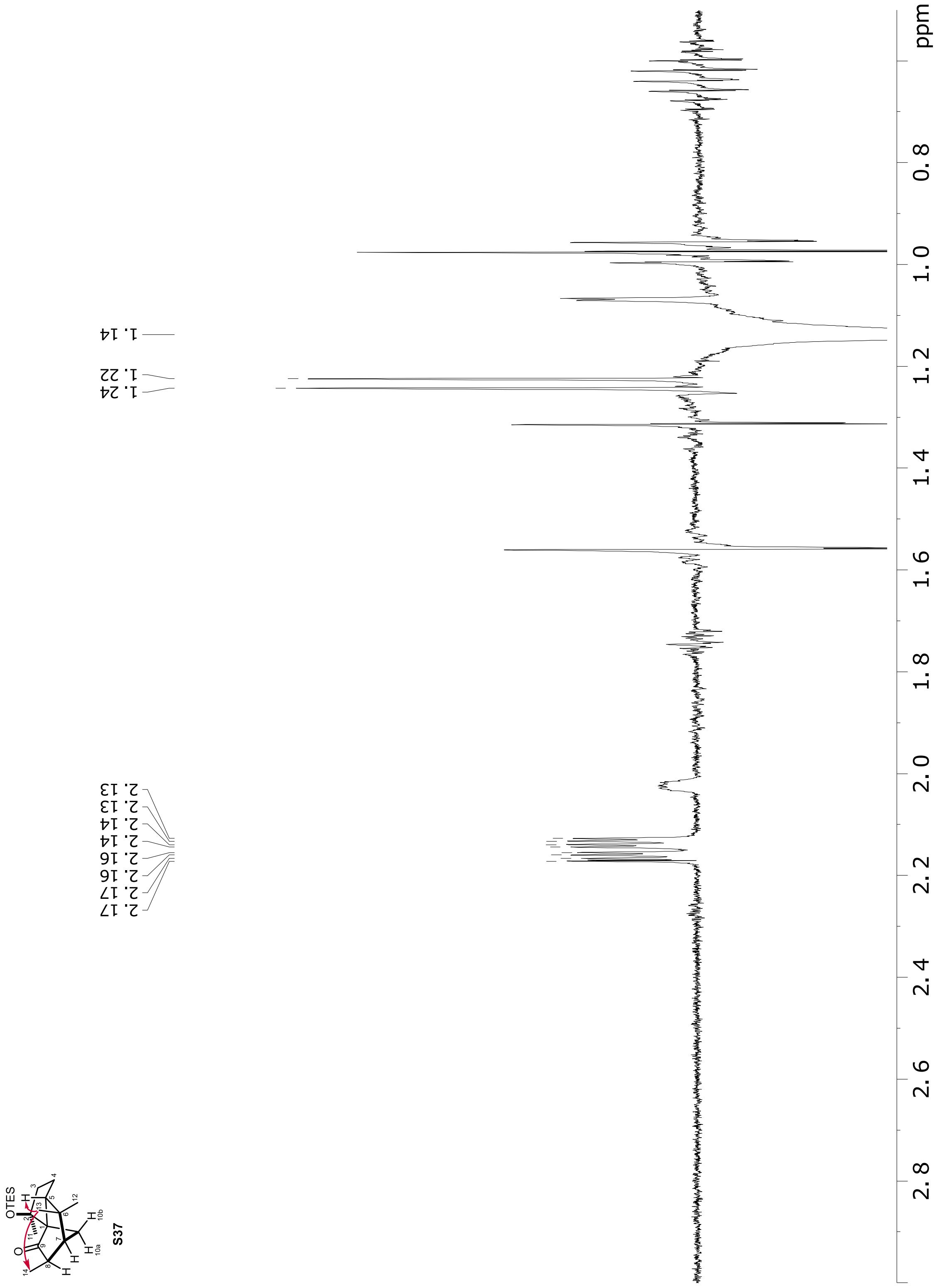

^{1}H-NOE spectrum of tricycle **S37** after irradiation at 1.14 ppm (CH_3-13), measured in $CDCl_3$ at 400 MHz.

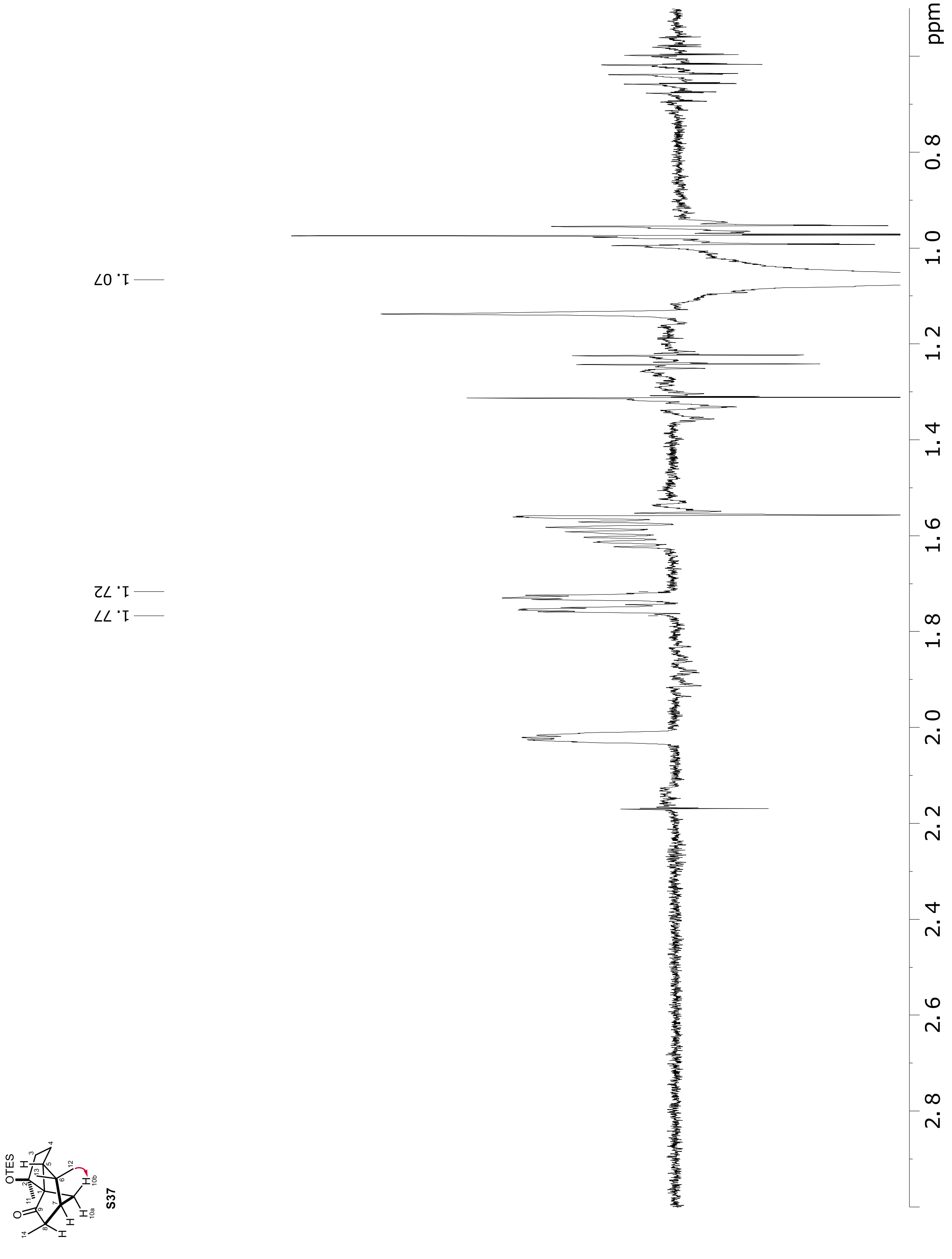

[1]**H-NOE spectrum** of tricycle **S37** after irradiation at 1.07 ppm (CH_3-12), measured in $CDCl_3$ at 400 MHz.

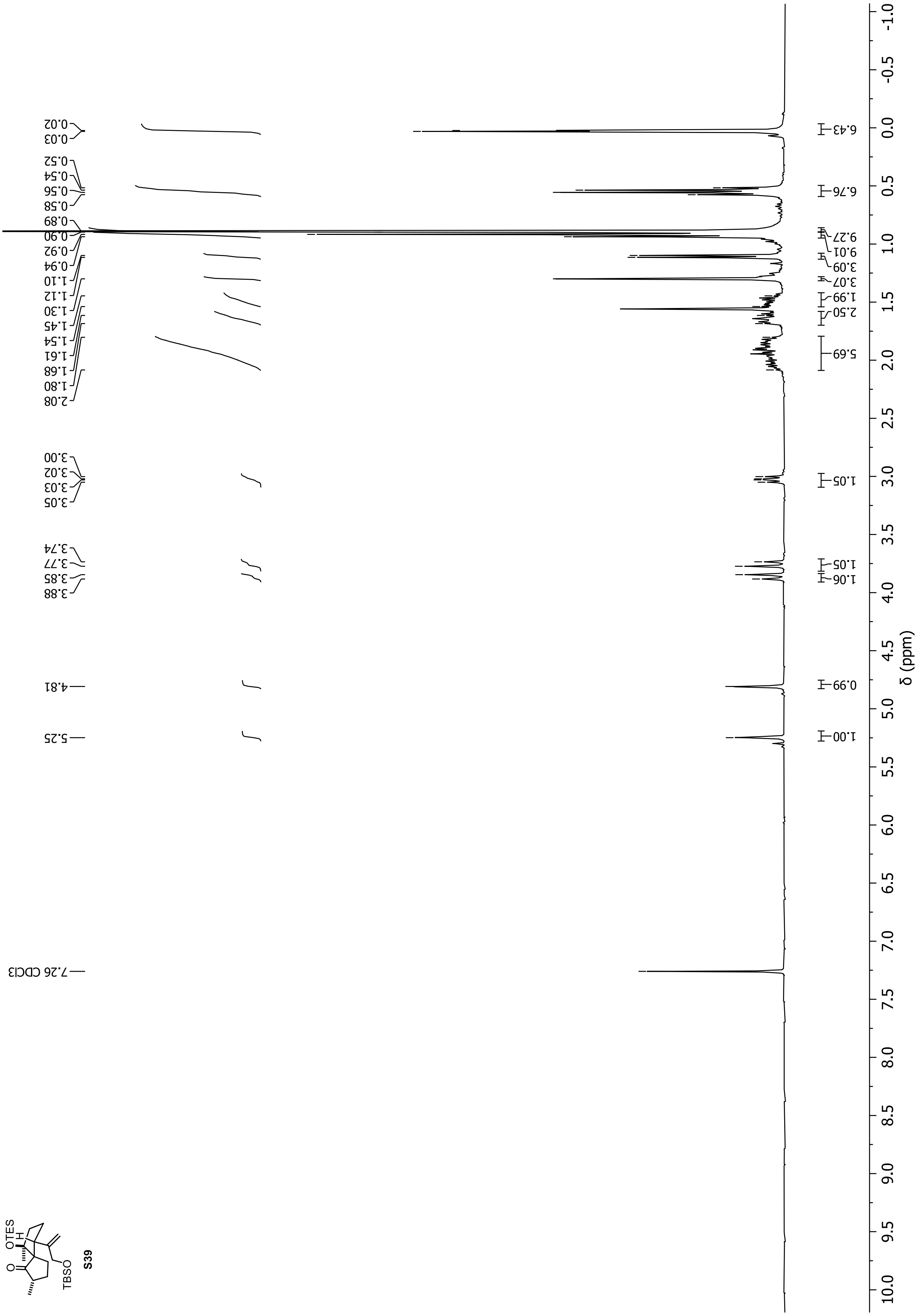

^{1}H NMR spectrum of spiroketone **S39** (OTBS derivative) measured in $CDCl_3$ at 400 MHz.

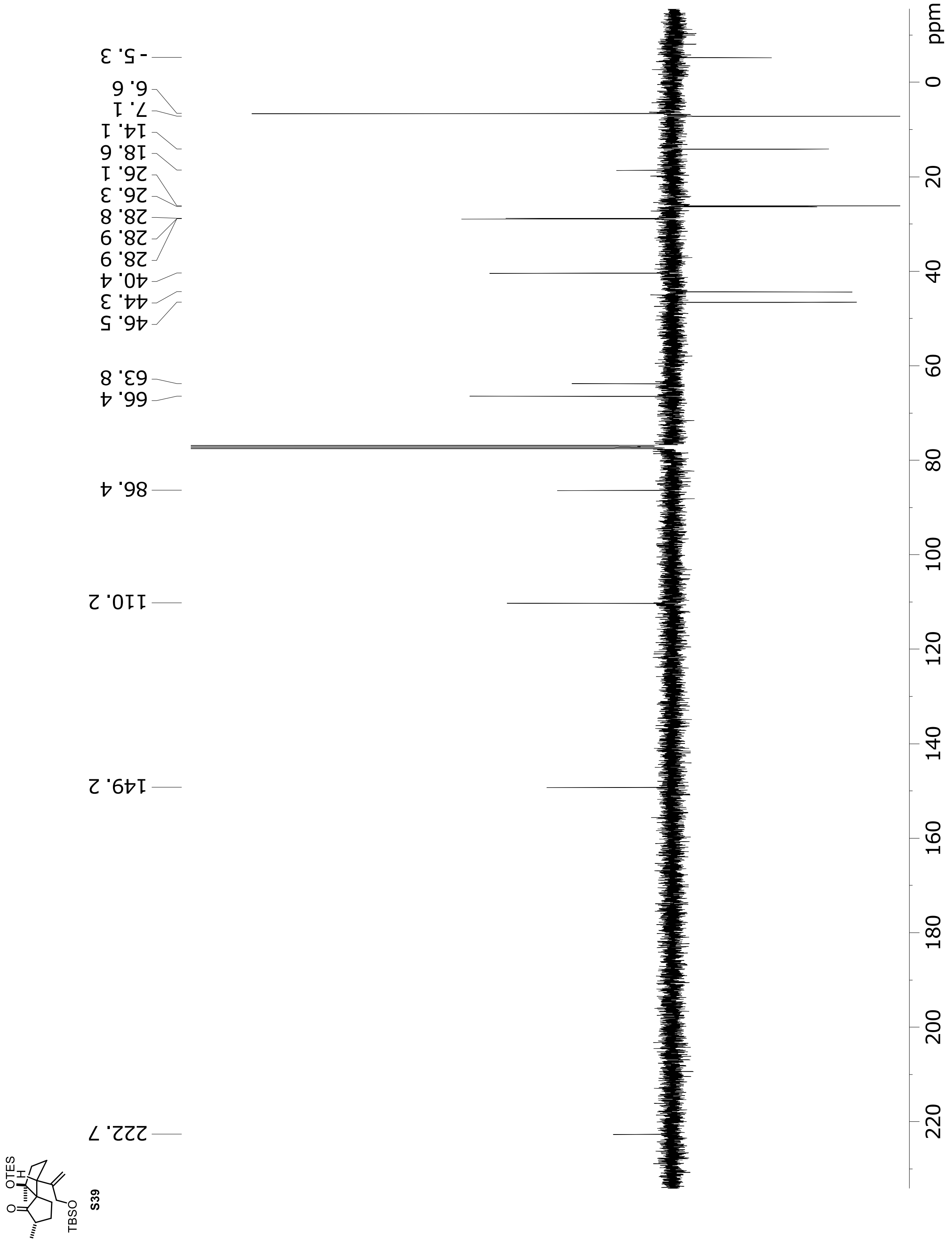

DEPT NMR spectrum of spiroketone **S39** (OTBS derivative) measured in $CDCl_3$ at 101 MHz.

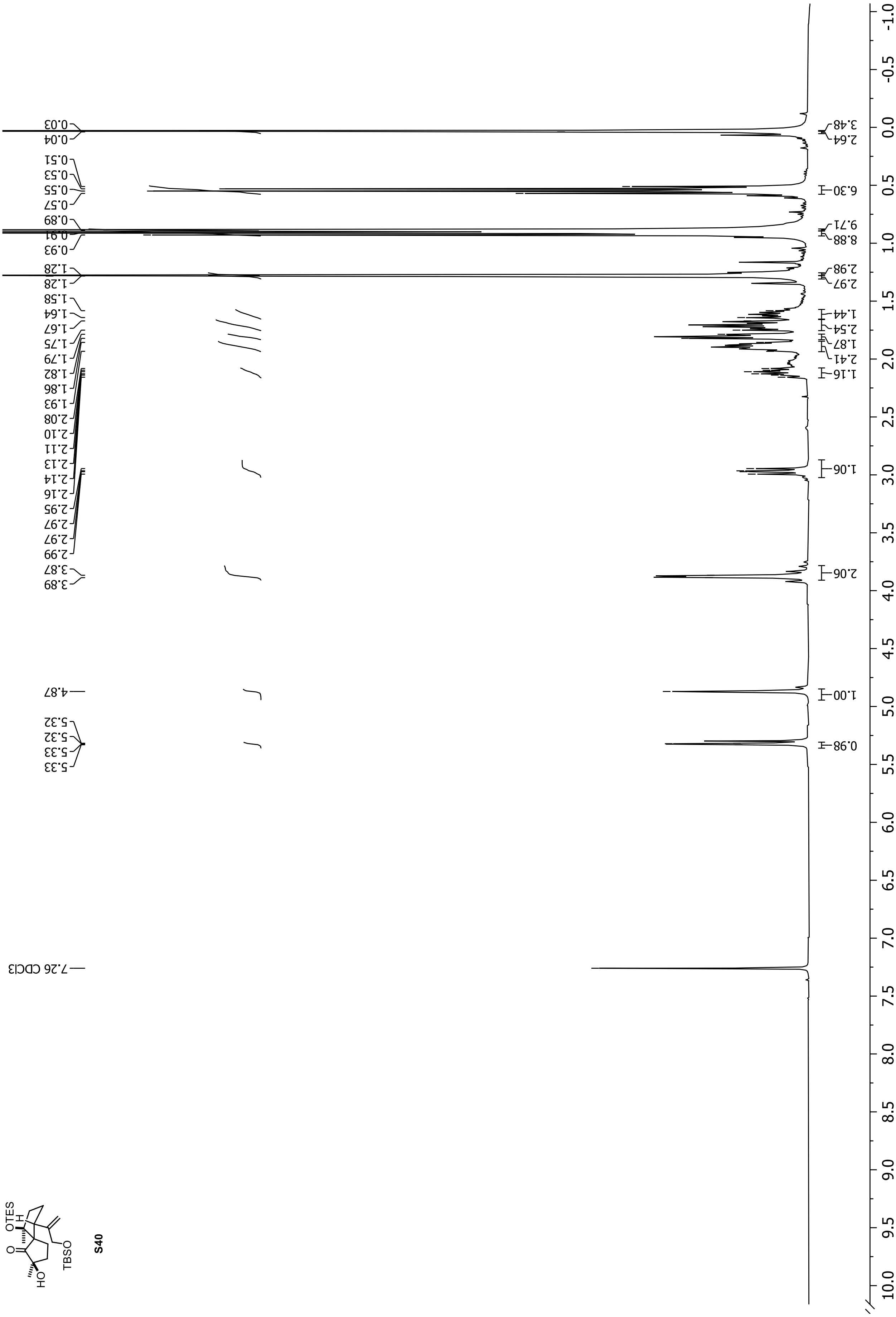

¹H NMR spectrum of hydroxyketone **S40** measured in $CDCl_3$ at 400 MHz.

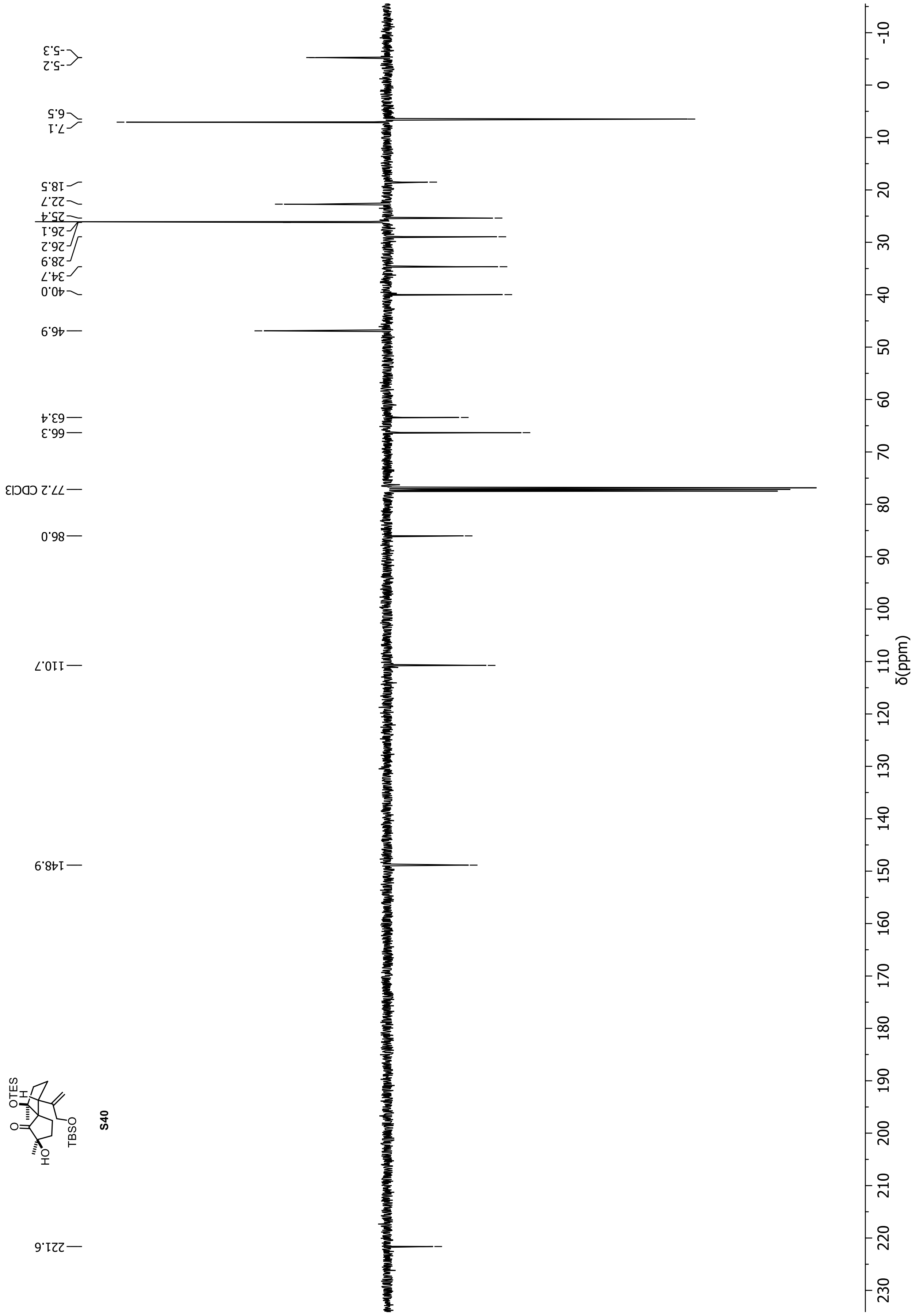

DEPT spectrum of hydroxyketone **S40** measured in $CDCl_3$ at 101 MHz.

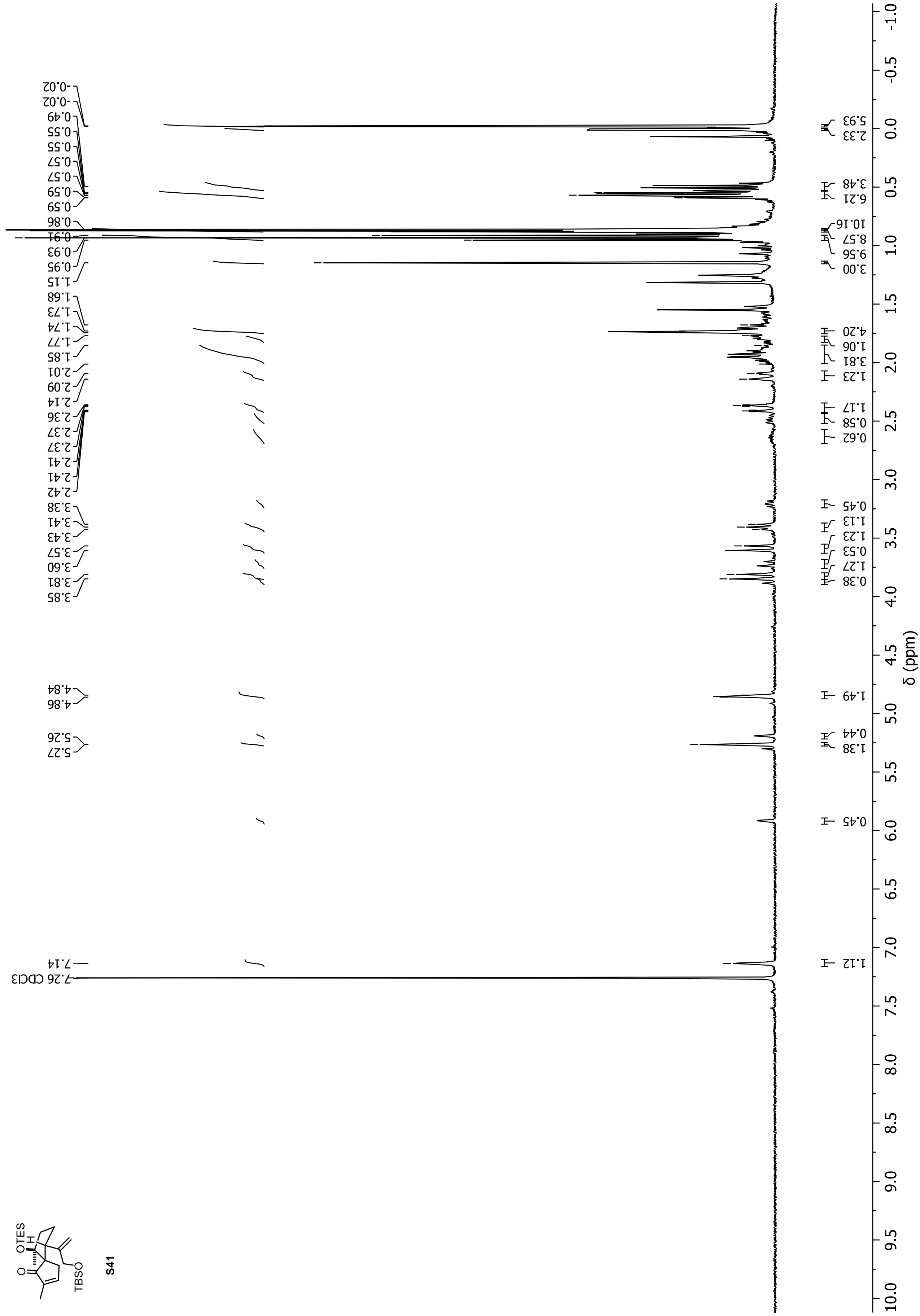

[1]**H NMR spectrum** of enone **S41** (OTBS derivative) measured in $CDCl_3$ at 400 MHz.

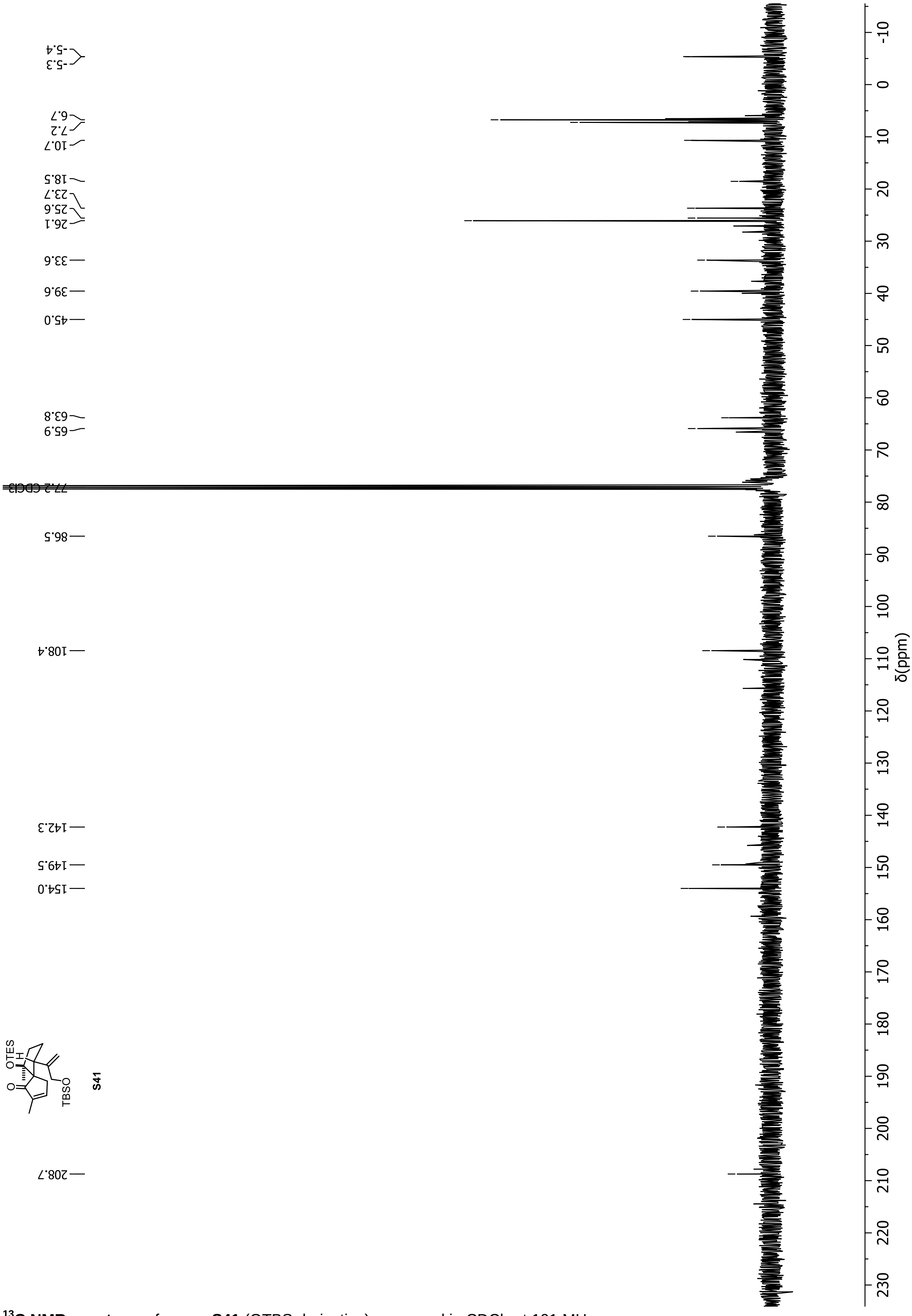

^{13}C NMR spectrum of enone **S41** (OTBS derivative) measured in $CDCl_3$ at 101 MHz.

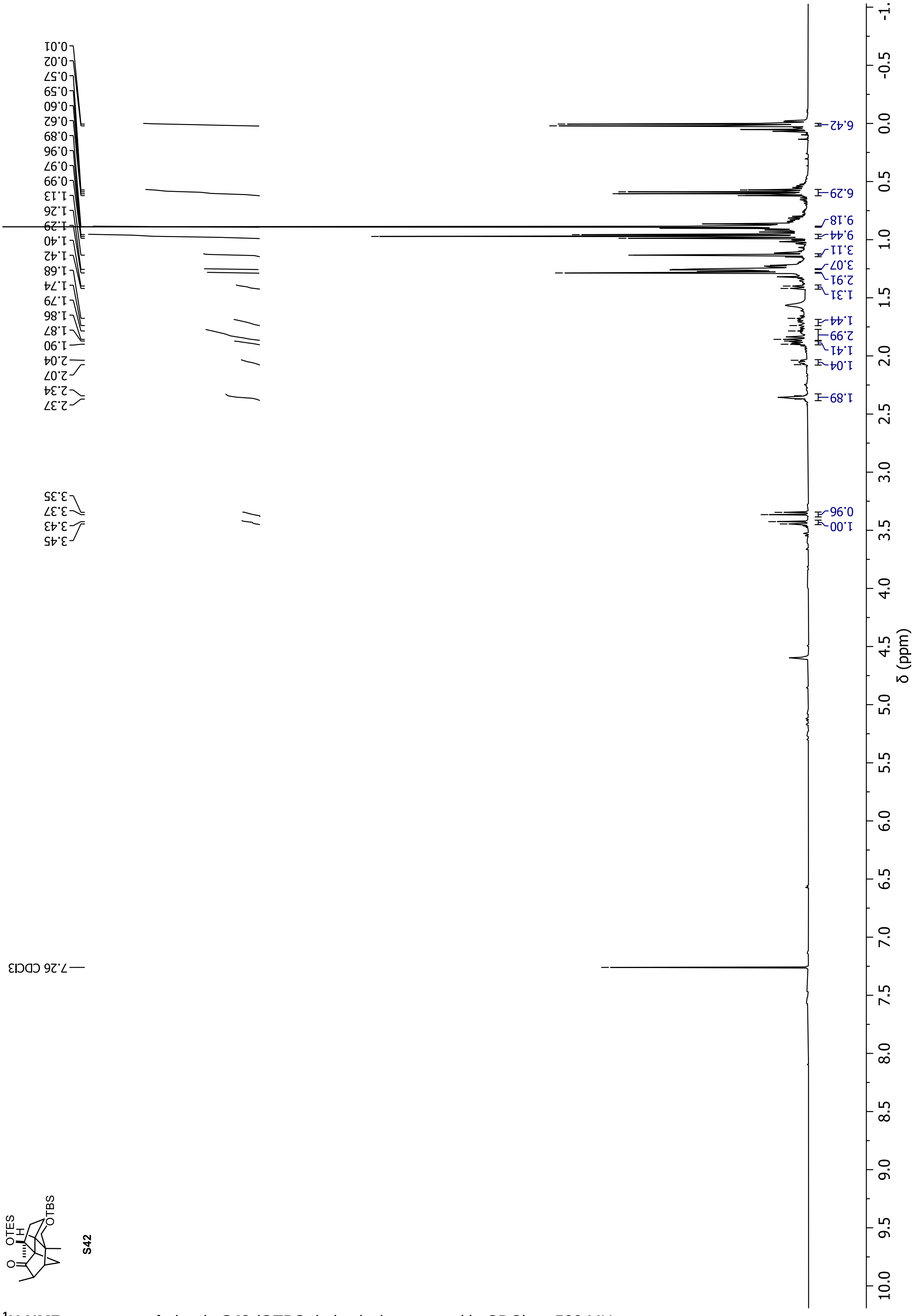

^{1}H NMR spectrum of tricycle **S42** (OTBS derivative) measured in $CDCl_3$ at 500 MHz.

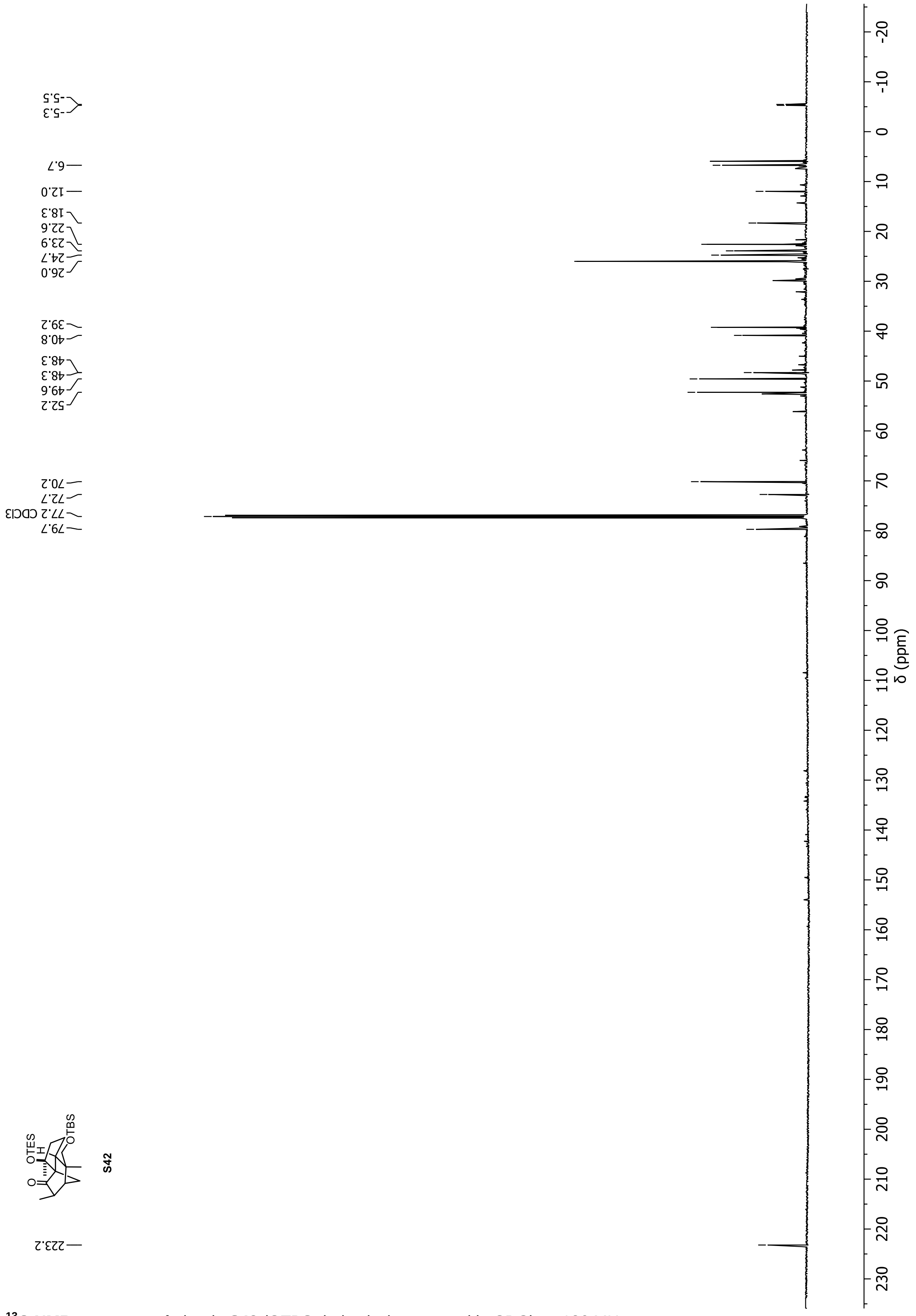

^{13}C NMR spectrum of tricycle **S42** (OTBS derivative) measured in $CDCl_3$ at 126 MHz.

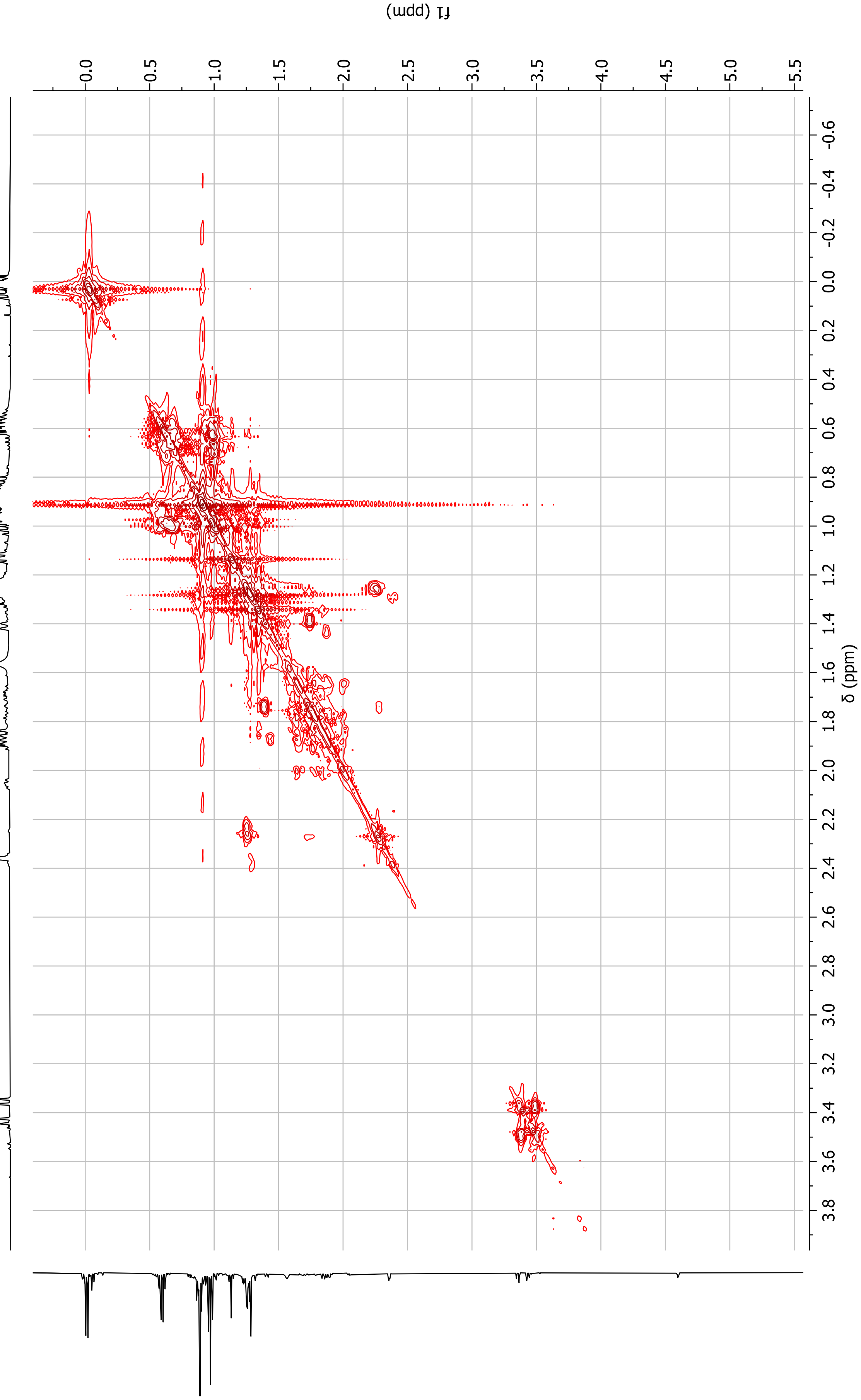

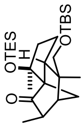

S42

COSY spectrum of tricycle **S42** (OTBS derivative) measured in $CDCl_3$ at 500 MHz.

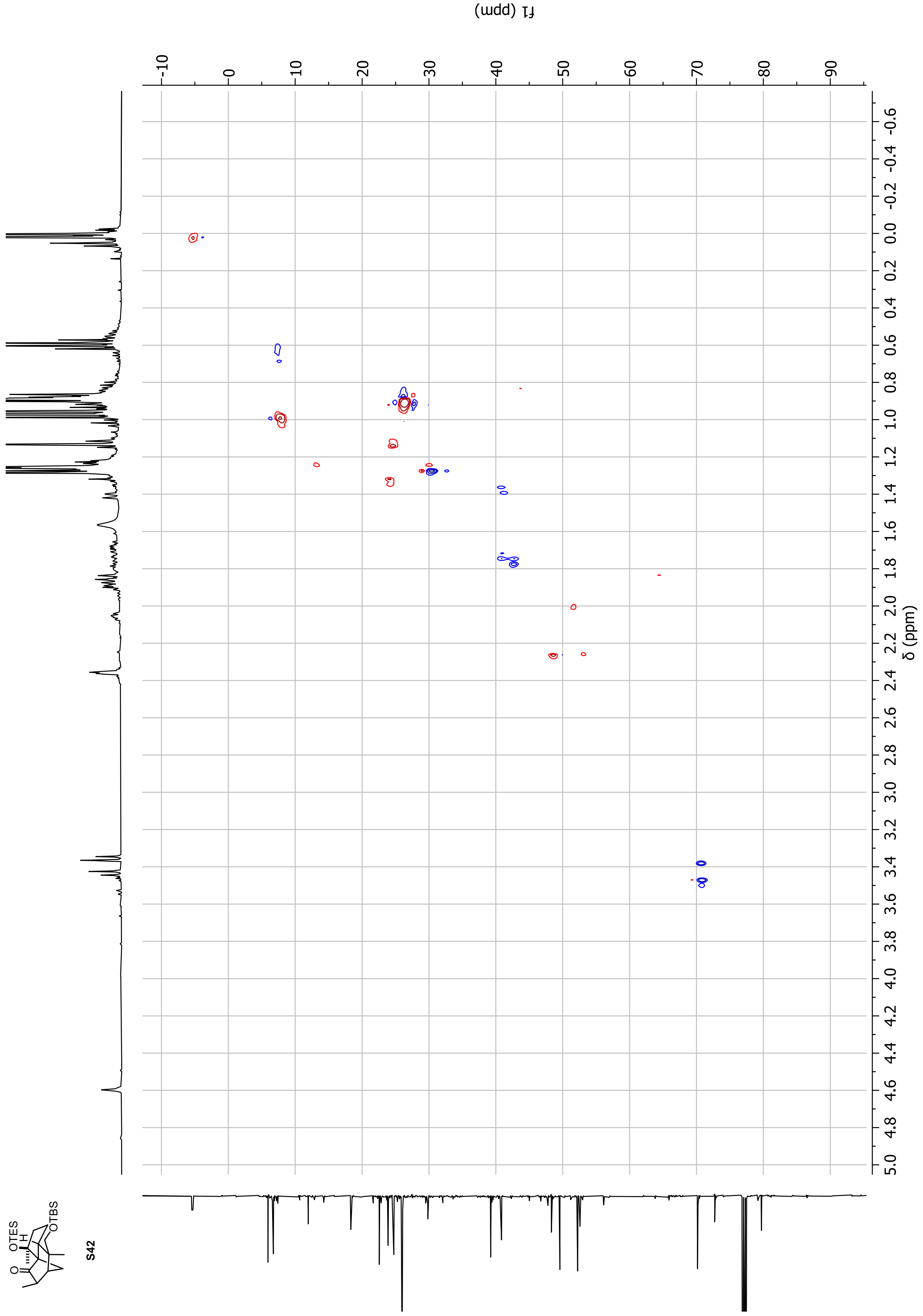

HSQC NMR spectrum of tricycle **S42** (OTBS derivative) measured in $CDCl_3$ at 500 MHz.

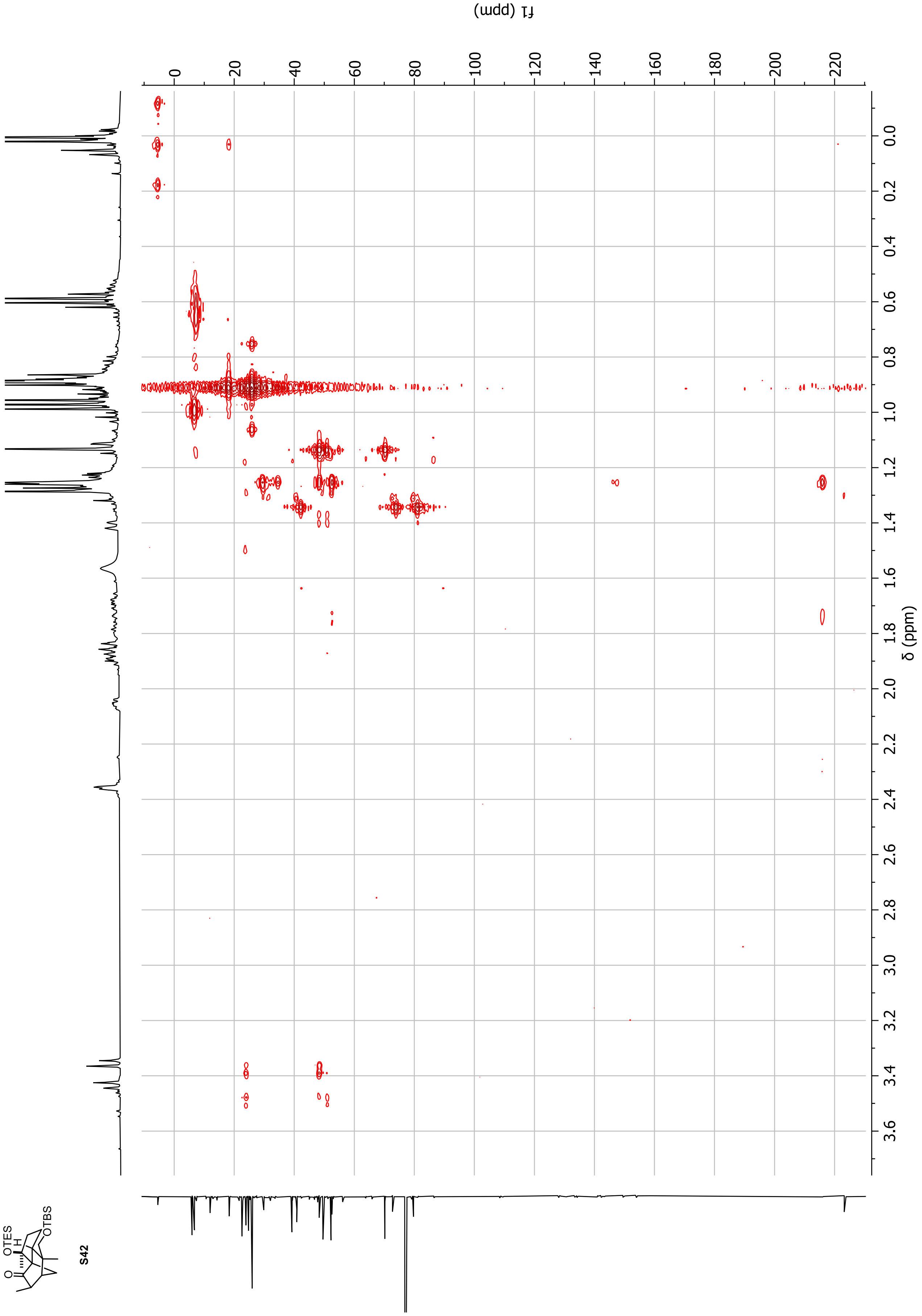

HMBC spectrum of tricycle **S42** (OTBS derivative) measured in $CDCl_3$ at 500 MHz.

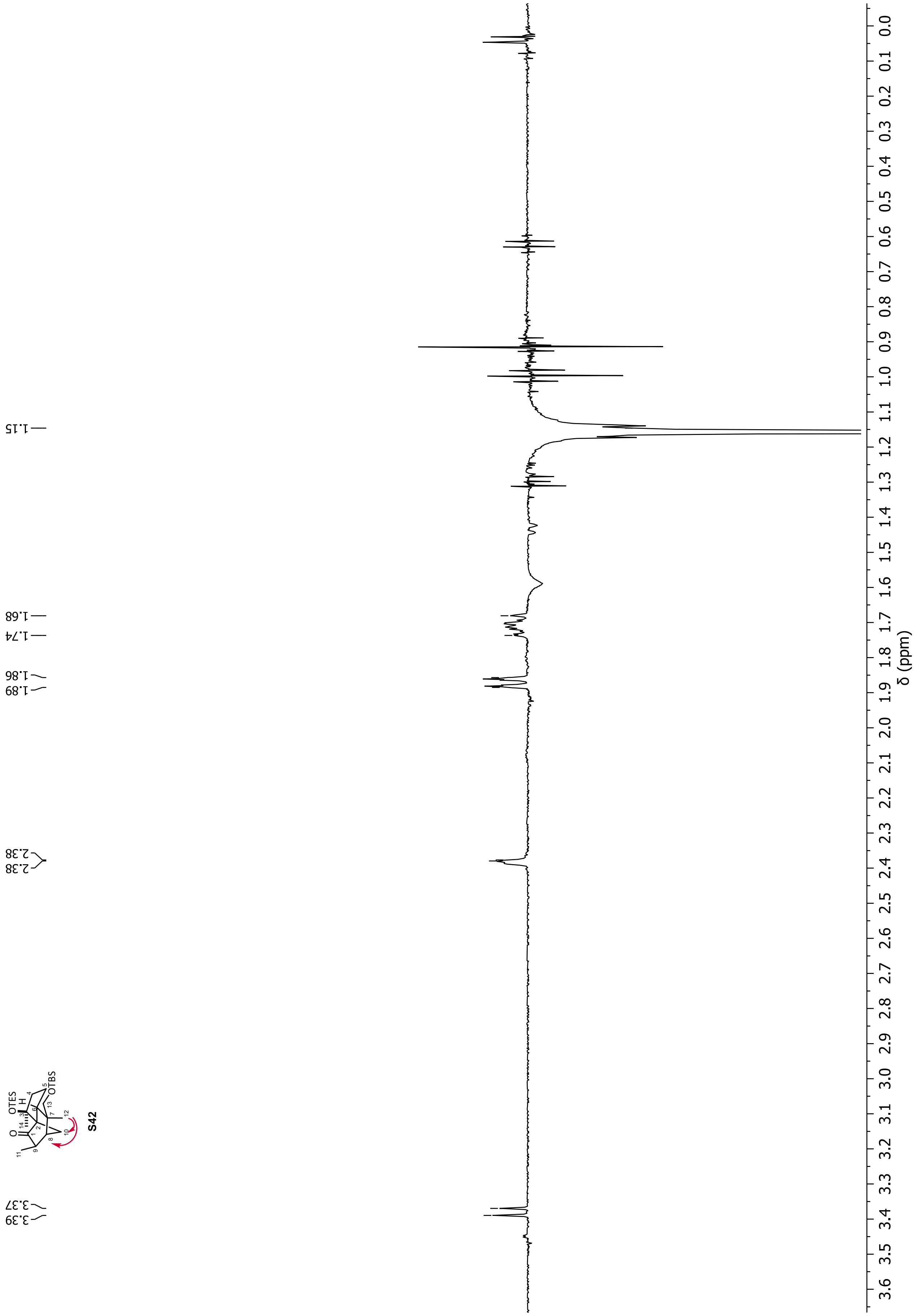

^{1}H NOE spectrum of tricycle **S42** (OTBS derivative) after irradiation at 1.15 ppm (CH_2-12) measured in $CDCl_3$ at 400 MHz.

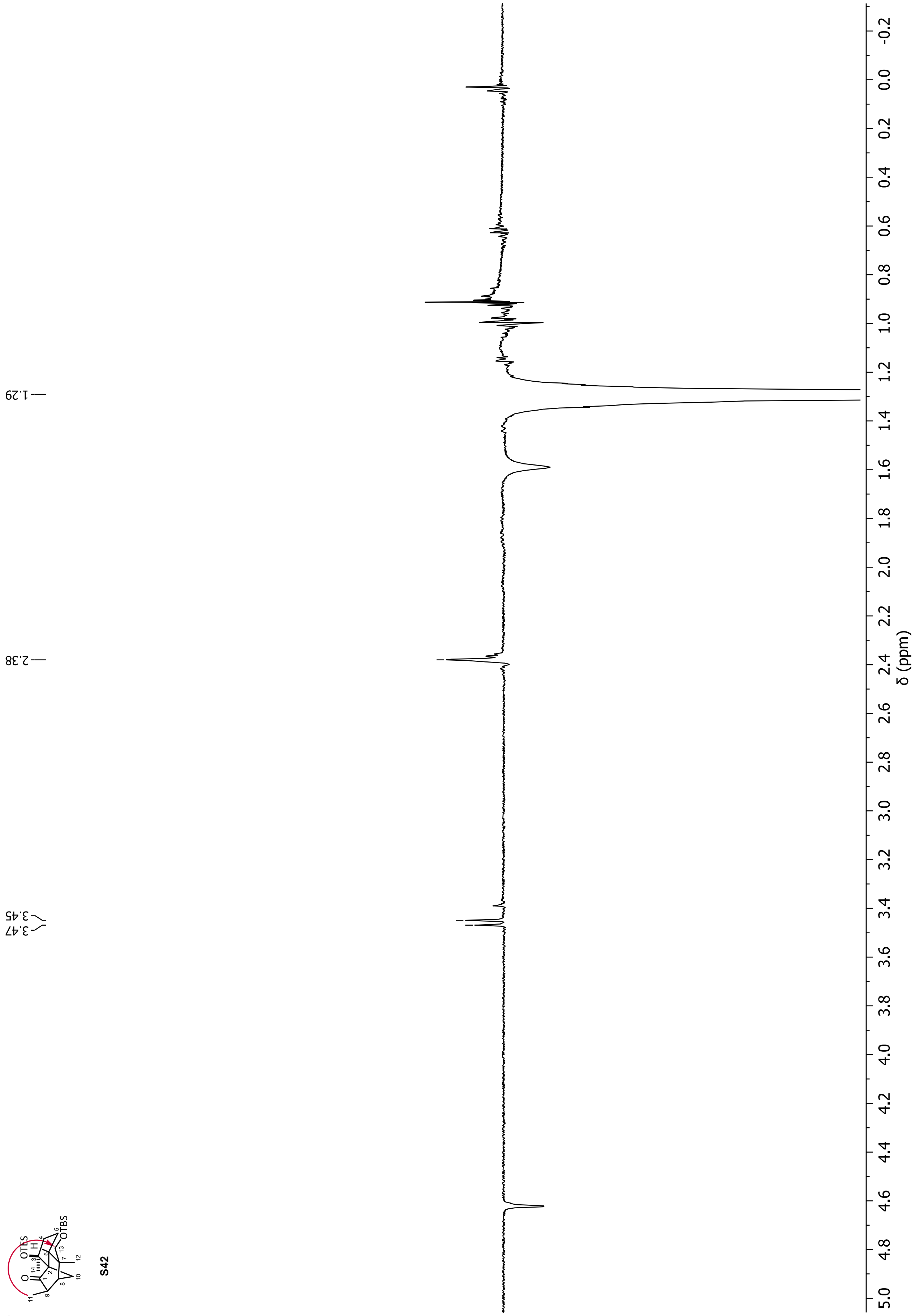

^{1}H NOE spectrum of tricycle **S42** (OTBS derivative) after irradiation at 1.29 ppm (CH_2-11) measured in $CDCl_3$ at 400 MHz.

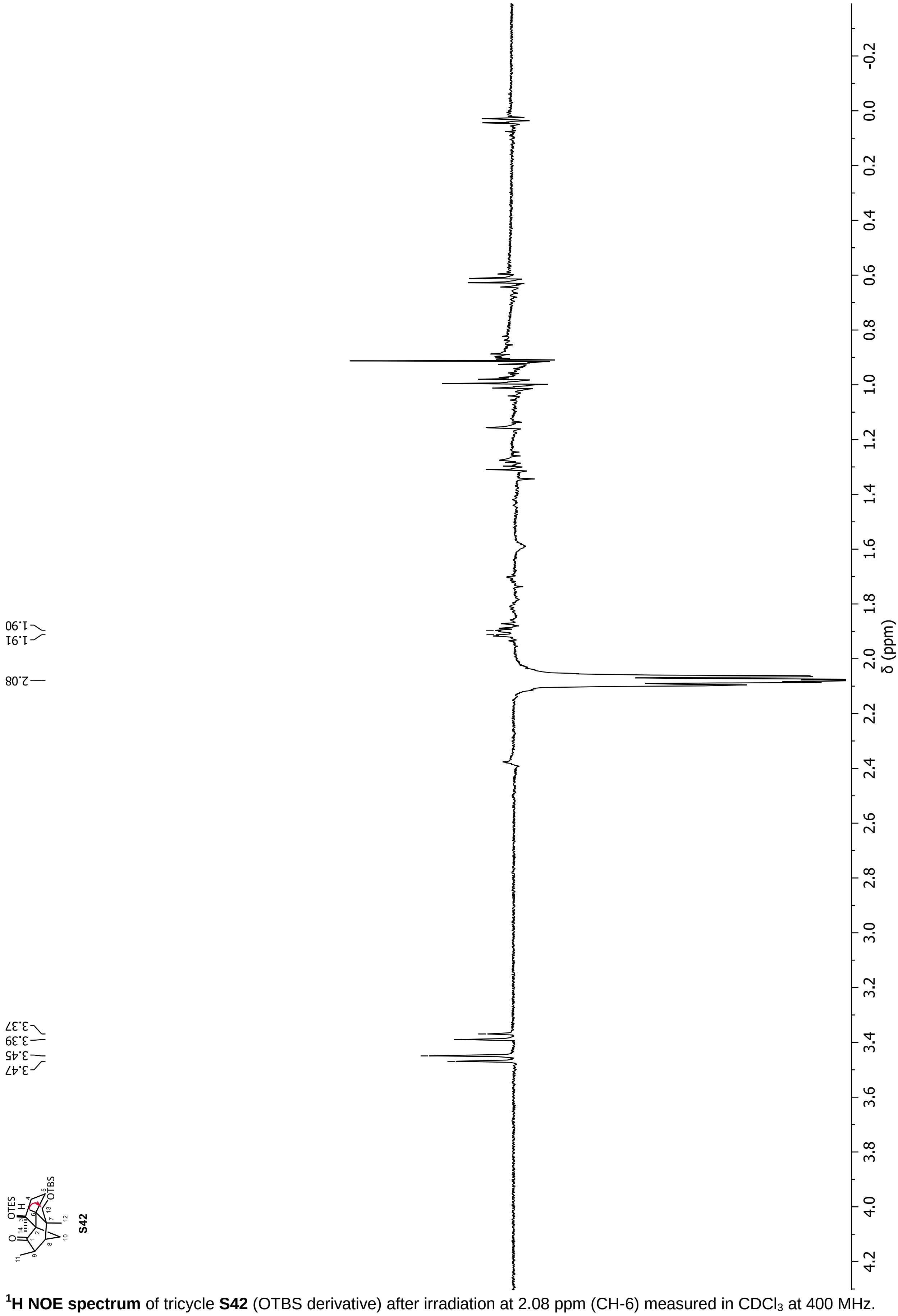

^{1}H NOE spectrum of tricycle **S42** (OTBS derivative) after irradiation at 2.08 ppm (CH-6) measured in $CDCl_3$ at 400 MHz.

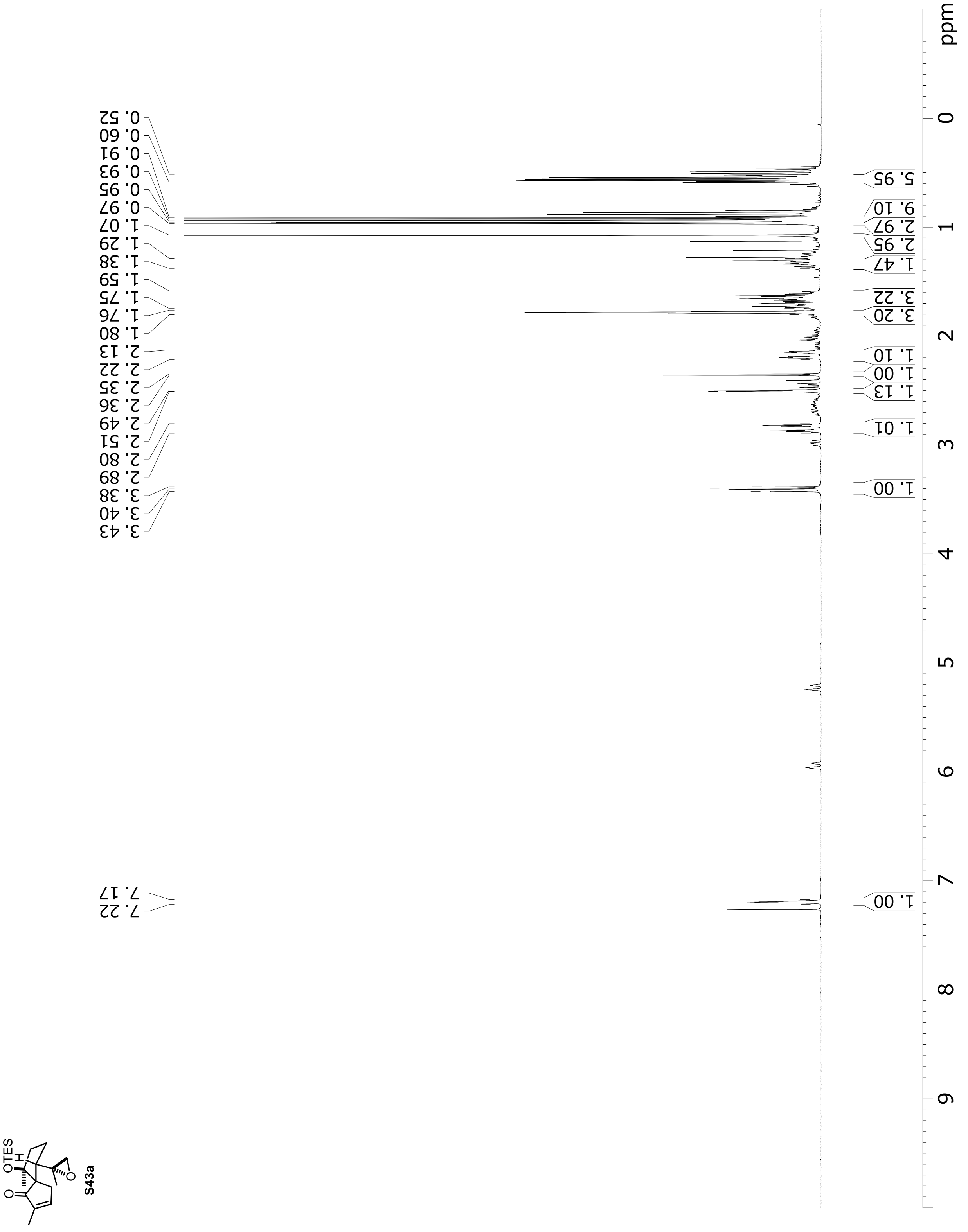

[1]**H NMR spectrum** of epoxide **S43a** measured in $CDCl_3$ at 400 MHz. Epoxide **S43a** was obtained as an inseparable mixture with two other compounds which we identified as the epoxides of *exo*-**S36:**

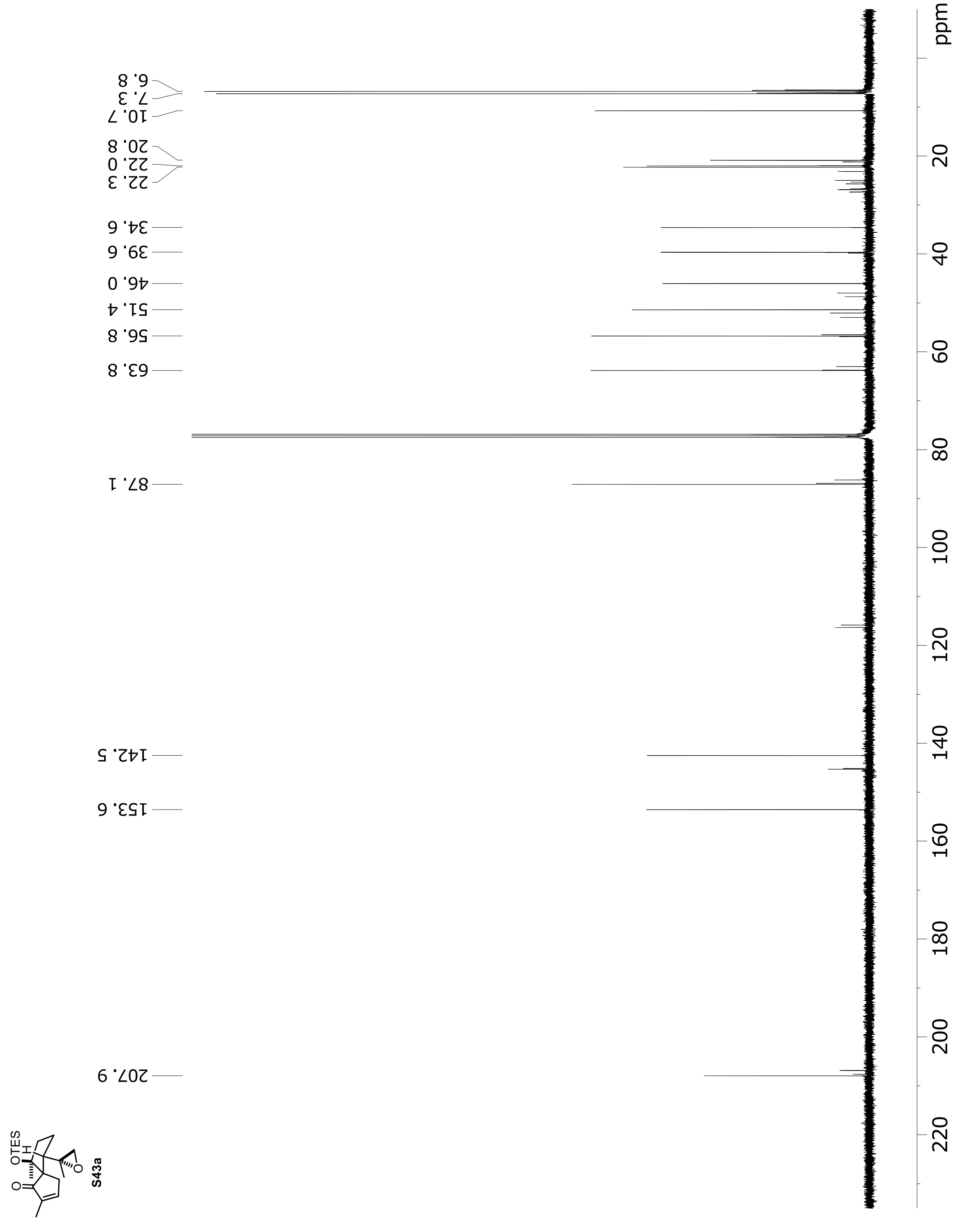

^{13}C NMR spectrum of epoxide **S43a** measured in $CDCl_3$ at 101 MHz. Epoxide **S43a** was obtained as an inseparable mixture with two other compounds which we identified as the epoxides of *exo*-**S36:**

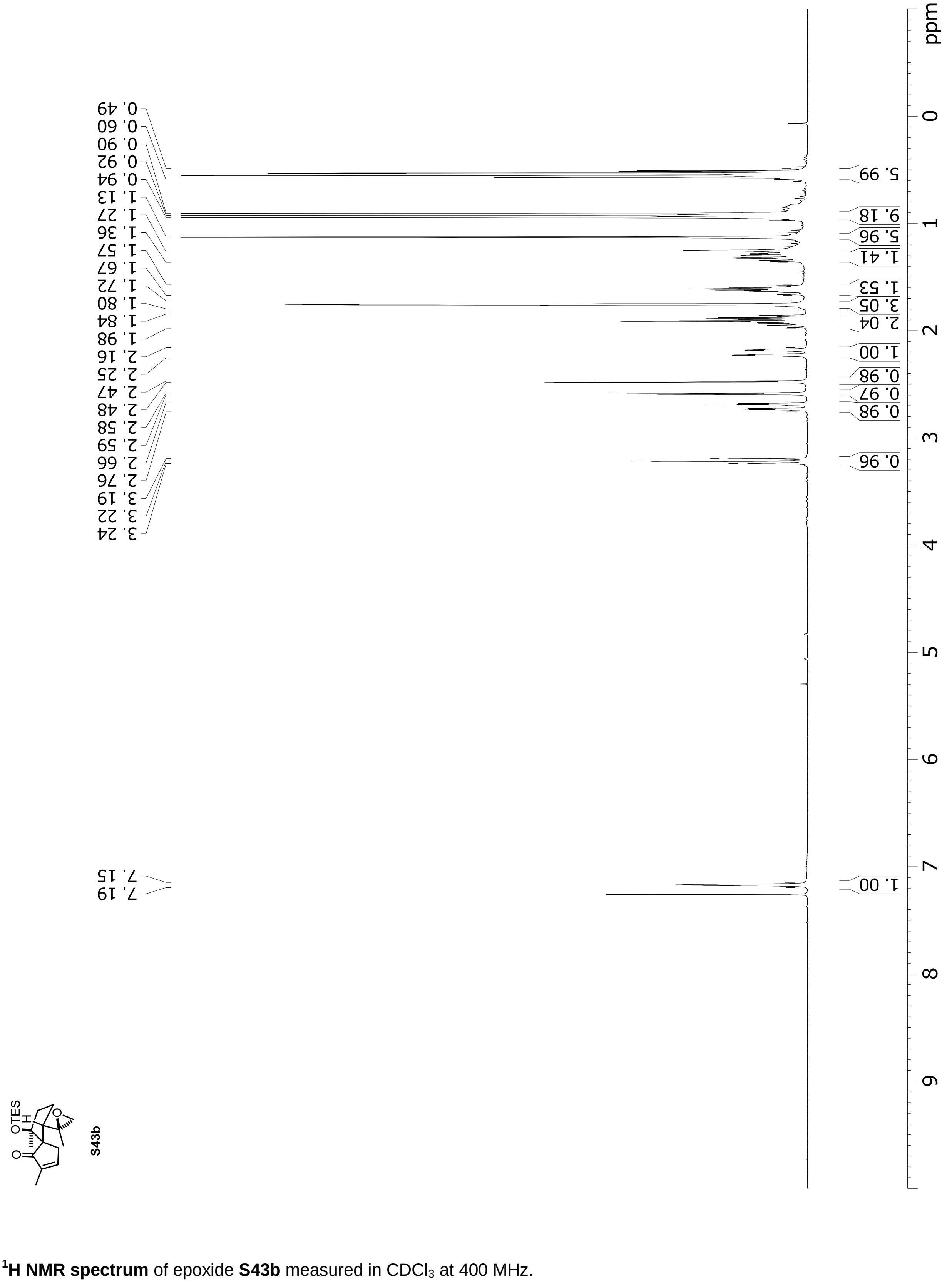

^{1}H NMR spectrum of epoxide **S43b** measured in $CDCl_3$ at 400 MHz.

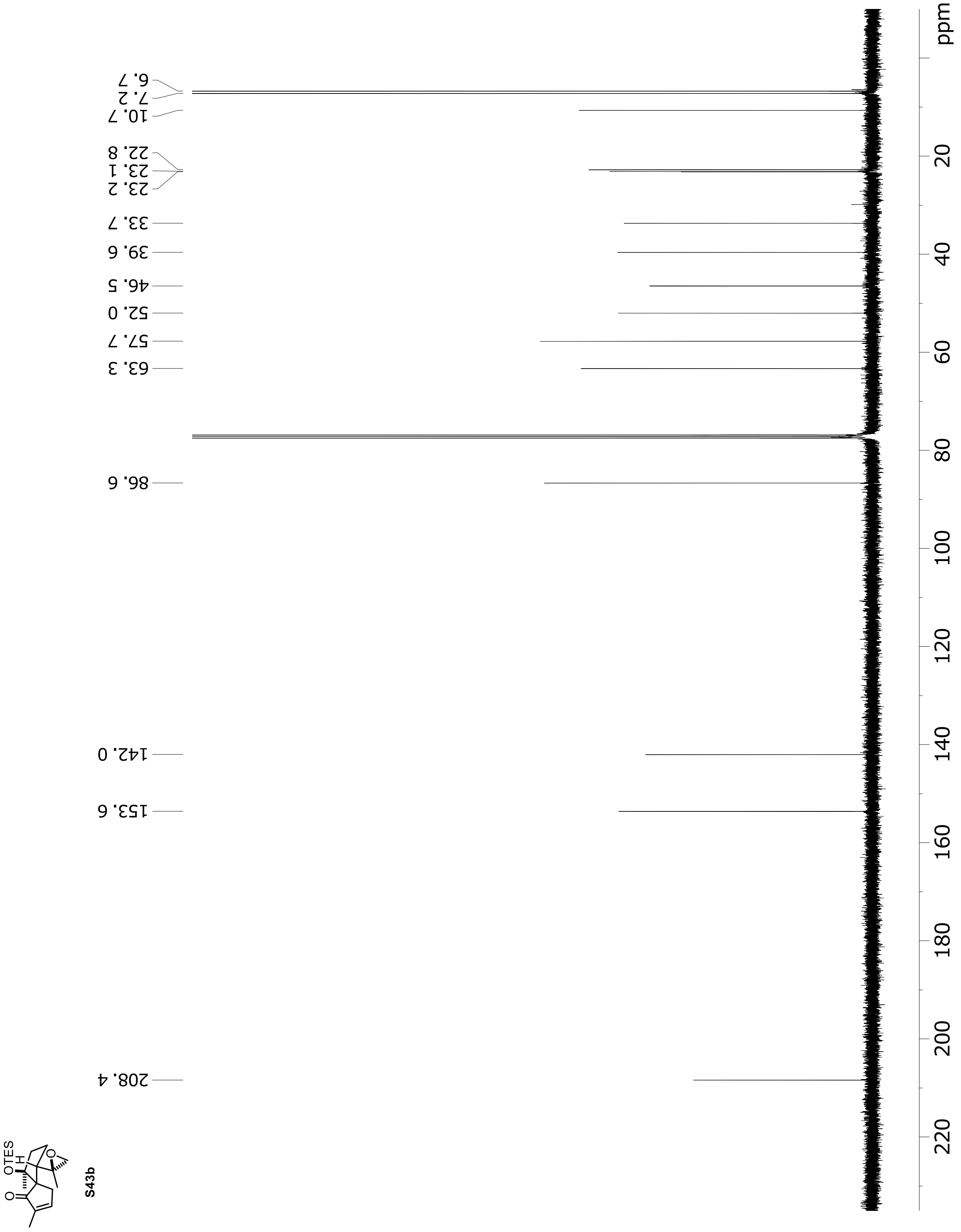

^{13}**C NMR spectrum** of epoxide **S43b** measured in $CDCl_3$ at 101 MHz.

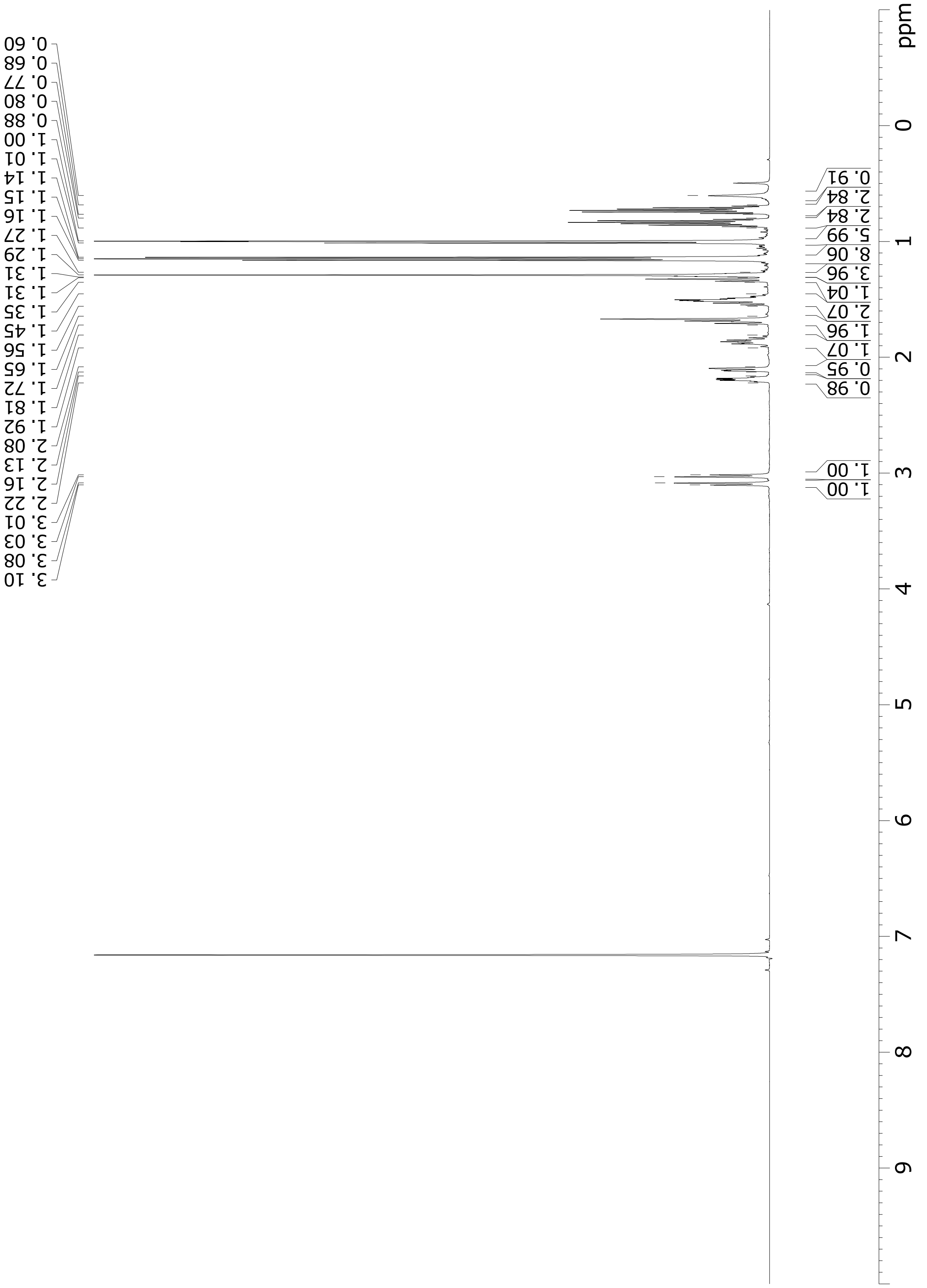

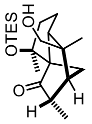

S44

^{1}H NMR spectrum of cyclization product S44 measured in C_6D_6 at 600 MHz.

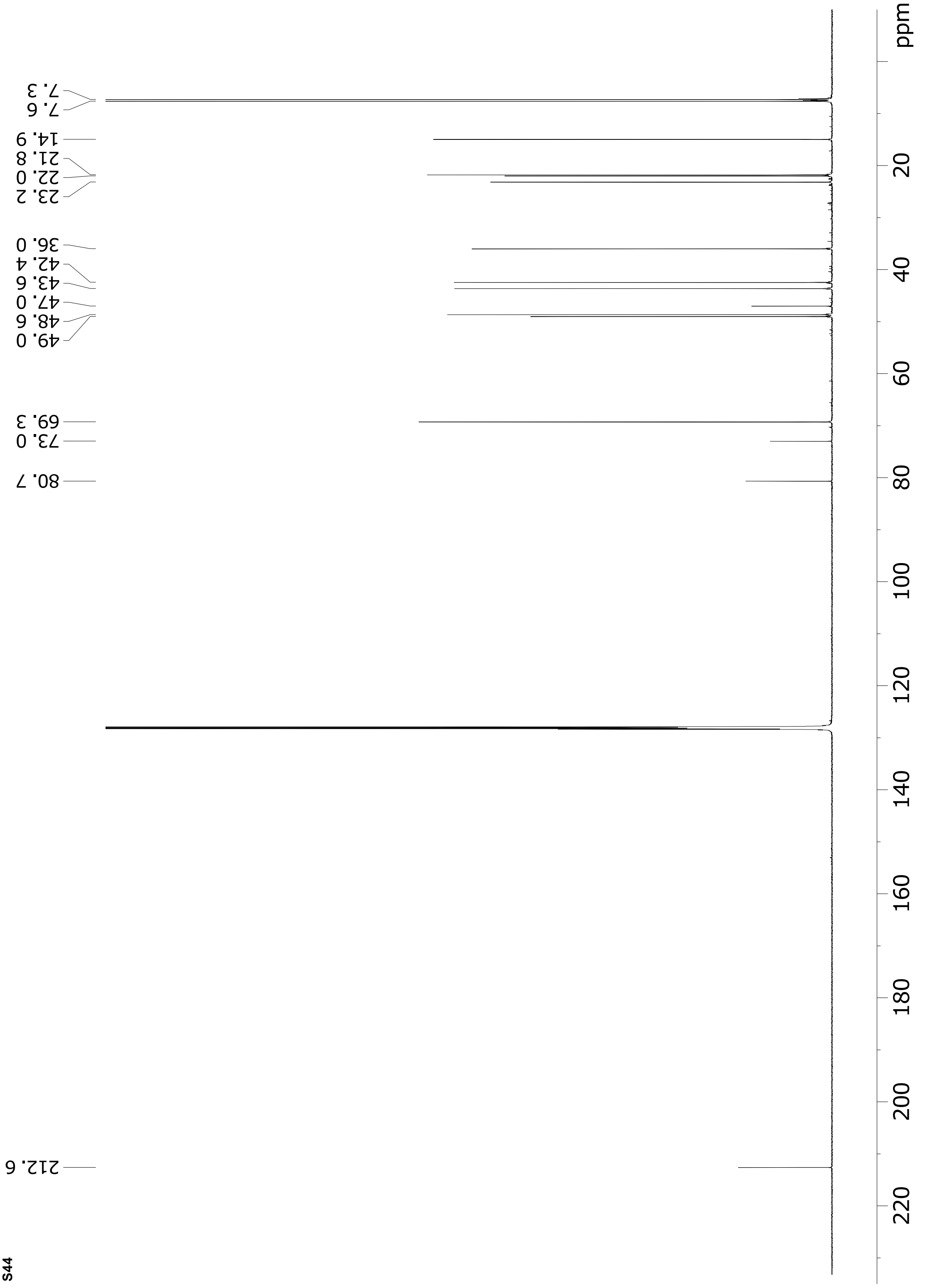

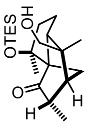

S44

^{13}C NMR spectrum of cyclization product **S44** measured in C_6D_6 at 151 MHz.

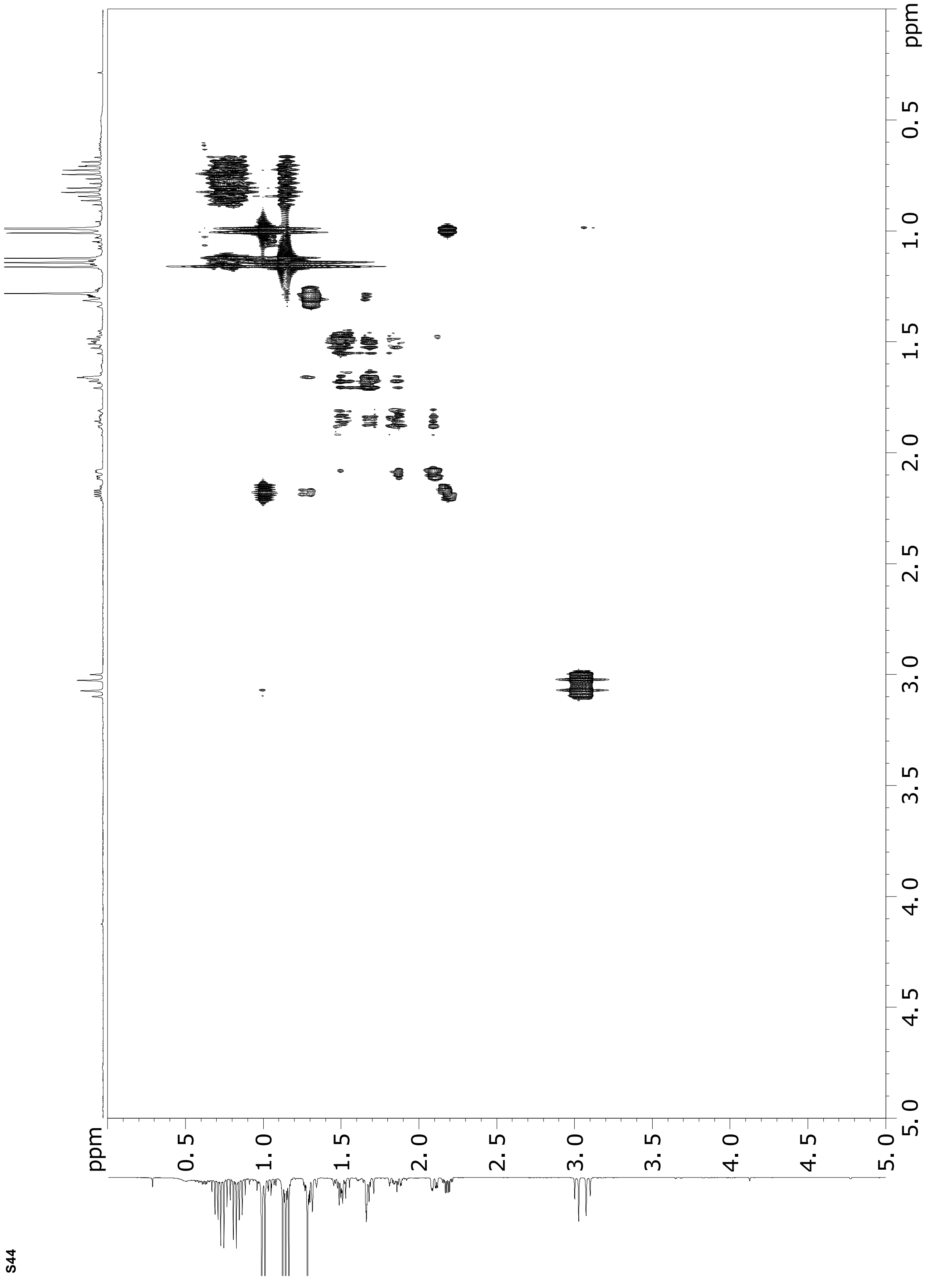

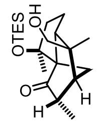

S44

COSY spectrum of cyclization product **S44** measured in C_6D_6 at 600 MHz.

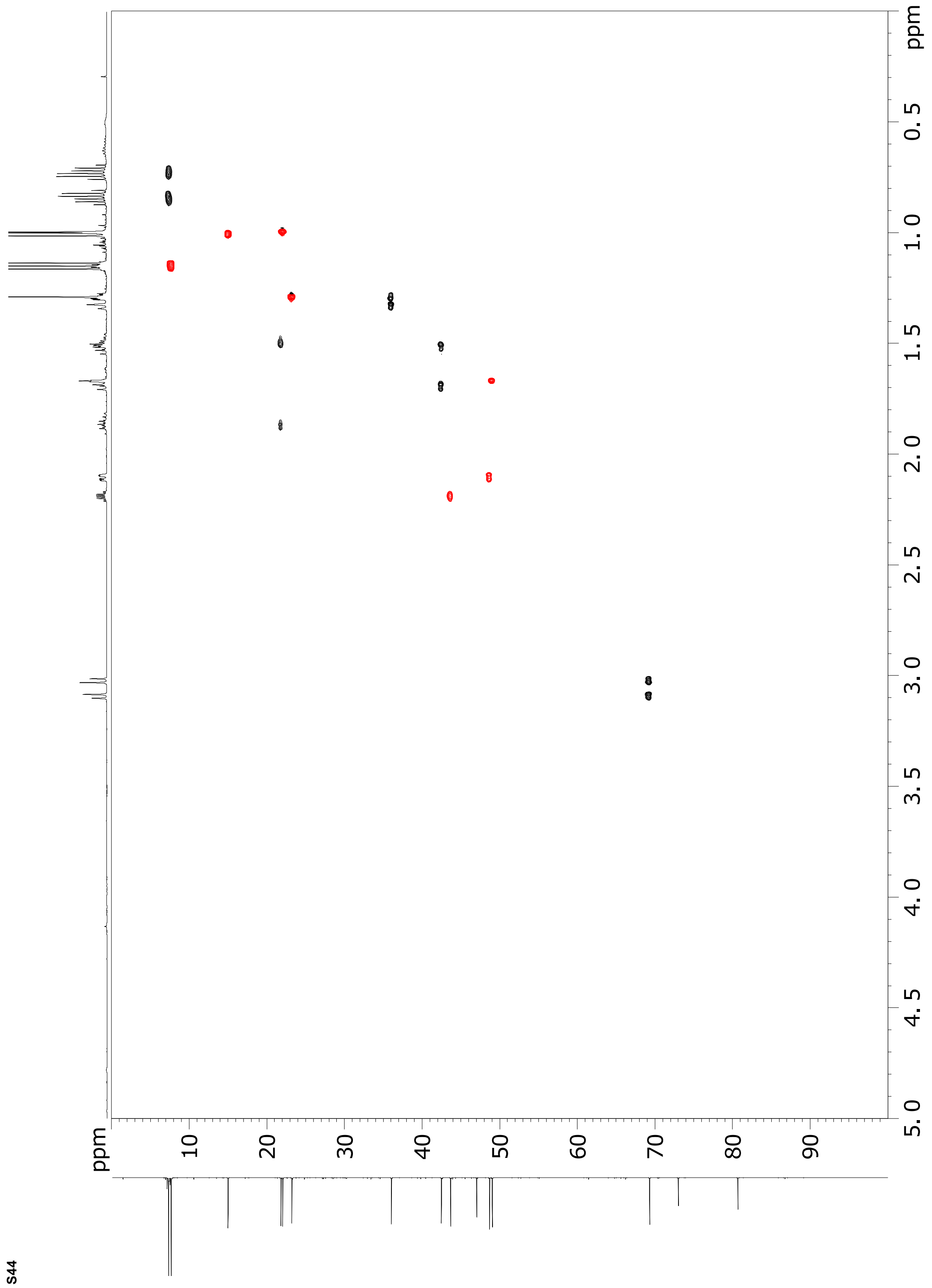

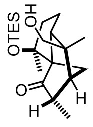

S44

HSQC spectrum of cyclization product **S44** measured in C_6D_6 at 600 MHz.

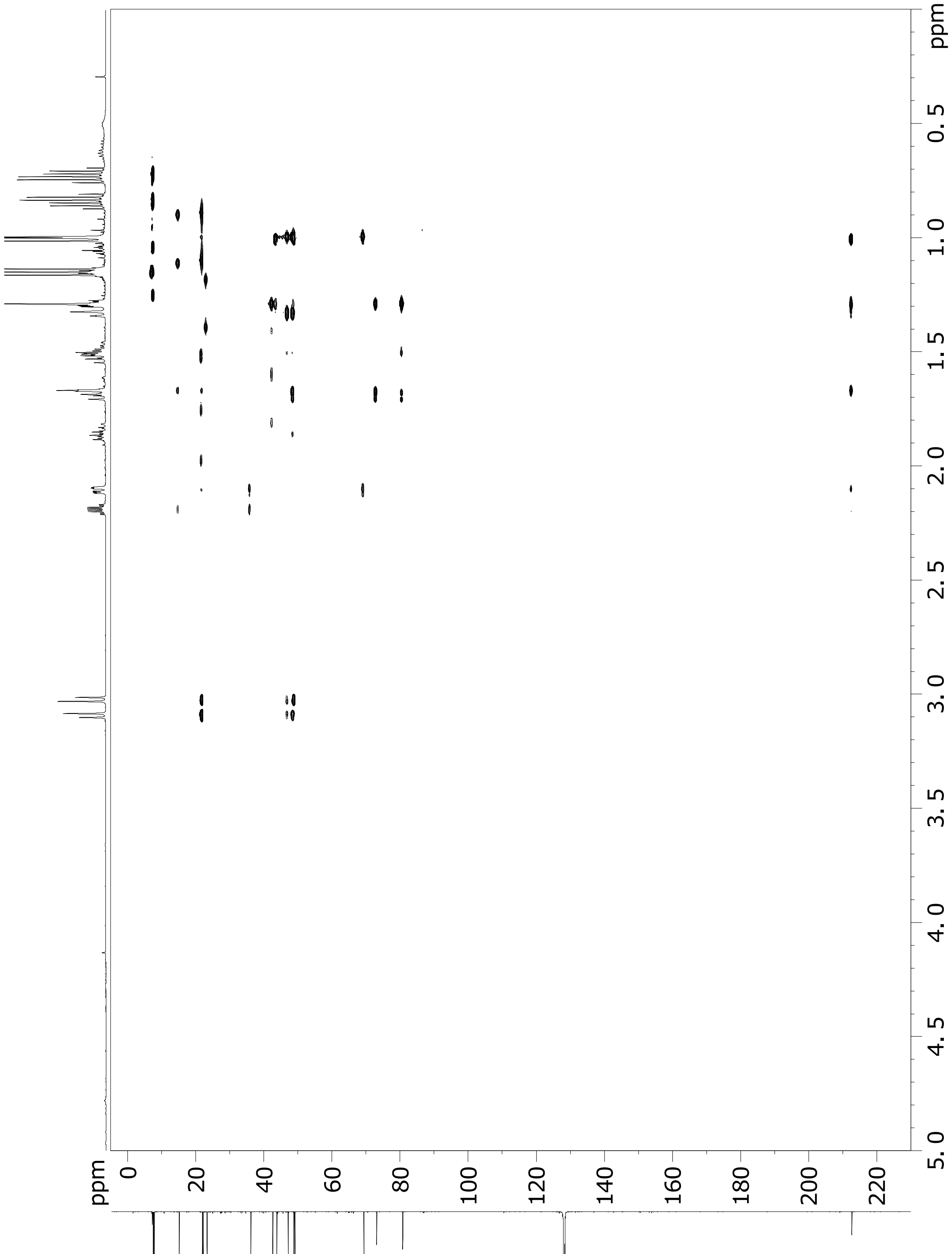

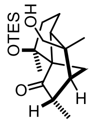

S44

HMBC spectrum of cyclization product **S44** measured in C_6D_6 at 600 MHz.

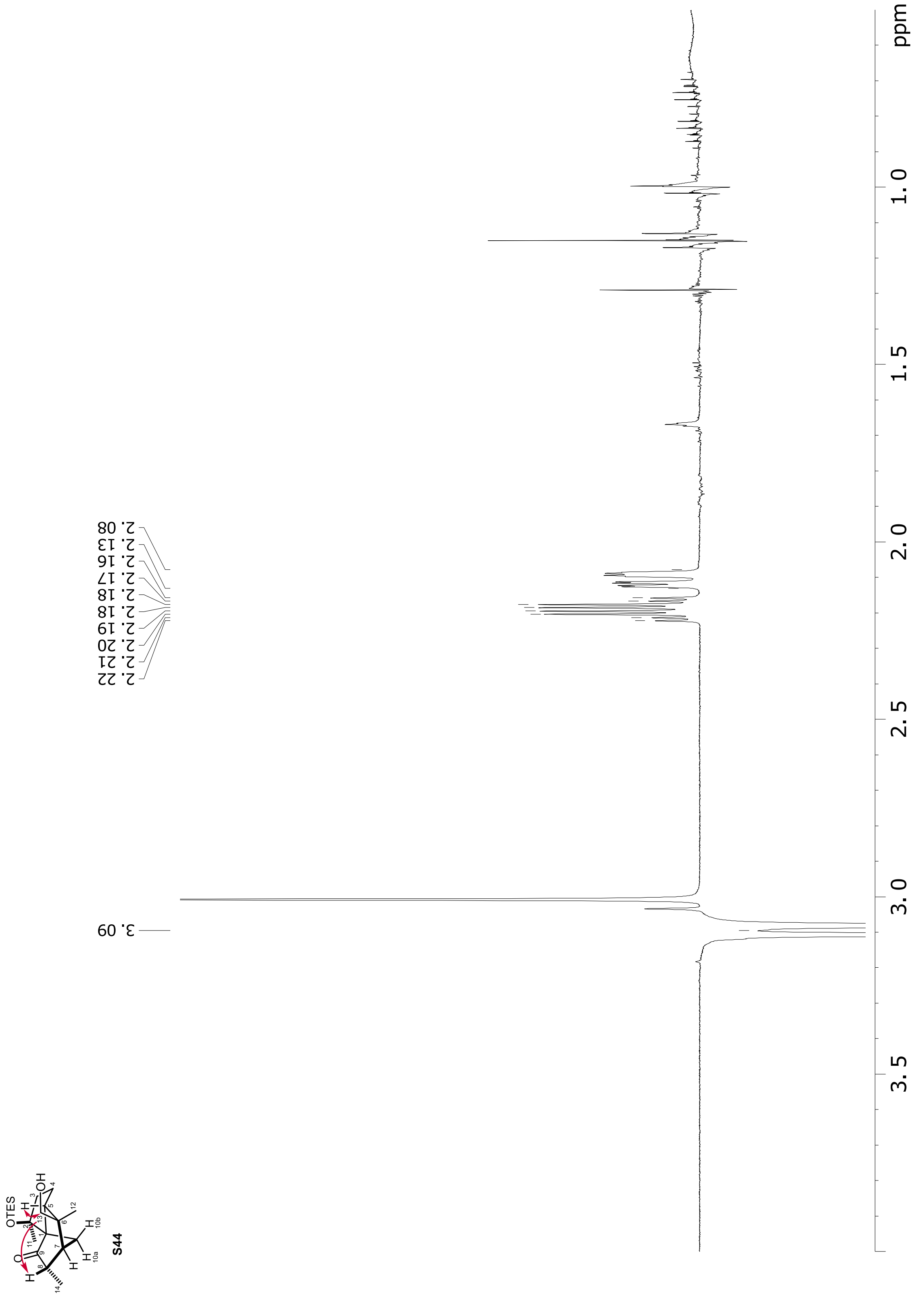

^{1}H NOE spectrum of cyclization product **S44** after irradiation at 3.09 ppm (CH_2-13a) measured in C_6D_6 at 400 MHz.

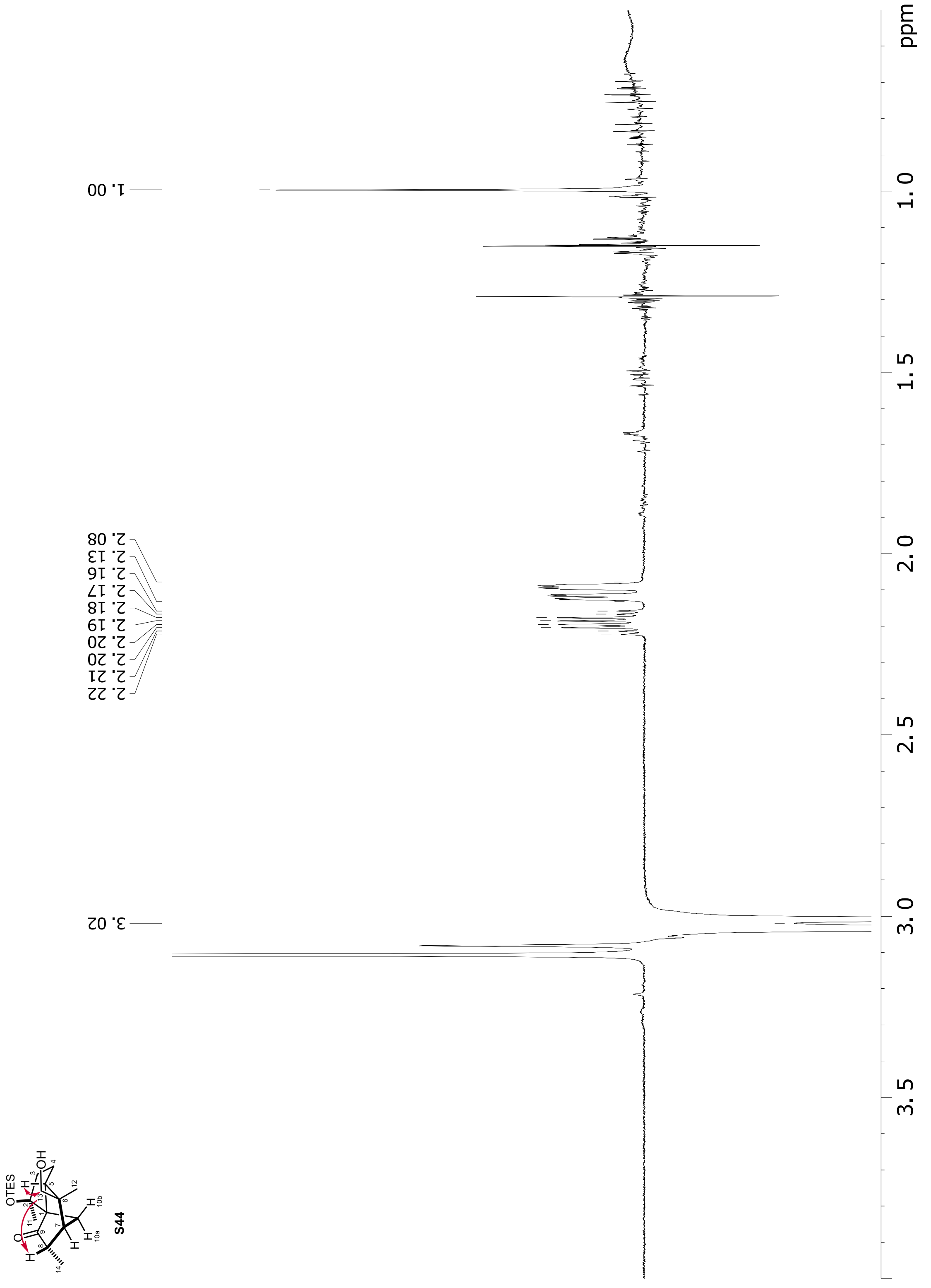

^{1}H NOE spectrum of cyclization product **S44** after irradiation at 3.02 ppm (CH_2-13b) measured in C_6D_6 at 400 MHz.

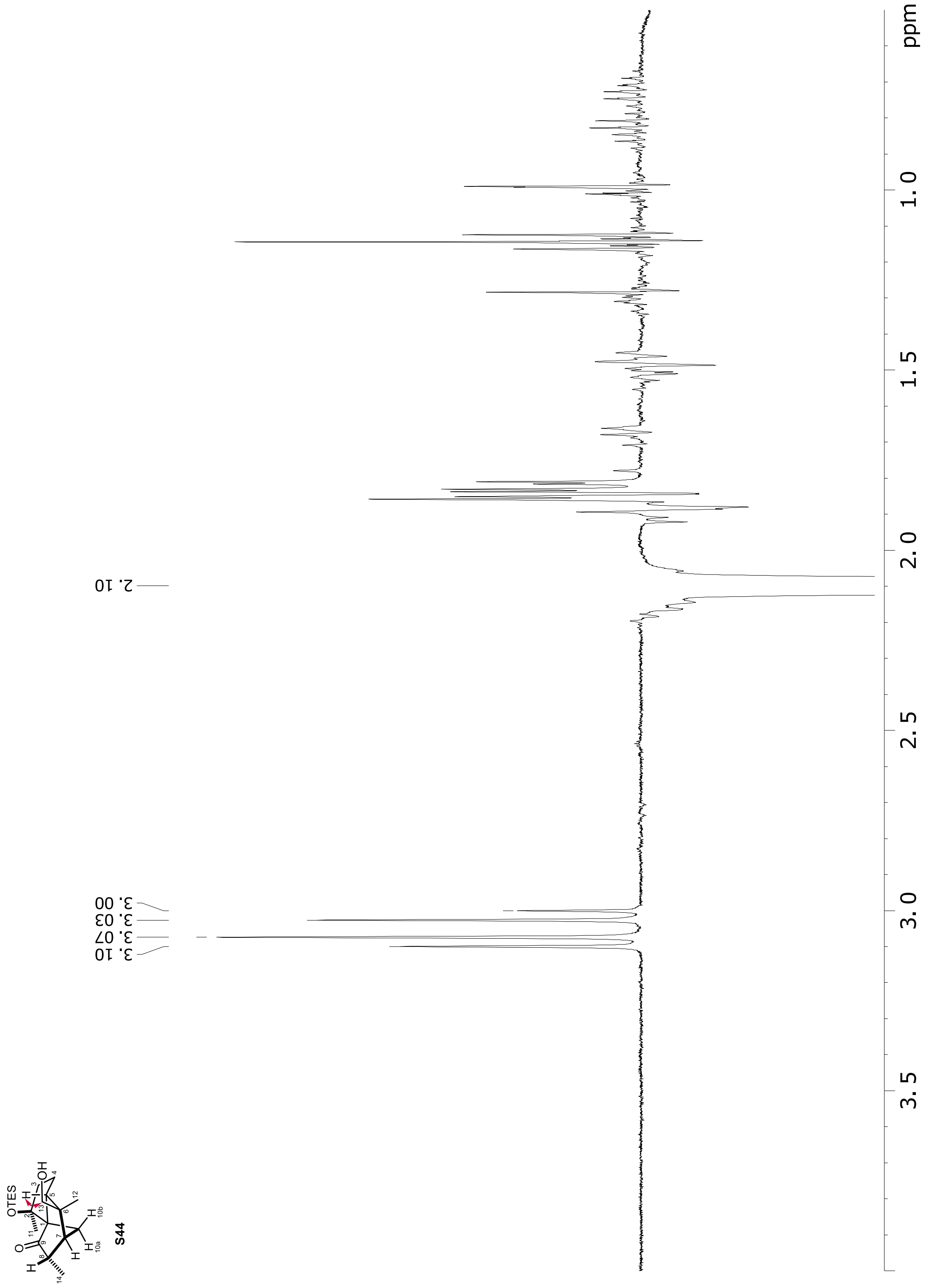

^{1}H NOE spectrum of cyclization product **S44** after irradiation at 2.10 ppm (CH-5) measured in C_6D_6 at 400 MHz.

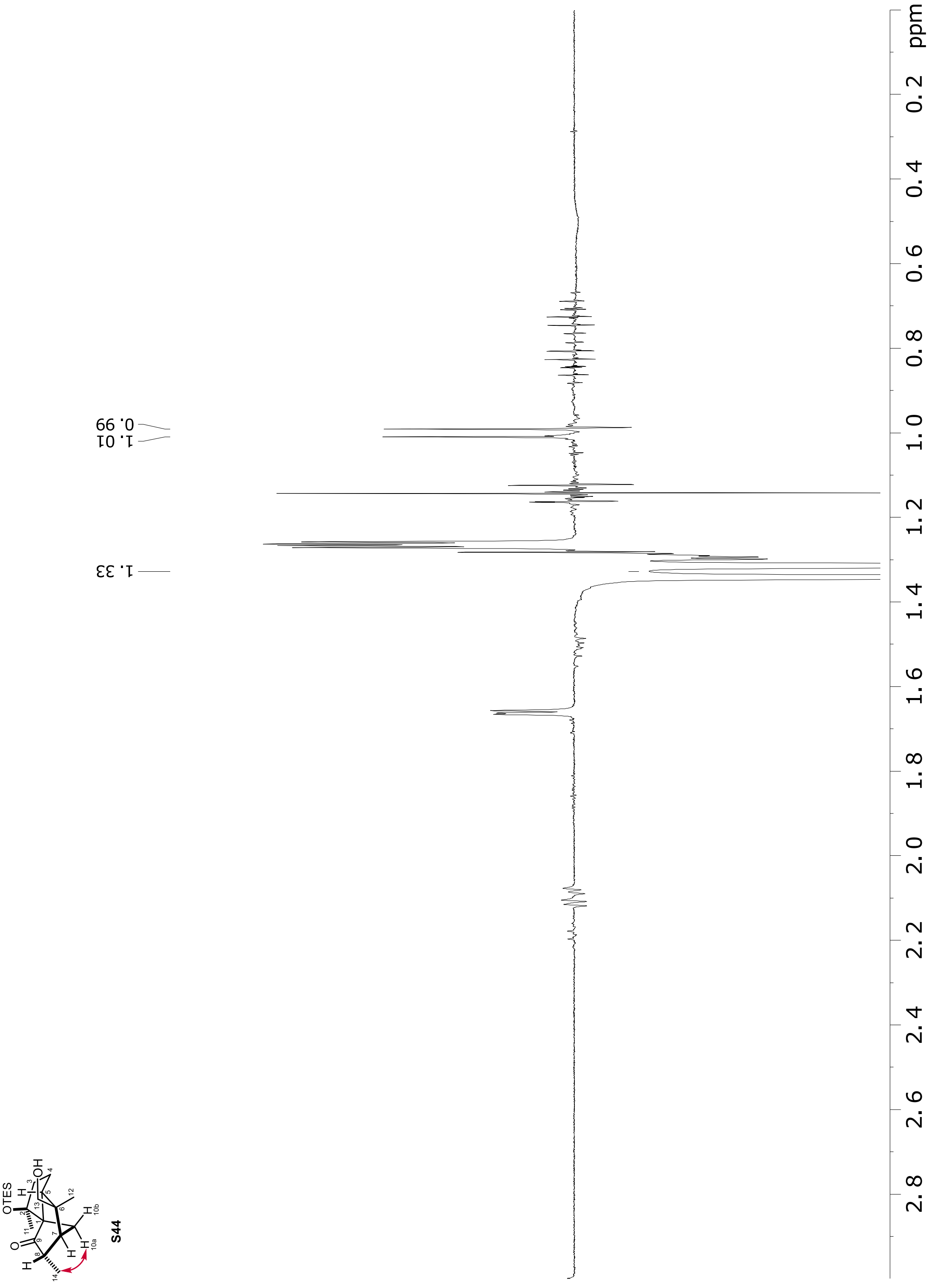

^{1}**H NOE spectrum** of cyclization product **S44** after irradiation at 1.33 ppm (CH_2-10a) measured in C_6D_6 at 400 MHz.

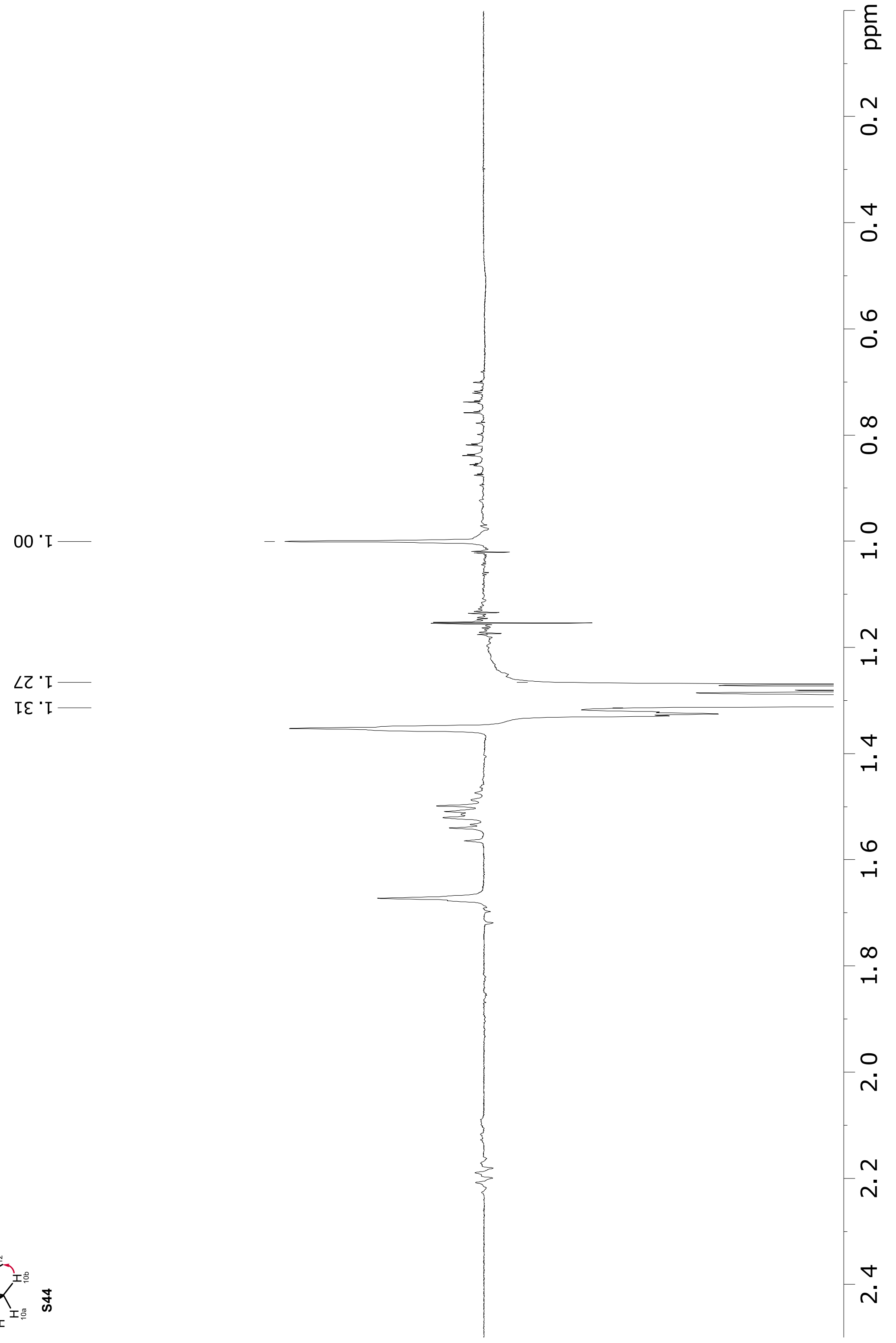

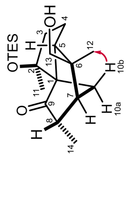

S44

^{1}H NOE spectrum of cyclization product **S44** after irradiation at 1.27 ppm (CH_2-10b) measured in C_6D_6 at 400 MHz.

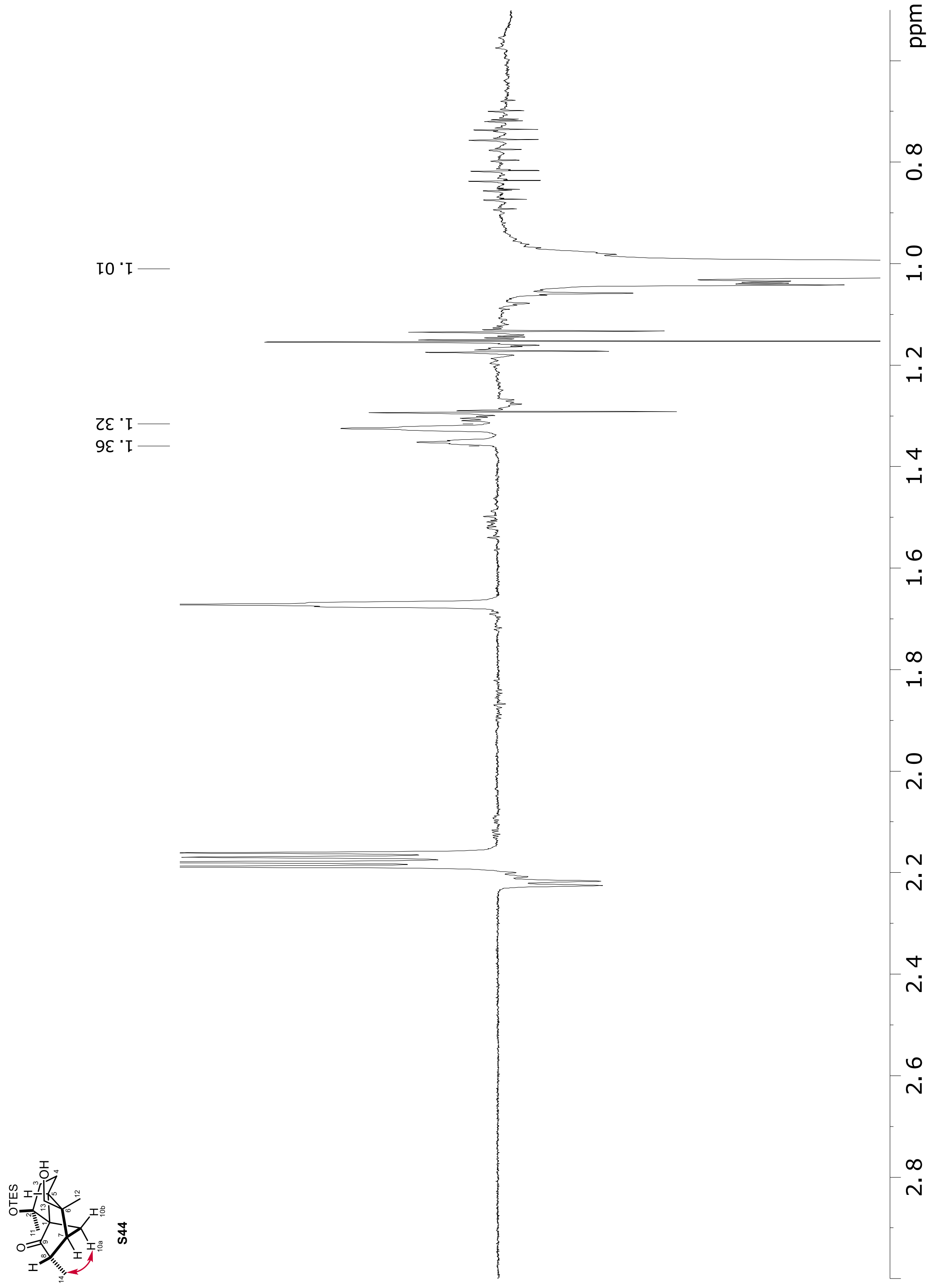

^{1}H NOE spectrum of cyclization product **S44** after irradiation at 1.01 ppm (CH_3-14) measured in C_6D_6 at 400 MHz.

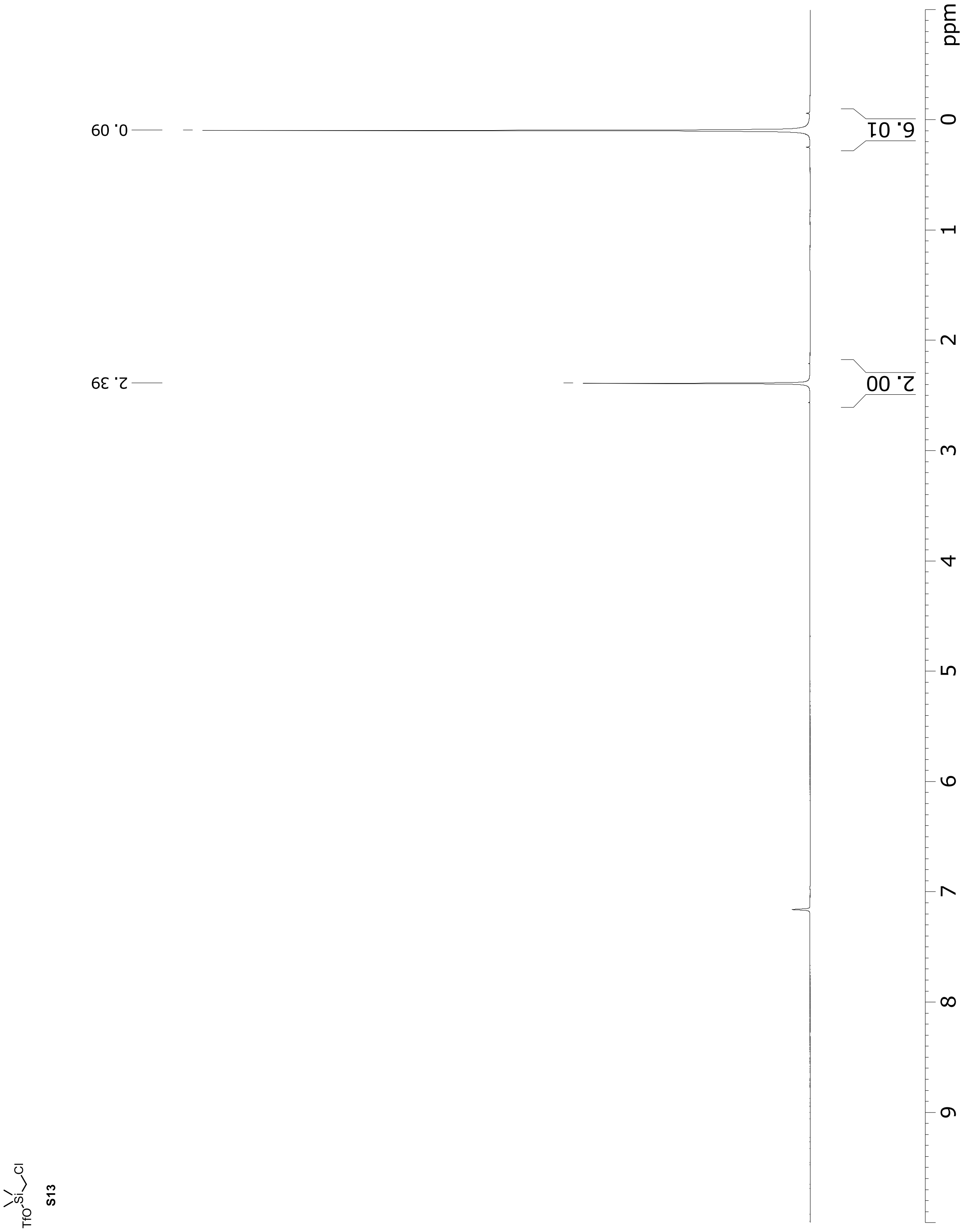

^{1}H NMR spectrum of (chlolomethyl)dimethylsilyl trifluoromethanesulfonate (**S13**) measured in C_6D_6 at 400 MHz.

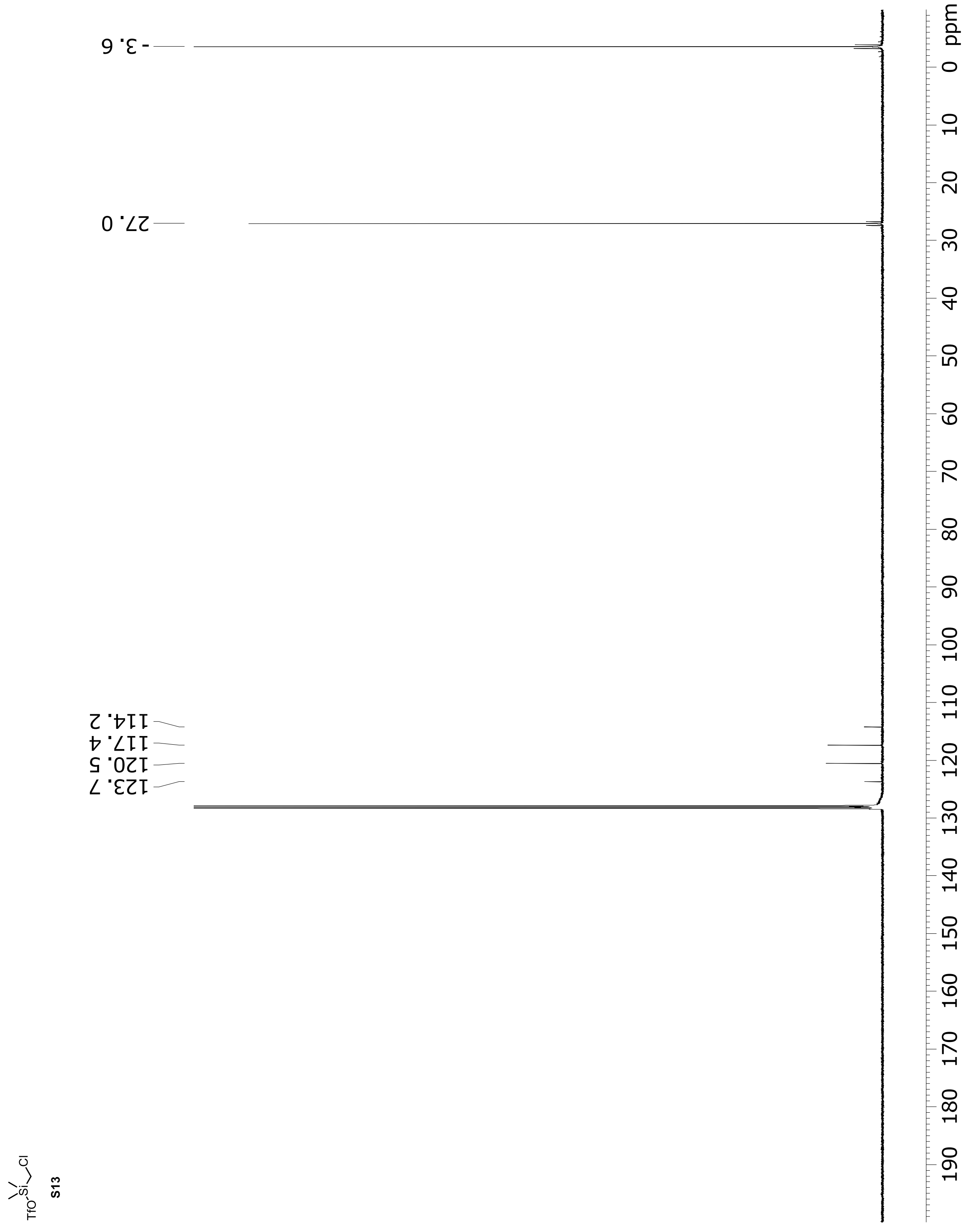

^{13}C NMR spectrum of (chlolomethyl)dimethylsilyl trifluoromethanesulfonate (**S13**) measured in C_6D_6 at 101 MHz.
